# Cryogenic Organometallic Carbon–Fluoride Bond Functionalization with Broad Functional Group Tolerance

**DOI:** 10.1021/jacs.4c13956

**Published:** 2025-02-06

**Authors:** D. Lucas Kane, Bryan C. Figula, Kaluvu Balaraman, Jeffery A. Bertke, Christian Wolf

**Affiliations:** Georgetown University, Chemistry Department, Washington, D.C. 20057, United States

## Abstract

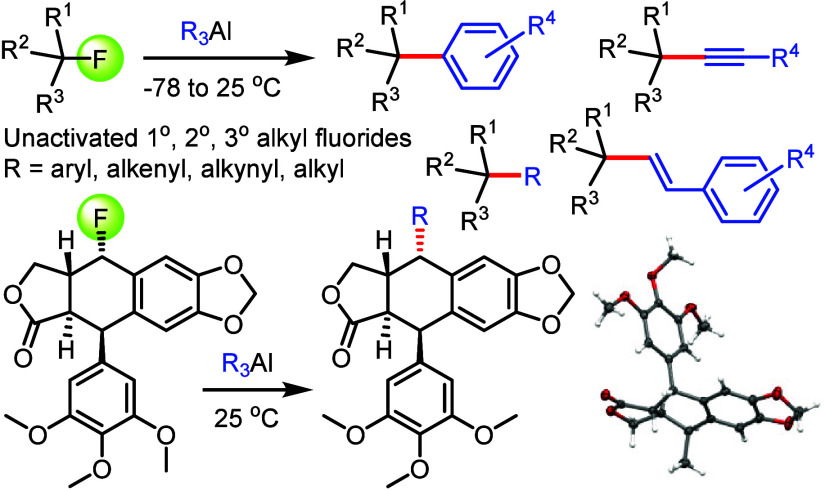

The unique properties of fluorinated organic compounds have received intense interest and have conquered a myriad of applications in the chemical and pharmaceutical sciences. Today, an impressive range of alkyl fluorides are commercially available, and there are many practical methods to make them exist. However, the unmatched stability and inertness of the C–F bond have largely limited its synthetic value, which is very different from the widely accepted utility of alkyl chlorides, bromides, and iodides that serve everyday as “workhorse” building blocks in countless carbon–carbon bond forming reactions. This study demonstrates practical and high-yielding functionalization of the C–F bond under mild conditions, i.e., at temperatures as low as −78 °C, in short reaction times and with unconventional chemoselectivity. Cryogenic Csp^3^–F bond cleavage using fluorophilic organoaluminum compounds together with fast nucleophile transfer of intermediate ate complexes forge carbon–carbon bonds with unactivated primary, secondary, and tertiary alkyl fluorides alike. This method, which exploits the stability of the Al–F bond as the thermodynamic driving force, is highly selective toward Csp^3^–F bond functionalization, whereas many other functional groups including alkyl chloride, bromide, iodide, aryl halide, alkenyl, alkynyl, difluoroalkyl, trifluoromethyl, ether, ester, hydroxyl, acetal, heteroaryl, nitrile, nitro, and amide groups are tolerated, which is an unexpected reversal of long-standing main group organometallic and alkyl halide cross-coupling reactivity and compatibility patterns. As a result, the strongest single bond in organic chemistry can now be selectively targeted in high-yielding arylation, alkylation, alkenylation, and alkynylation reactions and used in late-stage functionalization applications that are complementary to currently available methods.

## Introduction

Aryl–alkyl cross-coupling generates a highly sought-after, ubiquitous motif in organic molecules and is therefore of paramount importance in synthetic chemistry. Alkyl halides are among the most popular starting materials for the synthesis of Csp^2^–Csp^3^ bonds in part because they are easily accessible electrophiles, widely recognized as “workhorse” building blocks, and offer an impressive range of scaffolds from which to choose from. Particular emphasis in synthetic methodology developments has been placed on transition-metal-catalyzed cross-coupling of chlorides, bromides, and iodides with aryl magnesium, boron, zinc, or tin compounds.^1−8^ Persisting shortcomings with alkyl fluorides arise from competing β-hydride elimination, homocoupling, and protodemetalation processes that can prevail and lead to substantial byproduct formation, decreased yields, and continue to impede broader applications including late-stage functionalizations. Although coupling reactions with unactivated alkyl fluorides often exhibit limited substrate scope and low functional group tolerance,^9−16^ an impressive array of successful C–F bond functionalization^17−25^ and defluorinative degradation methods^26,27^ have been established, for example with spring-loaded compounds and benzylic, allylic, or propargylic substrates.^28−33^

Alkyl fluoride functionalization requires cleavage of the strongest single bond that carbon forms.^34^ The bond dissociation energy of a primary Csp^3^–F bond is 485 kJ/mol, which is substantially higher than those of the corresponding alkyl chlorides (339 kJ/mol) and bromides (276 kJ/mol) and twice as strong as in iodides (240 kJ/mol). Although Olah and Kuhn introduced boron Lewis-acid-catalyzed Friedel–Crafts reactions showing selectivity for the C–F bond over other halides 60 years ago, few examples of fluoride-specific C–C bond formations and halodefluorinations, in some cases with substantial formation of isomerization byproducts, are known.^35−37^ As a result, the pronounced increase in thermodynamic stability and decreasing polarizability in the alkyl halide series has cemented an age-old reactivity expectation (C–I > C–Br > C–Cl ≫ C–F) that largely disfavors the use of the Csp^3^–F bond in synthetic chemistry. To date, high-yielding chemoselective synthesis with notoriously inert alkyl fluorides remains very challenging. The introduction of main group organometallics has shown substantial promise in this field, and various transformations with boron-^38−47^ and silicon-derived^48−56^ complexes or frustrated Lewis pairs have appeared. Aluminum chemistry has proven less useful and is mostly confined to alkylations of primary or benzylic fluorides devoid of other functionalities, thus exhibiting a very narrow substrate scope, if any, and limited synthetic utility or applications.^57−59^ Similarly, Csp^2^–Csp^3^ cross-coupling with unactivated alkyl fluorides and arylaluminum reagents has been largely unexplored. In striking contrast to organic synthesis using aryl fluorides and organoaluminum compounds,^60−69^ reactions with alkyl fluorides have thus far been regarded impractical, in particular because of the widely anticipated functional group intolerance and lacking prospect of late-stage functionalization applications. As a result, the utility of alkyl fluorides, which have become increasingly available^70−75^ and of synthetic interest,^76^ continues to be dwarfed by the dominant role of alkyl chlorides, bromides, and iodides that are routinely applied in a plethora of carbon–carbon bond forming reactions.

**Figure 1:**
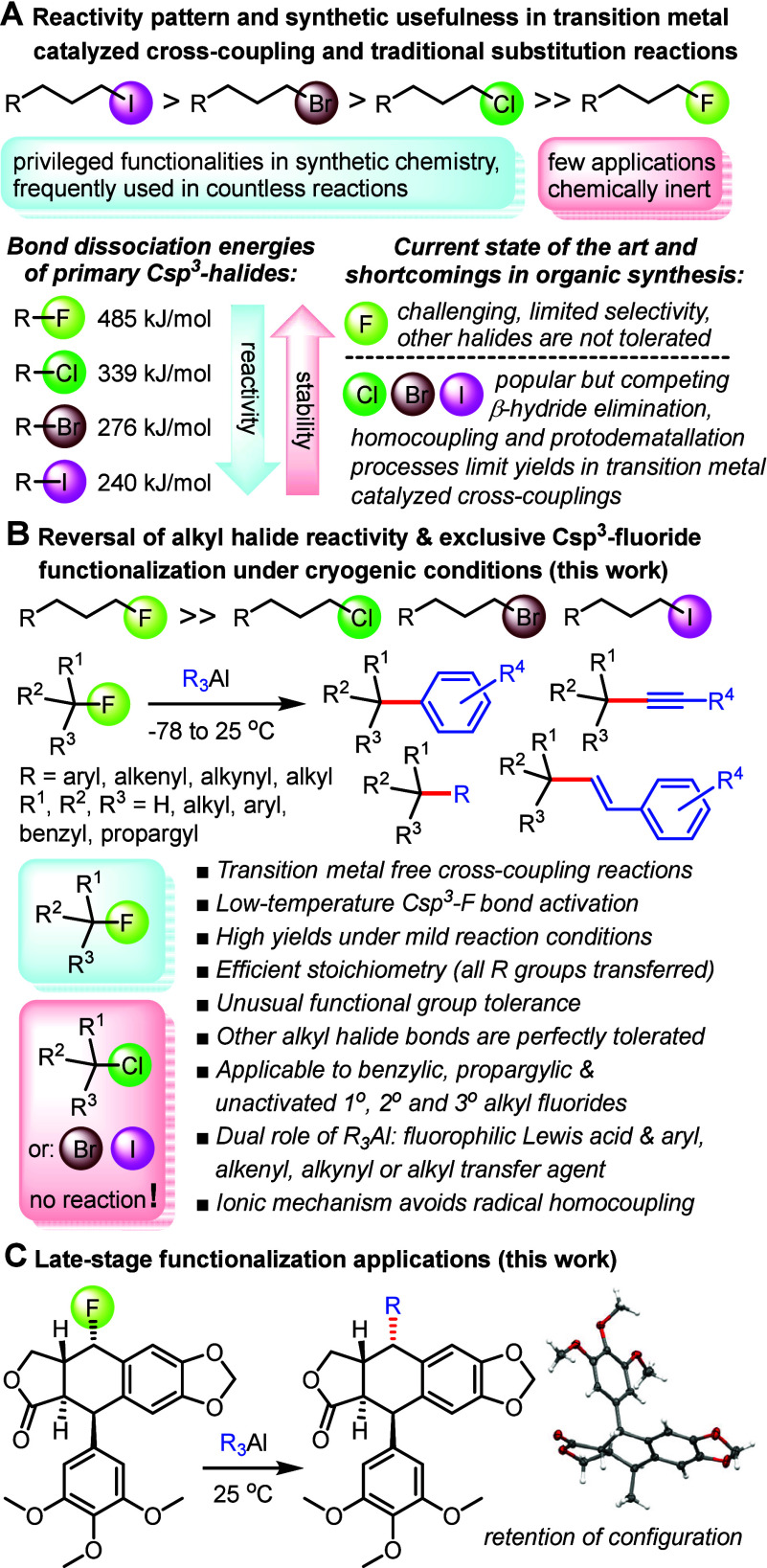
Features of traditional alkyl halide substitution and cross-coupling chemistry (**A**) versus cryogenic alkyl fluoride functionalization with organoaluminum compounds (**B** and **C**).

Herein, we demonstrate broadly applicable cross-coupling using fluorophilic organoaluminum compounds to forge carbon–carbon bonds with unactivated alkyl fluorides (Figure 1). The possibility to efficiently cleave the thermodynamically most stable single bond that carbon can form at temperatures as low as −78 °C sets the stage for high-yielding reactions exhibiting unique chemoselectivity, unprecedented functional group tolerance, and hitherto inconceivable late-stage functionalization applications. The utility of this chemistry spans from Csp^1^–Csp^3^ and Csp^2^–Csp^3^ to Csp^3^–Csp^3^ cross-couplings with primary, secondary, and tertiary alkyl fluorides that typically undergo complete arylation, alkylation, alkenylation, and alkynylation in just 30–120 min at −40 °C or below, although the reactions can also be conveniently conducted at room temperature if desirable.

## Results and Discussion

**Figure 2:**
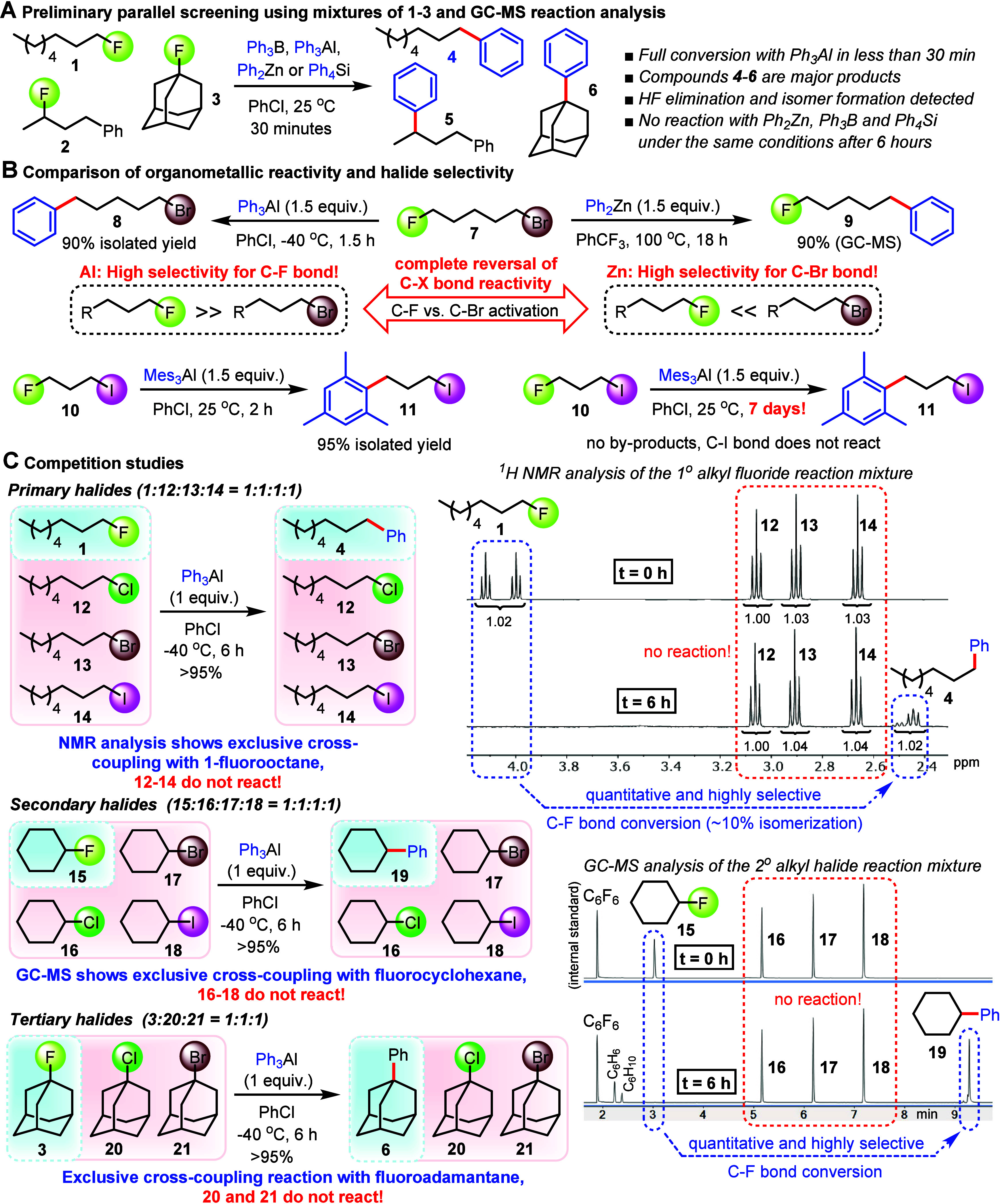
Investigation of cryogenic C–F bond functionalization and chemoselectivity among representative nonactivated alkyl halides. **A.** Preliminary screening of suitable organometallics using alkyl fluorides **1**-**3** in a single pot. **B.** Comparison of carbon–halide bond reactivity. **C.** Competition studies with alkyl fluorides, chlorides, bromides, and iodides.

To gauge the possibility of Csp^2^–Csp^3^ cross-coupling under mild conditions, we selected 1-octyl fluoride, **1**, (3-fluorobutyl)benzene, **2**, and adamantyl fluoride, **3**, representing the entire breadth of challenging nonactivated primary, secondary, and tertiary alkyl fluorides, for parallel screening with triphenylaluminum, triphenylboron, diphenylzinc, and tetraphenylsilane at room temperature. This array of organometallics was chosen because of their extensive fluorophilicity and propensity to form intermediate ate complexes that we postulated would engage in fast aryl transfer and thus achieve Csp^2^–Csp^3^ cross-coupling driven in part by the formation of stable metal fluoride salts. We observed full consumption of **1**–**3** and predominant conversion to the desired coupling products **4**–**6** within 30 min, albeit with noticeable formation of byproducts, using Ph_3_Al under these conditions, while the other reaction mixtures showed only starting materials even after 6 h (Figure 2A). The Csp^2^–Csp^3^ bond formation with Ph_3_Al was surprisingly smooth, which prompted us to test if this reaction could be achieved under cryogenic conditions and possibly enable selective transformation of an unactivated alkyl fluoride bond in the presence of other halides.

We chose 1-bromo-5-fluoropentane, **7**, to investigate the possibility of fluoride-selective Csp^2^–Csp^3^ bond formation at −40 °C (Figure 2B). We found that the fluoride was fully consumed under these conditions, while the alkyl bromide bond was left intact. Moreover, the reaction was complete within just 90 min despite the cryogenic conditions used, and we were able to isolate the desired product **8** in 90% yield with no sign of halogen scrambling. By contrast, highly selective carbon–bromide bond functionalization toward (5-fluoropentyl)benzene, **9**, was observed when **7** was exposed to diphenylzinc at 100 °C. The strikingly chemodivergent transformations of **7** indicated to us that organozinc reagents obey traditional reactivity rules as one would also expect in transition-metal-catalyzed cross-couplings, while organoaluminum chemistry does not and thus provides unexpected synthetic opportunities. We then examined the reaction of trimesitylaluminum with 1-fluoro-3-iodopropane, **10**, at room temperature. The explicit selectivity of triarylaluminum reagents for the Csp^3^–F bond remained unchanged, and we isolated **11** in 95% yield. In fact, we did not observe any sign of reaction with the alkyl iodide moiety even after 7 days at 25 °C in the presence of an excess of the coupling partner.

To illuminate if this chemistry is broadly applicable across unactivated alkyl fluorides, we performed a series of competition experiments. First, a mixture of stoichiometric amounts of the four *n*-octyl halides **1** and **12**–**14** was treated with Ph_3_Al at −40 °C for 6 h (Figure 2C). ^1^H NMR spectroscopic analysis of the crude reaction proved full conversion of the alkyl fluoride **1** to the desired product **4** while the chloride, bromide, and iodide analogs did not react. In addition, we noticed that elimination does not occur under cryogenic conditions and isomerization processes were kept at 10%, which is quite remarkable for primary halides. We then employed the secondary substrates **15**–**18** in the same experimental setup. Similarly, exclusive Csp^2^–Csp^3^ bond formation with fluorocyclohexane, which also gave small amounts of cyclohexene, was observed. Comparison with an internal standard revealed that the amounts of the thermodynamically less stable alkyl chloride, bromide, and iodide compounds were unaffected, showing no sign of C–C bond formation or elimination reactions. Lastly, the tertiary halides **3**, **20**, and **21** were tested under identical conditions, and we were able to confirm that the Csp^3^–F bond functionalization is both selective and quantitative as **6** was solely formed from adamantyl fluoride, while **20** and **21** remained unaltered in the reaction mixture (see Supporting Information). Since activation of all carbon–halide bonds should be thermodynamically possible, we investigated the reaction of Ph_3_Al with 1-chlorooctane, 1-bromooctane, 1-iodooctane, chlorocyclohexane, bromocyclohexane, iodocyclohexane, 1-chloroadamantane, and 1-bromoadamantane using chlorobenzene as the solvent at higher temperatures. We found that among the primary halides, 1-chlorooctane was most reactive. The reaction was slow at 50 °C, but conversion to 1-phenyloctane was observed. However, side reactions toward Friedel–Crafts products derived from the chlorobenzene solvent were favored at 100 °C. This was also the case with the primary bromide analog. By contrast, the coupling with Ph_3_Al was favored when 1-iodooctane was used, although large amounts of isomers were produced. The secondary and tertiary halides reacted quantitatively at 50 °C but mostly via Friedel–Crafts reaction with the chlorobenzene solvent. The adamantyl halides also showed substantial amounts of dehalogenation toward adamantane (see SI). These results show that without exception cross-coupling between Ph_3_Al and alkyl fluorides occurs much more smoothly and with substantially better reaction control. The carbon–halide bond activation trends and the significantly increased reactivity of the C–F bond compared to the other alkyl halides are in agreement with Olah’s analysis of Lewis-acid-catalyzed Friedel–Crafts reactions.^35^

**Figure 3:**
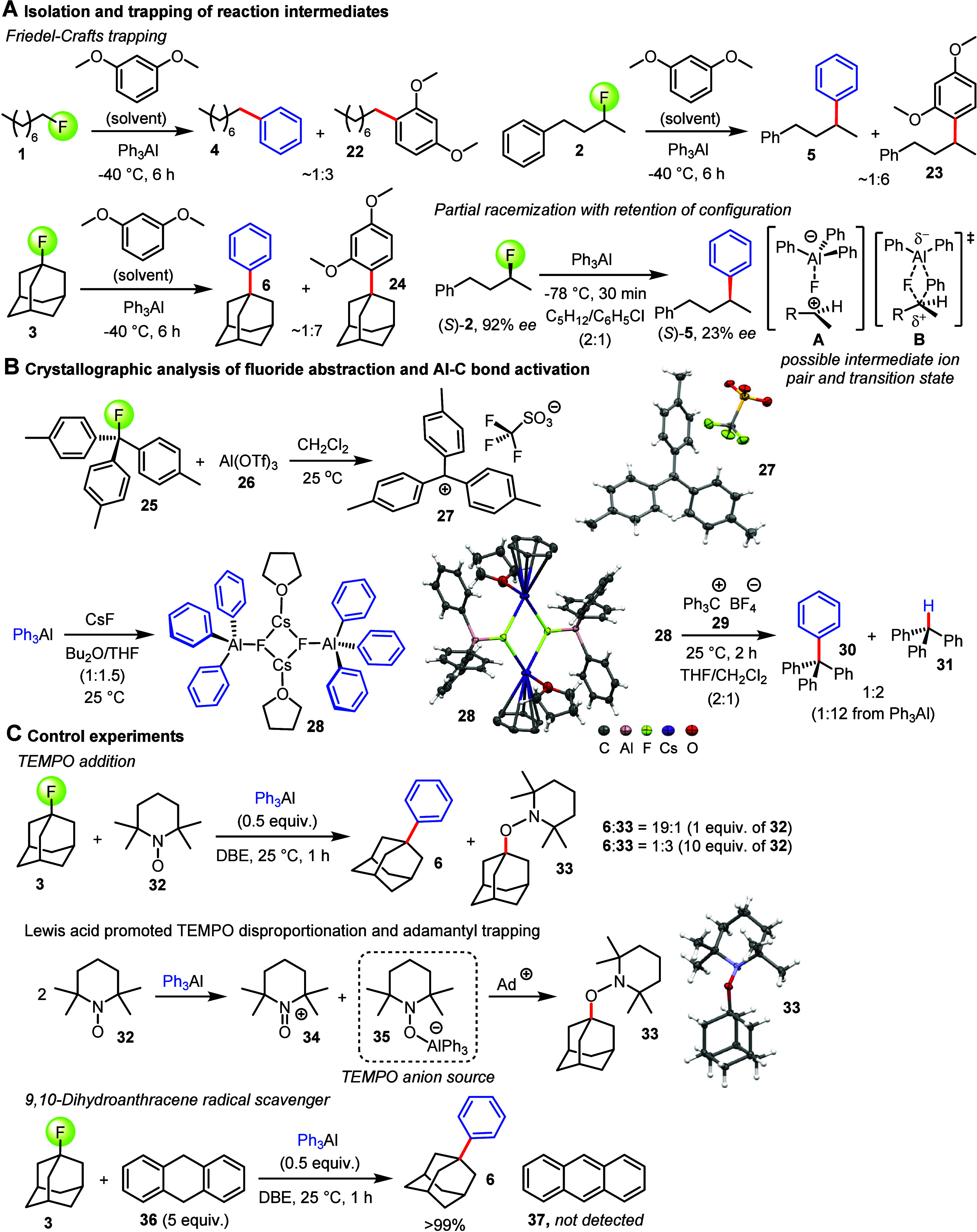
Mechanistic Investigation of the organoaluminum cross-coupling reactions with alkyl fluorides. **A.** Friedel–Crafts and racemization study. The absolute configuration of (*S*)-**5** was assigned by comparison with that in the literature. The *ee* values of **2** and **5** were determined by chiral HPLC on CHIRALCEL OJ-H and GC on 2,6-dimethyl-3-pentyl-γ-cyclodextrin, respectively. **B.** Crystallographic reaction intermediate analysis. The Al–F bond in activated aluminate **28** was determined as 1.739(3) Å. The Al–C bond lengths are 1.991(5) Å, 1.994(5) Å, and 2.001(5) Å. For comparison: The Al–C bond lengths in [(THF)AlPh_3_] range from 1.976 to 1.982 Å. **C.** Reaction analysis with TEMPO and 9,10-dihydroanthracene radical probes.

These findings motivated additional experiments to investigate the mechanistic underpinnings of our aryl-alkyl cross-coupling method. Lewis-acid-mediated halodefluorination reactions of alkyl fluorides have been reported and found to typically proceed via an ionic mechanism, i.e., through an intermediate ion pair, although concerted reactions have also been discussed.^17,36,37,40^ We first conducted carbocation trapping experiments with the representative alkyl fluorides **1**–**3** under identical conditions using 1,3-dimethoxybenzene as a solvent. In all three cases, **4**–**6** were obtained in relatively small amounts compared to the predominant Friedel–Crafts adducts **22**–**24** (Figure 3A). As the stability of the intermediate carbocations that are presumably formed via heterolytic cryogenic C–F bond cleavage increases, a steady change in the ratio of the cross-coupling product to the Friedel–Crafts compound from 1:3 to 1:7 was observed. The Friedel–Crafts reaction was found to overwhelmingly outcompete the cross-coupling pathway when a benzylic fluoride was tested under the same conditions (see SI). Careful GC-MS analysis of the cross-coupling and Friedel–Crafts reactions with unactivated primary and secondary alkyl fluorides revealed several isomerization byproducts showing C–C bond formation at the β-, γ-, and even δ-positions due to consecutive hydride shifts of rapidly interconverting intermediate carbocations (SI). Competition experiments with Ph_3_Al and equimolar amounts of 1-fluorooctane, fluorocyclohexane and 1-fluoroadamantane showed a reactivity trend (tertiary > secondary > primary alkyl fluoride) that is in agreement with a stepwise mechanism proceeding through a carbocation aluminate intermediate formed in the rate-determining step (SI). Accordingly, the Lewis acidity of the aluminum species is important, which was further examined by comparison of aluminum trichloride, tribromide, and triiodide. We observed that the reactivity of the primary alkyl fluoride **1** in the corresponding halodefluorinations indeed increases with the Al Lewis acidity (AlI_3_ > AlBr_3_ > AlCl_3_). The negligible amount of isomerization observed indicates relatively short lifetimes of intermediate ion pairs but would also be consistent with a concerted mechanism (*vide infra*). The secondary alkyl fluoride **2** proved more reactive than **1** under the same conditions, pointing toward a predominant ionic mechanism, as larger amounts of remaining starting materials would have been expected for a concerted pathway with the sterically more hindered substrate. Isomerization byproducts were detected in decreasing amounts as AlCl_3_ was replaced with AlBr_3_ and AlI_3_. This trend follows the increasing nucleophilicity of the halide (iodide > bromide > chloride) as one would expect for a stepwise mechanism in which the lifetime of the intermediate ion pairs decreases accordingly, i.e., the iodide is expected to be transferred significantly faster than the other halides. This leaves less time for the competing isomerization of the intermediate carbocation, as was observed (see SI for details). Further evidence for a stepwise ionic mechanism was obtained with (*S*)-(3-fluorobutyl)benzene, **2**, which was prepared in 92% *ee* in one step from (*R*)-4-phenylbutan-2-ol according to a literature procedure.^77^ The reaction with triphenylaluminum at −78 °C gave (*S*)-**5** in 23% *ee* suggesting that the mechanism may involve a short-lived carbocation-aluminate ion pair **A** that undergoes relatively fast aryl transfer (Figure 3A). Despite the considerable racemization, the prevailing retention of configuration indicates that the C–C bond formation occurs on a similar time scale with dissociation and tumbling processes, which are assumed to allow reorientation between the intermediate prochiral carbocation and the aluminate nucleophile. Alternatively, the retention of configuration could be explained with a contribution from a concerted mechanism via transition state **B**. We then sought additional insights into the mechanism and structure of a nucleophilic aluminate activated by fluoride coordination. We envisioned aluminum triflate to be sufficiently Lewis acidic to achieve fluoride abstraction with tertiary alkyl fluorides, but unlike triarylaluminates, would not react any further and form an isolable ion pair instead. Treatment of the trityl fluoride **25** with **26** indeed gave the ion pair **27**, and we were able to grow single crystals suitable for X-ray analysis, which indicates that **25** would react via alkyl cation formation (Figure 3B). We were very pleased to find that cesium fluoride and Ph_3_Al afford polynuclear assembly **28**, a unique snapshot of a plausible aryl transfer agent that would be formed by fluoride abstraction. Careful crystallographic inspection of this aggregate exhibiting a rare example of an organometallic main group complex with tricoordinate fluoride bridges revealed short Al–F bonds and slightly elongated Al–Csp^2^ separation compared to [(THF)AlPh_3_].^78^ These distinct structural properties corroborate the high fluorophilicity of Al and the enhanced nucleophilicity of the triarylaluminum moiety in **28** upon fluoride binding, important features that are in accordance with our initial mechanistic hypothesis. The ability of **28** to engage in the transfer of a phenyl group became evident when it was treated with trityllium tetrafluoroborate, **29**, to form the desired product **30**, albeit accompanied by significant hydrodefluorination toward **31**. By contrast, unactivated triphenylaluminum proved to be a very poor arylating agent under the same conditions. We then discovered that the cross-coupling reaction with **3** prevails in the presence of TEMPO, **32**, although we isolated the unexpected byproduct **33**, in particular when **32** was used in large excess (Figure 3C). A literature survey uncovered that the formation of **33** is unlikely due to a radical mechanism but a result of TEMPO disproportionation known to occur in the presence of an aluminum Lewis acid, thus producing **34** and the aminoxyl anion complex **35**.^79^ The reaction between **35** and the adamantyl cation would then afford **33**. To further rule out contributions from a homolytic C–F bond cleavage pathway, we used an excess of the well-known radical scavenger 9,10-dihydroanthracene, **36**. In this case, **6** was produced in quantitative amounts and no sign of the formation of **37**, which would have been generated via reaction with intermediate radical species, was observed.

The initial screening efforts discussed above revealed that the Csp^2^–Csp^3^ bond formation coincides with elimination and isomerization processes competing with the desired cross-coupling outcome. We therefore conducted an extensive analysis of side reactions and possible interferences, thereby optimizing solvent, temperature, and other parameters (see SI). The reaction with primary fluoride generally proceeds smoothly at −40 °C, whereas unactivated secondary fluorides proved prone to HF elimination, which we found can be effectively reduced at −78 °C. Moreover, we found that the reaction still goes to completion when only a third equivalent of Ph_3_Al is employed; albeit, longer reaction times are needed. We realized that the use of 1.5 equiv of the cross-coupling reagent at −40 °C is generally a good compromise to achieve fast alkyl fluoride conversion while maintaining wide functional group tolerance under these conditions (*vide infra*).

**Figure 4:**
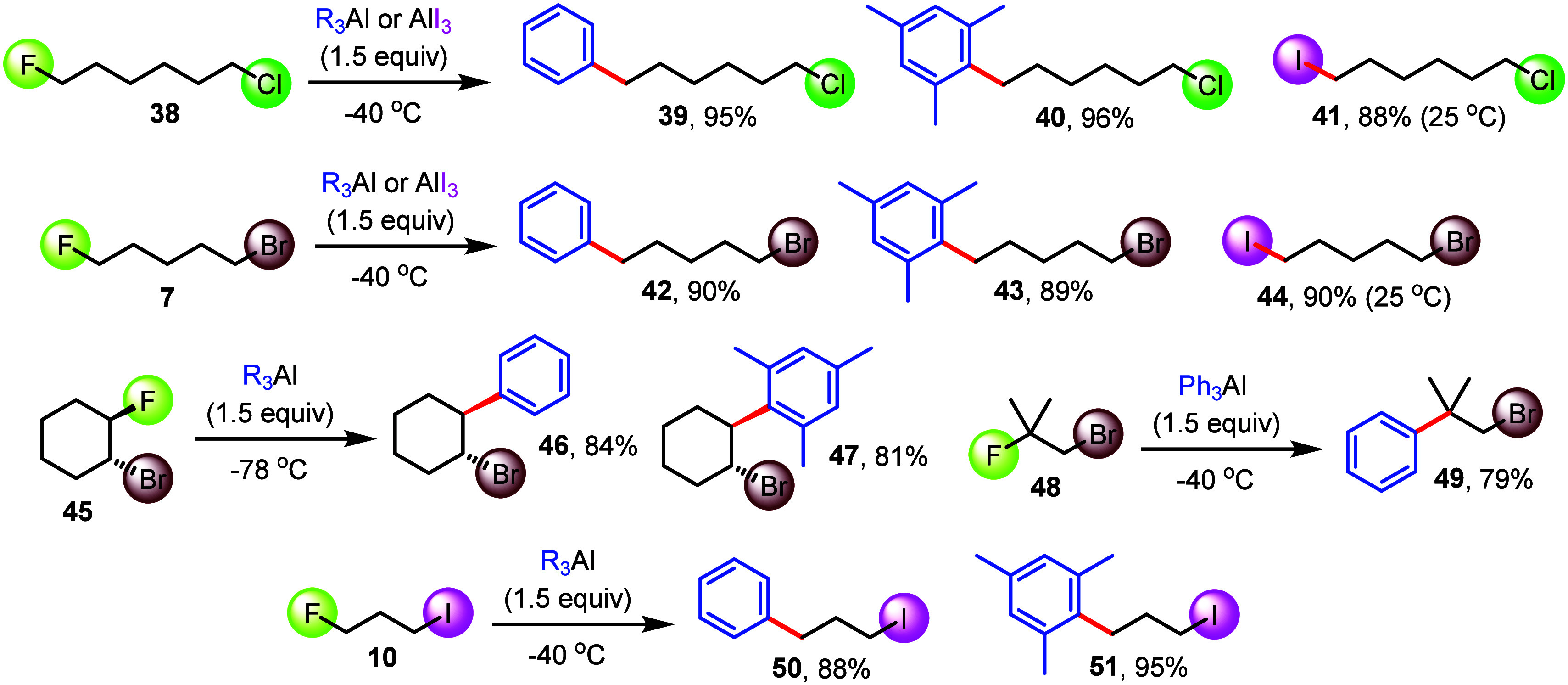
Highly chemoselective transformations with dihalides show favorable C–F bond reactivity.

**Figure 5:**
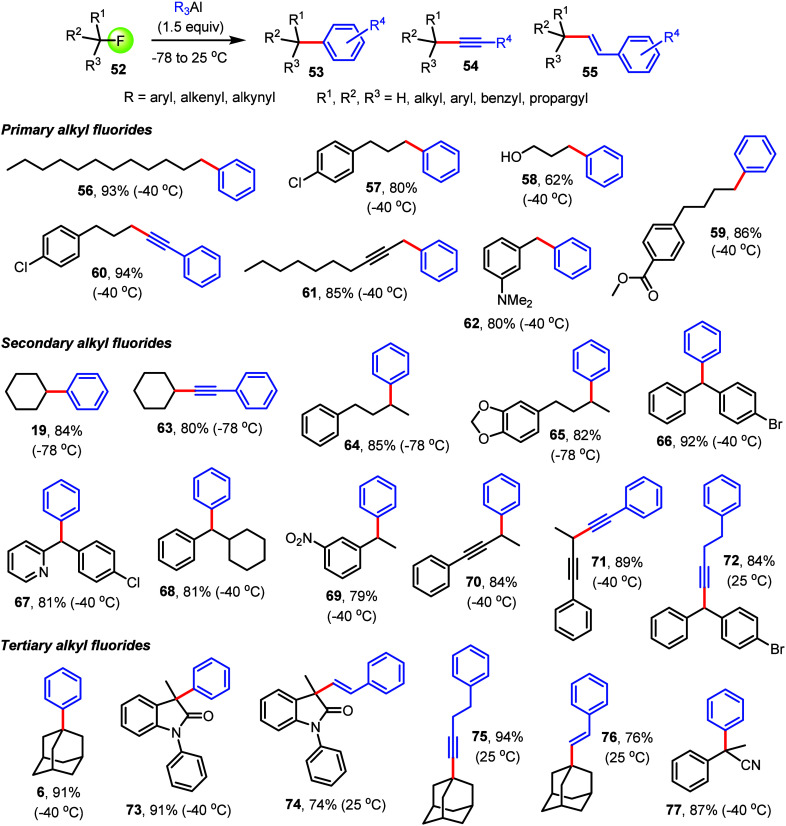
C–F bond reactivity and functionalization of unactivated, benzylic, or propargylic alkyl fluorides using aryl, alkenyl, and alkynyl aluminum complexes. All yields are based on isolated products.

With a set of optimized conditions in hand, we continued to explore the scope of alkyl fluoride cross-coupling with organoaluminum reagents and realized that this chemistry is broadly applicable (Figures 4 and 5). Low-temperature arylation, alkenylation, alkynylation, and modulation of the historic Finkelstein reaction^80^ with unique selectivity for replacing fluoride in the presence of another halide are possible regardless of the steric hindrance at the Csp^3^–F bond. Indeed, we found that primary, secondary, and tertiary substrates are equally viable candidates, while many functional groups in addition to chlorides, bromides, and iodides are tolerated. In the beginning, we screened a series of readily available aliphatic dihalides (Figure 4). The cross-coupling of 1-chloro-6-fluorohexane, **38**, at −40 °C gave **39** and **40** in excellent yield, leaving the carbon–chloride bond perfectly intact. Essentially the same impeccable selectivity toward Csp^3^–F bond functionalization was observed when **38** was treated with AlI_3_ which gave 1-chloro-6-iodohexane, **41**, in 88% yield. The presence of a more reactive alkyl bromide moiety does not alter the inherent chemoselectivity of this chemistry, and we isolated **42**–**44** from the primary fluoride **7** in 89 and 90% yield, respectively. Moreover, secondary and tertiary substrates can be used as well. This is demonstrated by the cross-coupling reactions of *trans*-1-bromo-2-fluorocyclohexane, **45**, producing **46** and **47** with retention of configuration, and the synthesis of **49** from 1-bromo-2-fluoro-2-methylpropane, **48**. Finally, the arylated products **50** and **51** were prepared from 1-fluoro-3-iodopropane and isolated in 88 and 95% yield.

Having demonstrated outstanding selectivity toward Csp^3^–F bond functionalization with dihalogenated compounds, which cannot be achieved with regular transition-metal-catalyzed cross-coupling methods, we turned our attention to other alkyl fluorides. First, several primary alkyl fluorides were successfully subjected to Csp^2^–Csp^3^ bond formation using our standard protocol at −40 °C. This gave direct access to **56**–**59** and we found that unprotected 3-fluoropropanol can be used as well, albeit with compromised yields. It is noteworthy that the cross-coupling is not restricted to alkyl–aryl bond formation, and alkyne moieties can be transferred with similar efficiency. We were thus able to isolate **60** in 94% yield without additional optimization for this Sonogashira-type reaction. Smooth C–C bond formation occurs in propargylic and benzylic positions, and **61** and **62** were obtained in 80–85% yield. Next, secondary alkyl fluorides were investigated. During method optimization, we learned that these are more challenging than primary and tertiary fluorides because of increasingly competitive HF elimination and rearrangement pathways, resulting in the formation of approximately 10% of alkene and 40% of isomeric byproducts when the reaction was carried out at −40 °C. These side reactions were largely reduced at −78 °C, and compounds **19** and **63**–**65** were thus produced via arylation and alkynylation in 80–85% yield. Another benefit of using fluorinated compounds in cross-coupling chemistry became apparent when we used doubly benzylic fluorides. While the corresponding chlorides, bromides, and iodides are increasingly unstable and known to undergo uncontrolled radical reactions including homocoupling toward tetraarylethanes and other byproducts, we obtained **66** and **67** with 81–92% yield without a sign of dimerization. Probably one of the most remarkable advantages of switching from standard cross-coupling conditions to our method is the ease of high-yielding arylation with α-fluorinated arylalkanes. To date, Csp^2^–Csp^3^ bond formation with secondary fluorides that predominantly forms styrenes via elimination has been a serious challenge, and unsatisfactory results have been reported.^15^ By contrast, we isolated **68** and **69** via defluorinative C–C coupling in 79–81% yield. The synthesis of **70**–**72** demonstrates the versatility of organoaluminum Csp^3^–F bond functionalization, as alkynes can be formed either by arylation of propargylic fluorides or via Sonogashira coupling chemistry. Finally, tertiary alkyl fluorides were tested, and we were pleased to observe successful arylation, alkenylation, and alkynylation toward **6** and **73**–**76**. The high-yielding conversion of 2-fluoro-2-phenylpropanenitrile to **77** rather than to strongly resonance-stabilized α-phenylacrylonitrile is another example demonstrating how difficulties commonly encountered during reactions with alkyl fluorides that predominantly form elimination products are effectively mitigated by our method. In addition to the advantageous features of the organoaluminum Csp^3^–F bond functionalization chemistry discussed above, the reactions presented in Figure 5 illustrate exceptional functional group compatibility. In fact, alkyl chloride, bromide, iodide, and aryl halide bonds or alkenyl, alkynyl, difluoalkyl, trifluoromethyl, ether, ester, hydroxyl, acetal, heteroaryl, nitrile, nitro, and amide groups are all tolerated.

**Figure 6:**
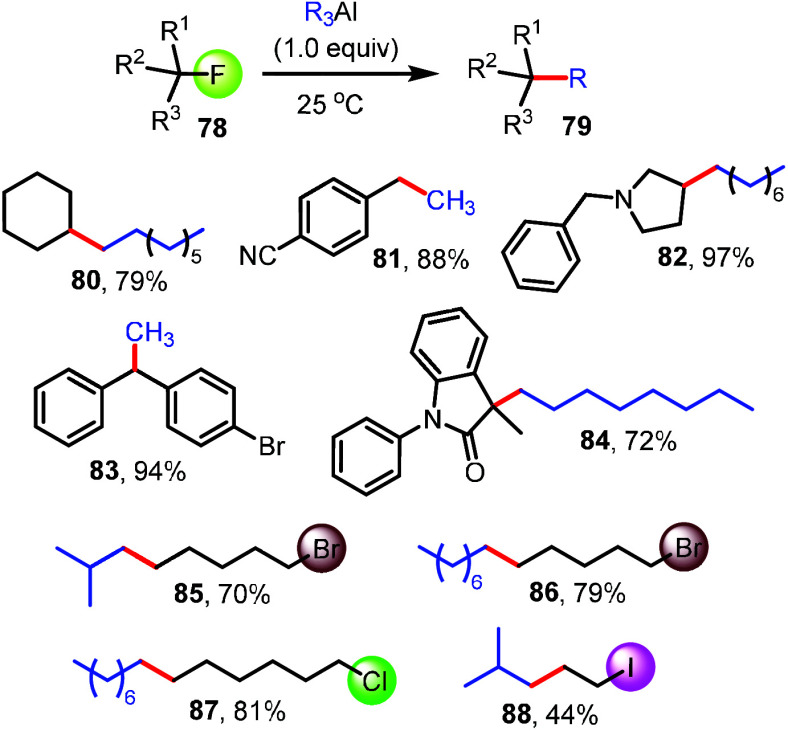
C(sp^3^)–C(sp^3^) bond formation with 1°, 2°, and 3° alkyl fluorides or dihalides using saturated organoaluminum compounds.

Encouraged by the scope and chemoselectivity of C(sp^3^)–F bond functionalization with aryl-, alkenyl-, and alkynylaluminum compounds, we decided to test the possibility of cross-coupling reactions with saturated analogs. As shown in Figure 6, we observed that C(sp^3^)–C(sp^3^) bond formation occurs smoothly with activated and unactivated alkyl fluorides, producing **80**–**84** in good to high yields. Competition with other alkyl halide moieties present in the same molecule continued to show stark preference for the C–F bond, and we isolated the corresponding alkyl bromides and chlorides **85**–**87** in 70–81% yield. As with every new synthetic methodology, there are remaining boundaries. Accordingly, we discovered that the defluorinative transfer of an isobutyl group to 1-fluoro-2-iodoethane, a challenging substrate prone to dihalide elimination, gave **88** in only 44% yield. Careful product analysis revealed that the compromised yield can be at least partially attributed to competing I/Al-exchange, a generally known pathway with aliphatic main group organometallics.^81^

**Figure 7:**
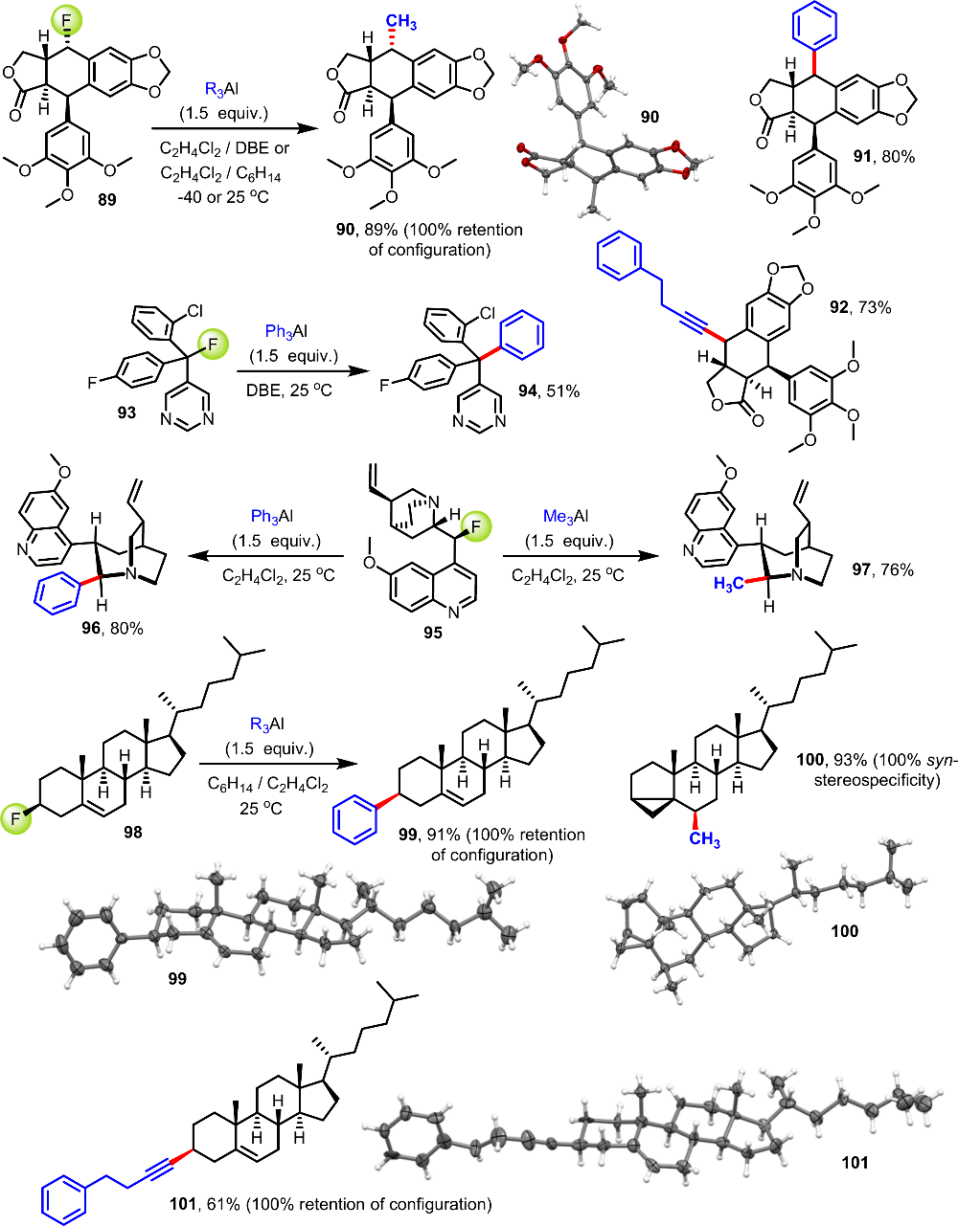
Selective late-stage C(sp^3^)–F bond functionalization with organoaluminum reagents. All yields are reported for isolated products.

Strategies that allow modification or diversification of highly decorated structures and agrochemical or pharmaceutical leads based on a single late-stage transformation have become attractive means to explore new chemical space and compound development opportunities without the cumbersome necessity for entirely new synthesis. This is possible with high functional group tolerance by using the C(sp^3^)–F bond as a chemical handle that is typically inert during traditional organic synthesis but awaits selective functionalization with organoaluminum compounds (Figure 7). Having performed a variety of high-yielding C(sp^3^)–F bond functionalizations under mild conditions, we first assessed the utility of this chemistry with fluoropodophyllotoxin, **89**. Podophyllotoxins are complex aryltetralin lactone lignans that have raised common interest due to their potent antitumor activities and use in the treatment of HPV infections. We were pleased to find that the reaction with trimethylaluminum results in stereoretentive defluorinative alkylation of **89** toward **90**, which was obtained as a single stereoisomer in 89% yield. The lactone, acetal, and ether groups as well as the configurations at all four chirality centers were left unaltered, and the characteristic *trans*-geometry between the trimethoxyphenyl ring and the introduced methyl group was unequivocally verified by crystallographic analysis. The corresponding phenylation and alkynylation reactions produced **91** and **92** in 73–80% yield. We then found that the fluoride derivative of fungicidal nuarimol **93** undergoes selective phenylation to the crowded tetraarylmethane **94**, leaving both the aryl halide bonds and the pyrimidyl ring intact. The profusely diverse biological activities, medicinal applications, usefulness in chemical synthesis and organocatalysis, and rich nucleophilic displacement and rearrangement chemistry of cinchona alkaloids render reactions with structures like **95** particularly appealing. We observed that this multifunctional fluoride undergoes stereoselective cage expansion to the 1-azabicyclo[3.2.2]nonane scaffolds **96** and **97** in high yields, which compares well with other C-9 and quinuclidine derivatization methods,^82^ providing new synthetic opportunities that cannot be realized with previously reported organometallic dehalogenative C–C bond formations.^83^ We finally investigated late-stage functionalization and diversity-oriented synthesis with 3β-fluorocholestane, **98**, and observed smooth conversion to 3β-phenylcholesterol, **99**, using triphenylaluminum at room temperature. This reaction shows no sign of diastereomerization and exclusively gives the 3*S* stereoisomer in a 91% yield. By contrast, the reaction with trimethylaluminum is strikingly different and outcompetes the known homoallylic rearrangement with cholesteryl tosylate and organocuprates,^84^ generating the cyclopropanation product **100** in 93% yield and with excellent *syn*-stereospecificity. In accordance with the formation of **99**, we found that defluorinative alkynylation results in highly stereoretentive C–C coupling toward **101**. Importantly, the individual stereochemistry and architectures of cholesterol derivatives **99**–**101** were unequivocally confirmed by single crystal X-ray analysis.

## Conclusions

The methodological advances discussed herein demonstrate that the Csp^3^–F bond, widely deemed chemically inert even under harsh conditions, can be smoothly functionalized with high chemoselectivity and large functional group tolerance at cryogenic temperatures as low as −78 °C. This is achieved through heterolytic C–F bond cleavage with highly fluorophilic organoaluminum compounds and subsequent nucleophile transfer within intermediate carbocation-aluminate ion pairs that are sufficiently short-lived to enable high-yielding C–C bond formation while outperforming a variety of conceivable side reactions, including β-hydride elimination, homodimerization, hydrodefluorination, and protodemetalation. It is now possible to apply nonactivated primary, secondary, and tertiary alkyl fluorides or benzylic and propargylic ones in transition-metal-free carbon–carbon bond cross-couplings while leaving thermodynamically less stable alkyl chloride, bromide, and iodide bonds as well as many other frequently encountered functional groups such as aryl halides, alkenyl, alkynyl, difluoroalkyl, trifluoromethyl, ether, ester, hydroxyl, acetal, heteroaryl, nitrile, nitro, and amide groups intact. The cryogenic fluoride abstraction and fast C-nucleophile transfer sequence discussed herein thus reverse commonly perceived functional group incompatibility of organoaluminum chemistry and traditional alkyl halide reactivity patterns. The broad utility and distinct chemoselectivity are supported by many examples and probably most strikingly exemplified by the opposing reactivity and chemodivergent reaction outcome observed with organoaluminum and organozinc compounds. As a result, the strongest single bond in organic chemistry can now be selectively functionalized under mild conditions, with broad utility and unusual synthetic applications that are orthogonal to current methodologies. The wide scope and variety of selective alkyl fluoride cross-couplings with saturated and unsaturated organoaluminum reagents and late-stage functionalizations exhibiting unprecedented stereoselectivities significantly expand the synthetic utility of the ubiquitous Csp^3^–F bond and set the stage for new chemical synthesis opportunities and retrosynthetic planning. The findings of this study are expected to advance the routine use of C–F bond functionalization strategies aimed at exploring challenging synthetic space and compound development venues that have not been accessible to date.

## Supporting Information Available

Experimental section: detailed synthetic procedures, mechanistic studies, compound characterization (PDF)

## References

[ref1] Zhou J.; Fu G. C. Palladium-Catalyzed Negishi Cross-Coupling Reactions of Unactivated Alkyl Iodides, Bromides, Chlorides, and Tosylates. J. Am. Chem. Soc. 2003, 125, 12527–12530. 10.1021/ja0363258. 14531697

[ref2] Zhou J.; Fu G. C. Suzuki Cross-Couplings of Unactivated Secondary Alkyl Bromides and Iodides. J. Am. Chem. Soc. 2004, 126, 1340–1341. 10.1021/ja039889k. 14759182

[ref3] Powell D. A.; Maki T.; Fu G. C. Stille Cross-Couplings of Unactivated Secondary Alkyl Halides Using Monoorganotin Reagents. J. Am. Chem. Soc. 2005, 127, 510–511. 10.1021/ja0436300. 15643860

[ref4] Yang C.-T.; Zhang Z.-Q.; Liu Y.-C.; Liu L. Copper-Catalyzed Cross-Coupling Reaction of Organoboron Compounds with Primary Alkyl Halides and Pseudohalides. Angew. Chem., Int. Ed. 2011, 50, 3904–3907. 10.1002/anie.201008007. 21455914

[ref5] Hatakeyama T.; Hashimoto T.; Kondo Y.; Fujiwara Y.; Seike H.; Takaya H.; Tamada Y.; Ono T.; Nakamura M. Iron-Catalyzed Suzuki–Miyaura Coupling of Alkyl Halides. J. Am. Chem. Soc. 2010, 132, 10674–10676. 10.1021/ja103973a. 20681696

[ref6] Liang Y.; Fu G. C. Catalytic Asymmetric Synthesis of Tertiary Alkyl Fluorides: Negishi Cross-couplings of Racemic α,α-Dihaloketones. J. Am. Chem. Soc. 2014, 136, 5520–5524. 10.1021/ja501815p. 24678878 PMC4004247

[ref7] Yin H.; Fu G. C. Mechanistic Investigation of Enantioconvergent Kumada Reactions of Racemic α-Bromoketones Catalyzed by a Nickel/Bis(oxazoline) Complex. J. Am. Chem. Soc. 2019, 141, 15433–15440. 10.1021/jacs.9b08185. 31502449 PMC7075318

[ref8] Li Y.; Luo Y.; Peng L.; Li Y.; Zhao B.; Wang W.; Pang H.; Deng Y.; Bai R.; Lan Y. G. Yin Reaction Scope and Mechanistic Insights of Nickel-catalyzed Migratory Suzuki–Miyaura Cross-coupling. Nature Comm. 2020, 11, 417 10.1038/s41467-019-14016-1. PMC6972863 31964876

[ref9] Terao J.; Ikumi A.; Kuniyasu H.; Kambe N. Ni- or Cu-Catalyzed Cross-Coupling Reaction of Alkyl Fluorides with Grignard Reagents. J. Am. Chem. Soc. 2003, 125, 5646–5647. 10.1021/ja034201p. 12733899

[ref10] Blessley G.; Holden P.; Walker M.; Brown J. M.; Gouverneur V. Palladium-Catalyzed Substitution and Cross-Coupling of Benzylic Fluorides. Org. Lett. 2012, 14, 2754–2757. 10.1021/ol300977f. 22594918

[ref11] Mo Z.; Zhang Q.; Deng L. Dinuclear Iron Complex-Catalyzed Cross-Coupling of Primary Alkyl Fluorides with Aryl Grignard Reagents. Organometallics 2012, 31, 6518–6521. 10.1021/om300722g.

[ref12] Iwasaki T.; Min X.; Fukuoka A.; Kuniyasu H.; Kambe N. Nickel-Catalyzed Dimerization and Alkylarylation of 1,3-Dienes with Alkyl Fluorides and Aryl Grignard Reagents. Angew. Chem., Int. Ed. 2016, 55, 5550–5554. 10.1002/anie.201601126. 26938137

[ref13] Iwasaki T.; Fukuoka A.; Yokoyama W.; Min X.; Hisaki I.; Yang T.; Ehara M.; Kuniyasu H.; Kambe N. Nickel-catalyzed Coupling Reaction of Alkyl Halides with Aryl Grignard Reagents in the Presence of 1,3-Butadiene: Mechanistic Studies of Four-Component Coupling and Competing Cross-coupling Reactions. Chem. Sci. 2018, 9, 2195–2211. 10.1039/C7SC04675H. 29719693 PMC5903371

[ref14] Wolfe M. M. W.; Shanahan J. P.; Kampf J. W.; Szymczak N. K. Defluorinative Functionalization of Pd(II) Fluoroalkyl Complexes. J. Am. Chem. Soc. 2020, 142, 18698–18705. 10.1021/jacs.0c09505. 33073563

[ref15] Balaraman K.; Wolf C. Palladium and Nickel Catalyzed Suzuki Cross-Coupling with Alkyl Fluorides. Org. Lett. 2021, 23, 8994–8999. 10.1021/acs.orglett.1c03515. 34723542 PMC8987895

[ref16] Figula B. C.; Kane D. L.; Balaraman K.; Wolf C. Organocuprate Cross–Coupling Reactions with Alkyl Fluorides. Org. Lett. 2022, 24, 8719–8723. 10.1021/acs.orglett.2c03775. 36394939 PMC10502612

[ref17] Traff A. M.; Janjetovic M.; Ta L.; Hilmersson G. G. Selective C-F Bond Activation: Substitution of Unactivated Alkyl Fluorides Using YbI_3_. Angew. Chem., Int. Ed. 2013, 52, 12073–12076. 10.1002/anie.201306104. 24115642

[ref18] Shen Q.; Huang Y.-G.; Liu C.; Xiao J.-C.; Chen Q.-Y.; Guo Y. Review of Recent Advances in C–F Bond Activation of Aliphatic Fluorides. J. Fluor. Chem. 2015, 179, 14–22. 10.1016/j.jfluchem.2015.07.007.

[ref19] Balaraman K.; Wolf C. Catalytic Enantioselective and Diastereoselective Allylic Alkylation with Fluoroenolates: Efficient Access to C3-Fluorinated and All-Carbon Quaternary Oxindoles. Angew. Chem., Int. Ed. 2017, 56, 1390–1395. 10.1002/anie.201608752. PMC5289271 28026079

[ref20] Zhang X.; Cao S. Recent Advances in the Synthesis and C-F Functionalization of *gem*-Difluoroalkenes. Tetrahedron Lett. 2017, 58, 375–392. 10.1016/j.tetlet.2016.12.054.

[ref21] Ge D.; Chu X.-Q. Multiple-fold C–F Bond Functionalization for the Synthesis of (Hetero)cyclic Compounds: Fluorine as a Detachable Chemical Handle. Org. Chem. Front. 2022, 9, 2013–2055. 10.1039/D1QO01749G.

[ref22] Yan G.; Qiu K.; Guo M. Recent Advance in the C–F bond Functionalization of Trifluoromethyl-Containing Compounds. Org. Chem. Front. 2021, 8, 3915–3942. 10.1039/D1QO00037C.

[ref23] Balaraman K.; Wolf C. Chemodivergent Csp^3^-F Bond Functionalization and Cross-Electrophile Alkyl-Alkyl Coupling with Alkyl Fluorides. Science Adv. 2022, 8, eabn7819 10.1126/sciadv.abn7819. PMC9140971 35622926

[ref24] Kane D. L.; Figula B. C.; Balaraman K.; Bertke J. A.; Wolf C. General Alkyl Fluoride Functionalization via Short-lived Carbocation-Organozincate Ion Pairs. Nat. Commun. 2024, 15, 1866 10.1038/s41467-024-45756-4. 38424080 PMC10904780

[ref25] Lye K.; Young R. D. A Review of Frustrated Lewis Pair Enabled Monoselective C–F Bond Activation. Chem. Sci. 2024, 15, 2712–2724. 10.1039/D3SC06485A. 38404400 PMC10882520

[ref26] Trang B.; Li Y.; Xue X.; Ateia M.; Houk K. N.; Dichtel W. R. Low-temperature Mineralization of Perfluorocarboxylic Acids. Science 2022, 377, 839–845. 10.1126/science.abm8868. 35981038

[ref27] Sheldon D. J.; Parr J. M.; Crimmin M. R. Room Temperature Defluorination of Poly(tetrafluoroethylene) by a Magnesium Reagent. J. Am. Chem. Soc. 2023, 145, 10486–10490. 10.1021/jacs.3c02526. 37154713 PMC10197119

[ref28] Erickson L. W.; Lucas E. L.; Tollefson E. J.; Jarvo E. R. Nickel-Catalyzed Cross-Electrophile Coupling of Alkyl Fluorides: Stereospecific Synthesis of Vinylcyclopropanes. J. Am. Chem. Soc. 2016, 138, 14006–14011. 10.1021/jacs.6b07567. 27706939

[ref29] Hamel J. D.; Paquin J.-F. Activation of C–F Bonds α to C–C Multiple Bonds. Chem. Commun. 2018, 54, 10224–10239. 10.1039/C8CC05108A. 30124701

[ref30] Trost B. M.; Gholami H.; Zell D. Palladium-catalyzed Asymmetric Allylic Fluoroalkylation/Trifluoromethylation. J. Am. Chem. Soc. 2019, 141, 11446–11451. 10.1021/jacs.9b06231. 31280565

[ref31] Butcher T. W.; Wang J. L.; Amberg W. M.; Watkins N. B.; Wilkinson N. D.; Hartwig J. F. Desymmetrization of Difluoromethylene Groups by C–F Bond Activation. Nature 2020, 583, 548–553. 10.1038/s41586-020-2399-1. 32480398 PMC10484566

[ref32] Sugihara N.; Suzuki K.; Nishimoto Y.; Yasuda M. Photoredox-Catalyzed C–F Bond Allylation of Perfluoroalkylarenes at the Benzylic Position. J. Am. Chem. Soc. 2021, 143, 9308–9313. 10.1021/jacs.1c03760. 34075740

[ref33] Li D.; Shen C.; Si Z.; Liu L. Palladium-Catalyzed Fluorinative Bifunctionalization of Aziridines and Azetidines with gem-Difluorocyclopropanes. Angew. Chem., Int. Ed. 2023, 62, e202310283 10.1002/anie.202310283. 37572320

[ref34] Amii H.; Uneyama K. C-F Bond Activation in Organic Synthesis. Chem. Rev. 2009, 109, 2119–2183. 10.1021/cr800388c. 19331346

[ref35] Olah G. A.; Kuhn S. J. Selective Friedel-Crafts Reactions. I. Boron Halide Catalyzed Haloalkylation of Benzene and Alkylbenzenes with Fluorohaloalkanes. J. Org. Chem. 1964, 29, 2317–2320. 10.1021/jo01031a051.

[ref36] Csókás D.; Gupta R.; Prasad P. K.; Goh K. K. K.; Young R. D. Insights into the Mechanism of Aluminum-Catalyzed Halodefluorination. J. Org. Chem. 2023, 88, 4397–4404. 10.1021/acs.joc.2c03005. 36926911

[ref37] Zerban J. J.; Bagnall B.; Davis T. A. Enhancing the Leaving Group Ability of Alkyl Fluorides: I/F Exchange Reactions Mediated by LiI. Tetrahedron Lett. 2022, 91, 153639 10.1016/j.tetlet.2022.153639.

[ref38] Mandal D.; Gupta R.; Young R. D. Selective Monodefluorination and Wittig Functionalization of gem-Difluoromethyl Groups to Generate Monofluoroalkenes. J. Am. Chem. Soc. 2018, 140, 10682–10686. 10.1021/jacs.8b06770. 30119600

[ref39] Caputo C. B.; Stephan D. W. Activation of Alkyl C–F Bonds by B(C_6_F_5_)_3_: Stoichiometric and Catalytic Transformations. Organometallics 2012, 31, 27–30. 10.1021/om200885c.

[ref40] Mizukami Y.; Song Z.; Takahashi T. Halogen Exchange Reaction of Aliphatic Fluorine Compounds with Organic Halides as Halogen Source. Org. Lett. 2015, 17, 5942–5945. 10.1021/acs.orglett.5b02589. 26629792

[ref41] Mancinelli J. P.; Kong W.-Y.; Guo W.; Tantillo D. J.; Wilkerson-Hill S. M. Borane-Catalyzed C–F Bond Functionalization of gem-Difluorocyclopropenes Enables the Synthesis of Orphaned Cyclopropanes. J. Am. Chem. Soc. 2023, 145, 17389–17397. 10.1021/jacs.3c05278. 37494703

[ref42] Koo J.; Kim W.; Jhun B. H.; Park S.; Song D.; You Y.; Lee H. G. Halogen Atom Transfer-Induced Homolysis of C–F Bonds by the Excited-State Boryl Radical. J. Am. Chem. Soc. 2024, 146, 22874–22880. 10.1021/jacs.4c06337. 39093360

[ref43] Dryzhakov M.; Moran J. Autocatalytic Friedel–Crafts Reactions of Tertiary Aliphatic Fluorides Initiated by B(C_6_F_5_)_3_·H_2_O. ACS Catal. 2016, 6, 3670–3673. 10.1021/acscatal.6b00866.

[ref44] Gupta R.; Mandal D.; Jaiswal A. K.; Young R. D. FLP-Catalyzed Monoselective C–F Functionalization in Polyfluorocarbons at Geminal or Distal Sites. Org. Lett. 2021, 23, 1915–1920. 10.1021/acs.orglett.1c00346. 33624500

[ref45] Mandal D.; Gupta R.; Jaiswal A. K.; Young R. D. Frustrated Lewis-Pair-Meditated Selective Single Fluoride Substitution in Trifluoromethyl Groups. J. Am. Chem. Soc. 2020, 142, 2572–2578. 10.1021/jacs.9b12167. 31935080

[ref46] Gupta R.; Csókás D.; Lye K.; Young R. D. Experimental and Computational Insights into the Mechanism of FLP Mediated Selective C–F Bond Activation. Chem. Sci. 2023, 14, 1291–1300. 10.1039/D2SC05632A. 36756325 PMC9891352

[ref47] Willcox D. R.; Nichol G. S.; Thomas S. P. Borane-Catalyzed C(sp3)–F Bond Arylation and Esterification Enabled by Transborylation. ACS Catal. 2021, 11, 3190–3197. 10.1021/acscatal.1c00282.

[ref48] Panisch R.; Bolte M.; Mueller T. Hydrogen- and Fluorine-Bridged Disilyl Cations and Their Use in Catalytic C–F Activation. J. Am. Chem. Soc. 2006, 128, 9676–9682. 10.1021/ja061800y. 16866520

[ref49] Douvris C.; Ozerov O. V. Hydrodefluorination of Perfluoroalkyl Groups Using Silylium-Carborane Catalysts. Science 2008, 321, 1188–1190. 10.1126/science.1159979. 18755971

[ref50] Caputo C. B.; Hounjet L. J.; Dobrovetsky R.; Stephan D. W. Lewis Acidity of Organofluorophosphonium Salts: Hydrodefluorination by a Saturated Acceptor. Science 2013, 341, 1374–1377. 10.1126/science.1241764. 24052304

[ref51] Scott V. J.; Celenligil-Cetin R.; Ozerov O. V. Room-Temperature Catalytic Hydrodefluorination of C(sp^3^)–F Bonds. J. Am. Chem. Soc. 2005, 127, 2852–2853. 10.1021/ja0426138. 15740111

[ref52] Yoshida S.; Shimomori K.; Kim Y.; Hosoya T. Single C–F Bond Cleavage of Trifluoromethylarenes with an ortho-Silyl Group. Angew. Chem., Int. Ed. 2016, 55, 10406–10409. 10.1002/anie.201604776. 27312982

[ref53] Mallov I.; Ruddy A. J.; Zhu H.; Grimme S.; Stephan D. W. C–F Bond Activation by Silylium Cation/Phosphine Frustrated Lewis Pairs: Mono-Hydrodefluorination of PhCF_3_, PhCF_2_H and Ph_2_CF_2_. Chem. Eur. J. 2017, 23, 17692–17696. 10.1002/chem.201705276. 29116666

[ref54] Okusu S.; Okazaki H.; Tokunaga E.; Soloshonok V. A.; Shibata N. Organocatalytic Enantioselective Nucleophilic Alkynylation of Allyl Fluorides Affording Chiral Skipped Ene-ynes. Angew. Chem., Int. Ed. 2016, 55, 6744–6748. 10.1002/anie.201601928. 27111713

[ref55] Tanaka J.; Suzuki S.; Tokunaga E.; Haufe G.; Shibata N. Asymmetric Desymmetrization via Metal-Free C–F Bond Activation: Synthesis of 3,5-Diaryl-5-fluoromethyloxazolidin-2-ones with Quaternary Carbon Centers. Angew. Chem., Int. Ed. 2016, 55, 9432–9436. 10.1002/anie.201603210. 27332650

[ref56] Zi Y.; Lange M.; Schultz C.; Vilotijevic I. Latent Nucleophiles in Lewis Base Catalyzed Enantioselective N-Allylations of N-Heterocycles. Angew. Chem., Int. Ed. 2019, 58, 10727–10731. 10.1002/anie.201903392. 31063225

[ref57] Terao J.; Nakamura M.; Kambe N. Non-catalytic Conversion of C–F Bonds of Benzotrifluorides to C–C Bonds Using Organoaluminium Reagents. Chem. Commun. 2009, 6011–6013. 10.1039/b915620h. 19809627

[ref58] Gu W.; Haneline M. R.; Douvris C.; Ozerov O. V. Carbon–Carbon Coupling of C(sp^3^)–F Bonds Using Alumenium Catalysis. J. Am. Chem. Soc. 2009, 131, 11203–11212. 10.1021/ja903927c. 19722677

[ref59] Terao J.; Begum S. A.; Shinohara Y.; Tomita M.; Naitoh Y.; Kambe N. Conversion of a (sp^3^)C–F Bond of Alkyl Fluorides to (sp^3^)C–X (X = Cl, C, H, O, S, Se, Te, N) Bonds Using Organoaluminium Reagents. Chem. Commun. 2007, 855–857. 10.1039/B613641A. 17308654

[ref60] Stahl T.; Klare H. F. T.; Oestreich M. Main-Group Lewis Acids for C–F Bond Activation. ACS Catal. 2013, 3, 1578–1587. 10.1021/cs4003244.

[ref61] Crimmin M. R.; Butler M. J.; White A. J. P. Oxidative Addition of Carbon–Fluorine and Carbon–Oxygen Bonds to Al(I). Chem. Commun. 2015, 51, 15994–15996. 10.1039/C5CC07140B. PMC4621529 26389715

[ref62] Chu T.; Boyko Y.; Korobkov I.; Nikonov G. I. Transition Metal-like Oxidative Addition of C–F and C–O Bonds to an Aluminum(I) Center. Organometallics 2015, 34, 5363–5365. 10.1021/acs.organomet.5b00793.

[ref63] Pitsch C. E.; Wang X. Aluminum(I) β-Diketiminato Complexes Activate C(sp^2^)–F and C(sp^3^)–F Bonds by Different Oxidative Addition Mechanisms: a DFT Study. Chem. Commun. 2017, 53, 8196–8198. 10.1039/C7CC03503A. 28681885

[ref64] Coates G.; Rekhroukh F.; Crimmin M. R. Breaking Carbon–Fluorine Bonds with Main Group Nucleophiles. Synlett 2019, 30, 2233–2246. 10.1055/s-0039-1690738.

[ref65] Fujii I.; Semba K.; Li Q.-Z.; Sakaki S.; Nakao Y. Magnesiation of Aryl Fluorides Catalyzed by a Rhodium–Aluminum Complex. J. Am. Chem. Soc. 2020, 142, 11647–11652. 10.1021/jacs.0c04905. 32515952

[ref66] Kysliak O.; Görls H.; Kretschmer R. C–F Bond Activation by Pentamethylcyclopentadienyl-Aluminium(I): A Combined Experimental/Computational Exercise. Chem. Commun. 2020, 56, 7865–7868. 10.1039/D0CC00003E. 32037419

[ref67] Kurumada S.; Takamori S.; Yamashita M. An Alkyl-substituted Aluminium Anion with Strong Basicity and Nucleophilicity. Nat. Chem. 2020, 12, 36–39. 10.1038/s41557-019-0365-z. 31767993

[ref68] Judge N. R.; Logallo A.; Hevia E. Main Group Metal-mediated Strategies for C–H and C–F Bond Activation and Functionalisation of Fluoroarenes. Chem. Sci. 2023, 14, 11617–11628. 10.1039/D3SC03548D. 37920337 PMC10619642

[ref69] Liu X.; Dong S.; Zhu J.; Inoue S. Dialumene as a Dimeric or Monomeric Al Synthon for C–F Activation in Monofluorobenzene. J. Am. Chem. Soc. 2024, 146, 23591–23597. 10.1021/jacs.4c08171. 39165246 PMC11345846

[ref70] Sandford C.; Rasappan R.; Aggarwal V. K. Synthesis of Enantioenriched Alkylfluorides by the Fluorination of Boronate Complexes. J. Am. Chem. Soc. 2015, 137, 10100–10103. 10.1021/jacs.5b05848. 26244235

[ref71] Lovett G. H.; Chen S.; Xue X.-S.; Houk K. N.; MacMillan D. W. C. Open-Shell Fluorination of Alkyl Bromides: Unexpected Selectivity in a Silyl Radical-Mediated Chain Process. J. Am. Chem. Soc. 2019, 141, 20031–20036. 10.1021/jacs.9b11434. 31774670

[ref72] Wang Z.-Y.; Freas D. J.; Fu G. C. Phosphine Catalysis of the Fluorination of Unactivated Tertiary Alkyl Chlorides under Mild and Convenient Conditions. J. Am. Chem. Soc. 2023, 145, 25093–25097. 10.1021/jacs.3c11042. 37939003 PMC10942731

[ref73] Chen F.; Zhang Q.; Li Y.; Yu Z.-X.; Chu L. Selective Hydrofunctionalization of Alkenyl Fluorides Enabled by Nickel-Catalyzed Hydrogen Atoms and Group Transfer: Reaction Development and Mechanistic Study. J. Am. Chem. Soc. 2024, 146, 11418–11431. 10.1021/jacs.4c01506. 38621358

[ref74] Ortalli S.; Ford J.; Trabanco A. A.; Tredwell M.; Gouverneur V. Photoredox Nucleophilic (Radio)fluorination of Alkoxyamines. J. Am. Chem. Soc. 2024, 146, 11599–11604. 10.1021/jacs.4c02474. 38651661 PMC11066844

[ref75] Lee C.; Kim M.; Han S.; Kim D.; Hong S. Nickel-Catalyzed Hydrofluorination in Unactivated Alkenes: Regio- and Enantioselective C–F Bond Formation. J. Am. Chem. Soc. 2024, 146, 9375–9384. 10.1021/jacs.4c01548. 38512796

[ref76] Hooker L. V.; Bandar J. S. Synthetic Advantages of Defluorinative C–F Bond Functionalization. Angew. Chem., Int. Ed. 2023, 62, e202308880 10.1002/anie.202308880. PMC10843719 37607025

[ref77] Middleton W. J. New Fluorinating Reagents. Dialkylaminosulfur Fluorides. J. Org. Chem. 1975, 40, 574–578. 10.1021/jo00893a007.

[ref78] Krieck S.; Görls H.; Westerhausen M. Synthesis and Properties of Calcium Tetraorganylalanates with [Me_4–n_AlPh_n_]^−^ Anions. Organometallics 2008, 27, 5052–5057. 10.1021/om800509p.

[ref79] Jiang S.; Xie Y.; Xie Y.; Yu L.-J.; Yan X.; Zhao F.-G.; Mudugamuwa C. J.; Coote M. L.; Jia Z.; Zhang K. Lewis Acid-Induced Reversible Disproportionation of TEMPO Enables Aqueous Aluminum Radical Batteries. J. Am. Chem. Soc. 2023, 145, 14519–14528. 10.1021/jacs.3c04203. 37350446

[ref80] Finkelstein H. Darstellung Organischer Jodide aus den Entsprechenden Bromiden und Chloriden. Ber. Dtsch. Chem. Ges. 1910, 43, 1528–1532. 10.1002/cber.19100430257.

[ref81] Sunagatullina A.; Lutter F.; Knochel P. Preparation of Primary and Secondary Dialkylmagnesiums by a Radical I/Mg-Exchange Reaction Using sBu_2_Mg in Toluene. Angew. Chem., Int. Ed. 2022, 61, e202116625 10.1002/anie.202116625. PMC9302629 35044040

[ref82] Franz M. H.; Röper S.; Wartchow R.; Hoffmann H. M. R. The First and Second Cinchona Rearrangement. Two Fundamental Transformations of Alkaloid Chemistry. J. Org. Chem. 2004, 69, 2983–2991. 10.1021/jo030363s. 15104435

[ref83] Boratynski P. J.; Turowska-Tyrk I.; Skarzewski J. Stereoselective C9 Arylation and Vinylation of Cinchona Alkaloids. Org. Lett. 2008, 10, 385–388. 10.1021/ol7026625. 18179221

[ref84] Posner G. H.; Ting J.-S.; Lentz C. M. A Mechanistic and Synthetic Study of Organocopper Substitution Reactions with Some Homoallylic and Cyclopropylcarbinyl Substrates: Application to Isoprenoid Synthesis. Tetrahedron 1976, 32, 2281–2287. 10.1016/0040-4020(76)88002-7.

