# Supplementary material for: Cryogenic Organometallic Carbon–Fluoride Bond Functionalization with Broad Functional Group Tolerance
Source: J Am Chem Soc. 2025 Feb 6;147(7):5764–74. doi: 10.1021/jacs.4c13956 (PMC11848826; doi:10.1021/jacs.4c13956)
Supplement: Supplementary file 1 — ja4c13956_si_001.pdf [file ja4c13956_si_001.pdf]

Supporting Information

Cryogenic Organometallic Carbon-Fluoride Bond Functionalization with Broad Functional Group Tolerance

D. Lucas Kane, Bryan C. Figula, Kaluvu Balaraman, Jeffery A. Bertke and Christian Wolf*

Georgetown University, Chemistry Department, Washington, DC 20057, USA

Table of Contents:

1. **General Information and Materials** **S2**
2. **Synthesis and Use of Organoaluminum Compounds** **S3**
3. **Competition Studies** **S5**
   - **3.1. Primary Alkyl Halide Competition** **S6**
   - **3.2. Secondary Alkyl Halide Competition** **S7**
   - **3.3. Tertiary Alkyl Halide Competition** **S9**
4. **Optimization Studies** **S11**
5. **General Procedures for C-C Coupling of Alkyl Fluorides** **S15**
6. **Mechanistic Studies** **S18**
   - **6.1. Friedel-Crafts Trapping Experiments** **S18**
   - **6.2. 1°, 2°, 3° Alkyl Fluoride Competition Experiments** **S22**
   - **6.3. General Procedure for AlX_3 Reactions** **S26**
   - **6.4. X-ray Characterization of a Carbocationic Intermediate** **S31**
   - **6.5. Studies with the Radical Scavengers TEMPO and 9,10-Dihydroanthracene** **S33**
   - **6.6. Stereochemical Analysis of the Reaction with (*S*)-(3-Fluorobutyl)benzene** **S39**

6.7. Synthesis of the Cesium-bridged Triphenylfluoroaluminate Dimer 28 and Electrophile Arylation **S41**

7. Alkyl Fluoride Selectivity Studies **S46**

8. Coupling of Alkyl Chlorides, Bromides and Iodides with Ph_3Al at 50 °C and 100 °C **S52**

9. Product Synthesis, Purification and Characterization **S59**

10. ^{1}H, ^{13}C and ^{19}F NMR Spectra **S92**

11. X-Ray Crystallography **S149**

12. References **S158**

1. General Information and Materials

The preparation and handling of air-sensitive chemicals were performed under nitrogen atmosphere using a glovebox or with standard Schlenk techniques. ^{1}H (400 MHz), $^{13}C\{1H\}$ (100 MHz) and ^{19}F NMR (376 MHz) NMR spectra were recorded at room temperature in deuterated chloroform. The chemical shift (δ) values are expressed in ppm relative to the residual signals of the solvent used. For reactions that require heating, a metallic beads bath was used as the heat source. All solvents, commercially available reagents, alkyl fluorides, triphenylaluminum (1.0 M in dibutylether) and trialkylaluminum reagents (solutions in hexanes) were used as purchased. Other alkyl fluorides were prepared in one step from the corresponding alcohol using diethylamino sulfurtrifluoride or via electrophilic fluorination according to routine literature protocols. Solvents were stored over 4 Å molecular sieves prior to use. Reaction products were purified by flash column chromatography on silica gel as described below. GC-MS measurements were acquired on an Agilent 5977C GC/MSD equipped with an HP-5ms Ultra Inert (5%-phenyl)methylpolysiloxane column (30 m, 0.25 mm, 0.25 μm). HR-ESI-MS data were obtained using electron spray ionization time-of-flight (ESI-TOF) spectrometry. Chiral HPLC was

performed using a CHIRALCEL OJ-H column. Enantioselective GC-MS analysis was conducted on a 2,6-dimethyl-3-pentyl-γ-cyclodextrin chiral stationary phase. Single crystals of each compound were mounted under parabar oil on a Mitegen micromount and immediately placed in a cold nitrogen stream at 100(2) K prior to data collection. Data were collected a Bruker DUO equipped with an APEXII CCD detector and Mo fine-focus sealed source. Data were integrated with the Bruker SAINT program. Structure solution and refinement was performed using the SHELXTL/PC suite and ShelXle. Intensities were corrected for Lorentz and polarization effects and an empirical absorption correction was applied using Blessing's method as incorporated into the program SADABS. Non-hydrogen atoms were refined with anisotropic thermal parameters. Hydrogen atoms were included in idealized positions unless otherwise noted.

2. Synthesis and Use of Organoaluminum Compounds

All reactions with trialkylaluminum reagents were performed using commercially available solutions in hexanes. The trialkylaluminum solutions were employed as received in the synthesis of products **80-87**, **89**, **91**, **98** and **101**. Commercially available triphenylaluminum in dibutyl ether solutions were used as received for the formation of **6**, **61**, **62**, **66-70**, **73**, **77**, **92**, **95**, **97** and **100**. We observed that primary and secondary alkyl fluorides form by-products (see 4. Optimization Studies) with dibutyl ether. For those substrates or when the organoaluminum compounds were not commercially available, fresh aluminum reagents in chlorobenzene were prepared as described below.

Step 1: In a nitrogen filled glovebox, iodobenzene (1.00 mL, 8.97 mmol) was dissolved in pentane (~40 mL) and cooled to -40 °C. *n*-BuLi (2.5 M in hexanes, 3.59 mL, 8.97 mmol, 1 equiv.) was cooled to -40 °C and added. The solution was hand-swirled for about 10 seconds, until a white

precipitate crashed out. The solution was immediately filtered through a frit and the solid was washed with 3 portions of 20 mL of pentane and dried to give phenyllithium (47%, 351.4 mg, 4.18 mmol). The solid was used without further purification. Mesityllithium was prepared according to the above procedure from mesityliodide and *n*-BuLi. Styryllithium was prepared according to the above procedure from (*E*)-bromostyrene and *sec*-BuLi. Alkynyllithium compounds were prepared according to a literature procedure.[1]

Step 2: In a nitrogen filled glovebox, phenyllithium (300.0 mg, 3.57 mmol, 1 equiv.) was dissolved in chlorobenzene (3.31 mL). Solid aluminum trichloride (158.6 mg, 1.19 mmol, 0.33 equiv.) was added and the reaction was heated to 50 °C for 16 hours. The reaction was filtered and the resulting triphenylaluminum solution (359.0 mM) was used without further purification. Trimesitylaluminum, tri((*E*)-styryl)aluminum, tri(phenylethynyl)aluminum, and tri(4-phenylbut-1-yn-1-yl)aluminum were prepared from the corresponding organolithium compounds according to the above procedure. These solutions were used assuming that the transmetalation is quantitative. This was confirmed by complete alkyl fluoride conversion using 1/3 equivalent of the calculated Ph_3Al amount. After approximately 1-2 weeks of storage the solutions turned cloudy due to formation of $(Ph_3Al)_2$ which was confirmed by crystallographic analysis of the precipitate. The reactions were sluggish, producing substantial amounts of rearrangement, dimerization and Friedel-Crafts byproducts when cloudy solutions were tested. Cloudy solutions were therefore not used for C-F bond functionalization. We note that the organoaluminum solutions prepared from organolithium precursors may contain residual LiCl which can affect the aggregation and reactivity of organometallic compounds, however, approximately half of the C-F bond functionalizations described herein were conducted with LiCl-free, commercially available triaryl- or trialkylaluminum solutions giving similar results. Alternatively, Ph_3Al can be formed via

transmetalation from Me_3Al and Ph_3B forming gaseous Me_3B as follows: In a 10 mL vessel, Ph_3B (173.7 mg, 0.717 mmol) and Me_3Al (2.0 M in hexane, 0.717 mmol) where combined in 2 mL of chlorobenzene. The vessel was heated to 100 °C and allowed to stir while connected to a Schlenk line for 48 hours. After cooling to room temperature, the vessel was then sealed and brought back into a glove box where the reaction mixture was filtered, stored at -40 °C, and used as is. This alternative method gives Ph_3Al solutions albeit in lower purity according to NMR and GC-MS analysis (for example, biphenyl and $MeBPh_2$ were identified). Using this solution, we observed 95% conversion of octyl fluoride to 1-phenyloctane at -40 ºC albeit with small amounts of rearrangement by-products.

3. Competition Studies

To investigate the selectivity of triphenylaluminum towards carbon-halogen bond functionalization of alkyl fluorides, competition studies were carried out using complete series of primary, secondary and tertiary alkyl halides. The reactions were carried out at -40 °C for six hours, after which they were analyzed by GC-MS or NMR. Perfluorobenzene was used as an internal standard. Alkyl chlorides, bromides, and iodides are entirely preserved in all cases, and the alkyl fluorides were completely consumed. Note that 1-iodoadamantane was not used due to its instability.

3.1. Primary Alkyl Halide Competition

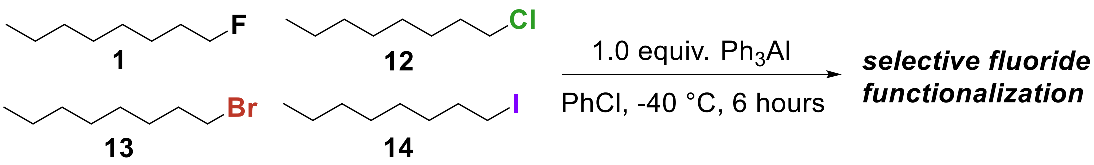

Figure S1. The competition between 1-fluorooctane (**1**), 1-chlorooctane (**12**), 1-bromooctane (**13**), and 1-iodooctane (**14**) in the presence of triphenylaluminum.

In a nitrogen-filled glove box, 1-fluorooctane (10.0 mg, 0.075 mmol), 1-chlorooctane (11.0 mg, 0.075 mmol), 1-bromooctane (15.0 mg, 0.075 mmol), and 1-iodooctane (18.0 mg, 0.075 mmol) were combined and chilled to -40 °C. An aliquot was taken for NMR analysis prior to addition of the aluminum reagent. Triphenylaluminum solution (359.0 mM in chlorobenzene, 0.211 mL, 0.075 mmol, 1.0 equiv.) was added, and the resulting solution was allowed to stir at -40 °C for six hours. The reaction mixture was quenched with methanol, and an aliquot was taken for NMR analysis. The chloride, bromide and iodide did not react, while the alkyl fluoride was fully consumed.

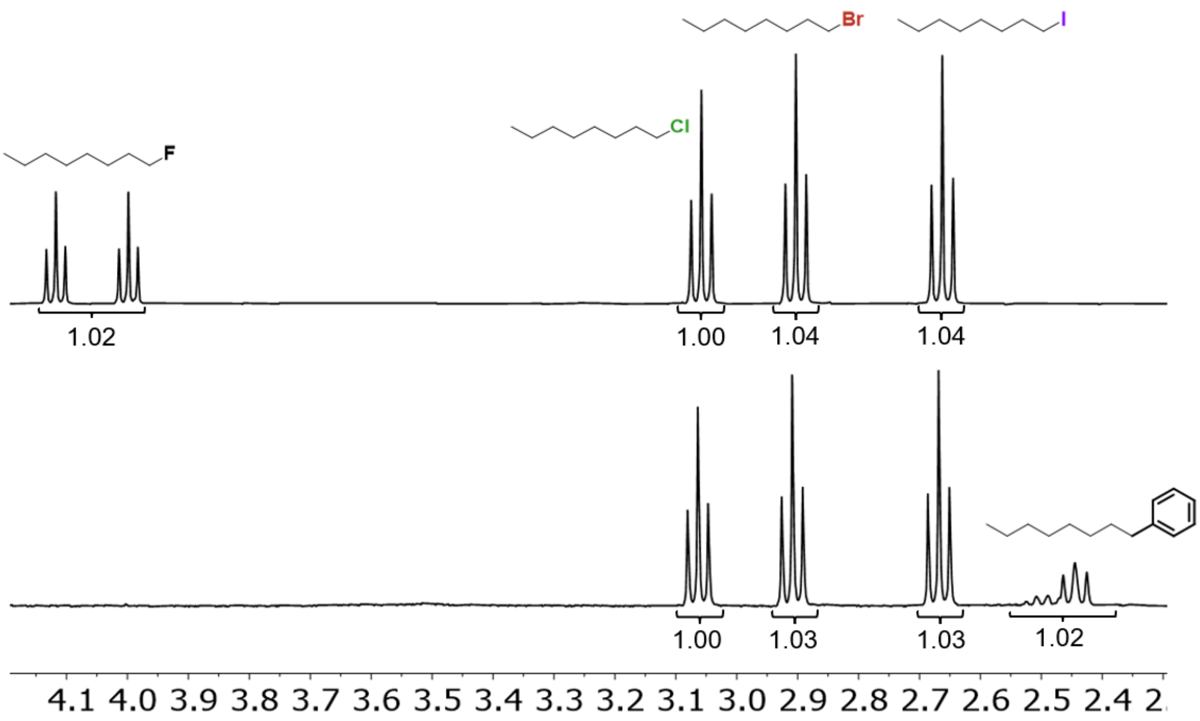

Figure S2. NMR analysis of the competition study between 1-fluorooctane, 1-chlorooctane, 1-bromooctane, and 1-iodooctane in the presence of triphenylaluminum. Top: NMR prior to Ph_3Al addition. Bottom: NMR obtained after six hours at -40 °C.

3.2. Secondary Alkyl Halide Competition

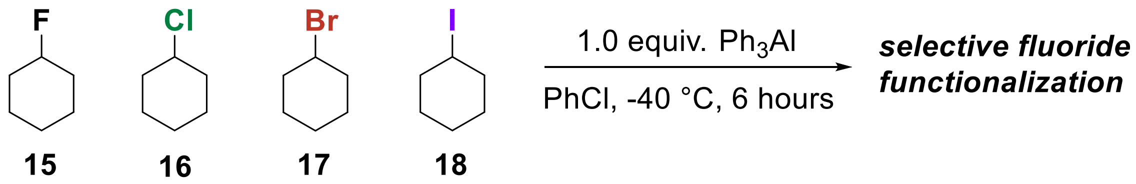

Figure S3. Competition between fluorocyclohexane (**15**), chlorocyclohexane (**16**), bromocyclohexane (**17**), and iodocyclohexane (**18**) in the presence of triphenylaluminum.

In a nitrogen-filled glove box, fluorocyclohexane (10.0 mg, 0.098 mmol), chlorocyclohexane (11.0 mg, 0.098 mmol), bromocyclohexane (16.0 mg, 0.098 mmol), and iodocyclohexane (21.0 mg, 0.098 mmol) were combined and chilled to -40 °C. Perfluorobenzene (10.0 μL) was added as an internal standard, and an aliquot was taken for GC-MS analysis prior to Ph_3Al addition. Triphenylaluminum solution (359.0 mM in chlorobenzene, 0.273 mL, 0.098 mmol, 1.0 equiv.) was added, and the resulting solution was allowed to stir at -40 °C for six hours. The reaction mixture was quenched with methanol, and an aliquot was taken for GC-MS analysis. Integration of the C_6F_6 peak vs. the alkyl halide peaks before and after addition of Ph_3Al reveals that the chloride, bromide and iodide are perfectly preserved, while the alkyl fluoride is fully consumed.

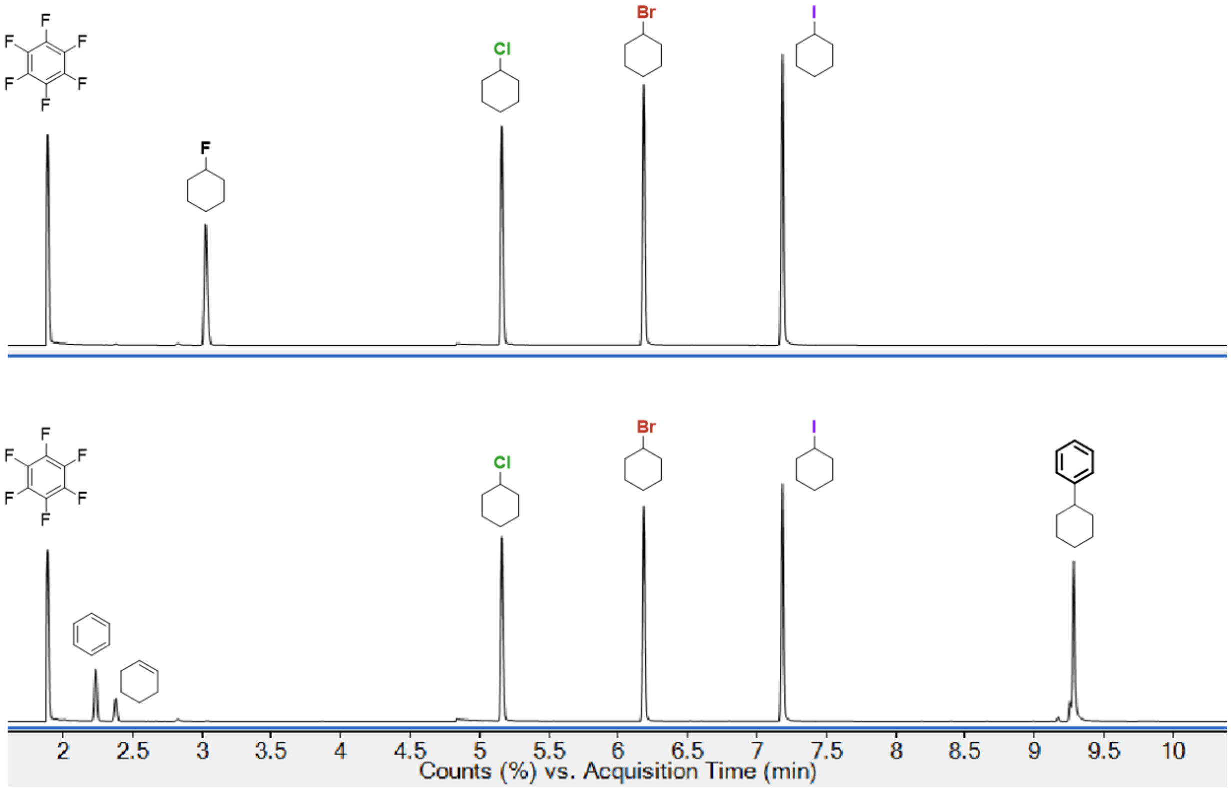

Figure S4. GC analysis of the competition study between fluorocyclohexane, chlorocyclohexane, bromocyclohexane, and iodocyclohexane in the presence of triphenylaluminum. Top: GC prior to Ph_3Al addition. Bottom: GC obtained after six hours at -40 °C. Benzene is observed as a

decomposition product of triphenylaluminum. Small amounts of cyclohexene, probably formed via HF elimination, are observed.

3.3. Tertiary Alkyl Halide Competition

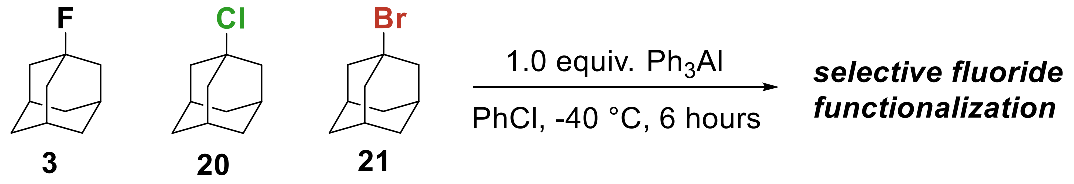

Figure S5. Competition between 1-fluoroadamantane (**3**), 1-chloroadamantane (**20**) and 1-bromoadamantane (**21**) in the presence of triphenylaluminum.

In a nitrogen-filled glove box, 1-fluoroadamantane (10.0 mg, 0.065 mmol), 1-chloroadamantane (11.0 mg, 0.065 mmol) and 1-bromoadamantane (14.0 mg, 0.065 mmol) were combined in 100.0 µL of dibutylether and chilled to -40 °C. Perfluorobenzene (10.0 µL) was added as an internal standard, and an aliquot was taken for GC-MS analysis prior to Ph_3Al addition. Triphenylaluminum solution (359.0 mM in chlorobenzene, 0.18 mL, 0.065 mmol, 1.0 equiv.) was added, and the resulting solution was allowed to stir at -40 °C for six hours. The reaction mixture was quenched with methanol, and aliquots were taken for GC-MS analysis. Integration of the C_6F_6 peak vs. the alkyl halide peaks before and after addition of Ph_3Al reveals that the chloride and bromide are perfectly preserved, while the alkyl fluoride is fully consumed. Note that 1-iodoadamantane was not used due to its instability.

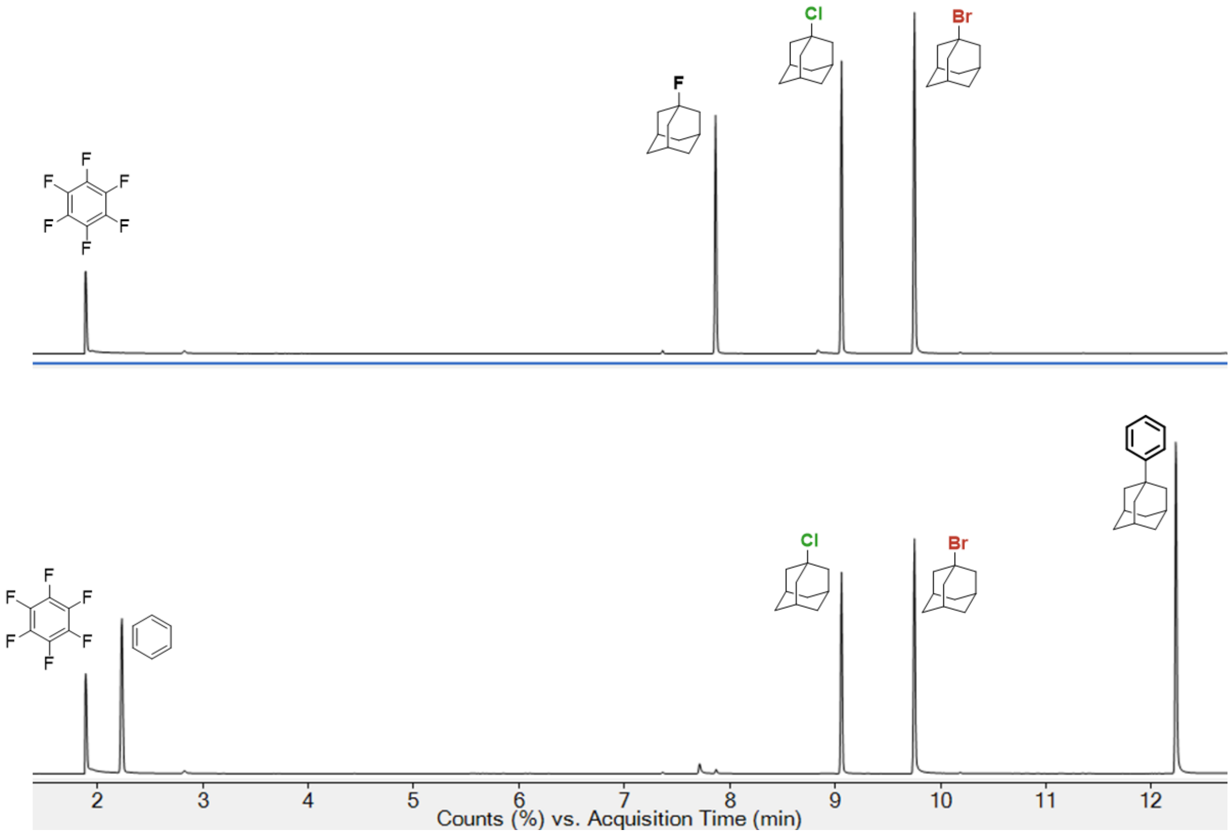

Figure S6. GC analysis of the competition study between 1-fluoroadamantane, 1-chloroadamantane, and 1-bromoadamantane in the presence of triphenylaluminum. Top: GC prior to Ph_3Al addition. Bottom: GC obtained after six hours at -40 °C. Benzene is observed as a decomposition product of triphenylaluminum.

4. Optimization Studies

Tertiary Alkyl Fluorides

We used adamantyl fluoride to optimize the cross coupling reaction. The C-C bond formation proved to proceed with quantitative conversion at both cryogenic and room temperatures. If desirable, one can use the triarylaluminum reagent in substoichiometric amounts as all aryl groups are transferred.

Table S1. Optimization of the reaction of tertiary fluorides and Ph_3Al.

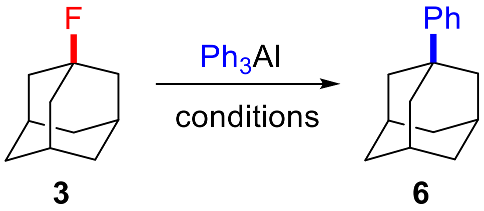

Entry	Ph_3Al (Equiv.)	Temperature	Time	Conversion (%)[a]
1	1	25 °C	16 h	100
2	1	-40 °C	16 h	100
3	1	-40 °C	1 h	100
4	0.33	25 °C	16 h	100[b]
5	0.33	-40 °C	16 h	25[b]
6	0.5	-40 °C	1 h	91
7	1	-78 °C	3 h	100[c]
8	1	-40 °C	5 min	100 (88)[d]
9	0.5	-40 °C	2 h	100 (91)[d]

[a]0.06 mmol **3** used. Ph_3Al 1.0 M in DBE. Reactions were quenched with methanol and conversions were determined by GC-MS. [b] 0.1 mL extra DBE added. [c] 90% conversion after 2 hours. [d] 0.25 mmol **3** used. Parentheses indicate isolated yield.

Primary Alkyl Fluorides

The optimization of the reaction with primary alkyl fluorides proved to be more difficult than with tertiary substrates. Addition of triphenylaluminum in dibutyl ether to 1-chloro-4-(3-

fluoropropyl)benzene **102** showed 33% conversion to the arylated species **53** and 67% conversion to the *n*-butoxy-coupled product **103** (Table S2, entry 1). This C-O coupling product is likely formed via coordination of dibutyl ether to the intermediate carbocation (Figure S7). The resulting oxonium cation releases a butyl cation, which can be captured by the aluminate complex. This process forms butylbenzene, an observed byproduct in this reaction. To avoid C-O coupling, triphenylaluminum was prepared in the absence of any ether solvents from phenyllithium and aluminum trichloride. The reaction in noncoordinating solvents was screened, with pentane, benzene, chlorobenzene, and 1,2-dichlorobenzene giving the most favorable results (entries 2,4,6, and 8). Recognizing the advantage of running reactions at lower temperatures and the need of slightly polar solvents to dissolve alkyl fluorides, chlorobenzene was chosen as the preferred solvent. Reactions with one equivalent or less of the aluminum reagent showed remaining starting material (entries 10-15), however, full conversion to **57** with minimal rearrangement and 80% isolated yield was observed with 1.5 equivalents (entry 19).

Table S2. Optimization of the reaction between 1-chloro-4-(3-fluoropropyl)benzene and Ph_3Al.

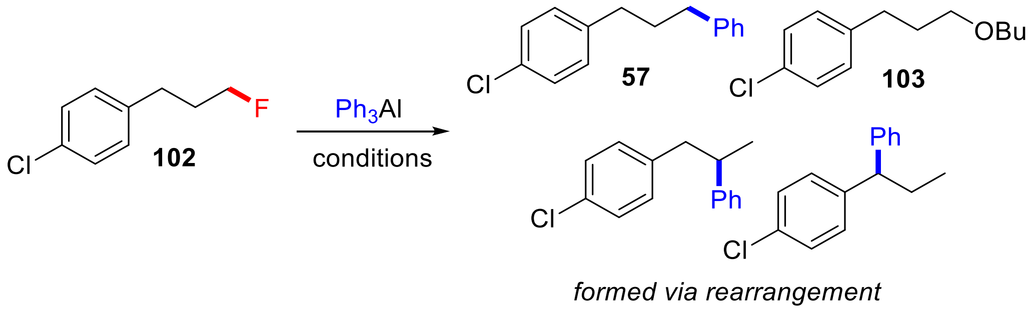

Entry	Ph_3Al solvent	Ph_3Al (Equiv.)	Temperature	Time	Conversion (%)[a]	Rearrangement (%)
1	DBE	1.0	25 °C	16 h	67[b]	0
2	Pentane	1.0	25 °C	16 h	100	10
3	Toluene	1.0	25 °C	16 h	75	10
4	Benzene	1.0	25 °C	16 h	100	10
5	Benzene	1.0	25 °C	1 h	100	10

6	1,2-$Cl_2C_6H_4$	1.0	25 °C	1 h	100	15
7	1,2-$Cl_2C_6H_4$	1.0	-40 °C	1 h	100	15
8	PhCl	1.0	-40 °C	1 h	100	10
9	PhCN	1.0	-40 °C	1 h	0	N/A
10	PhCl	0.5	-40 °C	3 h	52	19
11	PhCl	0.5	-40 °C	6.5 h	57	18
12	PhCl	0.5	-40 °C	23 h	84	28
13	PhCl/ Pentane (1:2)	1.0	-78 °C	3 h	25	10
14	PhCl	1.0	-40 °C	1.5 h	83	23
15	PhCl	1.0	-40 °C	6.5 h	87	27
16	PhCl	1.5	-40 °C	5 min	89	10
17	PhCl	1.5	-40 °C	15 min	93	10
18	PhCl	1.5	-40 °C	45 min	96	10
19	PhCl	1.5	-40 °C	1.5 h	100 (80)[c]	10

[a]0.05 mmol **3** used. The concentrations of Ph_3Al solutions were 1.0 M (DBE), 225.0 mM (pentane, toluene, and benzene), 150.0 mM (1,2-dichlorobenzene), and 359.0 mM (PhCl). Conversions were determined by GC-MS. [b] 33% desired product **53,** 67% *n*-butoxylated product **104**. [c] 0.25 mmol **103** used. Parentheses indicate isolated yield.

Figure S7. Proposed mechanism for C-O coupling of primary alkyl fluorides using Ph_3Al in DBE.

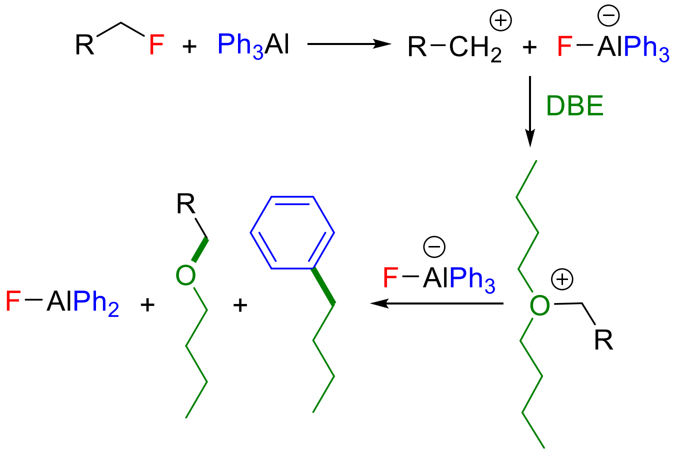

Secondary Alkyl Fluorides

The reaction with secondary fluorides was optimized using 3-fluoro-1-phenylbutane **2** and triphenylaluminum solutions in chlorobenzene. Unlike primary fluorides, secondary fluorides showed a higher propensity towards elimination and rearrangements. It was found that these side reactions could be limited at lower temperatures (Table S3, entries 3, 4, and 9). The triphenylaluminum solution, however, freezes at temperatures below -50 °C (entries 14 and 15). Addition of triphenylaluminum to the alkyl fluoride cooled to -78 °C gives approximately 50% conversion prior to freezing (entry 10). To ensure that the reaction occurs at -50 °C, individual frozen layers of the alkyl fluoride in chlorobenzene and the triphenylaluminum solution on top of each other were produced and allowed to slowly mix and warm to room temperature (entry 12). However, high amounts of rearrangement persisted. To reach lower temperatures, pentane was used as a solvent to dissolve the alkyl fluoride **2** thus allowing for homogenous reaction conditions at -78 °C and to reduce byproduct formation (entry 21). Optimized conditions include 1.5 equivalents of triphenylaluminum, a pentane co-solvent and 30-minute reaction times to give **5** in 85% isolated yield. The formation of 17% rearrangement products is apparent from NMR analysis of the isomeric product mixture.

Table S3. Optimization of the reaction with 3-fluoro-1-phenylbutane and Ph_3Al.

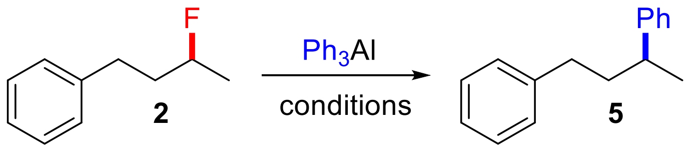

Entry	Solvent	Ph_3Al (Equiv.)	Temperature	Time	Conversion (%)[a]	Elimination (%)	Rearrangement (%)
1	DBE	1.0	25 °C	1 h	100	20	25
2	DBE	1.0	-40 °C	16 h	0	N/A	N/A
3	PhCl	1.0	25 °C	1 h	100	22	42
4	PhCl	1.0	-40 °C	1 h	100	10	41
5	PhCl	2.0	-40 °C	1 h	100	6	39

6	PhCl	0.5	-40 °C	1 h	100	4	45
7	PhCl	1.0	-40 °C	1 h	100	6	44[b]
8	PhCl	1.0	-40 °C	1 h	100	11	43[c]
9	PhCl	1.0	-78 °C	2 h	100	<1	13[d]
10	PhCl	1.0	-78 °C	1 min	50	<1	19[d,e]
11	PhCl	1.0	-78 °C	2 h	69	<1	15[d,e]
12	PhCl	1.0	-78 °C	1 h	100	<1	26[d,f]
13	PhCl	1.0	-50 °C	5 min	90	2	30
14	PhCl	1.0	-50 °C	10 min	90	2	30
15	PhCl/ Pentane (1:1)	0.5	-78 °C	3 h	41	4	20[d]
16	PhCl/ Pentane (1:1)	0.5	-78 °C	5.5 h	46	4	17[d]
17	PhCl/ Pentane (1:1)	0.5	-78 °C	22 h	85	31	19[d,g]
18	PhCl/ Pentane (1:1)	1.0	-78 °C	3 h	72	4	19[d]
19	PhCl	1.5	-78 °C	1 h	100 (89)	3	30[d,h]
20	PhCl/ Pentane (1:2)	1.5	-78 °C	1 h	100 (88)	3	20[h,i]
21	PhCl/ Pentane (1:2)	1.5	-78 °C	30 min	100 (85)	1	17[h]

[a] 0.07 mmol **6** used. Ph_3Al solutions were 1.0 M (DBE) and 359.0 mM (PhCl). Conversions were determined by GC-MS. [b] 1 mL extra solvent added. [c] Alkyl fluoride added dropwise to Ph_3Al. [d] Addition of Ph_3Al solution led to immediate freezing. [e] Quenched while frozen. [f] Ph_3Al solution at -40 °C added to alkyl fluoride dissolved in 200 μL of PhCl at -78 °C, then warmed to 25 °C. [g] Slowly warmed to 25 °C after 7 h. [h] 0.20 mmol **2** used. [i] Slowly warmed to 25 °C immediately after Ph_3Al addition. Parentheses indicate isolated yield.

5. General Procedures for C-C Coupling of Alkyl Fluorides

General Procedure for C-C Coupling of Tertiary Fluorides – Method A

In a nitrogen filled glovebox, 1-fluoroadamantane (46.8 mg, 0.303 mmol) was cooled to -40 °C. Triphenylaluminum (1.0 M in dibutyl ether, 151.0 μL, 0.151 mmol, 0.5 equiv.) was added and the mixture was stirred at -40 °C for 2 hours. The reaction was quenched by addition of methanol (4.0 mL), concentrated, and purified by flash chromatography (100% hexanes) to give a white solid

(91%, 58.6 mg, 0.276 mmol). ^{1}H NMR (400 MHz, chloroform-*d*) δ = 7.43 – 7.31 (m, 4H), 7.21 (m, 1H), 2.13 (m, 3H), 1.96 (d, J = 2.9 Hz, 6H), 1.81 (m, 6H); ^{13}C NMR (100 MHz, chloroform-*d*) δ = 151.3, 128.1, 125.5, 124.8, 43.2, 36.8, 36.2, 29.0. The spectroscopic data of 1-phenyladamantane (**6**) are in accordance with the literature.[2]

General Procedure for C-C Coupling of Secondary Fluorides – Method B

3-Fluoro-1-phenylbutane (30.2 mg, 0.198 mmol) was dissolved in pentane (1.648 mL) and cooled to -78 °C. In a nitrogen filled glovebox, triphenylaluminum (359.0 mM in chlorobenzene, 824.0 µL, 0.295 mmol, 1.5 equiv.) was placed in a syringe with a needle and cooled to -40 °C. This solution was added to the alkyl fluoride solution under nitrogen, while ensuring that the reaction mixture did not freeze. The reaction was kept at -78 °C for 30 minutes and was then quenched by addition of methanol (4.0 mL), concentrated by rotary evaporation, and purified by flash chromatography (100% hexanes) to give a colorless oil (85%, 35.3 mg, 0.168 mmol). ^{1}H NMR (400 MHz, chloroform-*d*) δ = 7.36 – 7.27 (m, 2H), 7.24 – 7.11 (m, 8H), 2.71 (m, 1H), 2.51 (m, 2H), 1.92 (m, 2H), 1.28 (d, J = 6.9 Hz, 3H); ^{13}C NMR (100 MHz, chloroform-*d*) δ = 147.3, 142.5, 128.3, 128.3, 128.2, 127.0, 125.9, 125.6, 39.9, 39.5, 33.9, 22.5. The spectroscopic data of 1,3-diphenylbutane (**5**) are in accordance with the literature.[3]

General Procedure for C-C Coupling of Primary Fluorides – Method C

In a nitrogen filled glovebox, 1-chloro-4-(3-fluoropropyl)benzene (43.1 mg, 0.250 mmol), was cooled to -40 °C. A solution of triphenylaluminum (359.0 mM in chlorobenzene, 1.045 mL, 0.375 mmol, 1.5 equiv.) was cooled to -40 °C, added to the alkyl fluoride and the solution was stirred for 90 minutes. The reaction was quenched by addition of methanol (4.0 mL), concentrated, and

purified by flash chromatography (100% hexanes) to give a colorless oil (80%, 46.0 mg, 0.199 mmol). ^{1}H NMR (400 MHz, chloroform-*d*) δ = 7.33 – 7.23 (m, 4H), 7.22 – 7.16 (m, 3H), 7.12 (m, 2H), 2.68 – 2.58 (m, 4H), 1.95 (m, 2H); ^{13}C NMR (100 MHz, chloroform-*d*) δ = 142.0, 140.6, 131.4, 129.7, 128.4, 128.4, 128.3, 125.8, 35.3, 34.7, 32.8. The spectroscopic data of 1-chloro-4-(3-phenylpropyl)benzene (**53**) are in accordance with the literature.[4]

6. Mechanistic Studies

6.1. Friedel-Crafts Trapping Experiments

To trap carbocationic reaction intermediates in the reaction of triphenylaluminum with primary, secondary and tertiary alkyl fluorides, 1,3-dimethoxybenzene was used as the reaction solvent. In all cases, Friedel-Crafts adducts were obtained as the major product. In the case of adamantyl fluoride, the Friedel-Crafts product **24** was generated in a 7:1 ratio vs the cross-coupling product **6**. The reaction with 3-fluorobutylbenzene gave a 6:1 ratio, and the reaction with *n*-octyl fluoride resulted in a 3:1 ratio. The steadily decrease in relative Friedel-Crafts product formation coincides with decreasing stability of the corresponding cationic intermediates that have shorter lifetimes and are therefore more likely to react with the fluorotriphenylaluminate counteranion compared to the trapping reaction with dimethoxybenzene after ion pair dissociation.

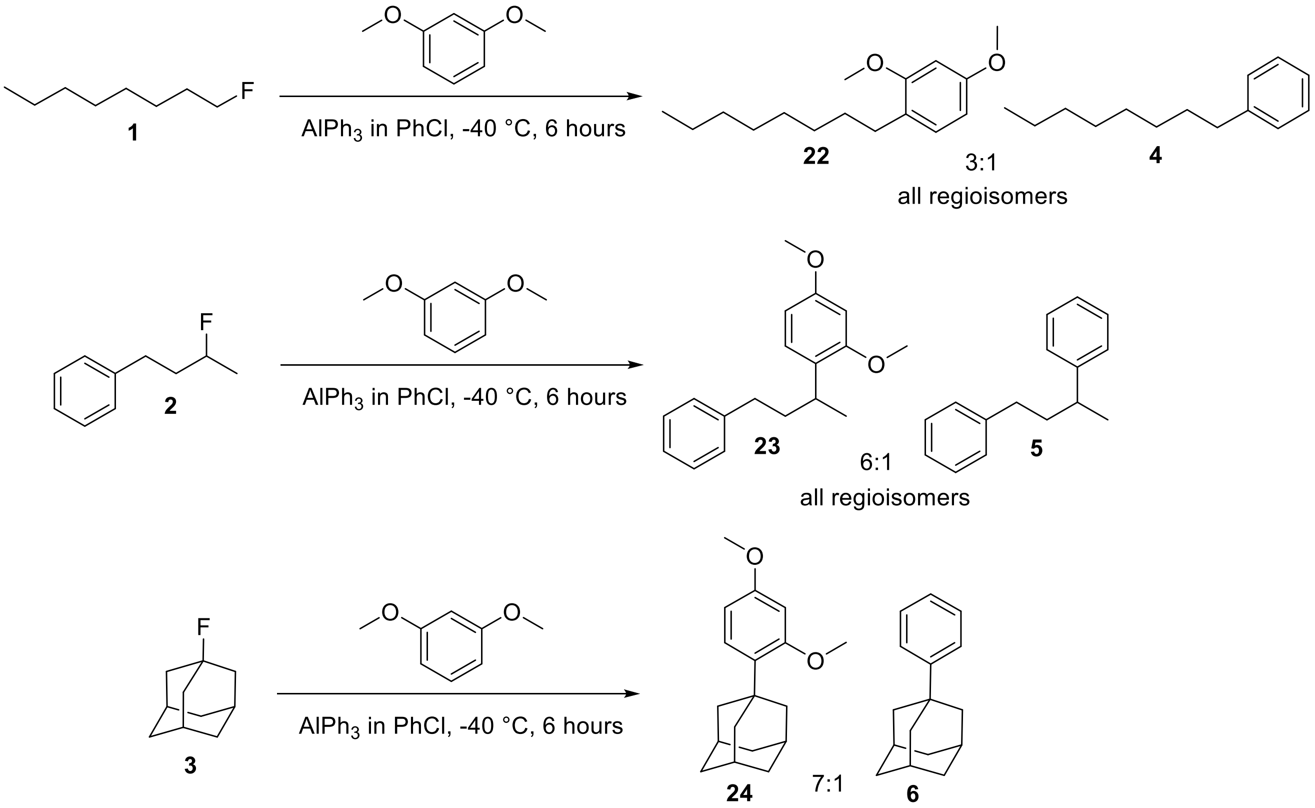

Figure S8. Intermediate cation trapping with 1,3-dimethoxybenzene.

In a nitrogen-filled glove box, the alkyl fluoride (0.033 mmol) was dissolved in 1.0 mL of 1,3-dimethoxybenzene and the resulting solution was chilled to -40 °C. Triphenylaluminum (359.0 mM in chlorobenzene, 91.0 μL, 0.033 mmol, 1 equiv.) was added, and the reaction was stirred for 6 hours at -40 °C. Aliquots were removed, quenched with methanol, and analyzed by GC-MS.

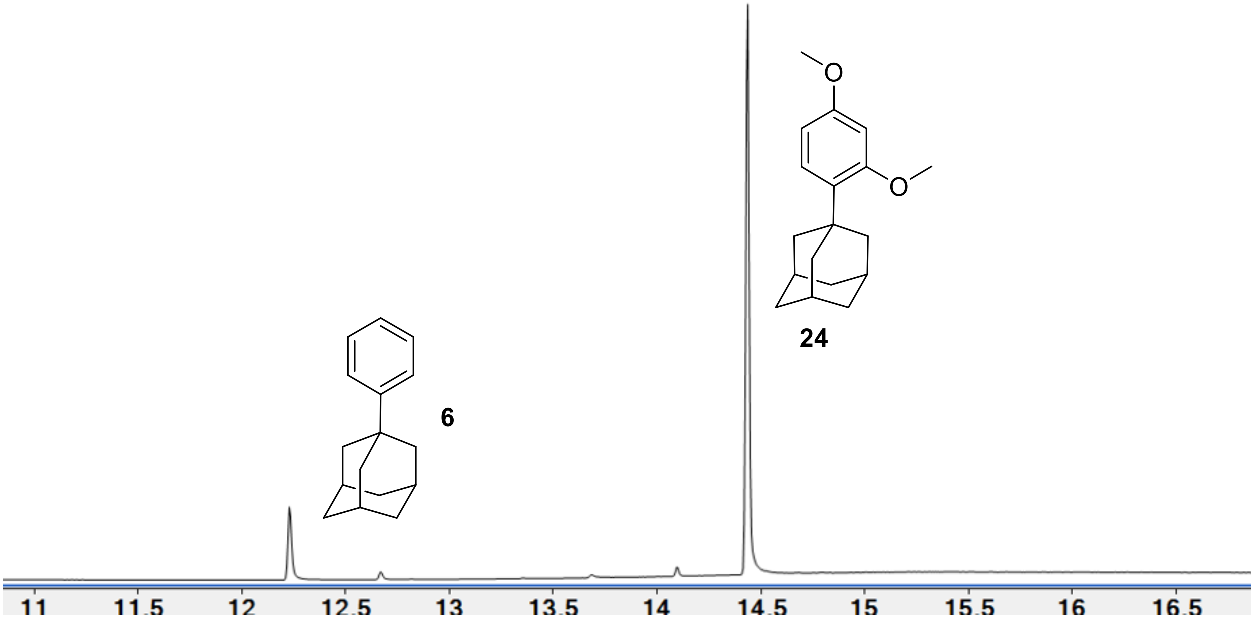

Figure S9. The reaction between triphenylaluminum and 1-fluoroadamantane (**3**) in 1,3-dimethoxybenzene. 1-(2,4-Dimethoxyphenyl)adamantane (**24**) is generated in a 7:1 ratio vs. 1-phenyladamantane (**6**).

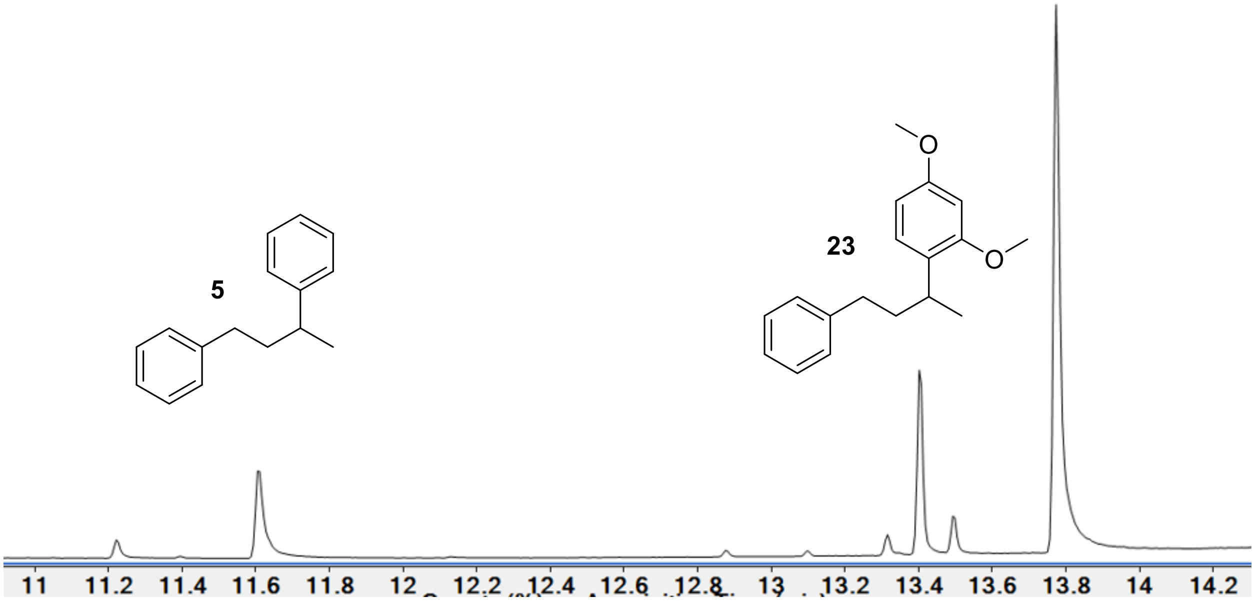

Figure S10. The reaction between triphenylaluminum and (3-fluorobutyl)benzene (**2**) in 1,3-dimethoxybenzene. Multiple isomers of the Friedel-Crafts and phenyl adducts are observed. Isomers of 2,4-dimethoxy-1-(4-phenylbutanyl)benzene (**23**) are generated in a 6:1 ratio vs isomers of diphenylbutane (**5**).

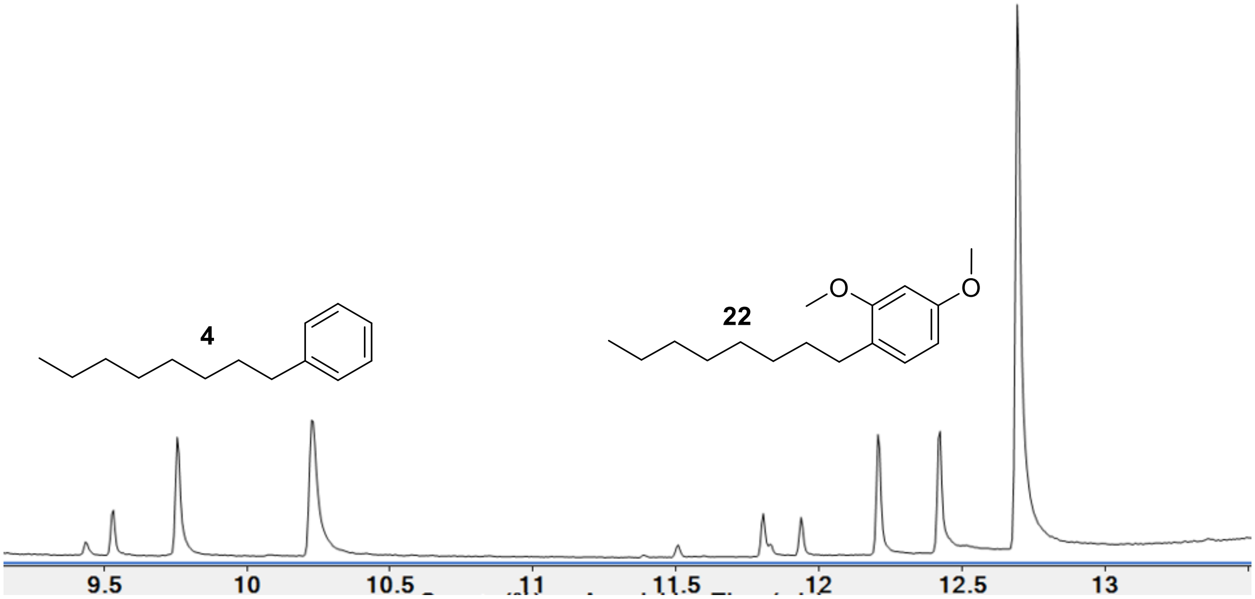

Figure S11. The reaction between triphenylaluminum and 1-fluorooctane (**1**) in 1,3-dimethoxybenzene. Multiple isomers of the Friedel-Crafts and phenyl adducts are observed.

Isomers of 2,4-dimethoxyoctylbenzene (**22**) are generated in a 3:1 ratio vs isomers of phenyloctane (**4**).

Benzyl fluoride trapping experiment

In a nitrogen-filled glove box, 4-bromo-1-(fluoromethyl)-2-methylbenzene (6.7 mg, 0.033 mmol) was dissolved in 1.0 mL of 1,3-dimethoxybenzene and the resulting solution was chilled to -40 °C. Triphenylaluminum (359.0 mM in chlorobenzene, 91.0 µL, 0.033 mmol, 1.0 equiv.) was added and the reaction was stirred for 6 hours at -40 °C. An aliquot was removed, quenched with methanol and analyzed by GC-MS.

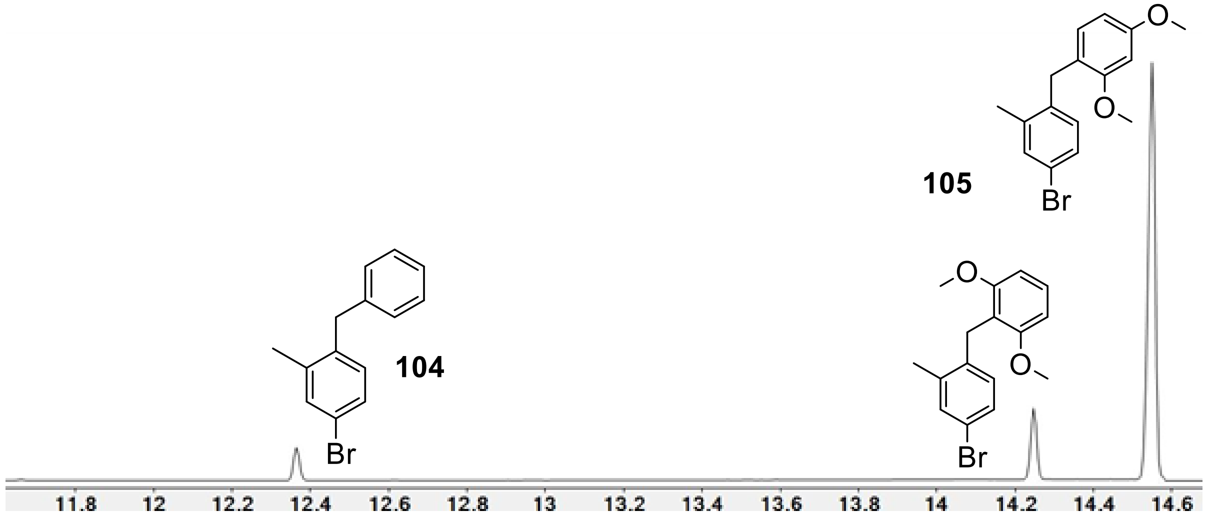

Figure S12. GC-MS analysis of the reaction between triphenylaluminum and 4-bromo-1-(fluoromethyl)-2-methylbenzene (**104**) in 1,3 dimethoxybenzene. Peak integration showed a ratio (Friedel-Crafts products/phenylation product) of 17.5: 1.

6.2. 1°, 2°, 3° Alkyl Fluoride Competition Experiments

We ran competition experiments with equimolar amounts of 1-fluorooctane, fluorocyclohexane and 1-fluoroadamantane using either 0.11 or 0.33 equivalents of triphenylaluminum. 1-Fluoroadamantane proved most reactive followed by fluorocyclohexane and 1-fluorooctane. The observed reactivity trend (tertiary > secondary > primary alkyl fluoride) is in agreement with a stepwise mechanism proceeding through a carbocation aluminate intermediate. The results also indicate that the C-F bond cleavage is the rate-determining step.

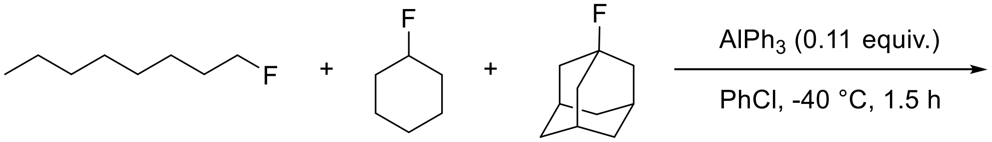

In a nitrogen-filled glove box, 1-fluorooctane (5.0 mg, 0.038 mmol), fluorocyclohexane (3.86 mg, 0.038 mmol) and 1-fluoroadamantane (5.83 mg, 0.038mmol) were combined in a vial and chilled to -40 °C. An aliquot was taken for GC-MS analysis prior to $AlPh_3$ addition. Triphenylaluminum (359.0 mM in chlorobenzene, 0.013 mmol, 0.11 equiv.) was precooled to the same temperature and added. The resulting solution was allowed to stir at -40 °C for 90 minutes. The reaction mixture was quenched with *tert*-butyl alcohol and an aliquot was taken for GC-MS analysis.

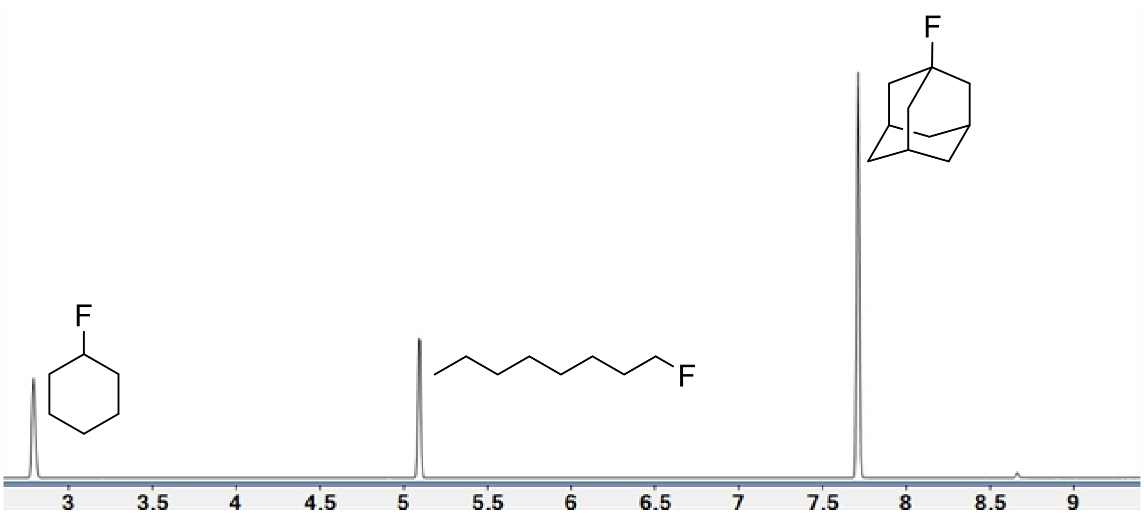

Figure S13. GC-MS analysis of the mixture prior to $AlPh_3$ addition.

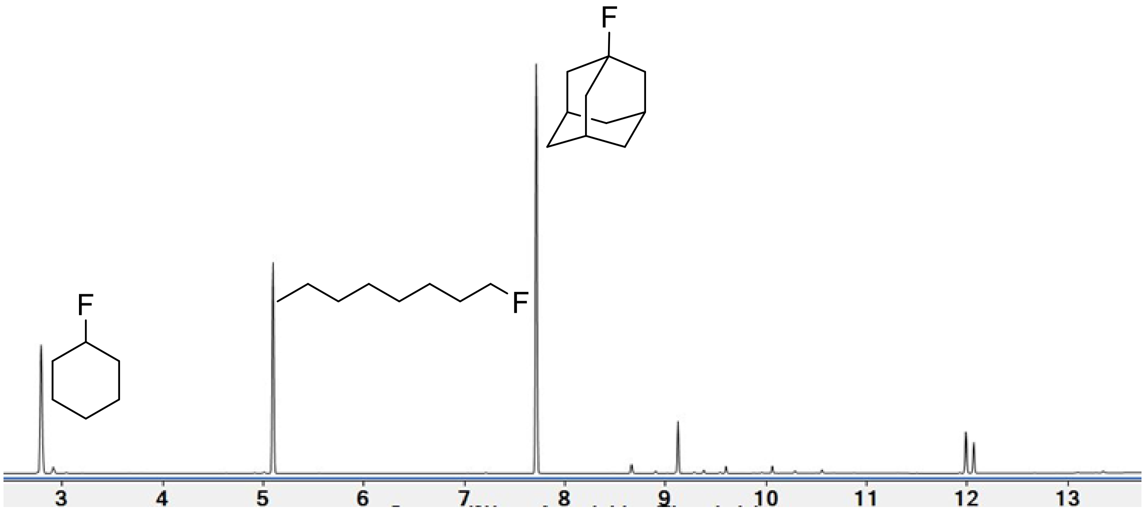

Figure S14. GC-MS analysis of the competition experiment after 90 minutes. Peak integration showed 2.7% consumption of 1-fluorooctane, 5.9% consumption of fluorocyclohexane, and 8.4% consumption of 1-fluoroadamantane.

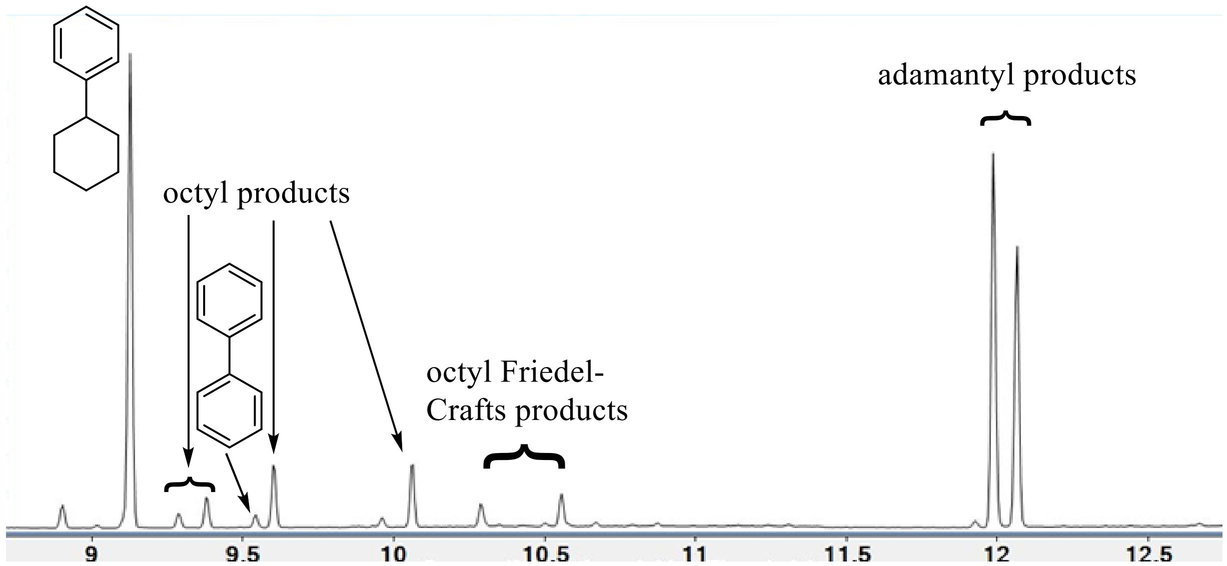

Figure S15. GC-MS analysis of the competition experiment after 90 minutes.

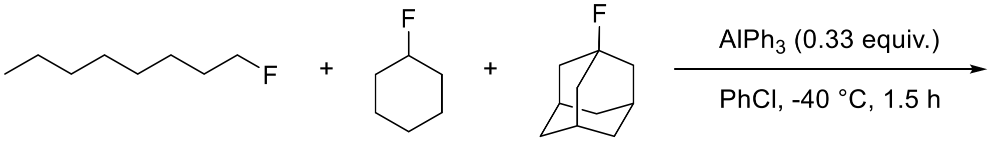

In a nitrogen-filled glove box, 1-fluorooctane (5.0 mg, 0.038 mmol), fluorocyclohexane (3.86 mg, 0.038 mmol) and 1-fluoroadamantane (5.83 mg, 0.038mmol) were combined in a vial and chilled to -40 °C. An aliquot was taken for GC-MS analysis prior to $AlPh_3$ addition. Triphenylaluminum (359.0 mM in chlorobenzene, 0.038 mmol, 0.33 equiv.) was precooled to the same temperature and added. The resulting solution was allowed to stir at -40 °C for 90 minutes. The reaction mixture was quenched with *tert*-butyl alcohol and an aliquot was taken for GC-MS analysis.

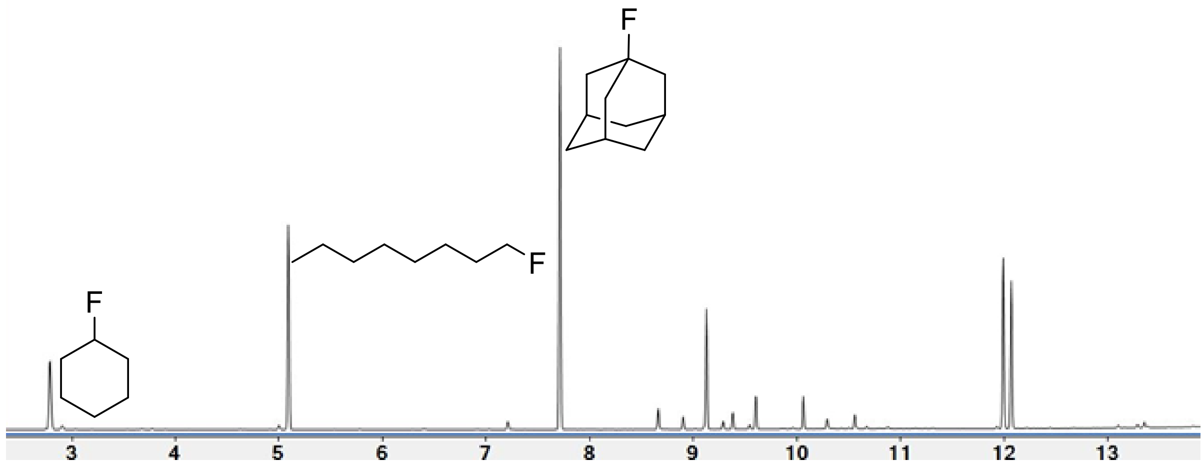

Figure S16. GC-MS analysis of the competition experiment after 90 minutes. Peak integration showed 8.8% consumption of 1-fluorooctane, 9.0% consumption of fluorocyclohexane, and 26.2% consumption of 1-fluoroadamantane.

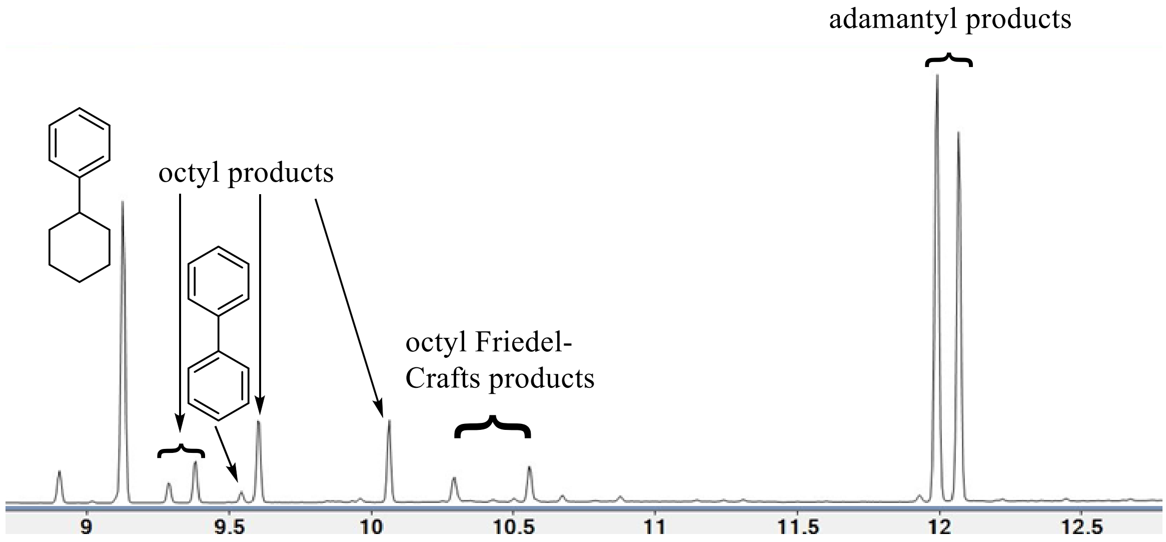

Figure S17. GC-MS analysis of the competition experiment after 90 minutes.

6.3. General Procedure for AlX_3 Reactions

The reactions of 1-fluorooctane with aluminum trichloride, tribromide and triiodide, respectively, were monitored by GC-MS analysis. This set of experiments were then repeated with 3-fluoro-1-phenylbutane.

a) Reactions with 1-fluorooctane

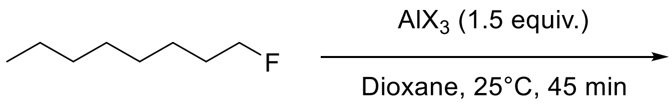

Under the same conditions, the halodefluorination was the fastest with AlI_3 and significantly slower when $AlCl_3$ was used, which follows the Al Lewis acidity trends (AlI_3>$AlBr_3$>$AlCl_3$). Negligible amounts of isomerization products were observed in all cases but there were some by-products from a reaction with the dioxane solvent. The negligible isomerization observed is consistent with a concerted mechanism or relatively short lifetimes of intermediate ion pairs.

In a nitrogen-filled glove box, 1-fluorooctane (5.0 mg, 0.038 mmol) was added to a solution of $AlCl_3$ (7.5 mg, 0.056 mmol, 1.5 equiv.) in 1,4-dioxane (150 μL) and the resulting solution was allowed to stir at 25 °C for 45 minutes. An aliquot was taken and quenched with *tert*-butyl alcohol for GC-MS analysis. The same procedure was repeated with $AlBr_3$ and AlI_3.

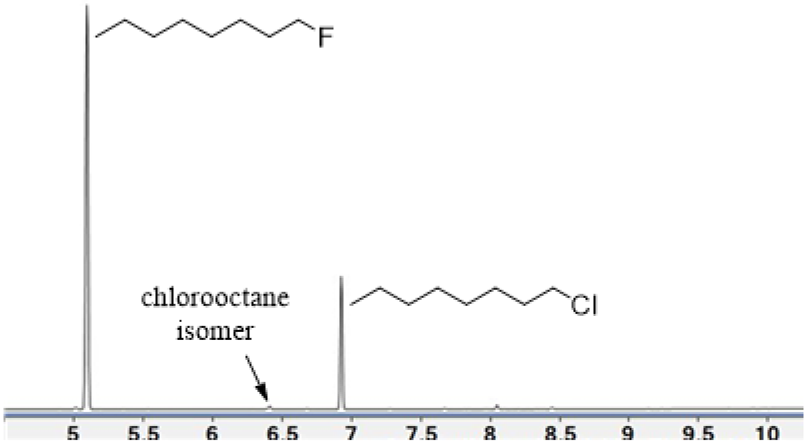

Figure S18. GC-MS analysis of the reaction between $AlCl_3$ and 1-fluorooctane. Peak integration gave a ratio (1-fluorooctane/1-chlorooctane) of 3.7:1.

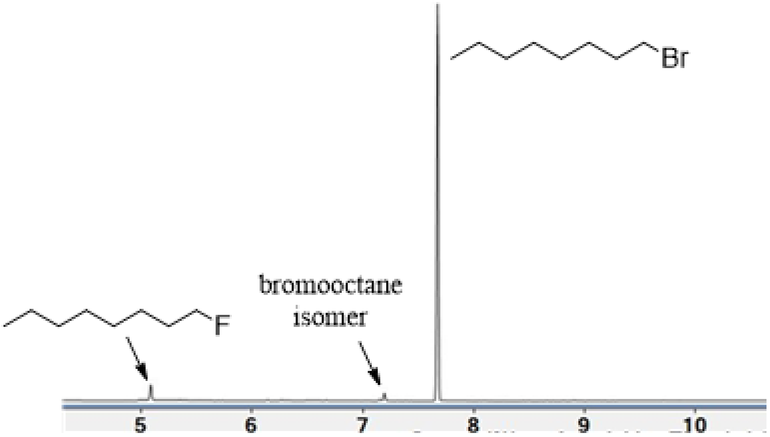

Figure S19. GC-MS analysis of the reaction between $AlBr_3$ and 1-fluorooctane. Peak integration gave a ratio (1-fluorooctane/1-bromoctane) of 1:16

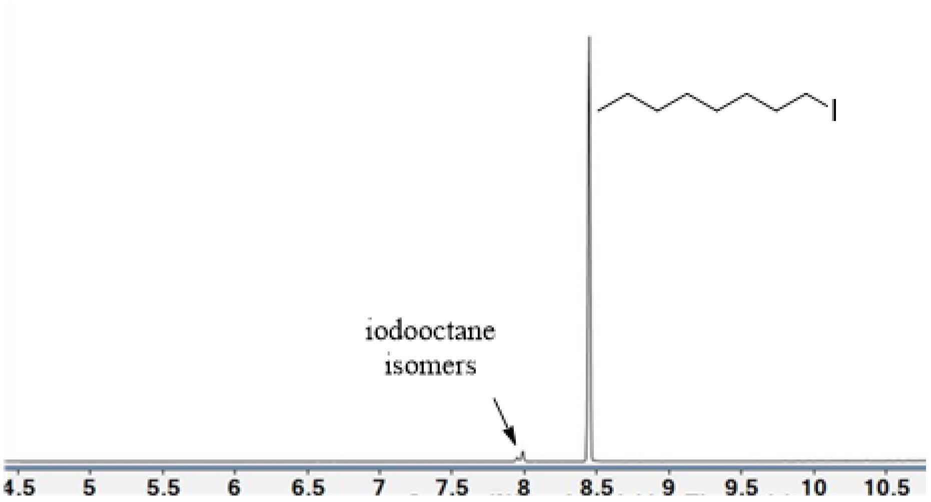

Figure S20. GC-MS analysis of the reaction between AlI_3 and 1-fluorooctane. 1-Fluorooctane was quantitatively consumed.

b) Reactions with 3-fluoro-1-phenylbutane

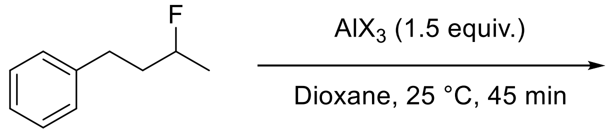

The secondary alkyl fluoride was fully consumed in all cases. The increased reactivity compared to the less sterically hindered 1-fluorooctane (which did not fully react with $AlCl_3$ and $AlBr_3$ under the same conditions) points toward a predominant ionic mechanism as larger amounts of remaining starting materials would have been expected for a concerted pathway with the sterically more hindered 3-fluoro-1-phenylbutane. We were able to detect isomerization products in all three reactions but in increasing amounts with $AlCl_3$ while AlI_3 gave significantly less isomerization. This trend follows the increasing nucleophilicity of the halide (iodide>bromide>chloride) which is in agreement with a stepwise mechanism in which the lifetime of the intermediate ion pairs would decrease in the opposite order ($AlCl_3$>$AlBr_3$>AlI_3), i.e. the iodide is expected to be

transferred significantly faster than the other halides. This would give less time for the competing isomerization of the intermediate carbocation. Elimination side reactions were found to follow the same trend ($AlCl_3$>$AlBr_3$>AlI_3).

In a nitrogen-filled glove box, 3-fluoro-1-phenylbutane (6.2 mg, 0.041 mmol) was added to a solution of $AlCl_3$ (8.13 mg, 0.061 mmol, 1.5 equiv.) in 1,4-dioxane (150 µL), and the resulting solution was allowed to stir at 25 °C for 45 minutes. An aliquot was taken and quenched with *tert*-butyl alcohol for GC-MS analysis. The same procedure was repeated with $AlBr_3$ and AlI_3.

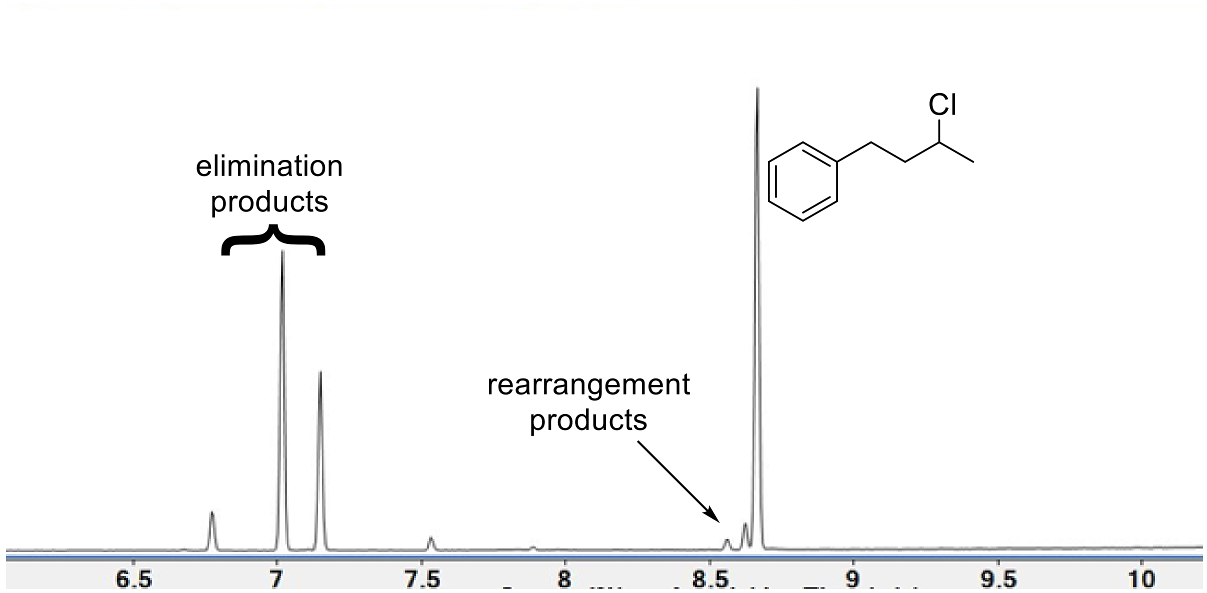

Figure S21. GC-MS analysis of the reaction between $AlCl_3$ and 3-fluoro-1-phenylbutane. Peak integration gave a ratio (3-chloro-1-phenylbutane/rearrangement products) of 12:1. The ratio between the chlorinated compounds and elimination products was determined as 0.97.

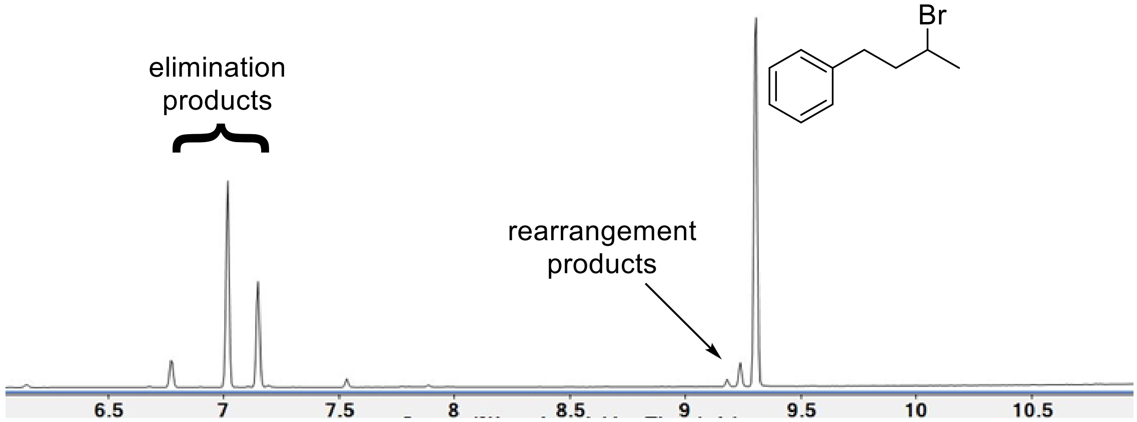

Figure S22. GC-MS analysis of the reaction between $AlBr_3$ and 3-fluoro-1-phenylbutane. Peak integration gave a ratio (3-bromo-1-phenylbutane/rearrangement products) of 13:1. The ratio between the brominated compounds and elimination products was determined as 1.22: 1.

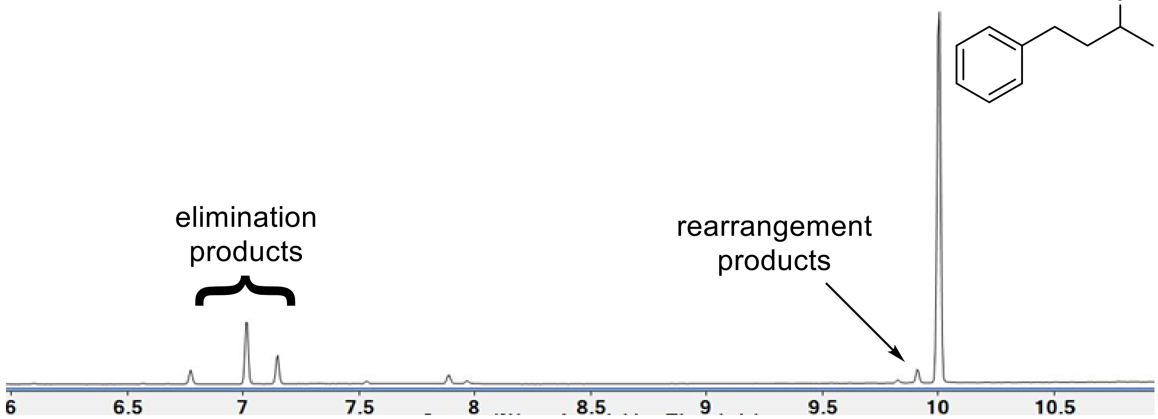

Figure S23. GC-MS analysis of the reaction between AlI_3 and 3-fluoro-1-phenylbutane. Peak integration gave a ratio (3-iodo-1-phenylbutane/rearrangement products) of 27:1. The ratio between the iodinated compounds and elimination products was determined as 3.8.

6.4.X-ray Characterization of a Carbocationic Intermediate

Given the smooth C-F functionalization by triarylaluminum reagents under cryogenic conditions, isolation of ionic reaction intermediates was not feasible. We therefore decided to investigate the reaction by employing aluminum triflate as the fluoride scavenger. This non-nucleophillic Lewis acid reacts with tri(4-tolyl)fluoromethane to form the isolable ion pair (**27**). The ion pair was characterized by X-ray crystallography.

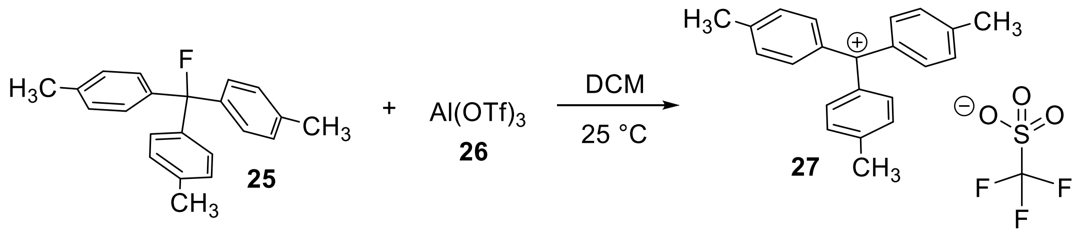

Figure S24. The reaction of tri(4-tolyl)fluoromethane and aluminum triflate in dichloromethane.

In a nitrogen-filled glove box, tri(4-tolyl)fluoromethane (76.0 mg, 0.25 mmol) was dissolved in 1.0 mL of dichloromethane. Aluminum triflate (119.0 mg, 0.25 mmol) was added, and the solution was allowed to stir at 25 °C for 1 hour. The resulting bright yellow solution was filtered to ensure homogeneity and the filtrate was layered with one volume of pentane. The resulting mixture was allowed to stand at -40 °C overnight, affording yellow crystals suitable for X-ray diffraction analysis.

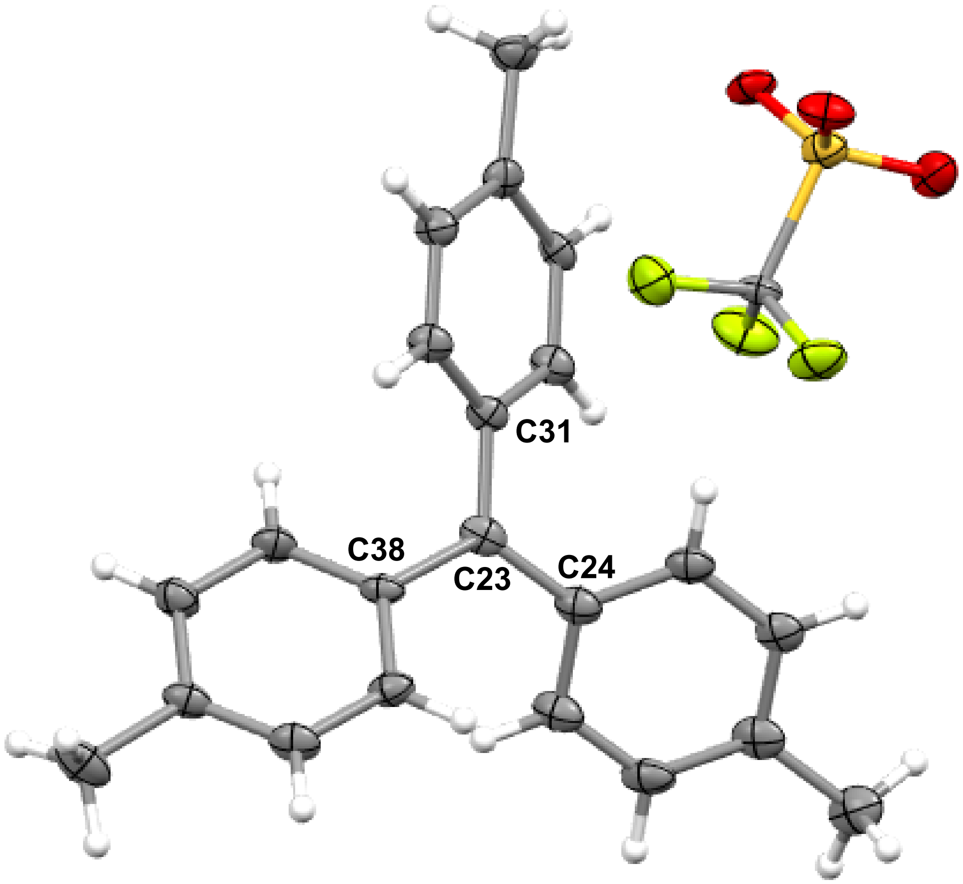

Figure S25. X-ray structure of ion pair **27**. Thermal ellipsoids are displayed at the 50% probability level. One out of two crystallographically distinct ion pairs are shown for clarity. Select bond lengths and angles: C23-C24 1.452(14) Å, C23-C31 1.461(15) Å, C23-C38 1.464(14) Å, C24-C23-C31 120.8°(9), C24-C23-C38 119.6°(9), C31-C23-C38 119.6°(9). Single crystals suitable for X-ray diffraction analysis were grown from a saturated dichloromethane solution layered with one volume of pentane at -40 °C.

6.5. Studies with the Radical Scavengers TEMPO and 9,10-Dihydroanthracene

To probe the possibility of a radical pathway, 1-fluoroadamantane (**3**) was treated with triphenylaluminum in the presence of TEMPO (**32**), a known radical scavenger. When treated with one equivalent of TEMPO, the reaction proceeds smoothly, giving 1-phenyladamantane (**6**) as the major product. This result is consistent with a prevailing ionic reaction mechanism. Surprisingly, ten equivalents of TEMPO afford the adamantyl TEMPO adduct (**33**) as the major product which was identified by GC-MS and X-ray analysis. Indeed, the C-O bond formation also proceeds via an ionic route. It is known that TEMPO disproportionation is energetically favored in the presence of aluminum Lewis acids.[5]

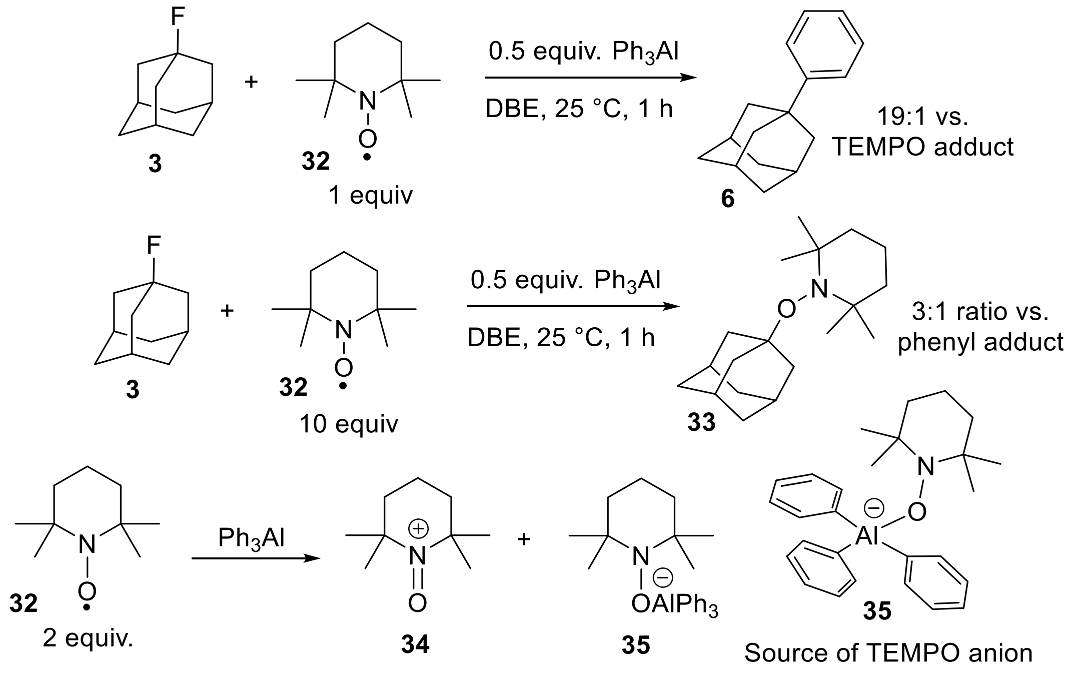

Figure S26. Top: Reaction of 1-fluoroadamantane and triphenylaluminum in the presence of TEMPO. Bottom: Proposed mechanism of the TEMPOate anion formation via aluminum facilitated disproportionation.

In a nitrogen-filled glove box, 1-fluoroadamantane (10.0 mg, 0.065 mmol) and TEMPO (10.1 mg, 0.065 mmol, 1 equiv. or 101 mg, 0.650 mmol, 10 equiv.) were treated with triphenylaluminum (1.0 M in DBE, 33.0 μL, 0.5 equiv.). With 10 equiv. TEMPO, additional 300 μL of DBE were added to improve solubility. The reactions were allowed to stir at 25 °C for 1 hour. Major products were determined by GC-MS.

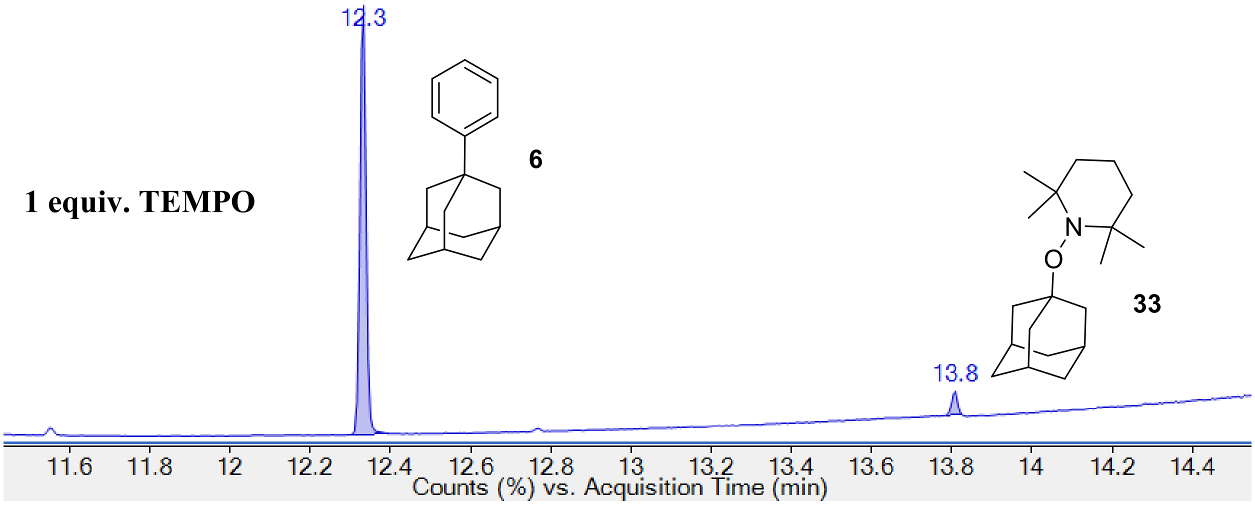

Figure S27. GC analysis of the reaction between 1-fluoroadamantane (**3**) and triphenylaluminum in the presence of one equivalent of TEMPO. 1-Phenyladamantane (**6**) and the adamantyl TEMPO adduct (**33**) are present in a 19:1 ratio.

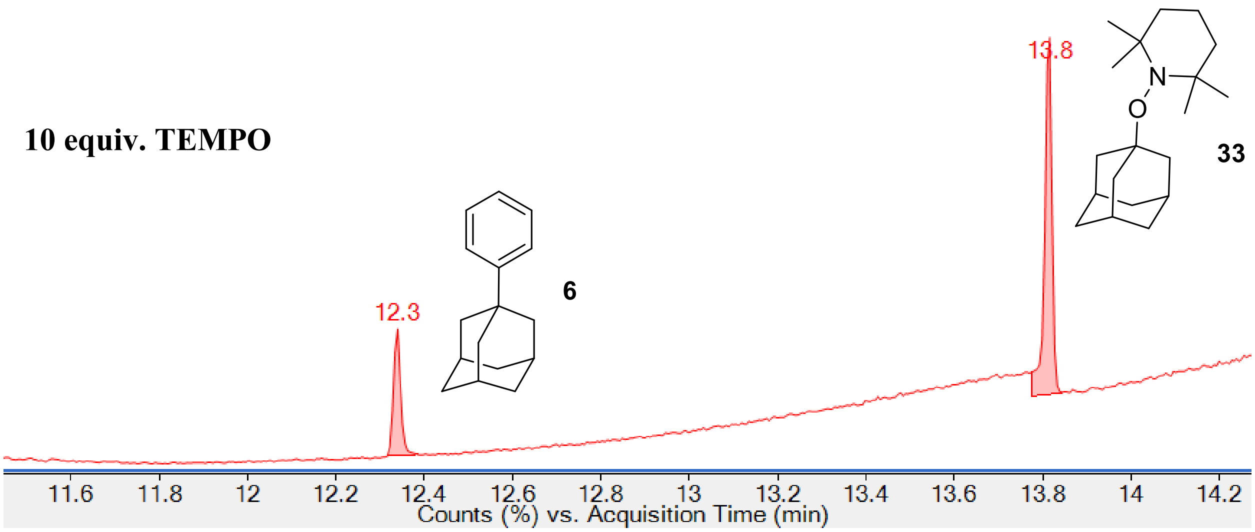

Figure S28. GC analysis of the reaction between 1-fluoroadamantane (**3**) and triphenylaluminum in the presence of ten equivalents of TEMPO. 1-Phenyladamantane (**6**) and the adamantyl TEMPO adduct (**33**) are present in a 1:3 ratio.

The TEMPO adduct was isolated and subjected to single crystal growth. We were pleased to find that single crystals suitable for X-ray diffraction were obtained by evaporation of a saturated pentane solution.

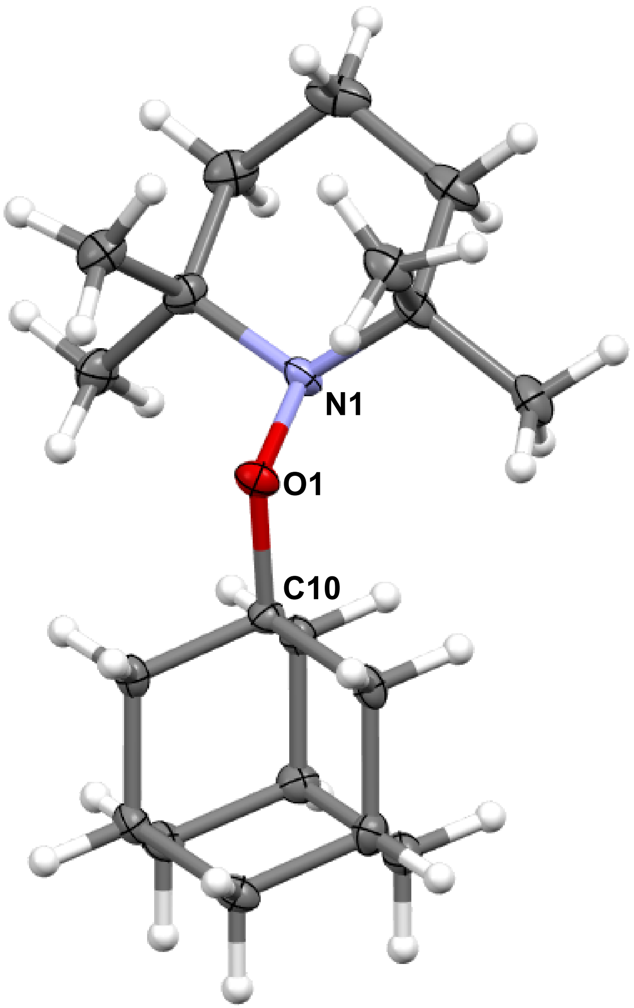

Figure S29. X-ray structure of compound **33**. Thermal ellipsoids are displayed at the 50% probability level. Select bond lengths and angles: C10-O1 1.469(2) Å, O1-N1 1.464(2) Å, N1-O1-C10 118.13°(15). Single crystals suitable for X-ray diffraction were grown from evaporation of a saturated pentane solution.

Reaction with 9,10-Dihydroanthracene

To rule out the incidence of a radical pathway, 1-fluoroadamantane (**3**) was treated with triphenylaluminum in the presence of five equivalents of 9,10-dihydroanthracene (**36**). In a nitrogen-filled glovebox, 1-fluoroadamantane (10.0 mg, 0.065 mmol) and 9,10-dihydroanthracene (58.0 mg, 0.325 mmol) were treated with triphenylaluminum (1.0 M in DBE, 33.0 μL, 0.5 equiv.). The reaction was allowed to stir at 25 °C for one hour and was monitored by GC-MS. Given its very weak C-H bonds (77 kcal/mol) the reaction of 9,10-dihydroanthracene with an alkyl radical is expected to rapidly form anthracene (**37**). By contrast, we found no evidence of anthracene formation, and 1-phenyladamantane (**6**) was generated quantitatively.

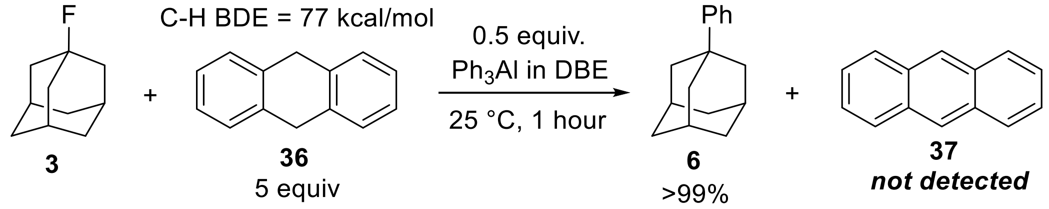

Figure S30. Reaction between 1-fluoroadamantane and triphenylaluminum in the presence of five equivalents of 9,10-dihydroanthracene.

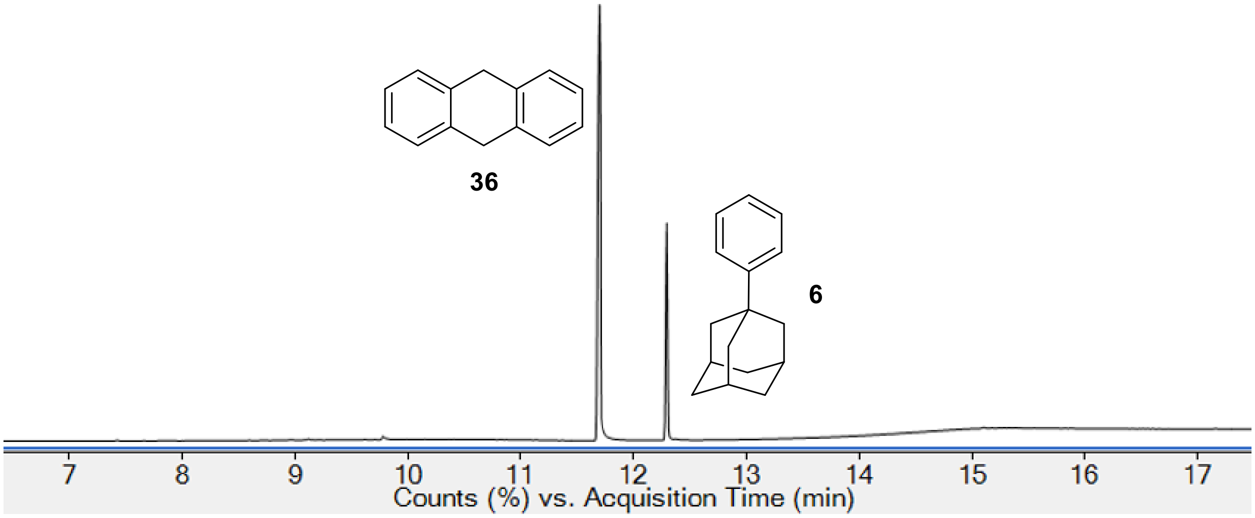

Figure S31. GC analysis of the reaction between 1-fluoroadamantane (**3**) and triphenylaluminum in the presence of five equivalents of 9,10-dihydroanthracene (**36**). The reaction gives complete conversion to 1-phenyladamantane (**6**) and anthracene (**37**) was not detected.

6.6. Stereochemical Analysis of the Reaction with (*S*)-(3-Fluorobutyl)benzene

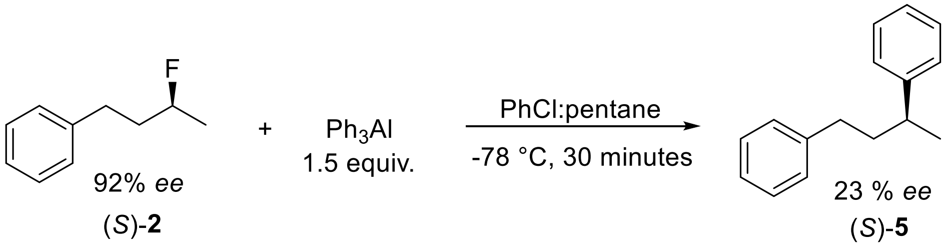

Figure S32. Phenylation of (*S*)-(3-fluorobutyl)benzene.

(*S*)-(3-Fluorobutyl)benzene was prepared according to literature procedure from (*R*)-4-phenylbutan-2-ol.[6] The *ee* was determined by chiral HPLC (CHIRALCEL OJ-H, mobile phase: n-hexanes = 100%, flow rate = 1.0 mL/min, UV detection at 214 nm), t_R = 9.9 min (major), t_R = 9.1 min (minor).

In a nitrogen filled glove box, (*S*)-(3-fluorobutyl)benzene (5.0 mg, 0.033 mmol, 92% *ee*) was dissolved in pentane (274 μL) and cooled to -78 °C. Triphenylaluminum (359.0 mM in chlorobenzene, 137.0 μL, 0.049 mmol, 1.5 equiv.) was placed in a syringe with a needle and cooled to -40 °C. This solution was added to the alkyl fluoride solution under nitrogen. The resulting mixture was stirred for 30 minutes, quenched with methanol, and an aliquot was taken for GC-MS analysis. The *ee* was determined by GC-MS (2,6-dimethyl-3-pentyl-γ-cyclodextrin, 120 °C) as 23%, t_R (major) = 5.7 min, t_R (minor) = 5.2 min. The absolute configuration of the major enantiomer was assigned as *S* via polarimetry according to the literature.[7]

The large reduction in *ee* observed in this experiment points to a carbocationic intermediate that is arylated prior to complete racemization.

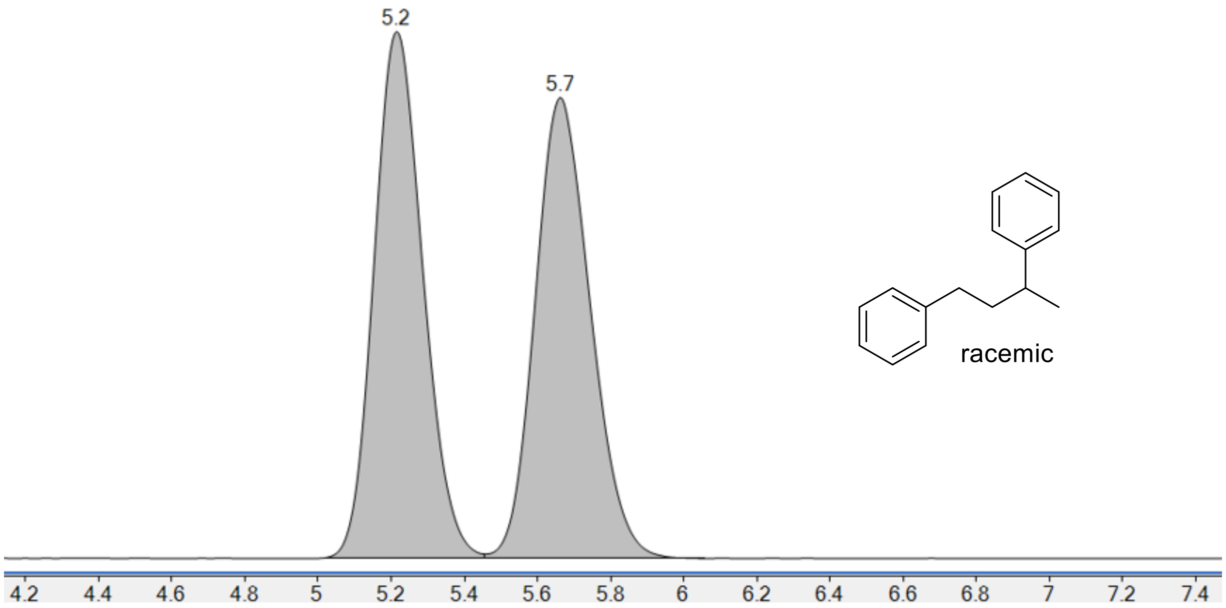

Figure S33. GC-MS Chromatogram of racemic 1,3-diphenylbutane.

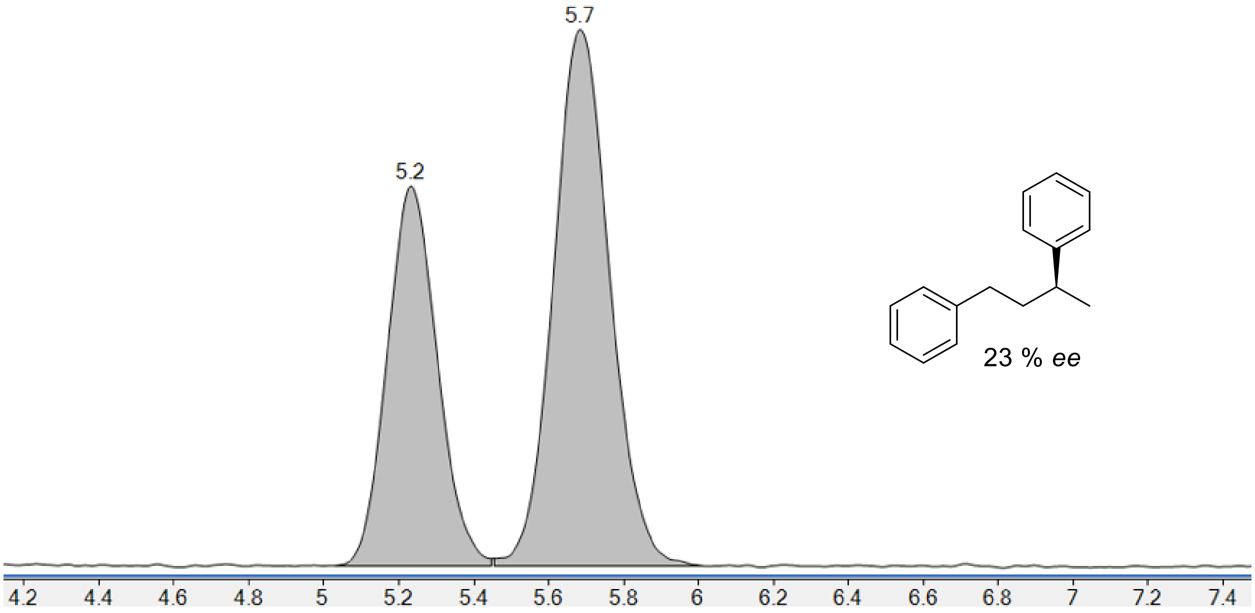

Figure S34. GC-MS Chromatogram of 1,3-diphenylbutane obtained from (*S*)-(3-fluorobutyl)benzene.

6.7. Synthesis of the Cesium-bridged Triphenylfluoroaluminate Dimer 28 and Electrophile Arylation

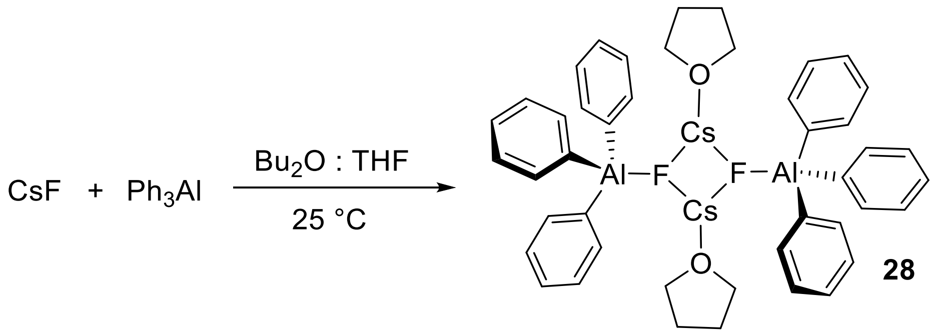

Figure S35. Synthesis of **28**.

Cesium fluoride (20.0 mg, 0.131 mmol) was added to a solution of triphenylaluminum (1.0 M in dibutylether, 131 μL, 0.131 mmol, 1.0 equiv.). The mixture was allowed to stir for 2 hours at room temperature. THF (200.0 μL) was added, and the mixture was stirred for an additional 10 minutes. The resulting solution was filtered, layered with one volume of pentane, and allowed to stand at -40 °C affording single crystals of compound **28** that were suitable for X-ray diffraction. The Al-C bonds in this activated aluminate are 1.991(5) Å, 1.994(5) Å, and 2.001(5) Å in length, while the non-bridged Al-C bonds in neutral triphenylaluminum are 1.979(3) Å and 1.972(2) Å (CCDC 1841031). The elongation of the Al-C bonds is indicative of enhanced nucleophilicity, which is further evidenced in the reaction with the tritylium tetrafluoroborate electrophile below.

X-Ray structure of compound 28.

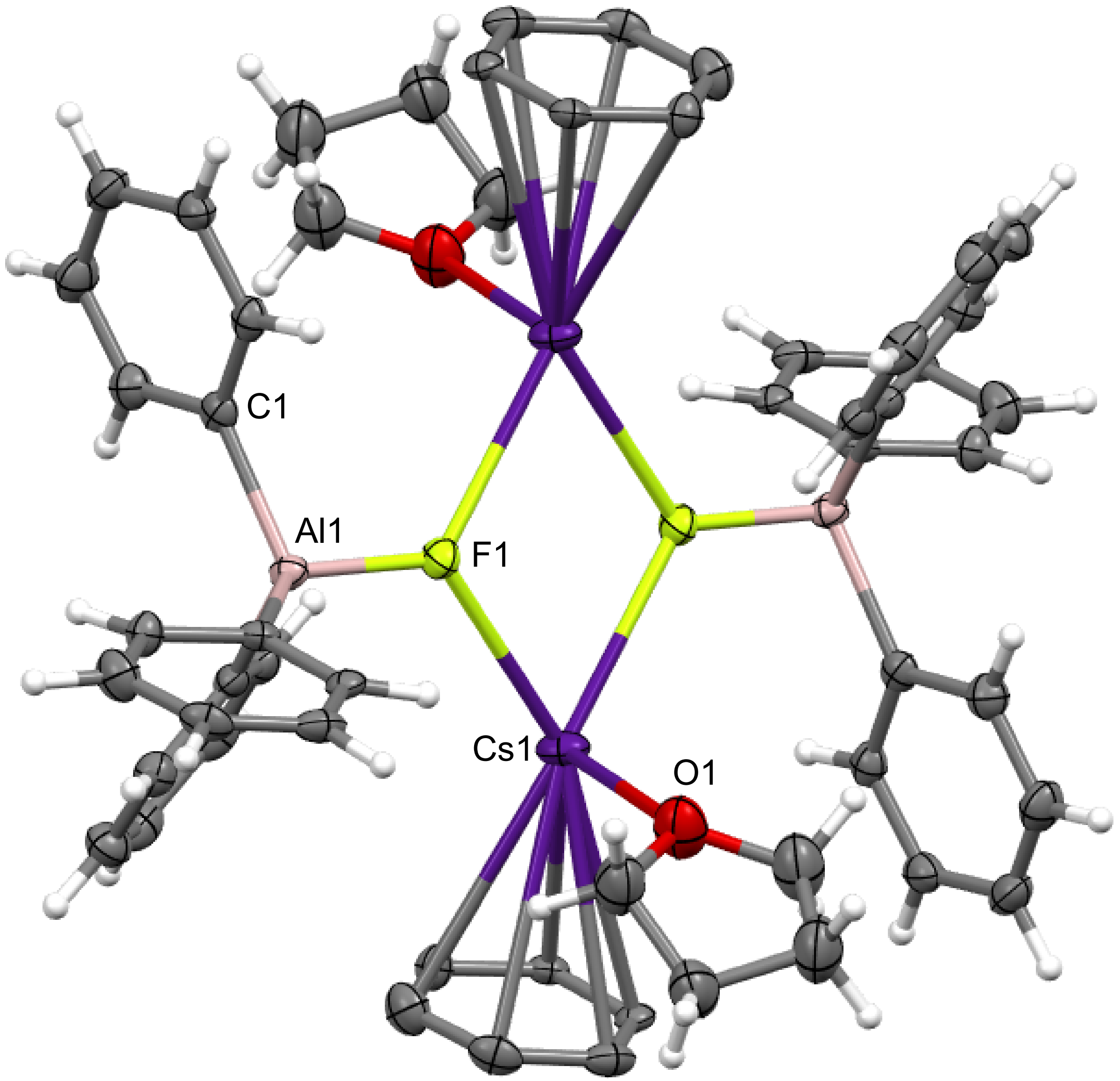

Figure S36. Dimeric X-ray structure of compound 28. Thermal ellipsoids are displayed at the 50% probability level. Cesium atoms are coordinated η-6 to adjacent triphenylaluminum groups. The THF molecule is disordered and is shown in the orientation of highest occupancy for clarity. Select bond lengths and angles: Cs1-F1 2.922(3) Å, Al1-F1 1.739(3) Å, Al1-C1 1.991(5) Å, Al1-C13 1.994(5) Å, Al1-C7 2.001(5) Å, F1-Al1-C1 107.80°(17), Cs1-F1-Cs1 106.14°(8). Single crystals were grown from THF/Bu_2O solution layered with one volume of pentane at -40 °C.

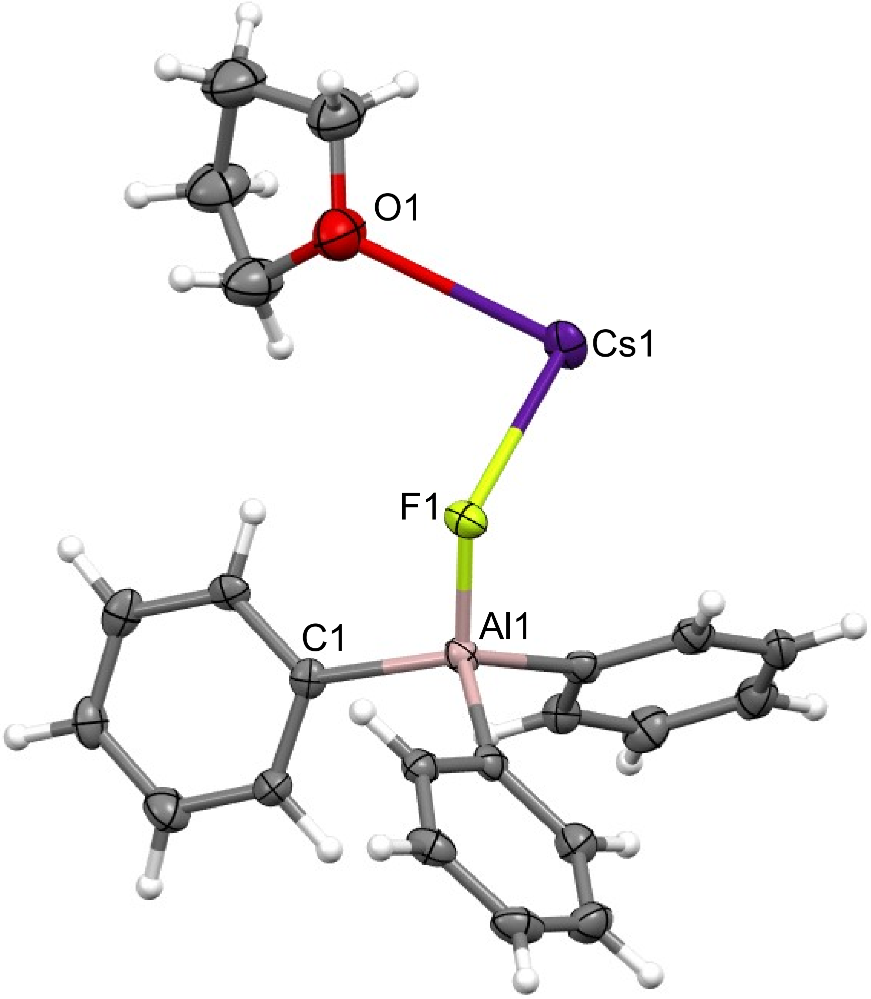

Figure S37. Asymmetric unit of compound 28. Thermal ellipsoids are displayed at the 50% probability level. The disordered THF molecule is shown in the orientation of highest occupancy for clarity. Select bond lengths and angles: Cs1-F1 2.922(3) Å, Al1-F1 1.739(3) Å, Al1-C1 1.991(5) Å, Al1-C13 1.994(5) Å, Al1-C7 2.001(5) Å, F1-Al1-C1 107.80°(17). Single crystals were grown from THF/Bu_2O solution layered with one volume of pentane at -40 °C.

Electrophile Arylation

In order to assess the reactivity of the activated aluminate (**28**) vs neutral triphenyl aluminum, each compound was treated with trityllium tetrafluoroborate. Triphenylaluminum arylates poorly, giving tetraphenylmethane in only 1:12 ratio vs hydrodefluorination. The activated aluminate (**28**) arylates more successfully, resulting in a 1:2 ratio vs hydrodefluorination.

In a nitrogen-filled glovebox, the aluminum reagent (0.061 mmol) was dissolved in 1:1 THF:DCM (1.0 mL). Trityllium tetrafluoroborate (0.061 mmol) was added, and the resulting solution was stirred for 2 hours at 25 °C. The reaction was monitored by GC-MS.

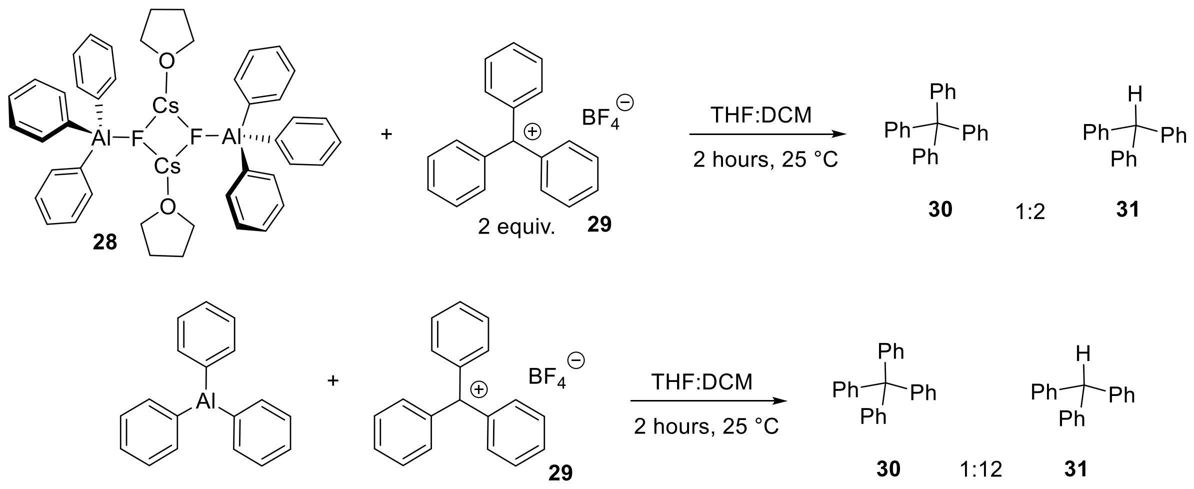

Figure S38. Arylation of trityllium tetrafluoroborate.

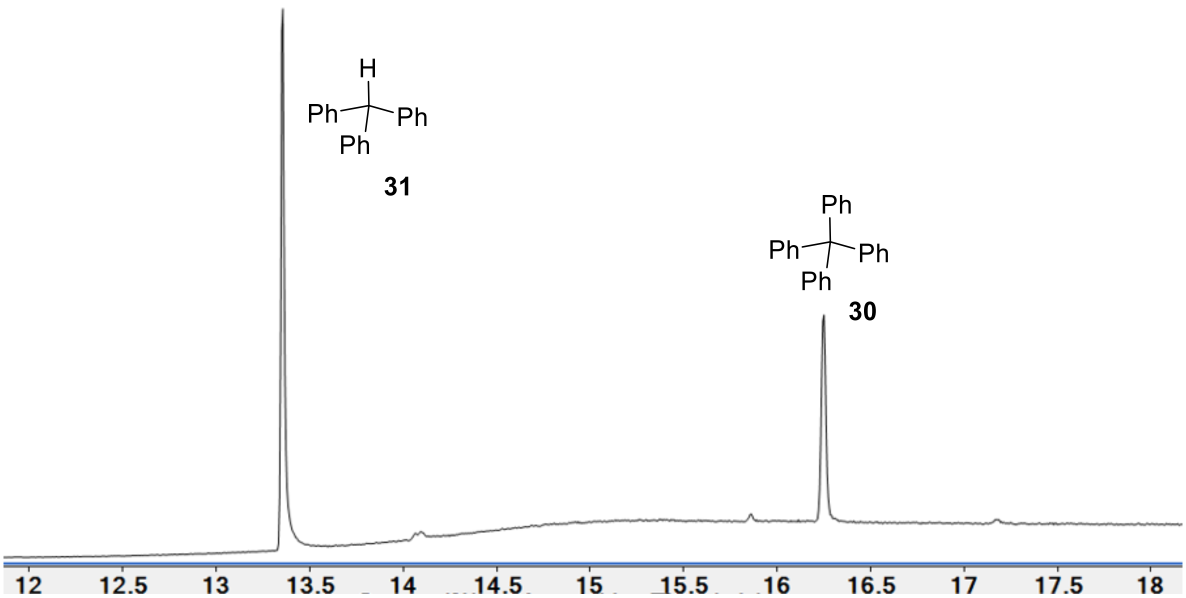

Figure S39. GC-MS analysis of the reaction between aluminate (**28**) and trityllium tetrafluoroborate (**29**).

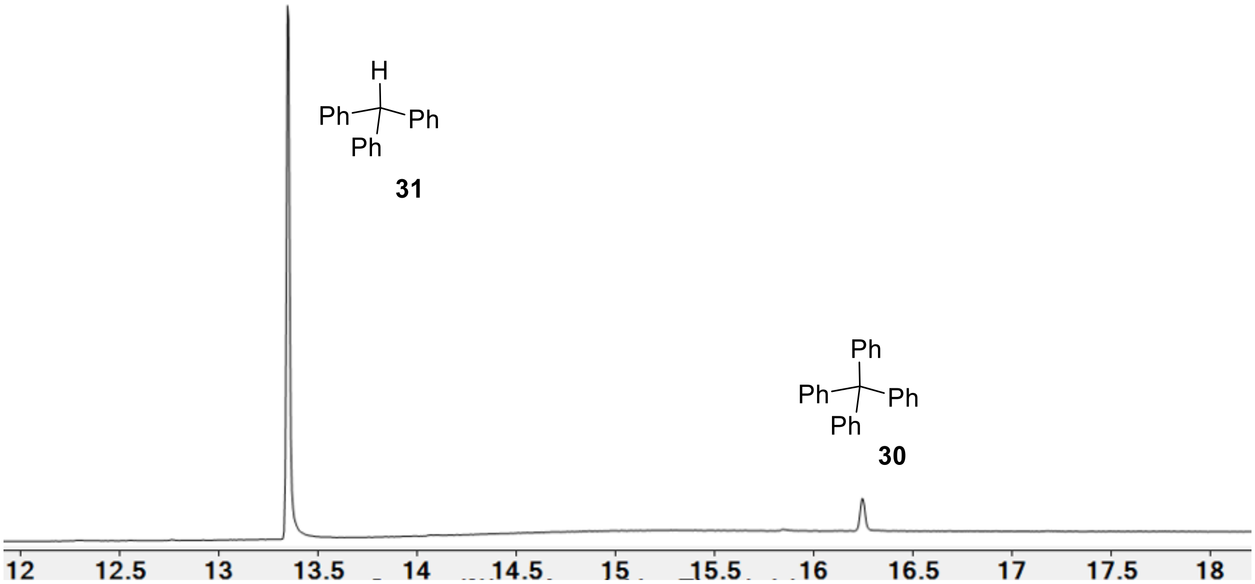

Figure S40. GC-MS analysis of the reaction between triphenylaluminum and trityllium tetrafluoroborate (**29**).

7. Alkyl Fluoride Selectivity Studies

Reactions of 1-Bromo-5-fluoropentane (7) with Ph_2Zn versus Ph_3Al

To demonstrate the high selectivity of triphenylaluminum for C-F bond functionalization, we treated 1-bromo-5-fluoropentane with diphenylzinc. In a 10 mL pressure vessel, 1-bromo-5-fluoropentane (10.0 mg, 0.06 mmol) and diphenylzinc (19.0 mg, 0.09 mmol) were combined in 50.0 μL of $PhCF_3$. The vessel was sealed and the reaction was allowed to stir at 100 °C for 18 hours. The reaction was monitored by GC-MS. Diphenylzinc demonstrates a strong preference for the alkyl bromide bond, giving (5-fluoropentyl)benzene as the major product. 1,5-Diphenylpentane was generated as a minor product, making up less than 10% of the product mixture. In contrast to the zinc chemistry, triphenylaluminum affords (5-bromopentyl)benzene in excellent yield. Not only is the fluoride selectivity outstanding, but the desired product is generated under far milder conditions.

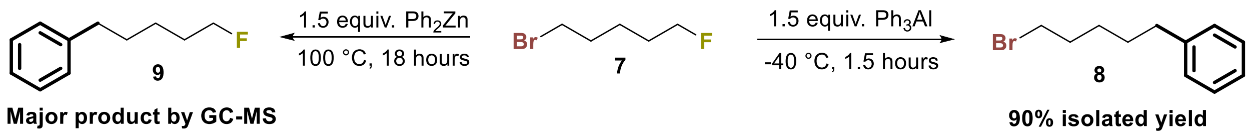

Figure S41. Reaction of 1-bromo-5-fluoropentane with diphenylzinc and with triphenylaluminum.

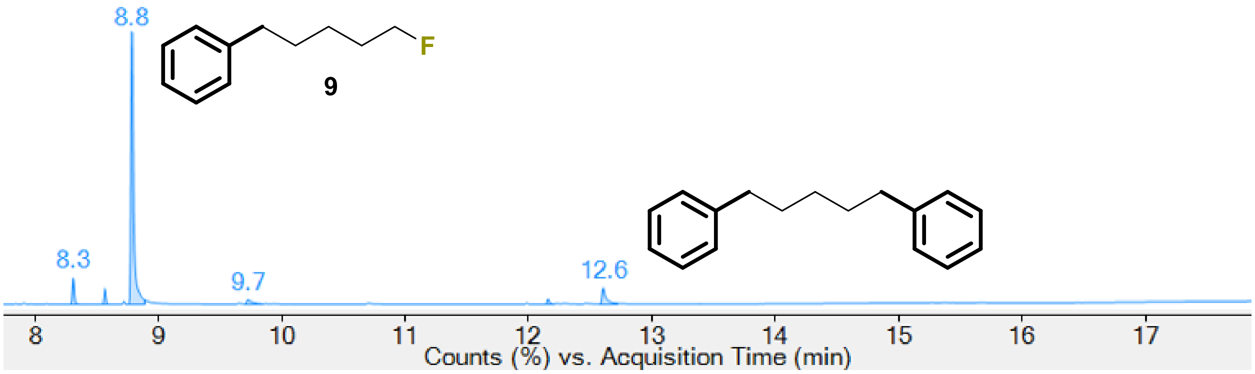

Figure S42. GC analysis of the reaction between 1-bromo-5-fluoropentane and diphenylzinc. (5-Fluoropentyl)benzene retention time = 8.8 minutes. 1,5-Diphenylpentane retention time = 12.6 minutes.

(5-Bromopentyl)benzene (8). Compound **8** was obtained from 1-bromo-5-fluoropentane (42.0 mg, 0.25 mmol) and triphenylaluminum (359.0 mM in chlorobenzene, 1.045 mL, 0.375 mmol, 1.5 equiv.) according to method C. The product was isolated by column purification using 100% hexanes as mobile phase as a colorless oil in 90% yield (51.0 mg, 0.23 mmol). ^{1}H NMR (400 MHz, chloroform-*d*) δ = 7.31 – 7.24 (m, 2H), 7.21 – 7.15 (m, 3H), 3.40 (t, *J* = 6.9 Hz, 2H), 2.63 (t, *J* = 7.7 Hz, 2H), 1.89 (m, 2H), 1.66 (m, 2H), 1.49 (m, 2H); ^{13}C NMR (100 MHz, chloroform-*d*) δ = 142.3, 128.4, 128.3, 125.7, 35.7, 33.7, 32.7, 30.6, 27.8. The spectroscopic data of (5-bromopentyl)benzene (**8**) are in accordance with the literature.[8]

Figure S43. ^{1}**H NMR spectrum of (5-bromopentyl)benzene (8).**

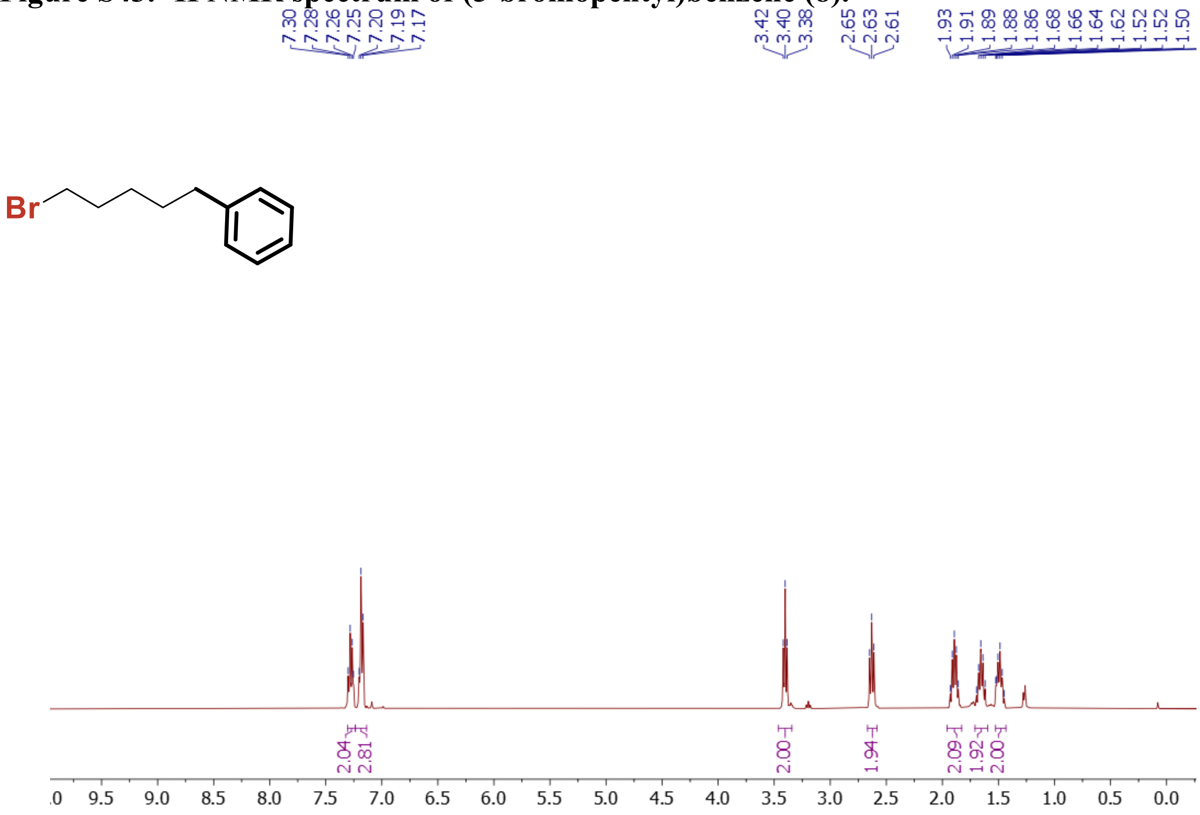

Figure S44. ^{13}**C NMR spectrum of (5-bromopentyl)benzene (8).**

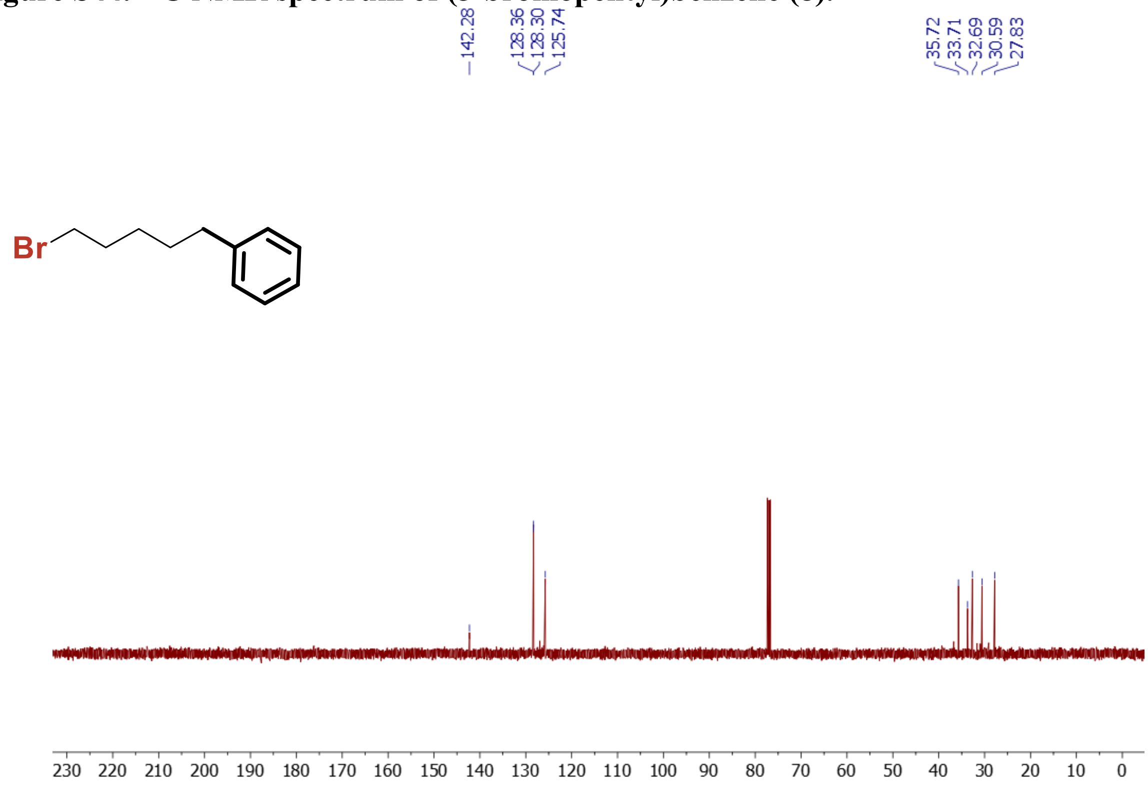

Reaction Time Variation with 1-Fluoro-3-iodopropane (10)

To explore the robustness of the alkyl fluoride selectivity, 1-fluoro-3-iodopropane (**10**) was combined with trimesitylaluminum for an extended period of time. 1-Fluoro-3-iodopropane (10.0 mg, 0.05 mmol) and trimesitylaluminum (359.0 mM in chlorobenzene, 0.148 μL, 0.080 mmol, 1.5 equiv.) were combined at room temperature. The reaction was allowed to stir for one week, after which an aliquot was quenched with methanol and diluted in dichloromethane for GC-MS analysis. We found that full conversion to 2-(3-iodopropyl)-1,3,5-trimethylbenzene (**11**) was observed after 2 hours at 25 °C and the product was isolated in excellent yield. Even though the alkyl fluoride is consumed in just two hours, the reaction was allowed to stir at 25 °C for an entire week with full preservation of the alkyl iodide bond.

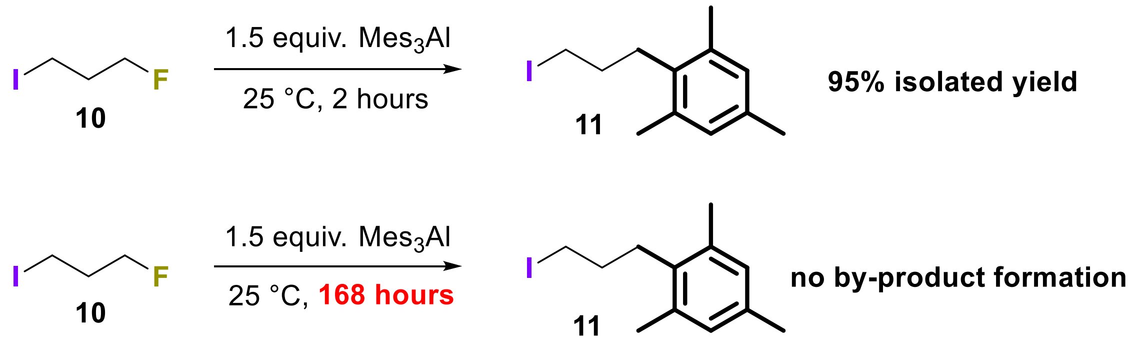

Figure S45. Reaction of 1-fluoro-3-iodopropane and trimesitylaluminum after two hours and one week, respectively. In both cases, the alkyl iodide bond is fully preserved, and no by-product formation is observed.

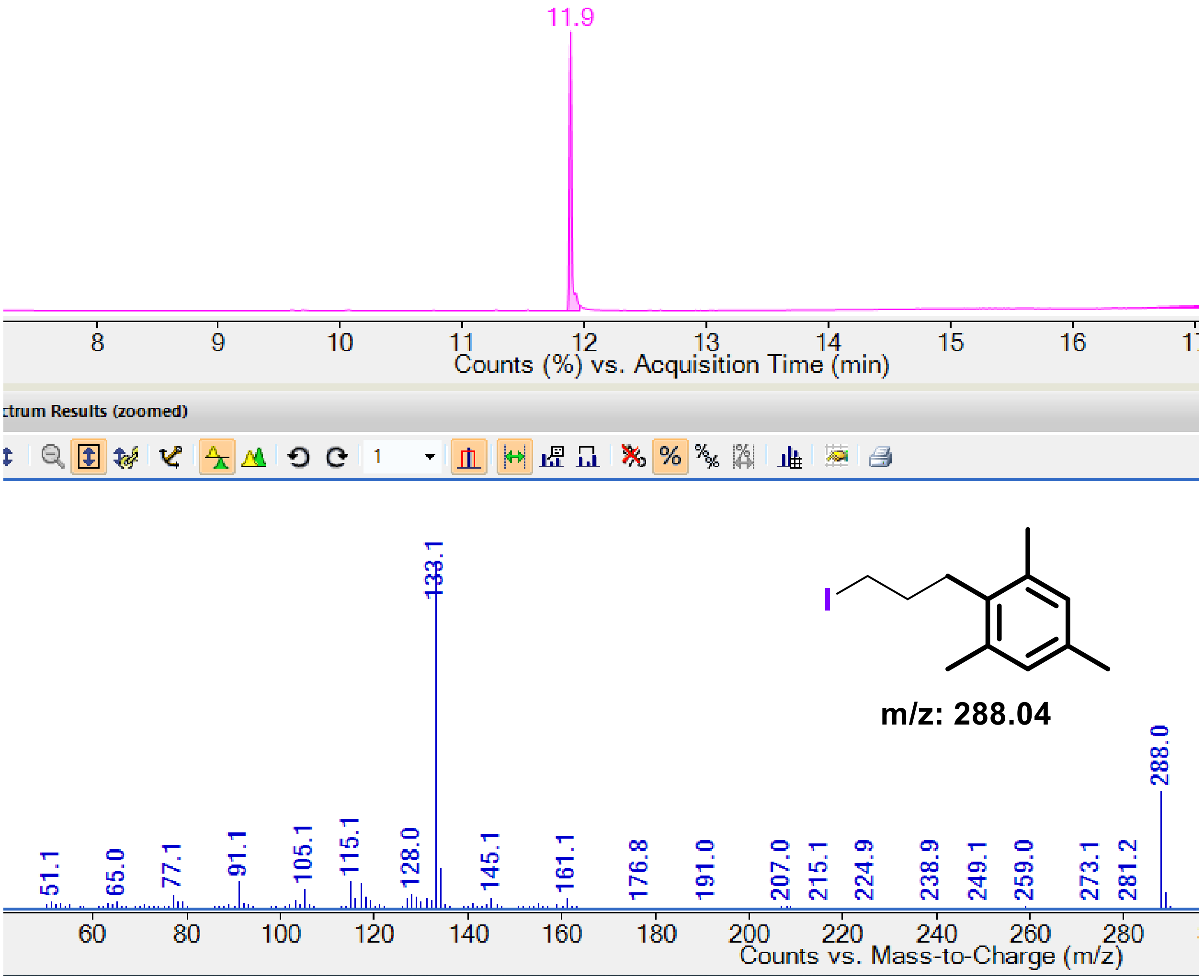

Figure S46. GC-MS analysis of the reaction between 1-fluoro-3-iodopropane (**10**) and trimesitylaluminum after stirring for one week at room temperature. No by-products are generated.

2-(3-Iodopropyl)-1,3,5-trimethylbenzene (11). Compound **11** was obtained from 1-fluoro-3-iodopropane (47.0 mg, 0.25 mmol) and trimesitylaluminum (359.0 mM in chlorobenzene, 1.045 mL, 0.375 mmol, 1.5 equiv.) according to a modified method C. Note that the reaction was allowed to stir for 2 hours at 25 °C. The product was isolated by column purification using 100% hexanes as mobile phase as a colorless oil in 95% yield (68.0 mg, 0.24 mmol). ^{1}H NMR (400 MHz, chloroform-*d*) δ = 6.84 (s, 2H), 3.29 (t, *J* = 6.8 Hz, 2H), 2.70 (m, 2H), 2.30 (s, 6H), 2.25 (s, 3H),

1.96 (m, 2H); ^{13}C NMR (100 MHz, chloroform-*d*) δ = 136.0, 135.4, 134.4, 129.0, 32.9, 30.5, 20.8, 19.8, 6.9. HRMS (ESI-TOF) *m/z*: $[M+H]^+$ Calcd for $C_{12}H_{18}I$ 289.0453; Found 289.0444.

Figure S47.^{1}H NMR spectrum of 2-(3-iodopropyl)-1,3,5-trimethylbenzene (11).

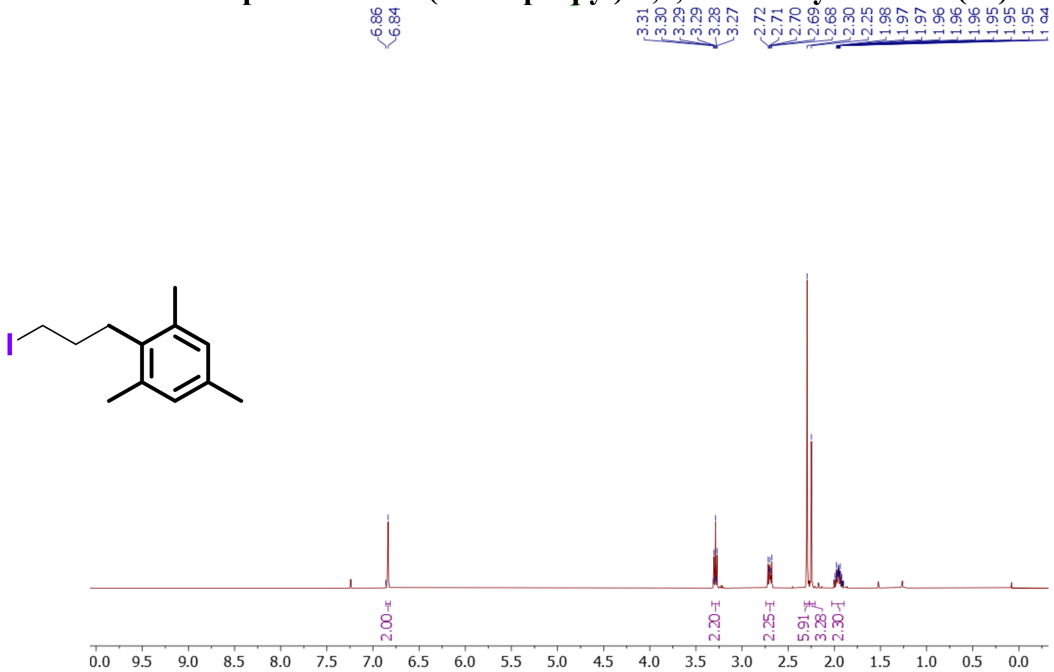

Figure S48.^{13}C NMR spectrum of 2-(3-iodopropyl)-1,3,5-trimethylbenzene (11).

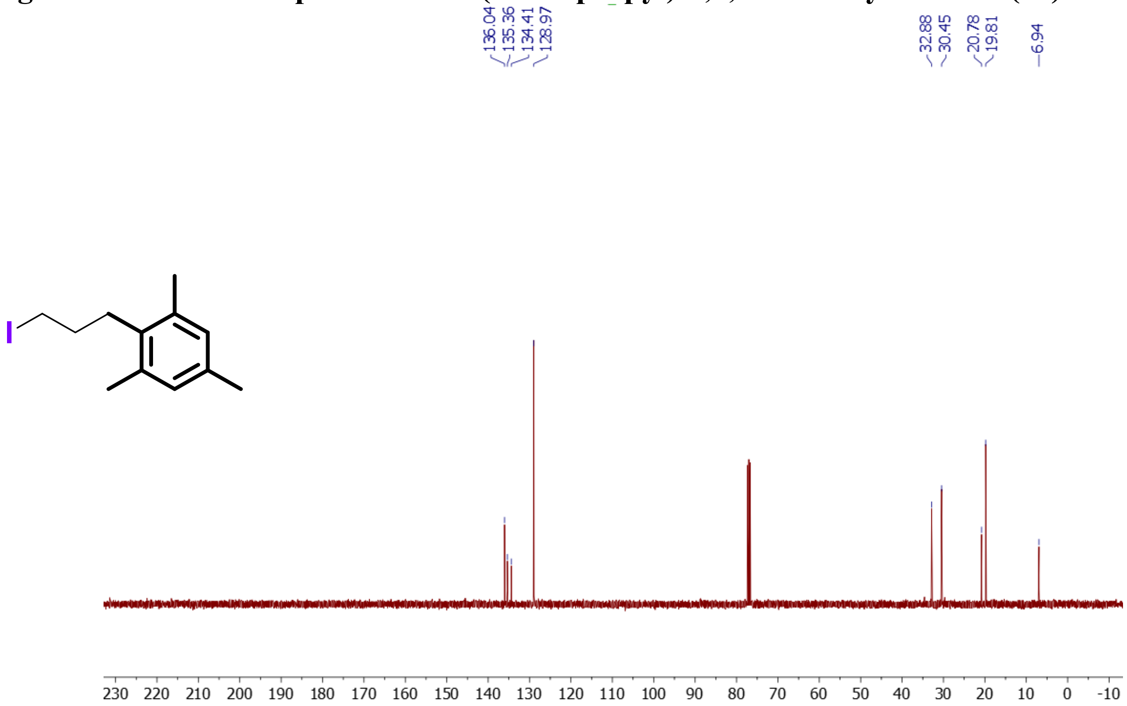

8. Coupling of Alkyl Chlorides, Bromides and Iodides with Ph_3Al at 50 ºC and 100 ºC

In a nitrogen-filled glove box, to a vial containing 1-iodooctane (6.97 mg, 0.029 mmol) was added triphenylaluminum (359.0 mM in chlorobenzene, 0.122 mL, 0.044 mmol, 1.5 equiv.) and the resulting solution was allowed to stir at 50 °C for two hours. An aliquot was taken and quenched with *tert*-butyl alcohol for GC-MS analysis. The reaction mixture was then allowed to stir for another two hours at 100 °C. After cooling to room temperature, the reaction mixture was then quenched with *tert*-butyl alcohol and analyzed by GC-MS. The same protocol was repeated with 1-chlorooctane, 1-bromooctane, chlorocyclohexane, bromocyclohexane, iodocyclohexane, 1-chloroadamantane and 1-bromoadamantane.

Among the primary halides, 1-chlorooctane was most reactive. The reaction was slow at 50 ºC but the formation of 1-phenyloctane was observed. However, side reactions toward Friedel-Crafts products derived from the chlorobenzene solvent were favored at 100 ºC. This was also the case with the primary bromide analog. By contrast, the reaction with Ph_3Al was favored when 1-iodooctane was used although large amounts of undesirable isomers were obtained. The secondary and tertiary halides reacted quantitatively at 50 ºC but mostly via Friedel-Crafts reaction with the chlorobenzene solvent. The adamantyl halides also showed substantial amounts of dehalogenation toward adamantane.

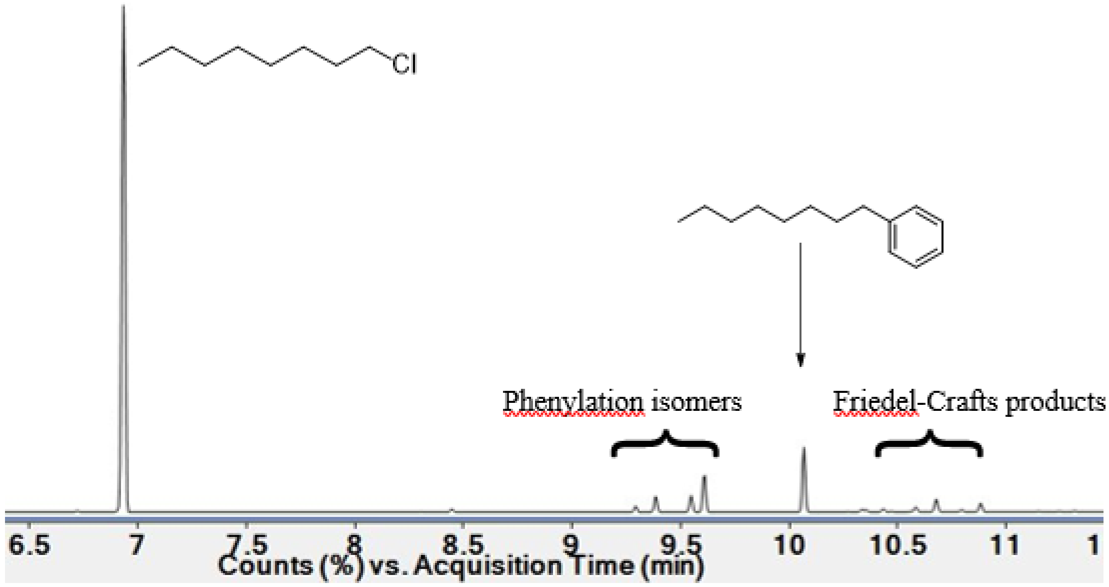

Figure S49. GC-MS analysis of the reaction between triphenylaluminum and 1-chloroctane after two hours at 50 °C. Multiple isomers of the Friedel-Crafts and the phenylation product were observed.

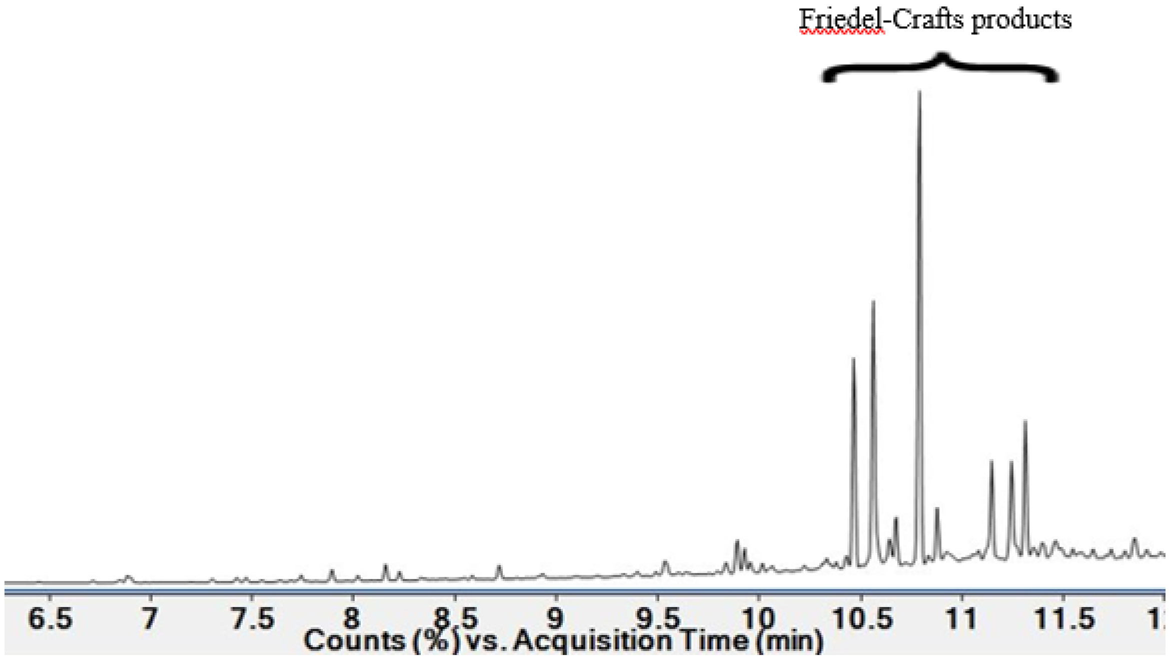

Figure S50. GC-MS analysis of the reaction between triphenylaluminum and 1-chloroctane after

two hours at 100 °C. Multiple isomers of the Friedel-Crafts reaction and only traces of phenylation products were observed.

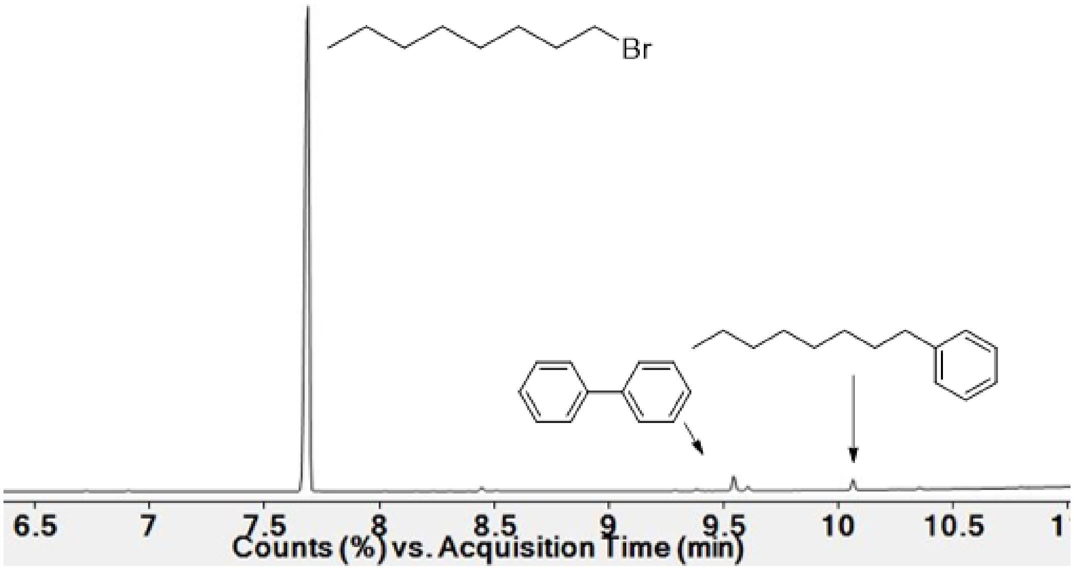

Figure S51. GC-MS analysis of the reaction between triphenylaluminum and 1-bromoctane after two hours at 50 °C. Trace amounts of the phenylation product were observed.

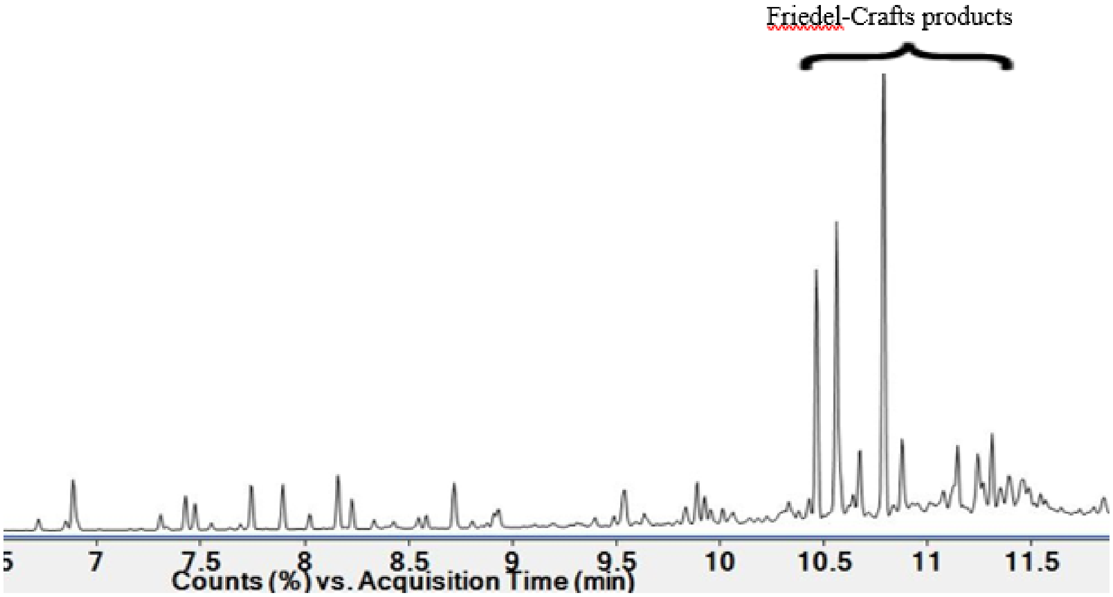

Figure S52. GC-MS analysis of the reaction between triphenylaluminum and 1-bromoctane after two hours at 100 °C. Mainly, Friedel-Crafts reaction products were formed.

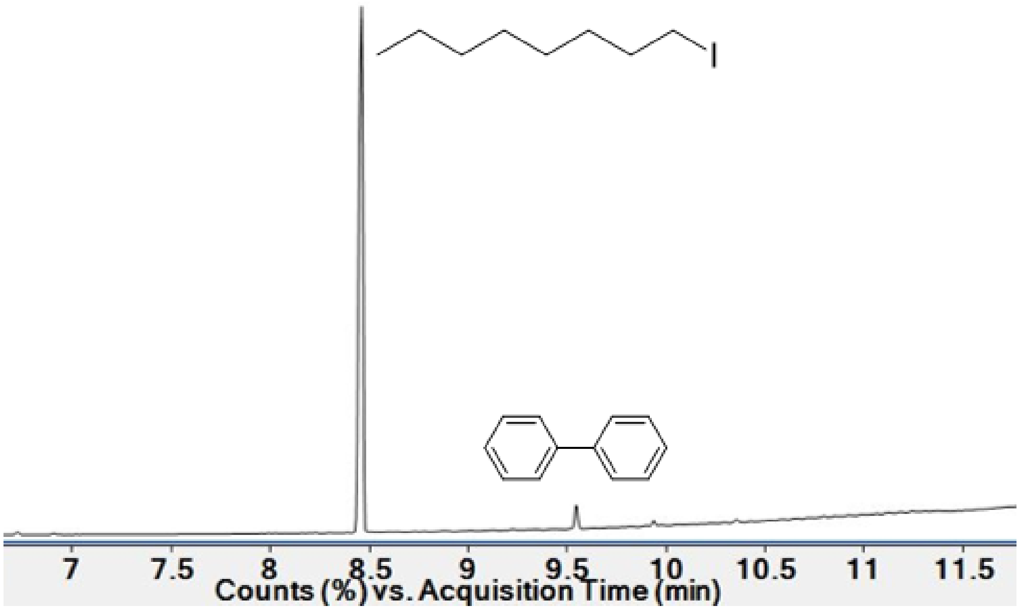

Figure S53. GC-MS analysis of the reaction between triphenylaluminum and 1-iodooctane after two hours at 50 °C.

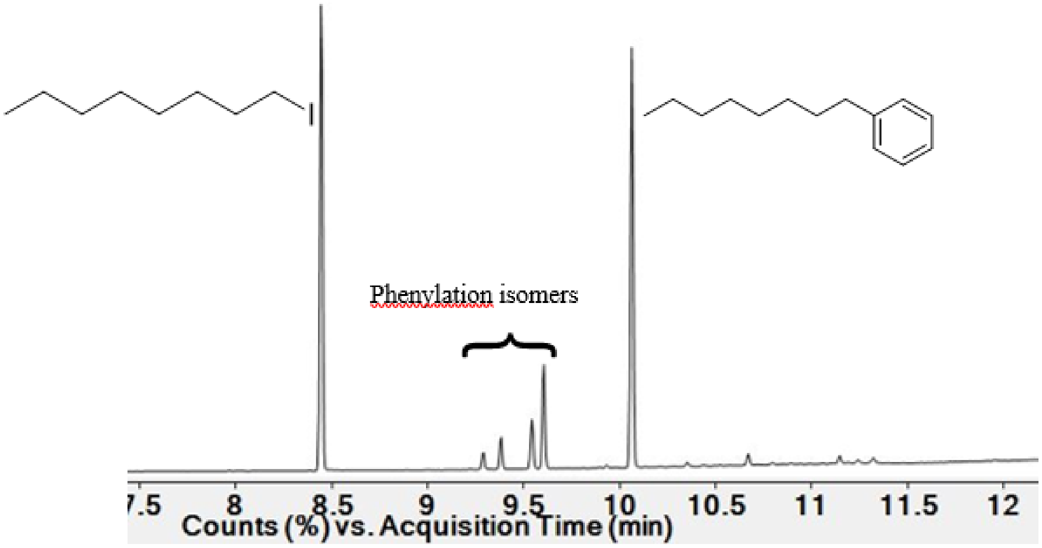

Figure S54. GC-MS analysis of the reaction between triphenylaluminum and 1-iodoctane after two hours at 100 °C.

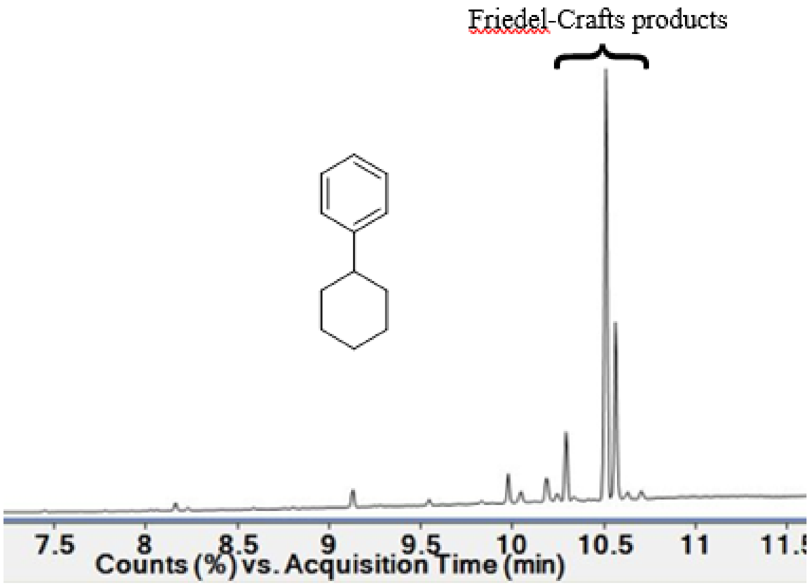

Figure S55. GC-MS analysis of the reaction between triphenylaluminum and chlorocyclohexane after two hours at 50 °C. The phenylation product and multiple isomers of the Friedel-Crafts products were observed.

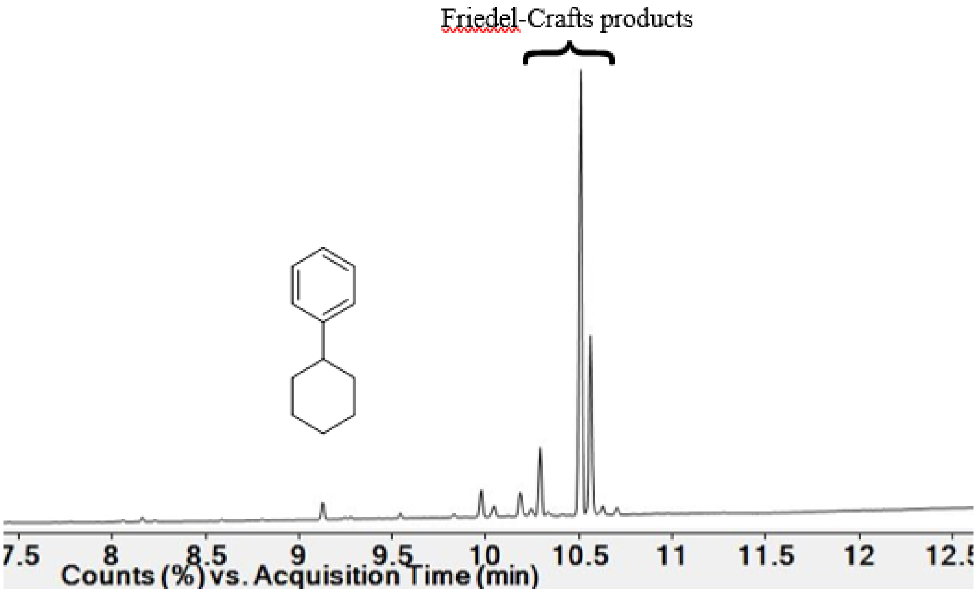

Figure S56. GC-MS analysis of the reaction between triphenylaluminum and bromocyclohexane after two hours at 50 °C. The phenylation product and multiple isomers of the Friedel-Crafts products were observed.

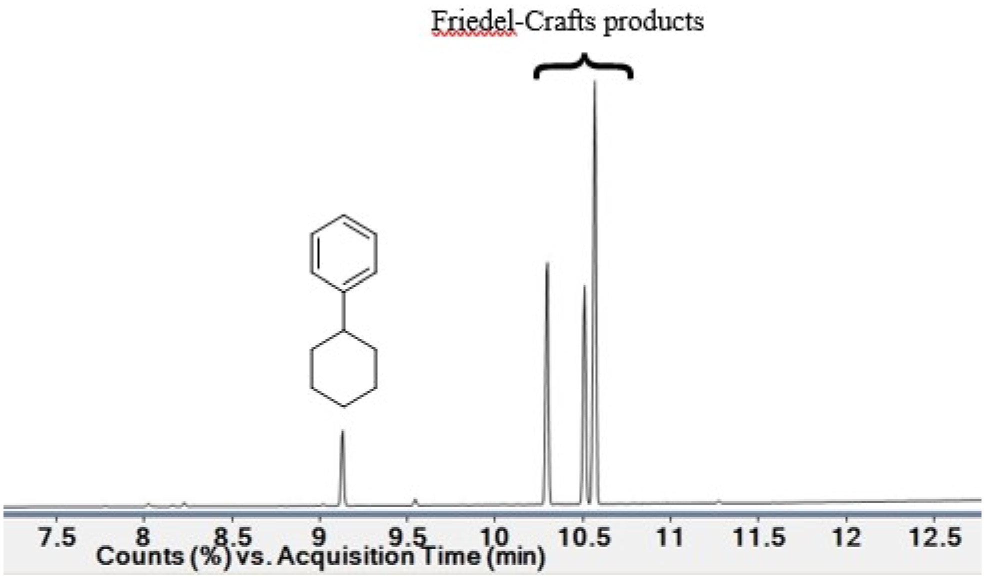

Figure S57. GC-MS analysis of the reaction between triphenylaluminum and iodocyclohexane after two hours at 50°C. A 13:1 ratio of Friedel-Crafts products to the phenylation product was obtained.

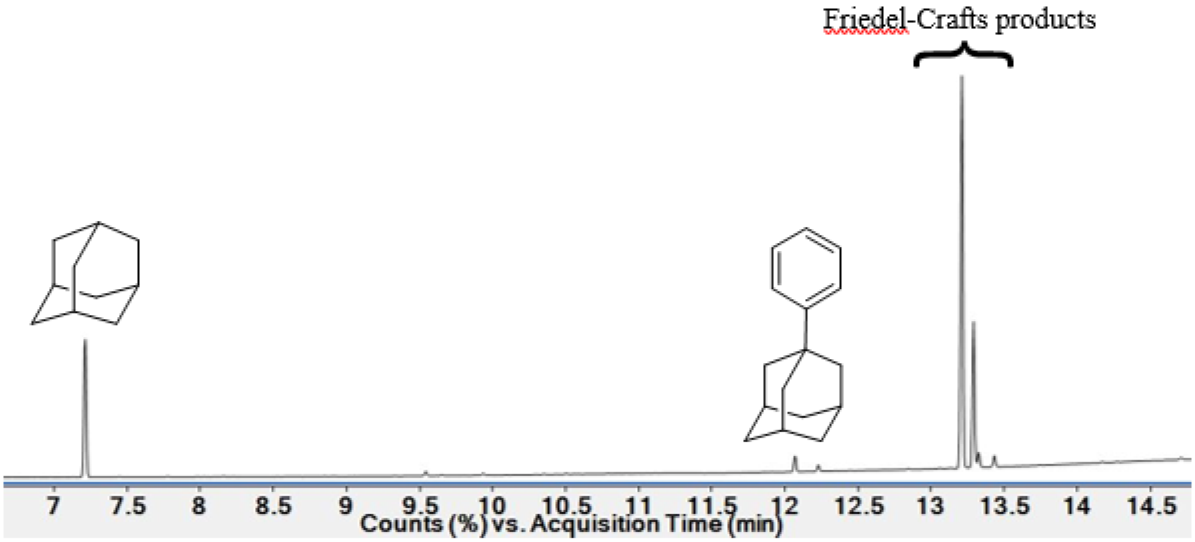

Figure S58. GC-MS analysis of the reaction between triphenylaluminum and 1-chloroadamantane after two hours at 50 °C. Multiple isomers of the Friedel-Crafts and phenylation products were observed.

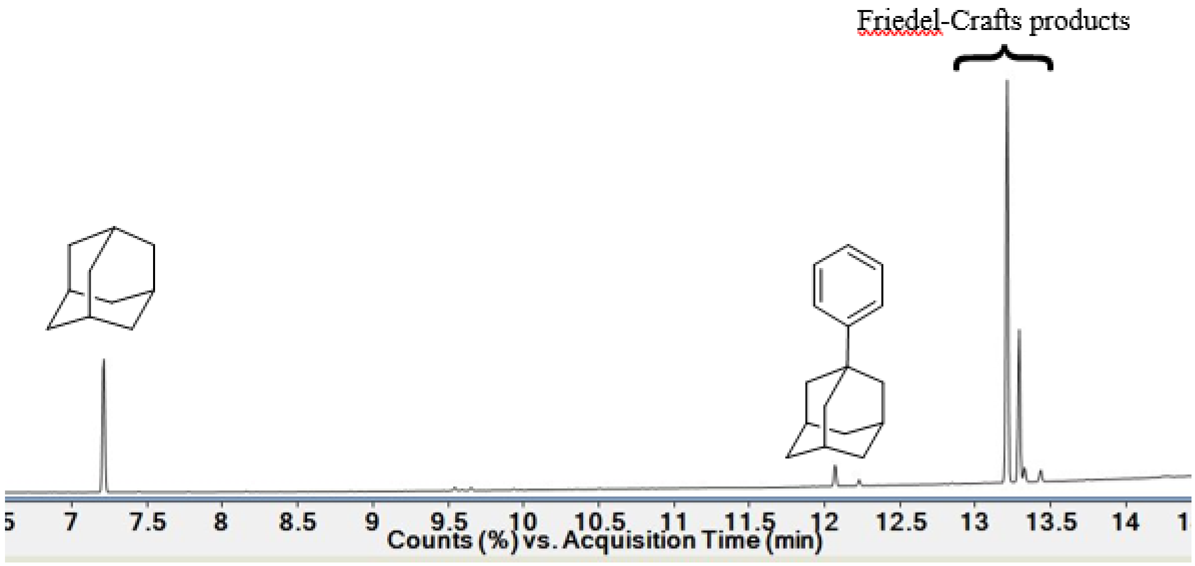

Figure S59. GC-MS analysis of the reaction between triphenylaluminum and 1-bromoadamantane after two hours at 50 °C. Multiple isomers of the Friedel-Crafts and phenylation products were observed

9. Product Synthesis, Purification and Characterization

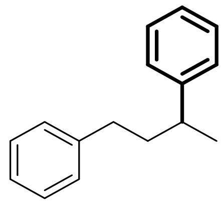

1,3-Diphenylbutane (5). Compound **5** was obtained from (3-fluorobutyl)benzene (38.0 mg, 0.25 mmol) and triphenylaluminum (359.0 mM in chlorobenzene, 1.045 mL, 0.375 mmol, 1.5 equiv.) according to method B. The product was isolated by column purification using 100% hexanes as mobile phase as a colorless oil in 85% yield (45.0 mg, 0.21 mmol). ^{1}H NMR (400 MHz, chloroform-*d*) δ = 7.36 – 7.27 (m, 2H), 7.24 – 7.11 (m, 8H), 2.71 (m, 1H), 2.51 (m, 2H), 1.92 (m, 2H), 1.28 (d, J = 6.9 Hz, 3H); ^{13}C NMR (100 MHz, chloroform-*d*) δ = 147.3, 142.5, 128.3, 128.3, 128.2, 127.0, 125.9, 125.6, 39.9, 39.5, 33.9, 22.5. The spectroscopic data of 1,3-diphenylbutane (**5**) are in accordance with the literature.[3]

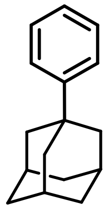

1-Phenyladamantane (6). Compound **6** was obtained from 1-fluoroadamantane (38.0 mg, 0.25 mmol) and triphenylaluminum (1.0 M in dibutyl ether, 125.0 μL, 0.125 mmol, 0.5 equiv.) according to method A. The product was isolated by column purification using 100% hexanes as mobile phase as a white crystalline solid in 91% yield (45.0 mg, 0.23 mmol). ^{1}H NMR (400 MHz, chloroform-*d*) δ = 7.43 – 7.31 (m, 4H), 7.21 (m, 1H), 2.13 (m, 3H), 1.96 (d, J = 2.9 Hz, 6H), 1.81 (m, 6H); ^{13}C NMR (100 MHz, chloroform-*d*) δ = 151.3, 128.1, 125.5, 124.8, 43.2, 36.8, 36.2, 29.0. The spectroscopic data of 1-phenyladamantane (**6**) are in accordance with the literature.[2]

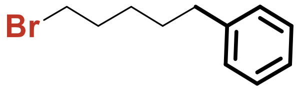

(5-Bromopentyl)benzene (8). Compound **8** was obtained from 1-bromo-5-fluoropentane (42.0 mg, 0.25 mmol) and triphenylaluminum (359.0 mM in chlorobenzene, 1.045 mL, 0.375 mmol, 1.5 equiv.) according to method C. The product was isolated by column purification using 100% hexanes as mobile phase as a colorless oil in 90% yield (51.0 mg, 0.23 mmol). ^{1}H NMR (400 MHz, chloroform-*d*) δ = 7.31 – 7.24 (m, 2H), 7.21 – 7.15 (m, 3H), 3.40 (t, *J* = 6.9 Hz, 2H), 2.63 (t, *J* = 7.7 Hz, 2H), 1.89 (m, 2H), 1.66 (m, 2H), 1.49 (m, 2H); ^{13}C NMR (100 MHz, chloroform-*d*) δ = 142.3, 128.4, 128.3, 125.7, 35.7, 33.7, 32.7, 30.6, 27.8. The spectroscopic data of (5-bromopentyl)benzene (**8**) are in accordance with the literature.[8]

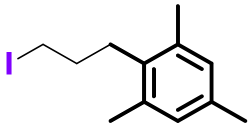

2-(3-Iodopropyl)-1,3,5-trimethylbenzene (11). Compound **11** was obtained from 1-fluoro-3-iodopropane (47.0 mg, 0.25 mmol) and trimesitylaluminum (359.0 mM in chlorobenzene, 1.045 mL, 0.375 mmol, 1.5 equiv.) according to a modified method C. Note that the reaction was allowed to stir at 25 °C. The product was isolated by column purification using 100% hexanes as mobile phase as a colorless oil in 95% yield (68.0 mg, 0.24 mmol). ^{1}H NMR (400 MHz, chloroform-*d*) δ = 6.84 (s, 2H), 3.29 (t, *J* = 6.8 Hz, 2H), 2.70 (m, 2H), 2.30 (s, 6H), 2.25 (s, 3H), 1.96 (m, 2H); ^{13}C NMR (100 MHz, chloroform-*d*) δ = 136.0, 135.4, 134.4, 129.0, 32.9, 30.5, 20.7, 19.8, 6.9. HRMS (ESI-TOF) *m/z*: $[M+H]^+$ Calcd for $C_{12}H_{18}I$ 289.0453; Found 289.0444.

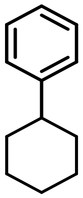

Cyclohexylbenzene (19). Compound **19** was obtained from fluorocyclohexane (26.0 mg, 0.25 mmol) and triphenylaluminum (359.0 mM in chlorobenzene, 1.045 mL, 0.375 mmol, 1.5 equiv.) according to method B. The product was isolated by column purification using 100% hexanes as mobile phase as a colorless oil in 84% yield (34.0 mg, 0.21 mmol). ^{1}H NMR (400 MHz, chloroform-*d*) δ = 7.34 – 7.26 (m, 2H), 7.26 – 7.16 (m, 3H), 2.50 (m, 1H), 1.97 – 1.83 (m, 4H), 1.76 (m, 1H), 1.52 – 1.27 (m, 5H); ^{13}C NMR (100 MHz, chloroform-*d*) δ = 148.1, 128.3, 126.8, 125.7, 44.6, 34.5, 26.9, 26.2. The spectroscopic data of cyclohexylbenzene (**19**) are in accordance with the literature.[9]

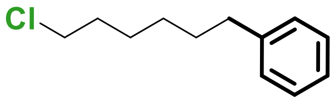

(6-Chlorohexyl)benzene (39). Compound **39** was obtained from 1-chloro-6-fluorohexane (35.0 mg, 0.25 mmol) and triphenylaluminum (359.0 mM in chlorobenzene, 1.045 mL, 0.375 mmol, 1.5 equiv.) according to method C. The product was isolated by column purification using 100% hexanes as mobile phase as a colorless oil in 95% yield (46.0 mg, 0.24 mmol). ^{1}H NMR (400 MHz, chloroform-*d*) δ = 7.31 – 7.23 (m, 2H), 7.21 – 7.14 (m, 3H), 3.51 (t, *J* = 6.7 Hz, 2H), 2.61 (t, *J* = 7.7 Hz, 2H), 1.76 (m, 2H), 1.63 (m, 2H), 1.46 (m, 2H), 1.36 (m, 2H); ^{13}C NMR (100 MHz, chloroform-*d*) δ = 142.6, 128.4, 128.2, 125.6, 45.1, 35.8, 32.5, 31.2, 28.5, 26.7. The spectroscopic data of (6-chlorohexyl)benzene (**39**) are in accordance with the literature.[10]

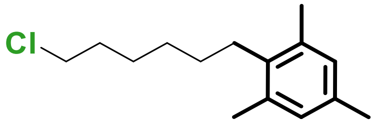

2-(6-Chlorohexyl)-1,3,5-trimethylbenzene (40). Compound **40** was obtained from 1-chloro-6-fluorohexane (35.0 mg, 0.25 mmol) and trimesitylaluminum (359.0 mM in chlorobenzene, 1.045 mL, 0.375 mmol, 1.5 equiv.) according to a modified method C. Note that the reaction was allowed to stir at 25 °C. The product was isolated by column purification using 100% hexanes as mobile phase as a colorless oil in 96% yield (57.0 mg, 0.24 mmol). ^{1}H NMR (400 MHz, chloroform-*d*) δ = 6.83 (s, 2H), 3.55 (t, *J* = 6.7 Hz, 2H), 2.57 (m, 2H), 2.28 (s, 6H), 2.25 (s, 3H), 1.79 (m, 2H), 1.52 – 1.42 (m, 6H); ^{13}C NMR (100 MHz, chloroform-*d*) δ = 136.4, 135.8, 134.8, 128.8, 45.1, 32.6, 29.5, 29.3, 29.2, 26.8, 20.8, 19.7. HRMS (ESI-TOF) *m/z*: $[M+H]^+$ Calcd for $C_{15}H_{24}Cl$ 239.1567; Found 239.1557.

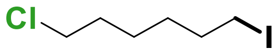

1-Chloro-6-iodohexane (41). Compound **41** was obtained from 1-chloro-6-fluorohexane (35.0 mg, 0.25 mmol) and aluminum iodide (153.0 mg, 0.375 mmol) stirring in 1.0 mL of 1,4-dioxane at 25 °C for 2 hours. The product was isolated by column purification using 100% hexanes as mobile phase as a yellow oil in 88% yield (54.0 mg, 0.22 mmol). ^{1}H NMR (400 MHz, chloroform-*d*) δ = 3.52 (t, *J* = 6.6 Hz, 2H), 3.17 (t, *J* = 7.0 Hz, 2H), 1.86 – 1.71 (m, 4H), 1.47 – 1.38 (m, 4H); ^{13}C NMR (100 MHz, chloroform-*d*) δ = 44.9, 33.2, 32.3, 29.7, 25.8, 6.8. The spectroscopic data of 1-chloro-6-iodohexane (**41**) are in accordance with the literature.[11]

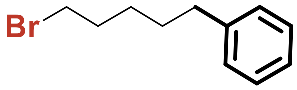

(5-Bromopentyl)benzene (42). Compound **42** was obtained from 1-bromo-5-fluoropentane (42.0 mg, 0.25 mmol) and triphenylaluminum (359.0 mM in chlorobenzene, 1.045 mL, 0.375 mmol, 1.5 equiv.) according to method C. The product was isolated by column purification using 100% hexanes as mobile phase as a colorless oil in 90% yield (51.0 mg, 0.23 mmol). ^{1}H NMR (400 MHz, chloroform-*d*) δ = 7.31 – 7.24 (m, 2H), 7.21 – 7.15 (m, 3H), 3.40 (t, *J* = 6.9 Hz, 2H), 2.63 (t, *J* = 7.7 Hz, 2H), 1.89 (m, 2H), 1.66 (m, 2H), 1.49 (m, 2H); ^{13}C NMR (100 MHz, chloroform-*d*) δ = 142.3, 128.4, 128.3, 125.7, 35.7, 33.7, 32.7, 30.6, 27.8. The spectroscopic data of (5-bromopentyl)benzene (**42**) are in accordance with the literature.[8]

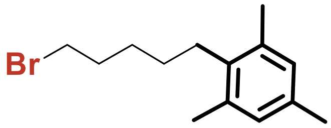

2-(5-Bromopentyl)-1,3,5-trimethylbenzene (43). Compound **43** was obtained from 1-bromo-5-fluoropentane (42.0 mg, 0.25 mmol) and trimesitylaluminum (359.0 mM in chlorobenzene, 1.045 mL, 0.375 mmol, 1.5 equiv.) according to a modified method C. Note that the reaction was allowed to stir at 25 °C. The product was isolated by column purification using 100% hexanes as mobile phase as a colorless oil in 89% yield (60.0 mg, 0.23 mmol). ^{1}H NMR (400 MHz, chloroform-*d*) δ = 6.83 (s, 2H), 3.43 (t, *J* = 6.8 Hz, 2H), 2.59 (m, 2H), 2.28 (s, 6H), 2.24 (s, 3H), 1.92 (m, 2H), 1.63 – 1.40 (m, 4H); ^{13}C NMR (100 MHz, chloroform-*d*) δ = 136.1, 135.8, 134.9, 128.9, 33.8, 32.6, 29.2, 28.8, 28.5, 20.8, 19.7. HRMS (ESI-TOF) *m/z*: $[M+H]^+$ Calcd for $C_{14}H_{22}Br$ 271.0884; Found 271.0880.

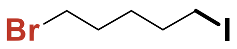

1-Bromo-5-iodopentane (44). Compound **44** was obtained from 1-bromo-5-fluoropentane (42.0 mg, 0.25 mmol) and aluminum iodide (153.0 mg, 0.375 mmol) stirring in 1.0 mL of 1,4-dioxane at 25 °C for 2 hours. The product was isolated by column purification using 100% hexanes as mobile phase as a brown oil in 90% yield (62.0 mg, 0.23 mmol). ^{1}H NMR (400 MHz, chloroform-*d*) δ = 3.39 (t, *J* = 6.8 Hz, 2H), 3.17 (t, *J* = 6.9 Hz, 2H), 1.91 – 1.80 (m, 4H), 1.55 (m, 2H); ^{13}C NMR (100 MHz, chloroform-*d*) δ = 33.2, 32.6, 31.6, 29.1, 6.2 The spectroscopic data of 1-bromo-5-iodopentane (**44**) are in accordance with the literature.[12]

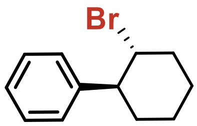

(*trans*-2-Bromocyclohexyl)benzene (46). Compound **46** was obtained from *trans*-1-bromo-2-fluorocyclohexane (45.0 mg, 0.25 mmol) and triphenylaluminum (359.0 mM in chlorobenzene, 1.045 mL, 0.375 mmol, 1.5 equiv.) according to method B. The product was isolated by column purification using 100% hexanes as mobile phase as a colorless oil in 84% yield (50.0 mg, 0.21 mmol). ^{1}H NMR (400 MHz, chloroform-*d*) δ = 7.35 – 7.28 (m, 2H), 7.26 – 7.15 (m, 3H), 4.17 (ddd, *J* = 11.5, 11.5, 4.2 Hz, 1H), 2.81 (ddd, *J* = 11.4, 11.4, 3.6 Hz, 1H), 2.52 (m, 1H), 1.97 (m, 2H), 1.82 (m, 2H), 1.62 – 1.37 (m, 3H); ^{13}C NMR (100 MHz, chloroform-*d*) δ = 144.7, 128.4, 127.1, 126.7, 57.8, 54.1, 39.0, 36.4, 27.6, 25.9.

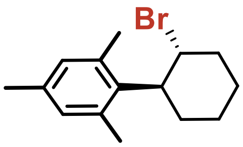

2-(*trans*-2-Bromocyclohexyl)-1,3,5-trimethylbenzene (47). Compound **47** was obtained from *trans*-1-bromo-2-fluorocyclohexane (45.0 mg, 0.25 mmol) and trimesitylaluminum (359.0 mM in chlorobenzene, 1.045 mL, 0.375 mmol, 1.5 equiv.) according to a method B. Note that the reaction was allowed to stir at 25 °C. The product was isolated by column purification using 100% hexanes as mobile phase as a colorless oil in 81% yield (57.0 mg, 0.20 mmol). ^{1}H NMR (400 MHz, chloroform-*d*) δ = 6.83 (s, 1H), 6.78 (s, 1H), 4.71 (ddd, *J* = 11.5, 11.5, 4.2 Hz, 1H), 3.34 (dd, *J* = 11.8, 11.8, 4.2 Hz, 1H), 2.52 (m, 1H), 2.43 (s, 3H), 2.32 (s, 3H), 2.22 (s, 3H), 1.92 – 1.82 (m, 5H), 1.46 – 1.38 (m, 2H); ^{13}C NMR (100 MHz, chloroform-*d*) δ = 137.2, 136.6, 135.5, 135.1, 131.2, 129.4, 55.5, 49.6, 39.7, 31.6, 27.7, 26.5, 21.6, 21.4, 20.7. HRMS (ESI-TOF) *m/z*: $[M+Na]^+$ Calcd for $C_{15}H_{21}BrNa$ 305.0704; Found 305.0692.

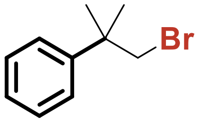

(1-Bromo-2-methylpropan-2-yl)benzene (49). Compound **49** was obtained from 1-bromo-2-fluoro-2-methylpropane (39.0 mg, 0.25 mmol) and triphenylaluminum (359.0 mM in chlorobenzene, 1.045 mL, 0.375 mmol, 1.5 equiv.) according to method C. Note that method C was used instead of method A for tertiary alkyl fluorides. The product was isolated by column purification using 100% hexanes as mobile phase as a colorless oil in 79% yield (42.0 mg, 0.20 mmol). ^{1}H NMR (400 MHz, chloroform-*d*) δ = 7.39 – 7.24 (m, 5H), 3.57 (s, 2H), 1.46 (s, 6H); ^{13}C

NMR (100 MHz, chloroform-*d*) δ = 146.0, 128.3, 126.5, 125.8, 46.9, 39.1, 27.2. The spectroscopic data of (1-bromo-2-methylpropan-2-yl)benzene (**49**) are in accordance with the literature.[13]

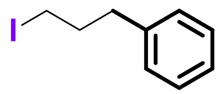

(3-Iodopropyl)benzene (50). Compound **50** was obtained from 1-fluoro-3-iodopropane (47.0 mg, 0.25 mmol) and triphenylaluminum (359.0 mM in chlorobenzene, 1.045 mL, 0.375 mmol, 1.5 equiv.) according to method C. The product was isolated by column purification using 100% hexanes as mobile phase as a colorless oil in 88% yield (54.0 mg, 0.22 mmol). ^{1}H NMR (400 MHz, chloroform-*d*) δ = 7.32 – 7.23 (m, 2H), 7.23 – 7.15 (m, 3H), 3.17 (t, *J* = 6.9 Hz, 2H), 2.72 (t, *J* = 7.3 Hz, 2H), 2.13 (m, 2H); ^{13}C NMR (100 MHz, chloroform-*d*) δ = 140.4, 128.5, 128.5, 126.2, 36.2, 34.9, 6.2. The spectroscopic data of (3-iodopropyl)benzene (**50**) are in accordance with the literature.[14]

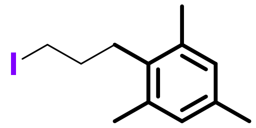

2-(3-Iodopropyl)-1,3,5-trimethylbenzene (51). Compound **51** was obtained from 1-fluoro-3-iodopropane (47.0 mg, 0.25 mmol) and trimesitylaluminum (359.0 mM in chlorobenzene, 1.045 mL, 0.375 mmol, 1.5 equiv.) according to a modified method C. Note that the reaction was allowed to stir at 25 °C. The product was isolated by column purification using 100% hexanes as mobile phase as a colorless oil in 95% yield (68.0 mg, 0.24 mmol). ^{1}H NMR (400 MHz, chloroform-*d*) δ = 6.84 (s, 2H), 3.29 (t, *J* = 6.8 Hz, 2H), 2.70 (m, 2H), 2.30 (s, 6H), 2.25 (s, 3H), 1.96 (m, 2H); ^{13}C NMR (100 MHz, chloroform-*d*) δ = 136.0, 135.4, 134.4, 129.0, 32.9, 30.5, 20.8, 19.8, 6.9. HRMS (ESI-TOF) *m/z*: $[M+H]^+$ Calcd for $C_{12}H_{18}I$ 289.0453; Found 289.0444.

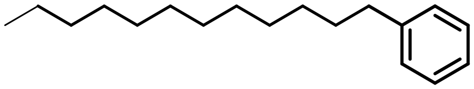

Dodecylbenzene (56). Compound **56** was obtained from 1-fluorododecane (47.0 mg, 0.25 mmol) and triphenylaluminum (359.0 mM in chlorobenzene, 1.045 mL, 0.375 mmol, 1.5 equiv.) according to method C. The product was isolated by column purification using 100% hexanes as mobile phase as a colorless oil in 93% yield (57.0 mg, 0.23 mmol). ^{1}H NMR (400 MHz, chloroform-*d*) δ = 7.29 (m, 2H), 7.20 – 7.16 (m, 3H), 2.60 (t, *J* = 7.8 Hz, 2H), 1.60 (m, 2H), 1.35 – 1.20 (m, 18H), 0.88 (m, 3H); ^{13}C NMR (100 MHz, chloroform-*d*) δ = 142.9, 128.4, 128.2, 125.5, 39.9, 38.4, 36.0, 31.9, 31.5, 29.7, 29.6, 29.6, 29.5, 29.3, 22.7, 14.1. The reaction gives a mixture of isomers that could not be separated. The given values correspond to the major isomer – 1-phenyldodecane. The spectroscopic data of dodecylbenzene (**56**) are in accordance with the literature.[15]

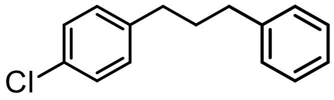

1-Chloro-4-(3-phenylpropyl)benzene (57). Compound **57** was obtained from 1-(3-chloropropyl)-4-fluorobenzene (43.0 mg, 0.25 mmol) and triphenylaluminum (359.0 mM in chlorobenzene, 1.045 mL, 0.375 mmol, 1.5 equiv.) according to method C. The product was isolated by column purification using 100% hexanes as mobile phase as a colorless oil in 80% yield (46.0 mg, 0.20 mmol). ^{1}H NMR (400 MHz, chloroform-*d*) δ = 7.33 – 7.23 (m, 4H), 7.22 – 7.16 (m, 3H), 7.12 (m, 2H), 2.68 – 2.58 (m, 4H), 1.95 (m, 2H); ^{13}C NMR (100 MHz, chloroform-

d) δ = 142.0, 140.6, 131.4, 129.7, 128.4, 128.4, 128.3, 125.8, 35.3, 34.7, 32.8. The spectroscopic data of 1-chloro-4-(3-phenylpropyl)benzene (**57**) are in accordance with the literature.[4]

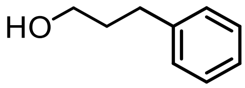

3-Phenylpropan-1-ol (58). Compound **58** was obtained from 3-fluoro-1-propanol (20.0 mg, 0.25 mmol) and triphenylaluminum (359.0 mM in chlorobenzene, 2.09 mL, 0.750 mmol, 3.0 equiv.) according to method C. Note that three equivalents of triphenylaluminum were used. The product was isolated by column purification using 5% ethyl acetate in hexanes as mobile phase as a colorless oil in 62% yield (21.0 mg, 0.16 mmol). ^{1}H NMR (400 MHz, chloroform-*d*) δ = 7.32 – 7.23 (m, 2H), 7.21 – 7.12 (m, 3H), 3.67 (t, *J* = 6.4 Hz, 2H), 2.74 – 2.66 (t, *J* = 7.6 Hz, 2H), 1.89 (m, 2H), 1.32 (s, 1H); ^{13}C NMR (100 MHz, chloroform-*d*) δ = 141.8, 128.4, 128.4, 125.8, 62.3, 34.2, 32.1. The spectroscopic data of 3-phenylpropan-1-ol (**58**) are in accordance with the literature.[16]

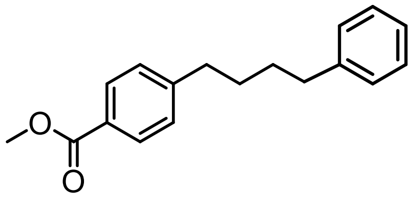

Methyl 4-(4-phenylbutyl)benzoate (59). Compound **59** was obtained from methyl 4-(4-fluorobutyl)benzoate (52.6 mg, 0.25 mmol) and triphenylaluminum (359.0 mM in chlorobenzene, 1.045 mL, 0.375 mmol, 1.5 equiv.) according to method C. The product was isolated by column purification using 2% ethyl acetate in hexanes as mobile phase as a colorless oil in 86% yield (46.0 mg, 0.20 mmol). ^{1}H NMR (400 MHz, chloroform-*d*) δ = 7.93 (d, *J* = 8.3 Hz, 2H), 7.29 - 7.19 (m,

4H), 7.19 - 7.11 (m, 3H), 3.89 (s, 3H), 2.67 (t, *J* = 7.1 Hz, 2H), 2.62 (t, *J* = 7.1 Hz, 2H), 1.72 - 1.59 (m, 4H); ^{13}C NMR (100 MHz, chloroform-*d*) δ = 167.1, 148.1, 142.3, 129.7, 129.6, 128.4, 128.4, 128.3, 125.7, 51.9, 35.8, 35.7, 31.0, 30.6. The spectroscopic data are of methyl 4-(4-phenylbutyl)benzoate (**59**) are in accordance with the literature.[4]

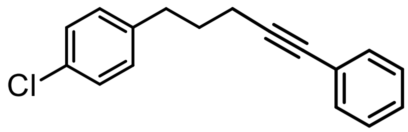

1-Chloro-4-(3-phenylpropyl)benzene (60). Compound **60** was obtained from 1-(3-chloropropyl)-4-fluorobenzene (43.0 mg, 0.25 mmol) and tris(phenylethynyl)aluminum (359.0 mM in chlorobenzene, 1.045 mL, 0.375 mmol, 1.5 equiv.) according to method C. The product was isolated by column purification using 100% hexanes as mobile phase as a brown oil in 94% yield (60.0 mg, 0.24 mmol). ^{1}H NMR (400 MHz, chloroform-*d*) δ = 7.42 (m, 2H), 7.34 – 7.22 (m, 5H), 7.15 (m, 2H), 2.77 (t, *J* = 7.2 Hz, 2H), 2.42 (t, *J* = 7.0 Hz, 2H), 1.90 (m, 2H); ^{13}C NMR (100 MHz, chloroform-*d*) δ = 140.0, 131.6, 131.6, 129.9, 128.5, 128.2, 127.6, 123.9, 89.5, 81.3, 34.1, 30.2, 18.7. HRMS (ESI-TOF) *m/z*: $[M+H]^+$ Calcd for $C_{17}H_{16}Cl$ 255.0941; Found 255.0933.

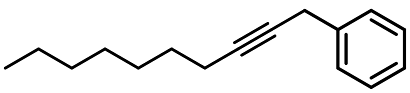

Dec-2-yn-1-ylbenzene (61). Compound **61** was obtained from 1-fluorodec-2-yne (39.0 mg, 0.25 mmol) and triphenylaluminum (1.0 M in dibutyl ether, 125.0 µL, 0.125 mmol, 0.5 equiv.) according to method A. The product was isolated by column purification using 100% hexanes as mobile phase as a colorless oil in 85% yield (45.0 mg, 0.21 mmol). ^{1}H NMR (400 MHz, chloroform-*d*) δ = 7.38 – 7.27 (m, 4H), 7.21 (m, 1H), 3.58 (t, *J* = 2.5 Hz, 2H), 2.22 (m, 2H), 1.53

(m, 2H), 1.41 (m, 2H), 1.34 – 1.28 (m, 6H), 0.90 (t, *J* = 6.9 Hz, 3H); ^{13}C NMR (100 MHz, chloroform-*d*) δ = 137.6, 128.3, 127.8, 126.3, 82.7, 77.4, 31.7, 29.0, 28.8, 28.8, 25.1, 22.6, 18.8, 14.1. HRMS (ESI-QTOF) *m/z:* $[M+H]^+$ Calcd for $C_{16}H_{22}$ 215.1800; Found 215.1787.

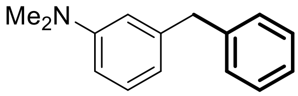

3-Benzyl-*N,N*-dimethylaniline (62). Compound **62** was obtained from 3-(fluoromethyl)-*N,N*-dimethylaniline (38.0 mg, 0.25 mmol) and triphenylaluminum (1.0 M in dibutyl ether, 125.0 µL, 0.125 mmol, 0.5 equiv.) according to method A. The product was isolated by column purification using 5% methanol in dichloromethane as mobile phase as a colorless oil in 80% yield (42.0 mg, 0.20 mmol). ^{1}H NMR (400 MHz, chloroform-*d*) δ = 7.25 – 7.17 (m, 3H), 7.17 – 7.08 (m, 3H), 7.00 (d, *J* = 8.2 Hz, 1H), 6.49 (d, *J* = 8.0 Hz, 2H), 4.06 (s, 2H), 2.85 (s, 6H); ^{13}C NMR (100 MHz, chloroform-*d*) δ = 149.7, 142.2, 140.8, 133.3, 129.0, 128.2, 125.7, 122.1, 114.6, 111.6, 40.5, 40.0. The spectroscopic data of 3-benzyl-*N,N*-dimethylaniline (**62**) are in accordance with the literature.[17]

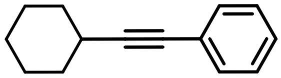

(Cyclohexylethynyl)benzene (63). Compound **63** was obtained from fluorocyclohexane (26.0 mg, 0.25 mmol) and tris(phenylethynyl)aluminum (359.0 mM in chlorobenzene, 1.045 mL, 0.375 mmol, 1.5 equiv.) according to method B. The product was isolated by column purification using 100% hexanes as mobile phase as a brown oil in 80% yield (37.0 mg, 0.20 mmol). ^{1}H NMR (400 MHz, chloroform-*d*) δ = 7.40 – 7.36 (m, 2H), 7.28 – 7.22 (m, 3H), 2.56 (m, 1H), 1.93 – 1.81 (m,

3H), 1.74 (m, 1H), 1.60 – 1.45 (m, 3H), 1.41 – 1.30 (m, 4H); ^{13}C NMR (100 MHz, chloroform-*d*) δ = 132.1, 131.5, 128.1, 124.1, 94.4, 80.4, 32.7, 31.9, 29.6, 29.3, 25.9, 24.9. The spectroscopic data of (cyclohexylethynyl)benzene (**63**) are in accordance with the literature.[18]

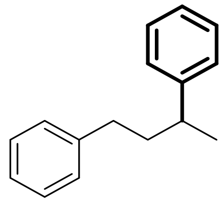

1,3-Diphenylbutane (64). Compound **64** was obtained from (3-fluorobutyl)benzene (38.0 mg, 0.25 mmol) and triphenylaluminum (359.0 mM in chlorobenzene, 1.045 mL, 0.375 mmol, 1.5 equiv.) according to method B. The product was isolated by column purification using 100% hexanes as mobile phase as a colorless oil in 85% yield (45.0 mg, 0.21 mmol). ^{1}H NMR (400 MHz, chloroform-*d*) δ = 7.36 – 7.27 (m, 2H), 7.24 – 7.11 (m, 8H), 2.71 (m, 1H), 2.51 (m, 2H), 1.92 (m, 2H), 1.28 (d, J = 6.9 Hz, 3H); ^{13}C NMR (100 MHz, chloroform-*d*) δ = 147.3, 142.5, 128.3, 128.3, 128.2, 127.0, 125.9, 125.6, 39.9, 39.5, 33.9, 22.5. The spectroscopic data of 1,3-diphenylbutane (**64**) are in accordance with the literature.[3]

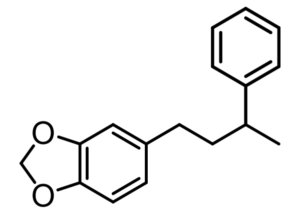

5-(3-Phenylbutyl)benzo[d][1,3]dioxole (65). Compound **65** was obtained from 5-(3-fluorobutyl)benzo[d][1,3]dioxole (49.0 mg, 0.25 mmol), and triphenylaluminum (359.0 mM in chlorobenzene, 1.045 mL, 0.375 mmol, 1.5 equiv.) according to method B. The product was isolated by column purification using 5% ethyl acetate in hexanes as mobile phase as a colorless oil in 82% yield (52.0 mg, 0.21 mmol). ^{1}H NMR (400 MHz, chloroform-*d*) δ = 7.31 (m, 2H), 7.22-

7.16 (m, 3H), 6.71 (d, J = 7.9 Hz, 1H), 6.63 (d, J = 1.7 Hz, 1H), 6.57 (dd, J = 7.9, 1.6 Hz, 1H), 5.91 (s, 2H), 2.71 (m, 1H), 2.43 (m, 2H), 1.87 (m, 2H), 1.27 (d, J = 6.9 Hz, 3H); ^{13}C NMR (100 MHz, chloroform-*d*) δ = 147.4, 147.2, 145.4, 136.4, 128.4, 127.0, 125.9, 121.0, 108.8, 108.0, 100.7, 40.2, 39.3, 33.6. The spectroscopic data of 5-(3-phenylbutyl)benzo[d][1,3]dioxole (**65**) are in accordance with the literature.[4]

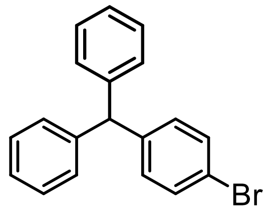

((4-Bromophenyl)methylene)dibenzene (66). Compound **66** was obtained from 1-bromo-4-(fluoro(phenyl)methyl)benzene (66.0 mg, 0.25 mmol) and triphenylaluminum (1.0 M in dibutyl ether, 125.0 μL, 0.125 mmol, 0.5 equiv.) according to method A. The product was isolated by column purification using 100% hexanes as mobile phase as a white crystalline solid in 92% yield (74.0 mg, 0.23 mmol). ^{1}H NMR (400 MHz, chloroform-*d*) δ = 7.61 (m, J = 8.0 Hz, 1H), 7.33 – 7.23 (m, 7H), 7.16 – 7.06 (m, 5H), 6.98 (m, 1H), 6.00 (s, 1H); ^{13}C NMR (100 MHz, chloroform-*d*) δ = 143.2, 142.6, 133.1, 131.4, 129.6, 128.3, 128.0, 127.2, 126.5, 125.6, 56.0. The spectroscopic data of ((4-bromophenyl)methylene)dibenzene (**66**) are in accordance with the literature.[19]

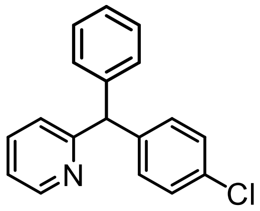

2-((4-Chlorophenyl)(phenyl)methyl)pyridine (67). Compound **67** was obtained from 2-((4-chlorophenyl)fluoromethyl)pyridine (55.0 mg, 0.25 mmol) and triphenylaluminum (1.0 M in dibutyl ether, 125.0 µL, 0.125 mmol, 0.5 equiv.) according to method A. The product was isolated by column purification using 2% ethyl acetate in hexanes as mobile phase as a brown solid in 81% yield (57.0 mg, 0.20 mmol). ^{1}H NMR (400 MHz, chloroform-*d*) δ = 8.59 (m, 1H), 7.60 (m, 1H), 7.30 – 7.22 (m, 5H), 7.16 – 7.05 (m, 6H), 5.64 (s, 1H); ^{13}C NMR (100 MHz, chloroform-*d*) δ = 162.6, 149.6, 142.2, 141.3, 136.5, 132.4, 130.7, 129.2, 128.5, 126.7, 123.7, 121.6, 110.0, 58.6. The spectroscopic data of 2-((4-chlorophenyl)(phenyl)methyl)pyridine (**67**) are in accordance with the literature.[20]

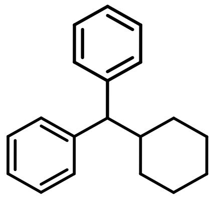

(Cyclohexylmethylene)dibenzene (68). Compound **68** was obtained from (cyclohexylfluoromethyl)benzene (48.0 mg, 0.25 mmol) and triphenylaluminum (1.0 M in dibutyl ether, 125.0 µL, 0.125 mmol, 0.5 equiv.) according to method A. The product was isolated by column purification using 100% hexanes as mobile phase as a colorless oil in 81% yield (51.0 mg, 0.20 mmol). ^{1}H NMR (400 MHz, chloroform-*d*) δ = 7.34 – 7.23 (m, 8H), 7.15 (m, 2H), 3.50 (d, *J* = 10.9 Hz, 1H), 2.14 (m, 1H), 1.77 – 1.56 (m, 5H), 1.35 – 1.10 (m, 3H), 0.90 (m, 2H); ^{13}C NMR

(100 MHz, chloroform-*d*) δ = 144.5, 128.4, 128.1, 125.9, 59.6, 41.3, 32.1, 26.6, 26.4. The spectroscopic data of (cyclohexylmethylene)dibenzene (**68**) are in accordance with the literature.[21]

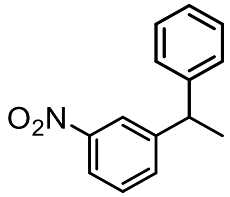

1-Nitro-3-(1-phenylethyl)benzene (69). Compound **69** was obtained from 1-(1-fluoroethyl)-3-nitrobenzene (42.0 mg, 0.25 mmol) and triphenylaluminum (1.0 M in dibutyl ether, 125.0 μL, 0.125 mmol, 0.5 equiv.) according to method A. The product was isolated by column purification using 5% ethyl acetate in hexanes as mobile phase as a brown oil in 79% yield (45.0 mg, 0.20 mmol). ^{1}H NMR (400 MHz, chloroform-*d*) δ = 8.12 (m, 1H), 8.05 (m, 1H), 7.54 (m, 1H), 7.44 (m, 1H), 7.32 (m, 2H), 7.26 – 7.19 (m, 3H), 4.27 (q, *J* = 7.2 Hz, 1H), 1.70 (d, *J* = 7.2 Hz, 3H); ^{13}C NMR (100 MHz, chloroform-*d*) δ = 148.5, 148.4, 144.7, 134.0, 129.3, 128.7, 127.5, 126.7, 122.4, 121.3, 44.5, 21.6. The spectroscopic data of 1-nitro-3-(1-phenylethyl)benzene (**65**) are in accordance with the literature.[9]

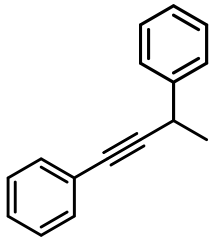

But-1-yne-1,3-diyldibenzene (70). Compound **70** was obtained from (3-fluorobut-1-yn-1-yl)benzene (37.0 mg, 0.25 mmol) and triphenylaluminum (1.0 M in dibutyl ether, 125.0 μL, 0.125 mmol, 0.5 equiv.) according to method A. The product was isolated by column purification using

100% hexanes as mobile phase as a colorless oil in 84% yield (43.0 mg, 0.21 mmol). ^{1}H NMR (400 MHz, chloroform-*d*) δ = 7.50 – 7.44 (m, 4H), 7.36 (m, 2H), 7.33 – 7.24 (m, 4H), 4.00 (q, *J* = 7.1 Hz, 1H), 1.60 (d, *J* = 7.1 Hz, 3H); ^{13}C NMR (100 MHz, chloroform-*d*) δ = 143.3, 131.6, 128.5, 128.2, 127.7, 126.9, 126.6, 123.7, 92.6, 82.4, 32.5, 24.5. The spectroscopic data of but-1-yne-1,3-diyldibenzene (**70**) are in accordance with the literature.[22]

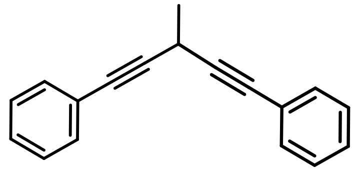

(3-Methylpenta-1,4-diyne-1,5-diyl)dibenzene (71). Compound **71** was obtained from (3-fluorobut-1-yn-1-yl)benzene (37.0 mg, 0.25 mmol) and tris(phenylethynyl)aluminum (359.0 mM in chlorobenzene, 1.045 mL, 0.375 mmol, 1.5 equiv.) according to a modified method C. Note that the reaction was allowed to stir for 24 hours. The product was isolated by column purification using 100% hexanes as mobile phase as a brown oil in 89% yield (51.0 mg, 0.22 mmol). ^{1}H NMR (400 MHz, chloroform-*d*) δ = 7.48 – 7.39 (m, 4H), 7.32 – 7.25 (m, 6H), 3.90 (q, *J* = 7.0 Hz, 1H), 1.62 (d, *J* = 7.1 Hz, 3H); ^{13}C NMR (100 MHz, chloroform-*d*) δ = 131.7, 128.2, 128.0, 123.1, 88.9, 80.4, 22.5, 19.1. HRMS (ESI-TOF) *m/z*: $[M+H]^+$ Calcd for $C_{18}H_{15}$ 231.1174; Found 231.1172.

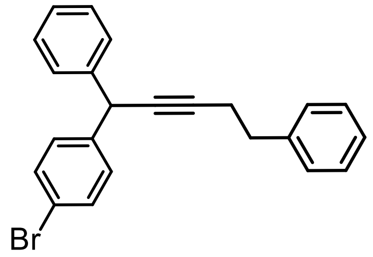

(1-(4-Bromophenyl)pent-2-yne-1,5-diyl)dibenzene (72). Compound **72** was obtained from 1-bromo-4-(fluoro(phenyl)methyl)benzene (66.0 mg, 0.25 mmol) and tris(4-phenylbut-1-yn-1-yl)aluminum (359.0 mM in chlorobenzene, 1.045 mL, 0.375 mmol, 1.5 equiv.) according to a modified method C. Note that the reaction was run at 25 °C. The product was isolated by column purification using 100% hexanes as mobile phase as a brown oil in 90% yield (84.0 mg, 0.23 mmol). ^{1}H NMR (400 MHz, chloroform-*d*) δ = 7.51 (m, 1H), 7.42 (m, 1H), 7.30 – 7.20 (m, 11H), 7.07 (m, 1H), 5.45 (s, 1H), 2.85 (t, *J* = 7.4 Hz, 2H), 2.57 (t, *J* = 7.4, Hz, 2H); ^{13}C NMR (100 MHz, chloroform-*d*) δ = 141.4, 140.8, 140.7, 132.8, 130.4, 128.6, 128.4, 128.4, 128.3, 127.9, 127.7, 126.7, 126.2, 123.7, 84.2, 81.0, 42.2, 35.1, 21.0.

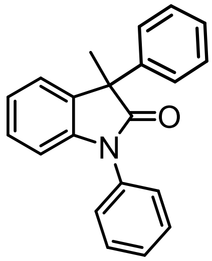

3-Methyl-1,3-diphenylindolin-2-one (73). Compound **73** was obtained from 3-fluoro-3-methyl-1-phenylindolin-2-one (60.0 mg, 0.25 mmol) and triphenylaluminum (1.0 M in dibutyl ether, 125.0 µL, 0.125 mmol, 0.5 equiv.) according to method A. The product was isolated by column purification using 5% ethyl acetate in hexanes as mobile phase as a white crystalline solid in 91% yield (68.0 mg, 0.23 mmol). ^{1}H NMR (400 MHz, chloroform-*d*) δ = 7.52 (m, 2H), 7.45 – 7.39 (m, 4H), 7.33 (m, 2H), 7.29 – 7.22 (m, 4H), 7.13 (m, 1H), 6.92 (m, 1H), 1.92 (s, 3H); ^{13}C NMR (100 MHz, chloroform-*d*) δ = 178.7, 143.1, 140.9, 134.7, 134.6, 129.5, 128.6, 127.9, 127.3, 126.7, 126.6, 124.5, 123.2, 115.3, 109.6, 52.2, 24.0. The spectroscopic data of 3-methyl-1,3-diphenylindolin-2-one (**73**) are in accordance with the literature.[23]

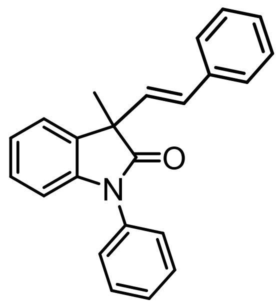

(*E*)-3-Methyl-1-phenyl-3-styrylindolin-2-one (74). Compound **74** was obtained from 3-fluoro-3-methyl-1-phenylindolin-2-one (60.0 mg, 0.25 mmol) and tri((*E*)-styryl)aluminum (359.0 mM in chlorobenzene, 1.045 mL, 0.375 mmol, 1.5 equiv.) according to a modified method C. Note that the reaction was allowed to stir at 25 °C for 24 hours. The product was isolated by column purification using 5% ethyl acetate in hexanes as mobile phase as a colorless oil in 74% yield (60.0 mg, 0.19 mmol). ^{1}H NMR (400 MHz, chloroform-*d*) δ = 7.57 – 7.48 (m, 2H), 7.46 – 7.32 (m, 8H), 7.32 – 7.20 (m, 2H), 7.17 (m, 1H), 6.88 (m, 1H), 6.50 (d, J = 16.1 Hz, 1H), 6.43 (d, J = 16.1 Hz, 1H), 1.71 (s, 3H); ^{13}C NMR (100 MHz, chloroform-*d*) δ = 178.0, 142.9, 136.5, 134.5, 132.7, 130.3, 130.0, 129.5, 128.5, 128.0, 128.0, 127.7, 126.6, 126.5, 124.3, 123.0, 109.7, 50.7, 23.6. HRMS (ESI-TOF) *m/z*: $[M+H]^+$ Calcd for $C_{23}H_{20}NO$ 326.1545; Found 326.1540.

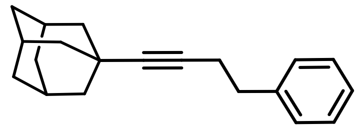

1-(4-Phenylbut-1-yn-1-yl)adamantane (75). Compound **75** was obtained from 1-fluoroadamantane (38.0 mg, 0.25 mmol) and tris(4-phenylbut-1-yn-1-yl)aluminum (359.0 mM in chlorobenzene, 1.045 mL, 0.375 mmol, 1.5 equiv.) according to a modified method C. Note that

the reaction was allowed to stir at 25 °C. The product was isolated by column purification using 100% hexanes as mobile phase as a brown solid in 94% yield (62.0 mg, 0.24 mmol). ^{1}H NMR (400 MHz, chloroform-*d*) δ = 7.31 – 7.19 (m, 5H), 2.78 (t, *J* = 7.6 Hz, 2H), 2.43 (t, *J* = 7.4 Hz, 2H), 1.93 (m, 3H), 1.81 (m, 6H), 1.66 (m, 6H); ^{13}C NMR (100 MHz, chloroform-*d*) δ = 141.1, 128.6, 128.2, 126.0, 89.8, 78.2, 43.3, 36.4, 35.8, 29.5, 28.1, 21.1. HRMS (ESI-TOF) *m/z*: $[M+H]^+$ Calcd for $C_{20}H_{25}$ 265.1956; Found 265.1950.

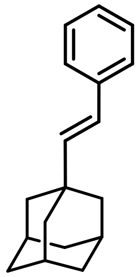

1-((*E*)-Styryl)adamantane (76). Compound **76** was obtained from 1-fluoroadamantane (38.0 mg, 0.25 mmol) and tri((*E*)-styryl)aluminum (359.0 mM in chlorobenzene, 1.045 mL, 0.375 mmol, 1.5 equiv.) according to a modified method C. Note that the reaction was allowed to stir at 25 °C for 24 hours. The product was isolated by column purification using 100% hexanes as mobile phase as a colorless oil in 76% yield (44.0 mg, 0.19 mmol). ^{1}H NMR (400 MHz, chloroform-*d*) δ = 7.37 – 7.33 (m, 2H), 7.30 – 7.26 (m, 2H), 7.16 (m, 1H), 6.23 (d, *J* = 16.2 Hz, 1H), 6.09 (d, *J* = 16.2 Hz, 1H), 2.02 (m, 3H), 1.75 – 1.63 (m, 12H); ^{13}C NMR (100 MHz, chloroform-*d*) δ = 142.1, 138.2, 128.4, 126.7, 126.0, 124.5, 42.2, 36.9, 35.1, 28.5. HRMS (ESI-TOF) *m/z*: $[M+H]^+$ Calcd for $C_{18}H_{23}$ 239.1800; Found 239.1787.

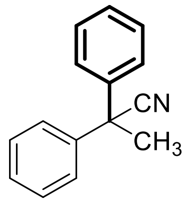

2,2-Diphenylpropanenitrile (77). Compound **77** was obtained from 2-fluoro-2-phenylpropanenitrile (37.0 mg, 0.25 mmol) and triphenylaluminum (1.0 M in dibutyl ether, 125.0 μL, 0.125 mmol, 0.5 equiv.) according to method A. The product was isolated by column purification using 5% ethyl acetate in hexanes as mobile phase as a colorless oil in 87% yield (45.0 mg, 0.22 mmol). ^{1}H NMR (400 MHz, chloroform-*d*) δ = 7.41 – 7.31 (m, 10H), 2.10 (s, 3H); ^{13}C NMR (100 MHz, chloroform-*d*) δ = 141.3, 128.9, 127.9, 126.6, 123.4, 46.2, 28.1. The spectroscopic data of 2,2-diphenylpropanenitrile (**73**) are in accordance with the literature.[24]

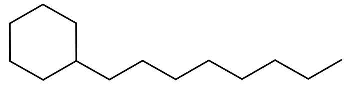

Octylcyclohexane (80). Compound **80** was obtained from fluorocyclohexane (26.0 mg, 0.25 mmol) and trioctylaluminum solution (25 wt% in hexanes, 0.25 mmol) according to the general procedure for alkylation. The reaction was allowed to stir at 25 °C for 2 hours. The product was isolated by column purification using 100% hexanes as mobile phase as a colorless oil in 79% yield (39.0 mg, 0.20 mmol). ^{1}H NMR (400 MHz, chloroform-*d*) δ = 1.71 – 1.58 (m, 4H), 1.32 – 1.07 (m, 18H), 0.90 – 0.77 (m, 6H); ^{13}C NMR (100 MHz, chloroform-*d*) δ = 37.7, 37.6, 33.5, 31.9, 30.0, 29.7, 29.4, 26.9, 26.8, 26.5, 22.7, 14.1. Characterization data are consistent with the literature.[25]

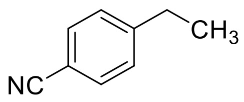

4-Ethylbenzonitrile (81). Compound **81** was obtained from 4-(fluoromethyl)benzonitrile (34.0 mg, 0.25 mmol) and trimethylaluminum solution (2.0 M hexanes, 0.50 mmol) according to the general procedure for alkylation. Dichloroethane (0.5 mL) was used as a co-solvent. The reaction was allowed to stir at 25 °C for 18 hours. The product was isolated by column purification using 10% ethyl acetate in hexanes as mobile phase as a yellow oil in 88% yield (29.0 mg, 0.23 mmol). ^{1}H NMR (400 MHz, chloroform-*d*) δ = 7.54 (d, *J* = 7.2 H, 2H), 7.27 (d, *J* = 7.2 H, 2H), 2.68 (q, *J* = 7.6 Hz, 2H), 1.24 (t, *J* = 7.6 Hz, 3H); ^{13}C NMR (100 MHz, chloroform-*d*) δ = 149.8, 132.3, 128.8, 119.2, 109.5, 29.1, 14.9. Characterization data are consistent with the literature.[26]

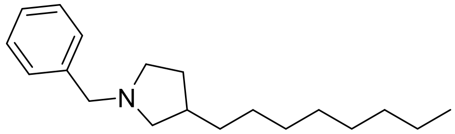

1-Benzyl-3-octylpyrrolidine (82). Compound **82** was obtained from 1-benzyl-3-fluoropyrrolidine (45.0 mg, 0.25 mmol) and trioctylaluminum solution (25 wt% in hexanes, 0.25 mmol) according to the general procedure for alkylation. The reaction was allowed to stir at 25 °C for 2 hours. Dichloroethane (0.5 mL) was used as a co-solvent. The product was isolated by column purification using 5% ethyl acetate in hexanes as mobile phase as a colorless oil in 97% yield (68.0 mg, 0.24 mmol). ^{1}H NMR (400 MHz, chloroform-*d*) δ = 7.34 – 7.20 (m, 5H), 3.67 – 3.52 (m, 2H), 2.81 (m, 1H), 2.69 (m, 1H), 2.39 (m, 1H), 2.11 (m, 1H), 2.03 - 1.93 (m, 2H), 1.55 (m, 1H), 1.43 – 1.17 (m, 14H), 0.87 (t, *J* = 6.9, 3H); ^{13}C NMR (100 MHz, chloroform-*d*) δ = 139.4, 128.8, 128.2, 126.8, 63.1, 60.9, 54.0, 37.6, 35.8, 31.9, 30.9, 29.8, 29.3, 28.4, 25.7, 22.7, 14.1. HRMS (ESI-TOF) m/z: [M+H]+ Calcd for $C_{19}H_{31}N$: 274.2535; Found: 274.2535.

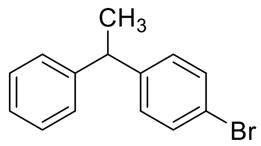

1-Bromo-4-(1-phenylethyl)benzene (83). Compound **83** was obtained from 1-bromo-4-(fluoro(phenyl)methyl)benzene (66.0 mg, 0.25 mmol) and trimethylaluminum (2.0 M in hexanes, 0.125 mmol.) according to the general procedure for alkylation. The product was isolated by column purification using 100% hexanes as mobile phase as a yellow oil in 94% yield (61.0 mg, 0.24 mmol). ^{1}H NMR (400 MHz, chloroform-*d*) δ = 7.53 (m, 1H), 7.30 – 7.25 (m, 2H), 7.24 – 7.15 (m, 5H), 7.04 (m, 1H), 4.63 (q, *J* = 7.2 Hz, 1H), 1.60 (d, *J* = 7.2 Hz, 3H); ^{13}C NMR (100 MHz, chloroform-*d*) δ = 145.4, 144.9, 133.0, 128.7, 128.4, 127.9, 125.7, 124.3, 43.4, 21.2. Characterization data are consistent with the literature.[27]

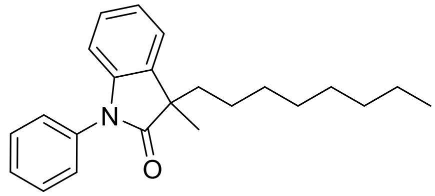

3-Methyl-3-octyl-1-phenylindolin-2-one (84). Compound **84** was obtained from 3-fluoro-3-methyl-1-phenylindolin-2-one (60.0 mg, 0.25 mmol) and trioctylaluminum solution (25 wt% in hexanes, 0.25 mmol) according to the general procedure for alkylation. The reaction was allowed to stir at 25 °C for 3 hours. Dichloroethane (0.5 mL) was used as a co-solvent. The product was isolated by column purification using 5% ethyl acetate in hexanes as mobile phase as an amorphous solid in 72% yield (60.0 mg, 0.18 mmol). ^{1}H NMR (400 MHz, chloroform-*d*) δ = 7.53 – 7.46 (m, 2H), 7.42 – 7.34 (m, 3H), 7.24 – 7.14 (m, 2H), 7.08 (m, 1H), 6.82 (m, 1H), 1.98 (m, 1H), 1.79 (m, 1H), 1.45 (s, 3H), 1.29 – 1.07 (m, 12H), 0.83 (t, *J* = 7.0 Hz, 3H); ^{13}C NMR (100 MHz, chloroform-*d*) δ = 180.2, 143.2, 134.7, 134.1, 129.5, 127.8, 127.4, 126.5, 122.8, 122.8, 109.1, 48.5, 39.0, 31.7,

29.7, 29.3, 29.2, 24.5, 24.1, 22.6, 14.0. HRMS (ESI-TOF) m/z: [M+H]+ Calcd for $C_{23}H_{30}NO$: 336.2327; Found: 336.2322.

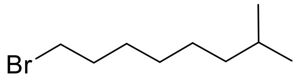

1-Bromo-7-methyloctane (85). Compound **85** was obtained from 1-bromo-5-fluoropentane (42.0 mg, 0.25 mmol) and triisobutylaluminum solution (1.0 M in hexanes, 0.25 mmol) according to the general procedure for alkylation. The reaction was allowed to stir at 25 °C for 2 hours. The product was isolated by column purification using 100% hexanes as mobile phase as a colorless oil in 70% yield (36.0 mg, 0.18 mmol). ^{1}H NMR (400 MHz, chloroform-*d*) δ = 3.39 (t, *J* = 6.9 Hz, 2H), 1.84 (m, 2H), 1.51 (m, 1H), 1.40 (m, 2H), 1.29 – 1.21 (m, 4H), 1.14 (m, 2H), 0.85 (d, *J* = 6.6 Hz, 6H); ^{13}C NMR (100 MHz, chloroform-*d*) δ = 38.9, 34.1, 32.8, 29.0, 28.2, 27.9, 27.2, 22.6. Characterization data are consistent with the literature.[28]

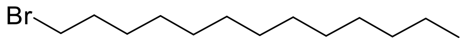

1-Bromotridecane (86). Compound **86** was obtained from 1-bromo-5-fluoropentane (42.0 mg, 0.25 mmol) and trioctylaluminum solution (25 wt% in hexanes, 0.25 mmol) according to the general procedure for alkylation. The reaction was allowed to stir at 25 °C for 3 hours. The product was isolated by column purification using 100% hexanes as mobile phase as a colorless oil in 79% yield (52.0 mg, 0.20 mmol). ^{1}H NMR (400 MHz, chloroform-*d*) δ = 3.39 (t, *J* = 6.9 Hz, 2H), 1.84 (m, 2H), 1.40 (m, 2H), 1.32 – 1.18 (m, 18H), 0.86 (t, *J* = 6.8 Hz, 3H); ^{13}C NMR (100 MHz, chloroform-*d*) δ = 34.0, 32.8, 31.9, 29.6, 29.6, 29.6, 29.5, 29.4, 29.3, 28.8, 28.2, 22.7, 14.1. Characterization data are consistent with the literature.[29]

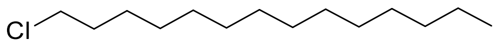

1-Chlorotetradecane (87). Compound **87** was obtained from 1-chloro-6-fluorohexane (35.0 mg, 0.25 mmol) and trioctylaluminum solution (25 wt% in hexanes, 0.25 mmol) according to the general procedure for alkylation. The reaction was allowed to stir at 25 °C for 3 hours. The product was isolated by column purification using 100% hexanes as mobile phase as a colorless oil in 81% yield (47.0 mg, 0.20 mmol). ^{1}H NMR (400 MHz, chloroform-*d*) δ = 3.51 (t, *J* = 6.8 Hz, 2H), 1.75 (m, 2H), 1.40 (m, 2H), 1.31 – 1.20 (m, 20H), 0.86 (t, *J* = 6.8 Hz, 3H); ^{13}C NMR (100 MHz, chloroform-*d*) δ = 45.2, 32.6, 31.9, 29.7, 29.6, 29.6, 29.6, 29.5, 29.4, 29.3, 28.9, 26.9, 22.7, 14.1. Characterization data are consistent with the literature.[30]

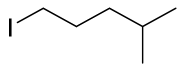

1-Iodo-4-methylpentane (88). Compound **88** was obtained from 1-fluoro-2-iodoethane (87.0 mg, 0.50 mmol) and triisobutylaluminum solution (1.0 M in hexanes, 0.50 mmol) according to the general procedure for alkylation. The reaction was allowed to stir at 25 °C for 2 hours. The product was isolated by filtration through a silica gel plug using 100% pentane as the eluent as a colorless oil in 44% yield (47.0 mg, 0.22 mmol). ^{1}H NMR (400 MHz, chloroform-*d*) δ = 3.16 (t, *J* = 7.1 Hz, 2H), 1.81 (m, 2H), 1.56 (m, 1H), 1.25 (m, 2H), 0.88 (d, *J* = 6.6 Hz, 6H); ^{13}C NMR (100 MHz, chloroform-*d*) δ = 39.7, 31.6, 27.0, 22.6, 7.4. Characterization data are consistent with the literature.[31]

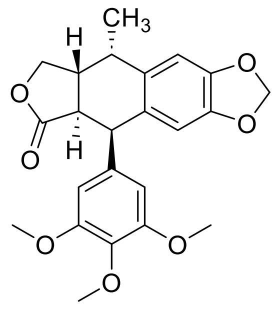

(5*R*,5a*R*,8a*R*,9*S*)-9-Methyl-5-(3,4,5-trimethoxyphenyl)-5,8,8a,9-tetrahydrofuro[3',4':6,7]naphtho[2,3-d][1,3]dioxol-6(5aH)-one (90). Compound **90** was obtained from (5*R*,5a*R*,8a*R*,9*S*)-9-fluoro-5-(3,4,5-trimethoxyphenyl)-5,8,8a,9-tetrahydrofuro[3',4':6,7]naphtho[2,3-d][1,3]dioxol-6(5aH)-one (104.0 mg, 0.25 mmol) and trimethylaluminum solution (2.0 M in hexanes, 0.375 mmol.) according to the general procedure for alkylation. Dichloroethane (0.5 mL) was used as a co-solvent. The reaction was allowed to stir at 25 °C for 18 hours. The product was isolated by column purification using 20% ethyl acetate in hexanes as mobile phase as a white crystalline solid in 89% yield (92.0 mg, 0.22 mmol). ^{1}H NMR (400 MHz, chloroform-*d*) δ = 6.69 (s, 1H), 6.45 (s, 1H), 6.29 (s, 2H), 5.92 (d, *J* = 7.9 Hz, 2H), 4.54 (d, *J* = 4.0 Hz, 1H), 4.29 (m, 1H), 4.08 (m, 1H), 3.78 (s, 3H), 3.73 (s, 6H), 3.20 (m, 1H), 3.01 – 2.85 (m, 2H), 1.23 (d, *J* = 7.1 Hz, 3H); ^{13}C NMR (100 MHz, chloroform-*d*) δ = 175.2, 152.4, 147.1, 146.7, 137.1, 136.1, 135.1, 130.2, 110.3, 108.6, 108.3, 101.2, 68.8, 60.6, 56.3, 44.1, 41.2, 35.8, 33.7, 18.6. HRMS (ESI-TOF) m/z: [M+Na]+ Calcd for $C_{23}H_{24}O_{7}Na$: 435.1420; Found: 435.1413.

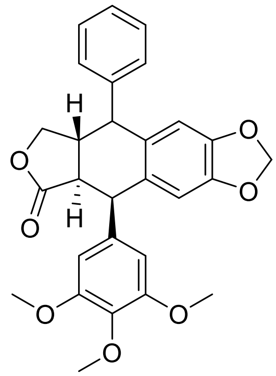

(5*R*,5a*R*,8a*R*,9*R*)-9-Phenyl-5-(3,4,5-trimethoxyphenyl)-5,8,8a,9-tetrahydrofuro[3',4':6,7]naphtho[2,3-d][1,3]dioxol-6(5aH)-one (91). Compound **91** was obtained from (5*R*,5a*R*,8a*R*,9*S*)-9-fluoro-5-(3,4,5-trimethoxyphenyl)-5,8,8a,9-tetrahydrofuro[3',4':6,7]naphtho[2,3-d][1,3]dioxol-6(5aH)-one (104.0 mg, 0.25 mmol) and triphenylaluminum solution (1.0 M in DBE, 0.375 mmol) according to a modified method A. Dichloroethane (0.5 mL) was used as a co-solvent. The reaction was allowed to stir at -40 °C for 18 hours. The product was isolated by column purification using 20% ethyl acetate in hexanes as mobile phase as a white crystalline solid in 80% yield (95.0 mg, 0.20 mmol) with 2:1 *dr*. ^{1}H NMR (400 MHz, chloroform-*d*) δ = 7.37 – 7.29 (m, 3H), 6.89 (m, 2H), 6.52 (s, 1H), 6.47 (s, 1H), 6.36 (m, 2H), 5.91 – 5.87 (m, 2H), 4.67 (d, *J* = 4.9 Hz, 1H), 4.48 (d, *J* = 6.1 Hz, 1H), 4.23 (m, 1H), 3.80 (s, 3H), 3.75 (s, 6H), 3.34 (dd, *J* = 10.7, 8.6 Hz, 1H), 3.10 (m, 1H), 3.02 (dd, *J* = 14.2, 4.9 Hz, 1H); ^{13}C NMR (100 MHz, chloroform-*d*) δ = 174.7, 152.5, 147.2, 140.7, 137.2, 136.0, 131.8, 131.4, 129.6, 129.2, 128.6, 109.9, 108.5, 101.3, 61.0, 56.4, 46.0, 43.9, 41.2, 38.9, 36.6. HRMS (ESI-TOF) m/z: [M+H]+ Calcd for $C_{28}H_{27}O_7$: 475.1757; Found: 475.1755.

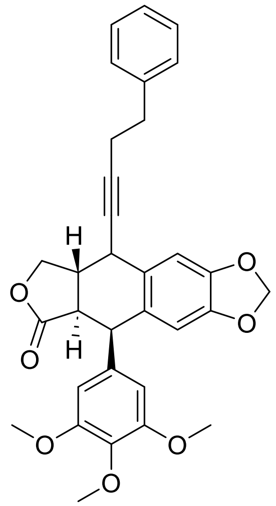

(5*R*,5a*R*,8a*R*)-9-(4-phenylbut-1-yn-1-yl)-5-(3,4,5-trimethoxyphenyl)-5,8,8a,9-tetrahydrofuro[3',4':6,7]naphtho[2,3-d][1,3]dioxol-6(5aH)-one (92). Compound **92** was obtained from (5*R*,5a*R*,8a*R*,9*S*)-9-fluoro-5-(3,4,5-trimethoxyphenyl)-5,8,8a,9-tetrahydrofuro[3',4':6,7]naphtho[2,3-d][1,3]dioxol-6(5aH)-one (104.0 mg, 0.25 mmol) and tris(4-phenylbut-1-yn-1-yl)aluminum (359.0 mM in chlorobenzene, 0.375 mmol.) according to a modified method C. Dichloroethane (0.5 mL) was used as a co-solvent. The reaction was allowed to stir at 25 °C for 18 hours. The product was isolated by column purification using 20% ethyl acetate in hexanes as mobile phase as a brown oil in 73% yield (96.0 mg, 0.18 mmol) with 1.4:1 *dr*. ^{1}H NMR (400 MHz, chloroform-*d*) δ = 7.27 (m, 1H), 7.23 – 7.10 (m, 4H), 6.76 (s, 1H), 6.46 (s, 1H), 6.27 (s, 2H), 5.97 – 5.92 (m, 2H), 4.51 (d, *J* = 5.2 Hz, 1H), 4.23 (m, 1H), 4.05 (dd, *J* = 10.6, 8.6 Hz, 1H), 3.89 (m, 1H), 3.78 (s, 3H), 3.72 (s, 6H), 2.95 (d, *J* = 5.2 Hz, 1H), 2.81 – 2.70 (m, 3H), 2.50 (t, *J* = 7.2, 2H); ^{13}C NMR (100 MHz, chloroform-*d*) δ = 177.4, 155.2, 150.1, 149.9, 142.9, 139.9, 138.6, 132.8, 132.7, 131.1, 131.0, 129.1, 113.0, 111.7, 111.1, 104.0, 88.8, 81.6, 72.3, 63.4, 58.9, 46.4, 45.4, 37.79, 37.4, 36.4, 23.4. HRMS (ESI-TOF) m/z: [M+H]+ Calcd for $C_{32}H_{31}O_7$: 527.2070; Found: 527.2068.

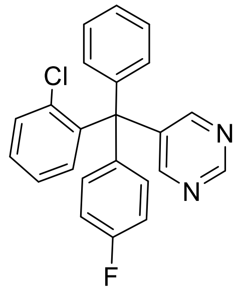

5-((2-Chlorophenyl)(4-fluorophenyl)(phenyl)methyl)pyrimidine (94). Compound **94** was obtained from 5-((2-chlorophenyl)fluoro(4-fluorophenyl)methyl)pyrimidine (79.0 mg, 0.25 mmol) and triphenylaluminum solution (1.0 M in DBE, 0.375 mmol) according to a modified method A. The reaction was allowed to stir at 25 °C for 18 hours. The product was isolated by column purification using 20% ethyl acetate in hexanes as mobile phase as a yellow oil in 51% yield (48.0 mg, 0.13 mmol). ^{1}H NMR (400 MHz, chloroform-*d*) δ = 8.51 (m, 2H), 8.43 (m, 2H), 7.52 – 7.41 (m, 4H), 7.26 – 7.20 (m, 2H), 7.12 – 7.01 (m, 4H), 6.95 (m, 1H), 5.95 (s, 1H); ^{13}C NMR (100 MHz, chloroform-*d*) δ = 163.1, 163.0, 160.6, 157.9, 139.3, 137.1, 136.1 (d, J_{C-F} = 3.3 Hz), 134.3, 133.1, 130.8, 130.7, 130.0 (d, J_{C-F} = 26.9 Hz), 129.7 (d, J_{C-F} = 215.7 Hz), 128.6, 128.0, 127.1, 115.8 (d, J_{C-F} = 21.4 Hz), 48.1. ^{19}F NMR (376 MHz, chloroform-*d*) δ = -115.11. Characterization data are consistent with the literature.[32]

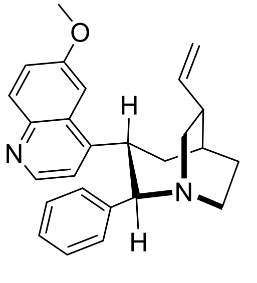

(1*R*,2*R*,3*S*,6*S*)-3-(6-Methoxyquinolin-4-yl)-2-phenyl-6-vinyl-1-azabicyclo[3.2.2]nonane (96). Compound **96** was obtained from from (1*S*,2*S*,4*S*,5*R*)-2-((*S*)-fluoro(6-methoxyquinolin-4-yl)methyl)-5-vinylquinuclidene (82.0 mg, 0.25 mmol) and triphenylaluminum solution (1.0 M in

DBE, 0.375 mmol) according to a modified method A. Dichloroethane (0.5 mL) was used as a co-solvent. The reaction was allowed to stir at 25 °C for 18 hours. The product was isolated by column purification using 3% methanol in dichloromethane as mobile phase as an amporphous solid in 80% yield (77.0 mg, 0.20 mmol). ^{1}H NMR (400 MHz, chloroform-*d*) δ = 8.55 (d, *J* = 4.6 Hz, 1H), 7.99 (d, *J* = 9.2 Hz, 1H), 7.50 (m, 1H), 7.38 (m, 1H), 7.24 – 7.19 (m, 2H), 7.14 (d, *J* = 4.7 Hz, 1H), 7.10 – 7.01 (m, 3H), 6.00 – 5.87 (m, 1H), 5.15 – 5.06 (m, 2H), 4.41 (d, *J* = 11.6 Hz, 1H), 4.30 (dd, *J* = 13.5, 9.4 Hz, 1H), 4.03 (s, 3H), 3.77 (dd, *J* = 14.5, 9.4 Hz, 1H), 3.24 (dd, *J* = 14.3, 8.7 Hz, 1H), 3.14 (m, 1H), 2.79 (m, 1H), 2.51 (m, 1H), 2.27 – 2.14 (m, 2H), 1.92 – 1.82 (m, 2H), 1.29 (m, 1H); ^{13}C NMR (100 MHz, chloroform-*d*) δ = 157.9, 148.5, 147.9, 144.6, 141.0, 132.1, 128.2, 127.7, 127.5, 126.9, 120.4, 118.7, 114.8, 101.6, 74.6, 57.7, 55.6, 43.7, 39.1, 38.2, 34.7, 32.9, 29.4, 28.5. HRMS (ESI-TOF) *m/z*: $[M+H]^+$ Calcd for $C_{26}H_{29}N_2O$ 385.2274; Found 385.2273.

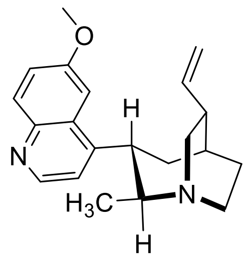

(1*R*,2*S*,3*S*,6*S*)-3-(6-Methoxyquinolin-4-yl)-2-methyl-6-vinyl-1-azabicyclo[3.2.2]nonane (97). Compound **97** was obtained from from (1*S*,2*S*,4*S*,5*R*)-2-((*S*)-fluoro(6-methoxyquinolin-4-yl)methyl)-5-vinylquinuclidene (82.0 mg, 0.25 mmol) and trimethylaluminum solution (2.0 M in hexanes, 0.375 mmol) according to a modified method A. Dichloroethane (0.5 mL) was used as a co-solvent. The reaction was allowed to stir at 25 °C for 18 hours. The product was isolated by column purification using 3% methanol in dichloromethane as mobile phase as an amporphous solid in 76% yield (61.0 mg, 0.19 mmol). ^{1}H NMR (400 MHz, chloroform-*d*) δ = 8.71 (d, *J* = 4.6 Hz, 1H), 8.03 (d, *J* = 9.0 Hz, 1H), 7.43 – 7.33 (m, 2H), 7.21 (d, *J* = 4.6 Hz, 1H), 5.90 (m, 1H), 5.16

– 5.04 (m, 2H), 3.96 (s, 3H), 3.67 (m, 1H), 3.49 (m, 1H), 3.30 (m, 2H), 2.97 (m, 2H), 2.49 (m, 1H), 2.16 (m, 1H), 2.03 (m, 1H), 1.88 – 1.77 (m, 2H), 1.64 (s, 1H), 0.93 (d, J = 6.4 Hz, 3H); ^{13}C NMR (100 MHz, chloroform-*d*) δ = 157.8, 149.2, 148.0, 144.7, 140.7, 132.1, 128.2, 120.7, 118.6, 115.1, 101.6, 68.8, 56.6, 55.4, 42.9, 42.4, 38.8, 34.2, 32.5, 27.6, 19.7. HRMS (ESI-TOF) m/z: [M+H]+ Calcd for $C_{21}H_{27}N_2O$: 323.2123; Found: 323.2120.

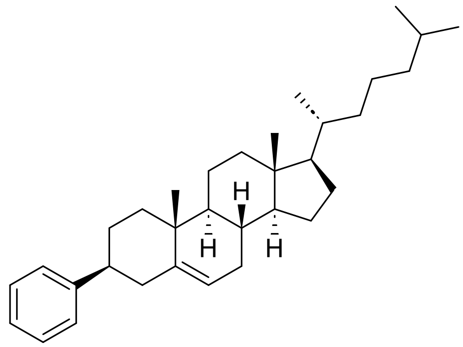

(3*S*,8*S*,9*S*,10*R*,13*R*,14*S*,17*R*)-10,13-Dimethyl-17-((*R*)-6-methylheptan-2-yl)-3-phenyl-2,3,4,7,8,9,10,11,12,13,14,15,16,17-tetradecahydro-1H-cyclopenta[a]phenanthrene (99). Compound **99** was obtained from 3β-fluorocholestane (97.0 mg, 0.25 mmol) and triphenylaluminum solution (1.0 M in DBE, 0.375 mmol) according to a modified method A. Dichloroethane (0.5 mL) was used as a co-solvent. The reaction was allowed to stir at 25 °C for 18 hours. The product was isolated by column purification using 100% hexanes as mobile phase as a white crystalline solid in 91% yield (101.0 mg, 0.23 mmol). ^{1}H NMR (400 MHz, chloroform-*d*) δ = 7.33 – 7.13 (m, 5H), 5.34 (m, 1H), 2.48 (m, 2H), 2.17 (m, 1H), 2.07 – 1.91 (m, 3H), 1.88 – 1.70 (m, 3H), 1.63 – 0.96 (m, 23H), 0.92 (d, J = 6.4 Hz, 3H), 0.87 – 0.83 (d, J = 6.6, 6H), 0.69 (s, 3H); ^{13}C NMR (100 MHz, chloroform-*d*) δ = 147.1, 143.0, 128.2, 126.8, 126.1, 120.1, 56.8, 56.1, 50.4, 45.9, 42.3, 38.8, 36.9, 35.8, 31.9, 30.0, 28.0, 24.3, 23.8, 22.9, 22.7, 22.6, 22.5, 21.0, 19.7, 19.5, 18.8, 18.7, 11.9, 11.8. Characterization data are consistent with the literature.[33]

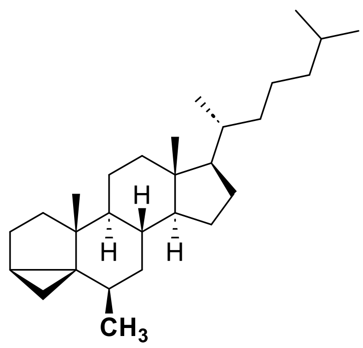

(1a*R*,3a*R*,3b*S*,5a*R*,6*R*,8a*S*,8b*S*,10*R*,10a*R*)-3a,5a,10-Trimethyl-6-((*R*)-6-methylheptan-2-yl)hexadecahydrocyclopenta[a]cyclopropa[2,3]cyclopenta[1,2-f]naphthalene (100). Compound **100** was obtained from 3β-fluorocholestane (97.0 mg, 0.25 mmol) and trimethylaluminum solution (2.0 M hexanes, 0.375 mmol) according to the general procedure for alkylation. Dichloroethane (0.5 mL) was used as a co-solvent. The reaction was allowed to stir at 25 °C for 18 hours. The product was isolated by column purification using 100% hexanes as mobile phase as a white crystalline solid in 93% yield (90.0 mg, 0.23 mmol). ^{1}H NMR (400 MHz, chloroform-*d*) δ = 1.96 (m, 1H), 1.86 – 1.65 (m, 2H), 1.61 – 0.81 (m, 40H), 0.69 (s, 3H), 0.36 (m, 1H), 0.02 (dd, *J* = 7.8, 4.8 Hz, 1H); ^{13}C NMR (100 MHz, chloroform-*d*) δ = 56.6, 56.4, 48.1, 42.9, 42.7, 40.3, 39.5, 37.7, 36.7, 36.1, 35.8, 35.1, 33.8, 30.4, 28.3, 28.0, 26.8, 26.7, 25.3, 24.4, 23.8, 22.7, 20.5, 19.1, 18.7, 12.2, 11.9. Characterization data are consistent with the literature.[34]

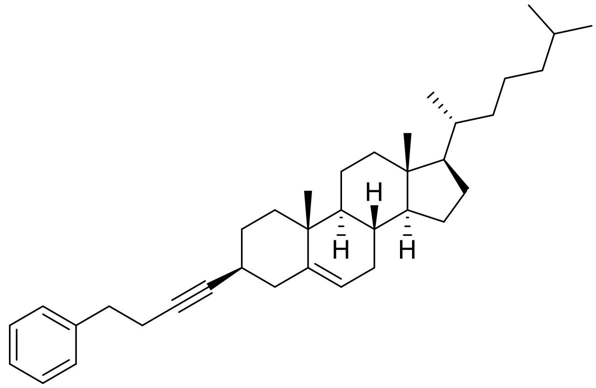

(3*S*,8*S*,9*S*,10*R*,13*R*,14*S*,17*R*)-10,13-Dimethyl-17-((*R*)-6-methylheptan-2-yl)-3-(4-phenylbut-1-yn-1-yl)-2,3,4,7,8,9,10,11,12,13,14,15,16,17-tetradecahydro-1H-cyclopenta[a]phenanthrene (101). Compound **101** was obtained from 3β-fluorocholestane (97.0 mg, 0.25 mmol) and tris(4-phenylbut-1-yn-1-yl)aluminum (359.0 mM in chlorobenzene, 0.375 mmol.) according to a modified method C. Dichloroethane (0.5 mL) was used as a co-solvent. The reaction was allowed to stir at 25 °C for 18 hours. The product was isolated by column purification using 100% hexanes as mobile phase as a white crystalline solid in 61% yield (76.0 mg, 0.15 mmol). ^{1}H NMR (400 MHz, chloroform-*d*) δ = 7.29 – 7.15 (m, 5H), 5.29 (d, 1H), 2.78 (t, *J* = 7.6 Hz, 2H), 2.43 (t, *J* = 7.6 Hz, 2H), 2.32 – 2.07 (m, 3H), 1.98 (m, 2H), 1.87 – 1.72 (m, 4H), 1.61 – 0.80 (m, 32H), 0.66 (s, 3H); ^{13}C NMR (100 MHz, chloroform-*d*) δ = 141.7, 141.0, 128.6, 128.4, 120.6, 120.4, 85.2, 79.4, 56.5, 55.6, 50.2, 42.3, 39.5, 38.9, 36.8, 35.8, 35.2, 35.2, 32.7, 30.0, 28.0, 24.0, 24.0, 22.9, 22.5, 21.1, 19.8, 19.3, 19.1, 18.4, 14.8, 13.6, 11.9, 11.3. HRMS (ESI-TOF) m/z: [M+H]+ Calcd for $C_{37}H_{55}$: 499.4304; Found: 499.4289.

10. ^{1}H, ^{13}C and ^{19}F NMR Spectra

Figure S60. ^{1}H NMR spectrum of 1,3-diphenylbutane (5).

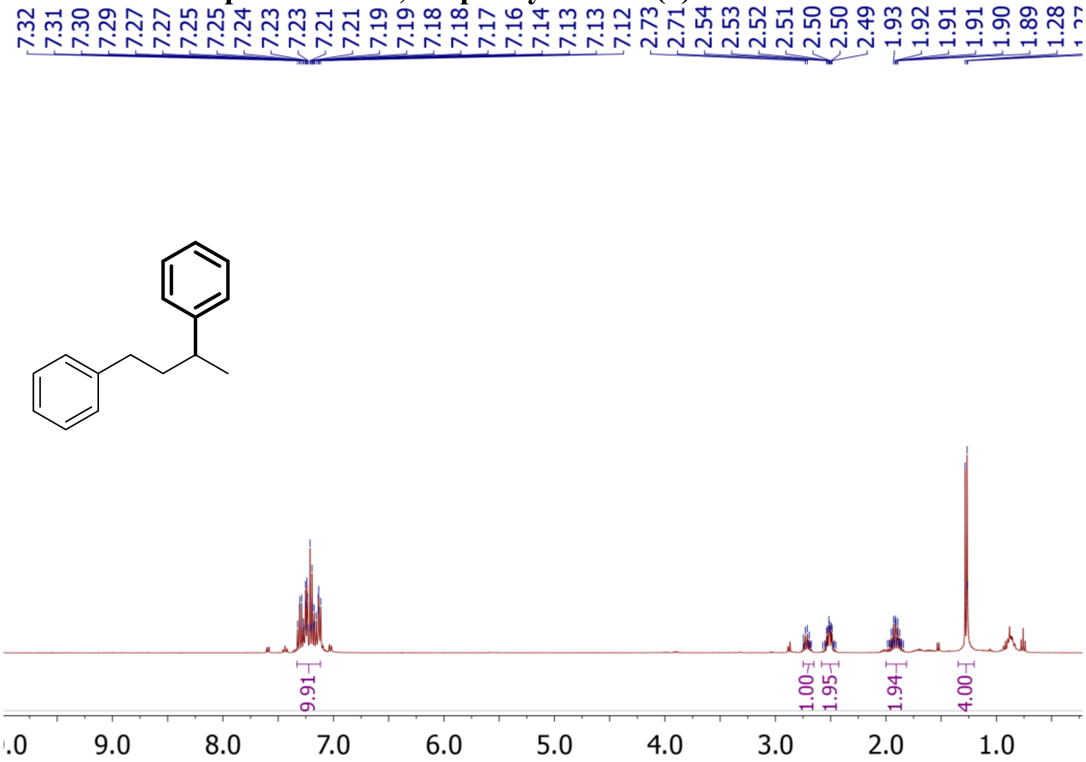

Figure S61. ^{13}C NMR spectrum of 1,3-diphenylbutane (5).

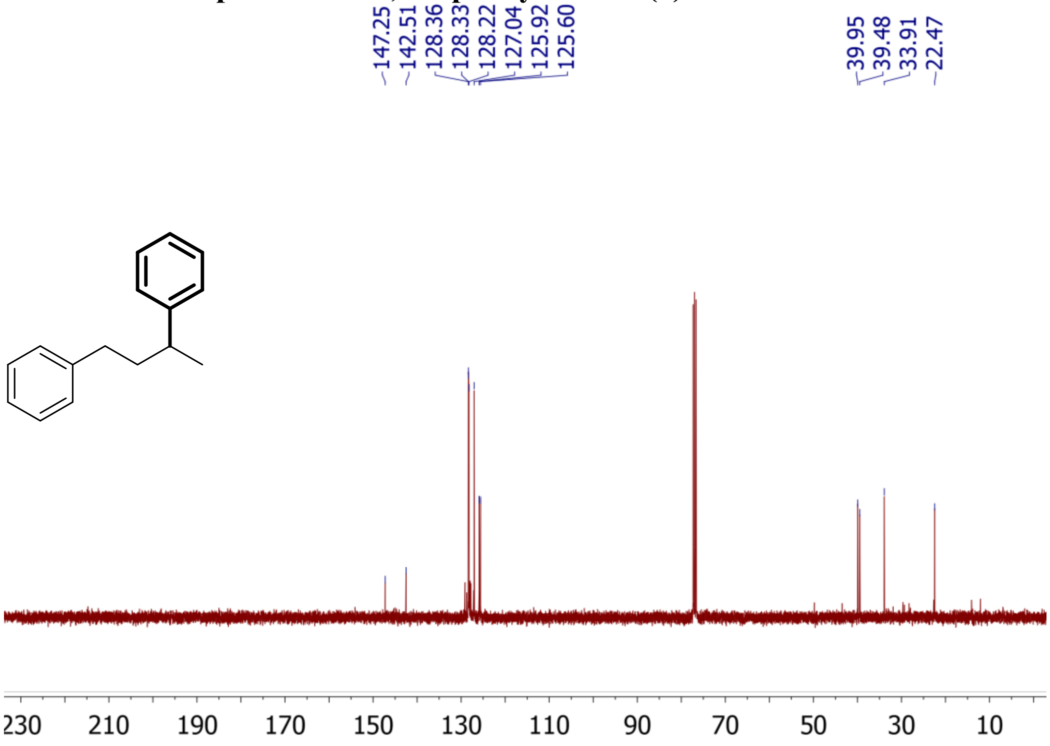

Figure S62. ^{1}H **NMR spectrum of 1-phenyladamantane (6).**

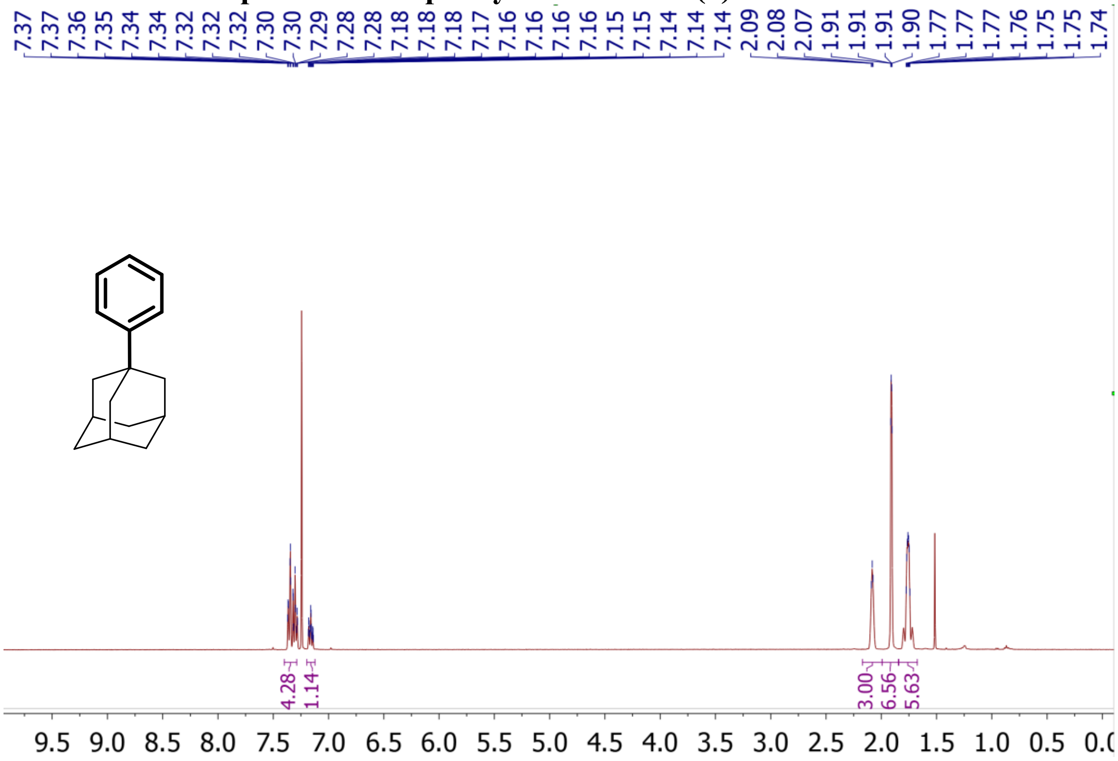

Figure S63. ^{13}C **NMR spectrum of 1-phenyladamantane (6).**

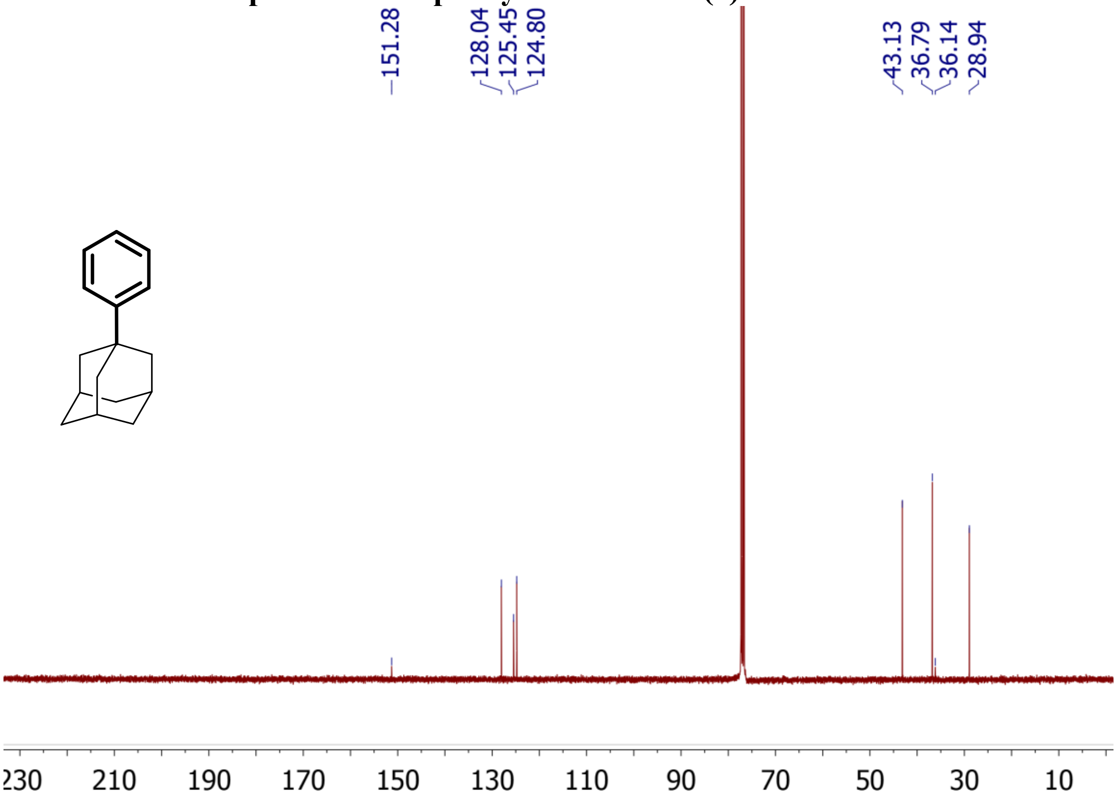

Figure S64. ^{1}**H NMR spectrum of (5-bromopentyl)benzene (8).**

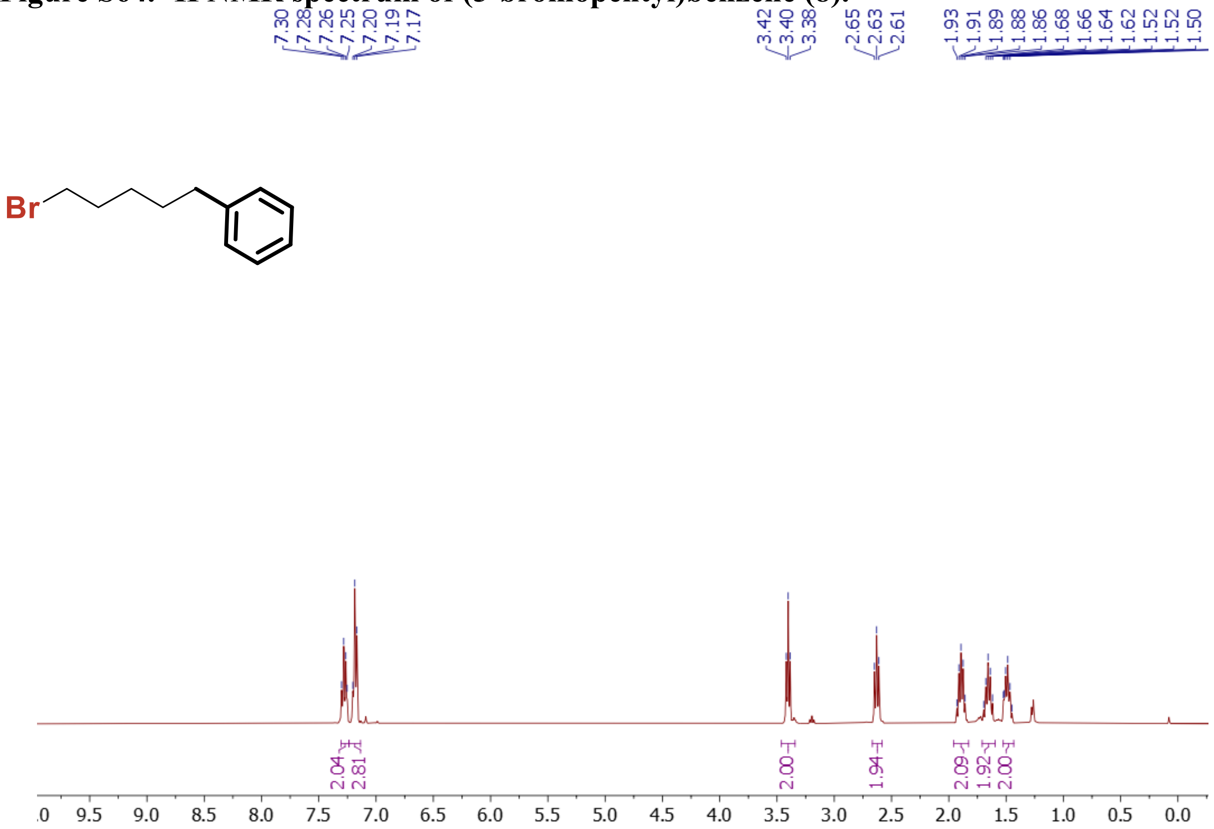

Figure S65. ^{13}**C NMR spectrum of (5-bromopentyl)benzene (8).**

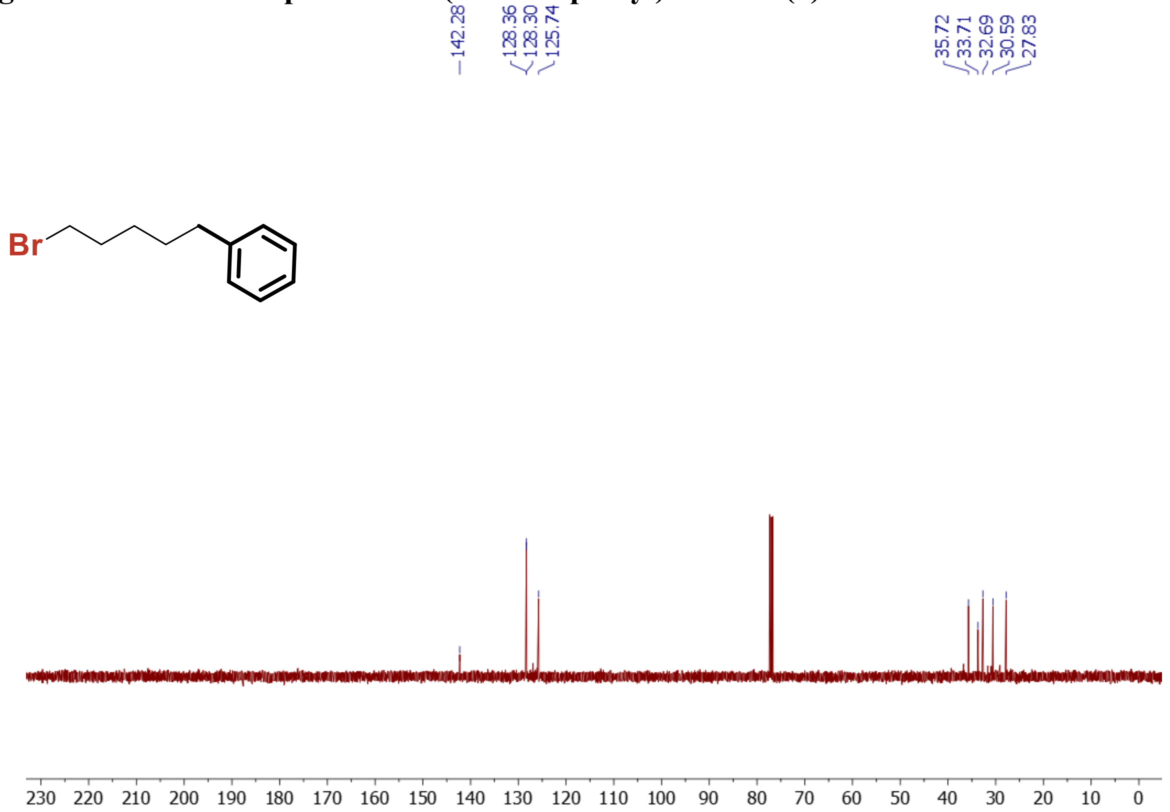

Figure S66. ^{1}**H NMR spectrum of 2-(3-iodopropyl)-1,3,5-trimethylbenzene (11).**

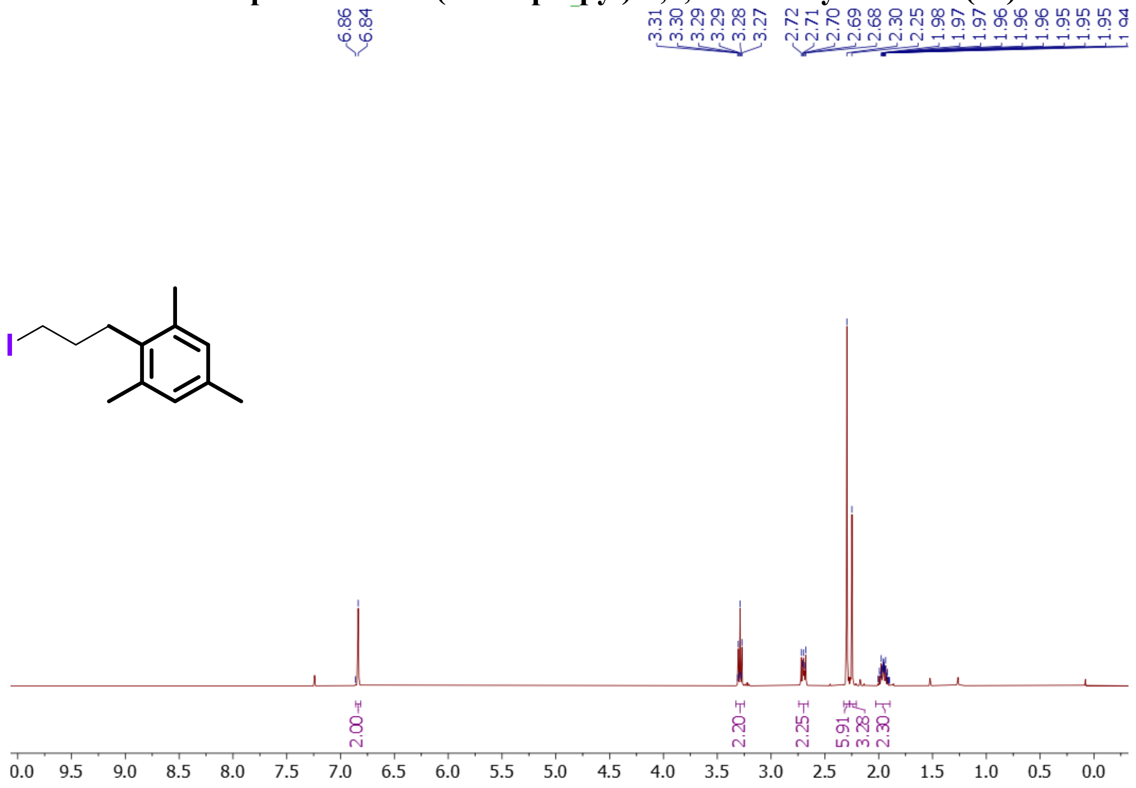

Figure S67. ^{13}**C NMR spectrum of 2-(3-iodopropyl)-1,3,5-trimethylbenzene (11).**

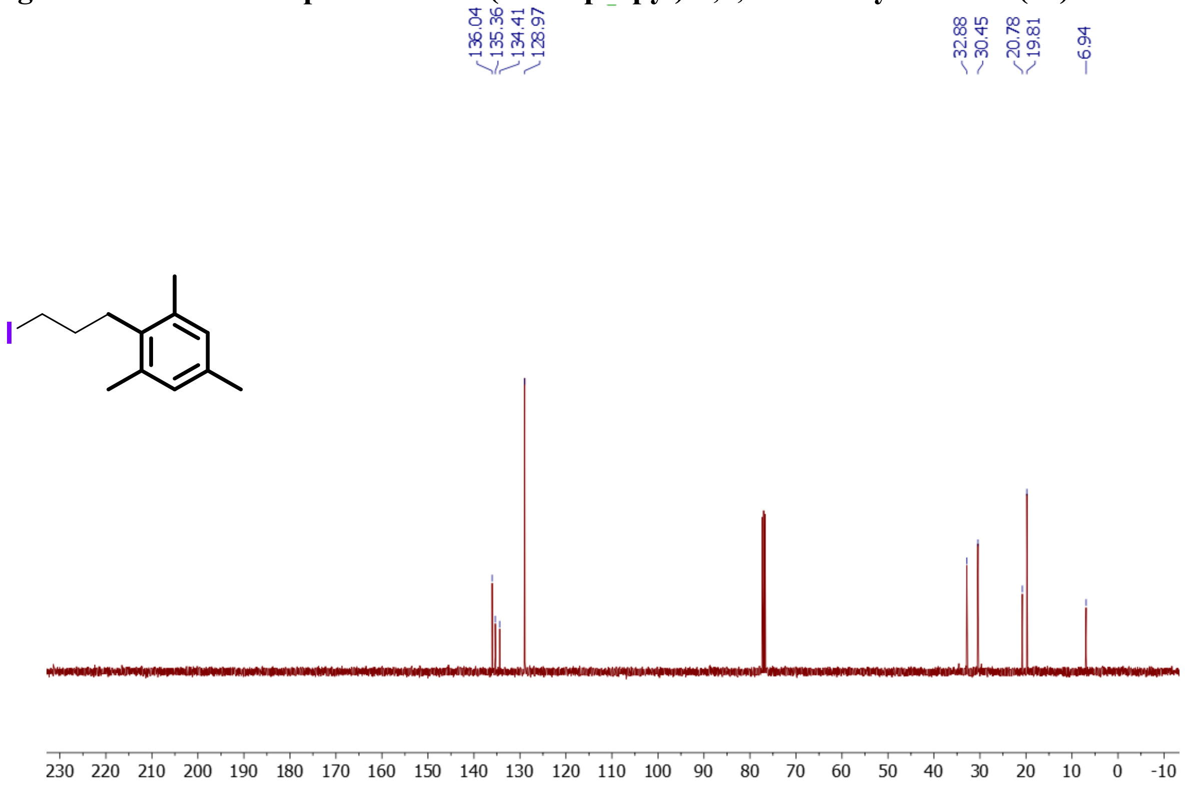

Figure S68. ^{1}H NMR spectrum of cyclohexylbenzene (19).

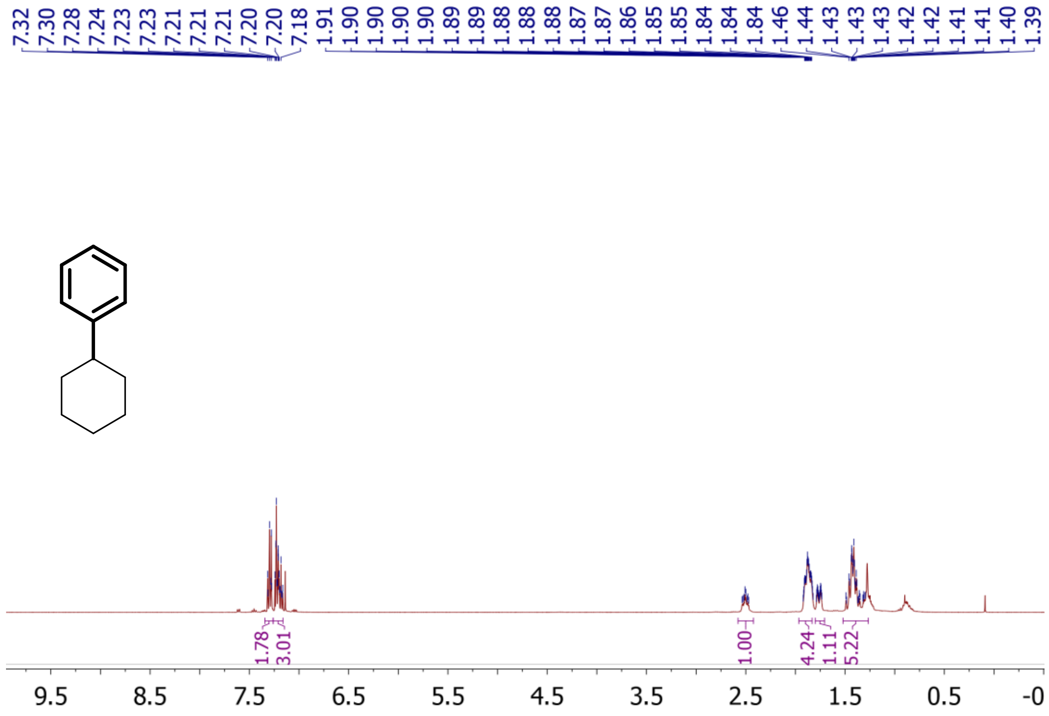

Figure S69. ^{13}C NMR spectrum of cyclohexylbenzene (19).

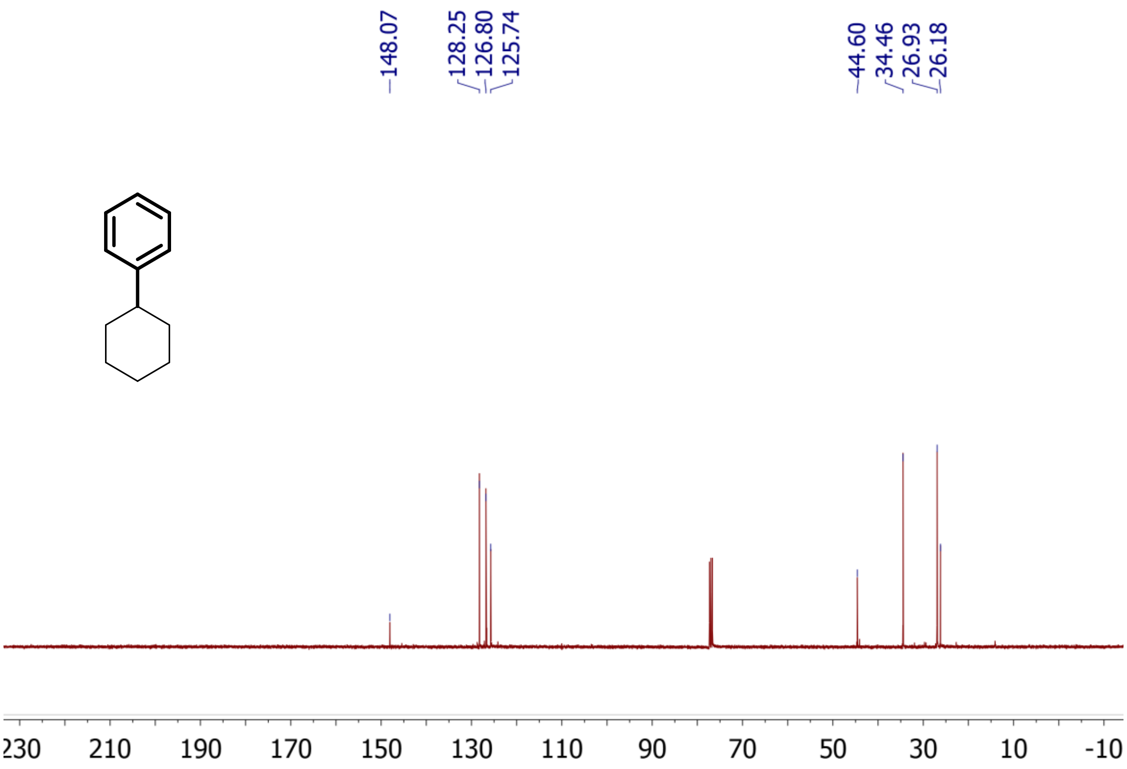

Figure S70. ^{1}H NMR spectrum of (6-chlorohexyl)benzene (39).

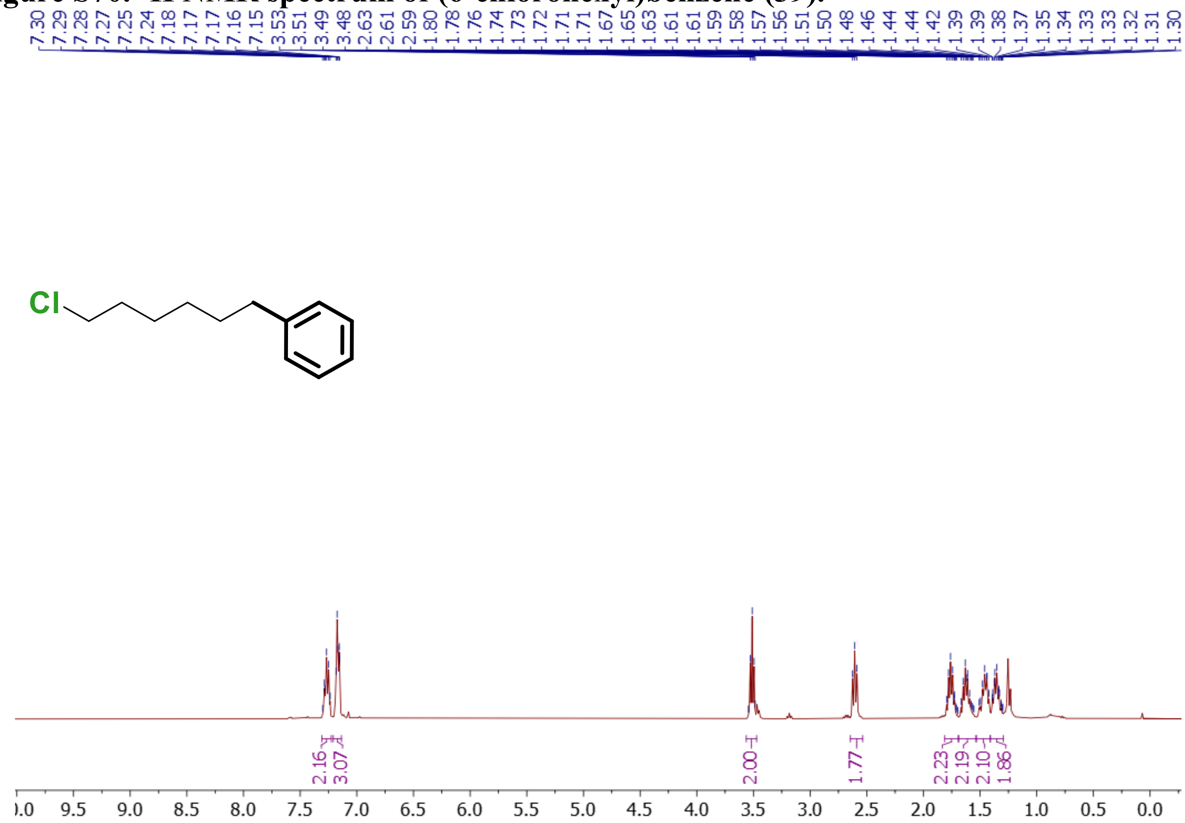

Figure S71. ^{13}C NMR spectrum of (6-chlorohexyl)benzene (39).

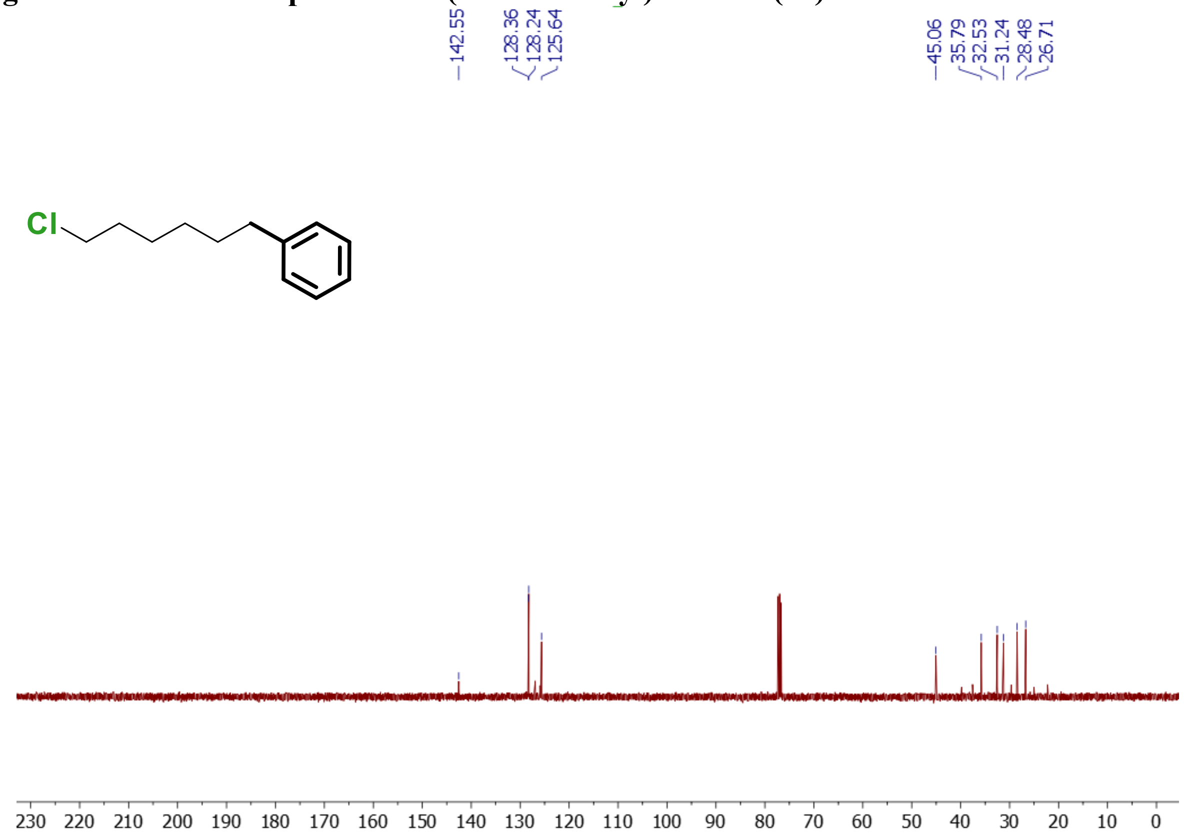

Figure S72. ^{1}H NMR spectrum of 2-(6-chlorohexyl)-1,3,5-trimethylbenzene (40).

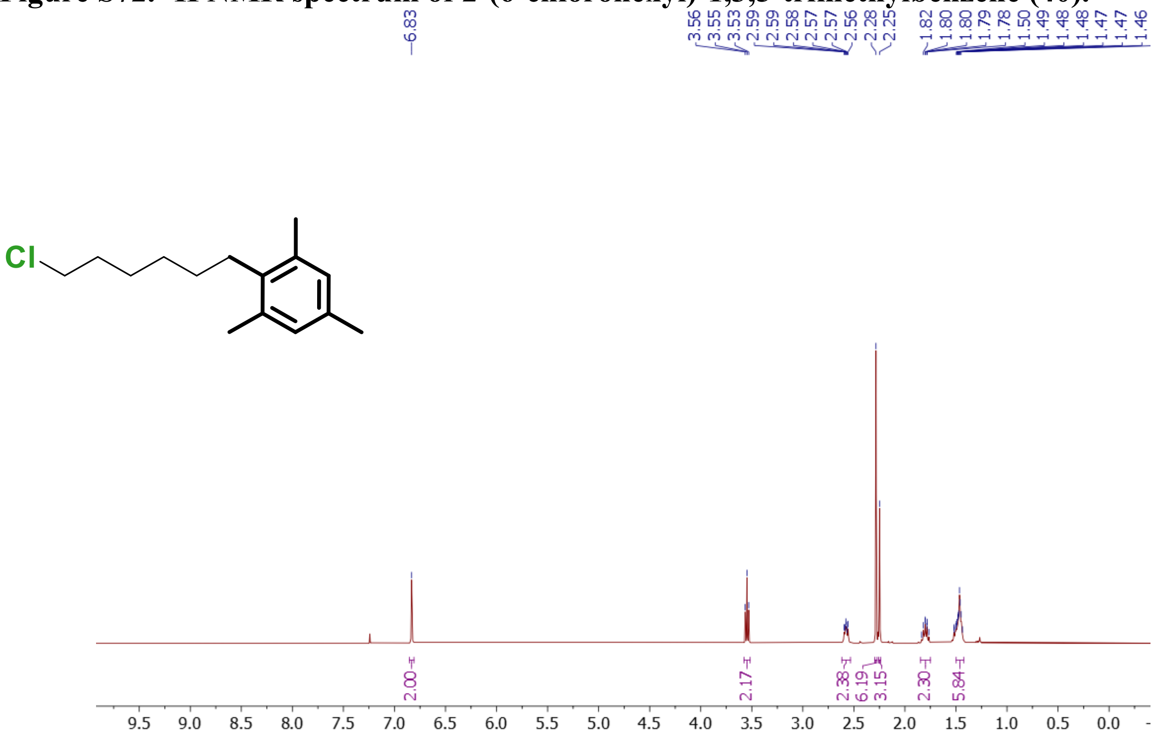

Figure S73. ^{13}C NMR spectrum of 2-(6-chlorohexyl)-1,3,5-trimethylbenzene (40).

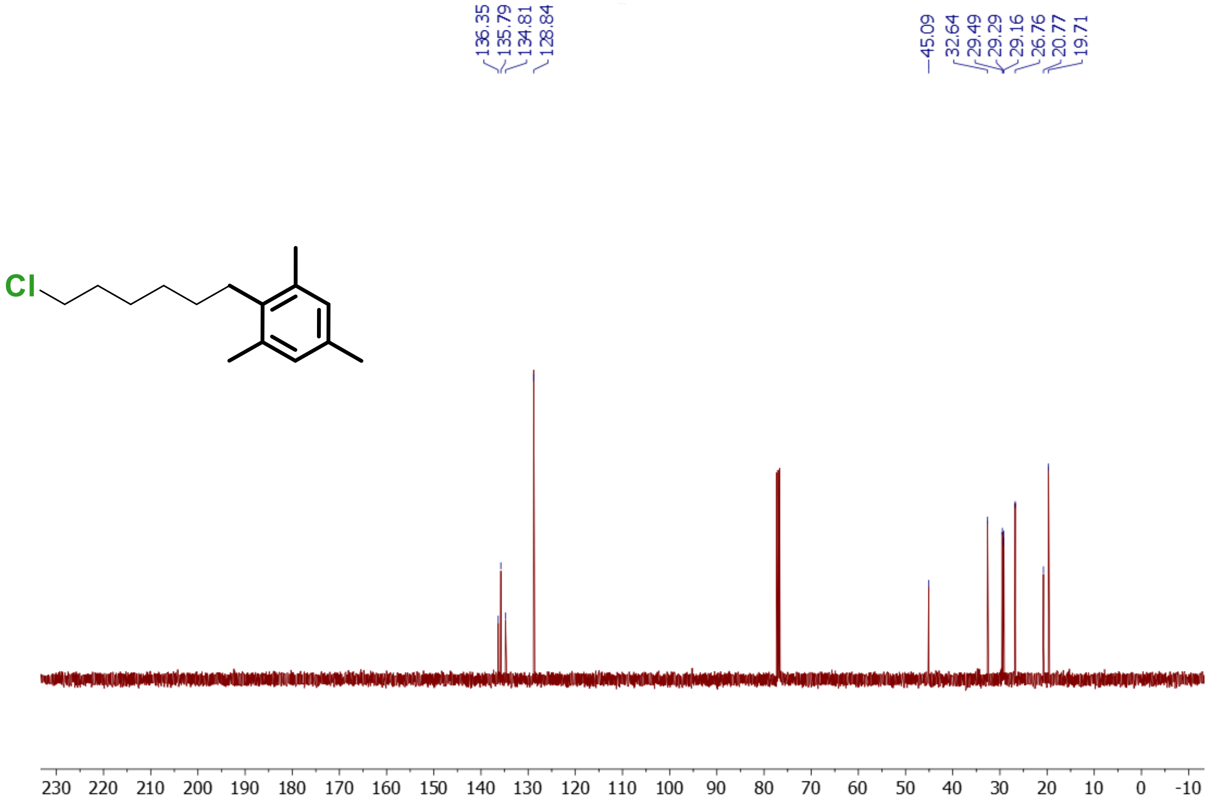

Figure S74. ^{1}**H NMR spectrum of 1-chloro-6-iodohexane (41).**

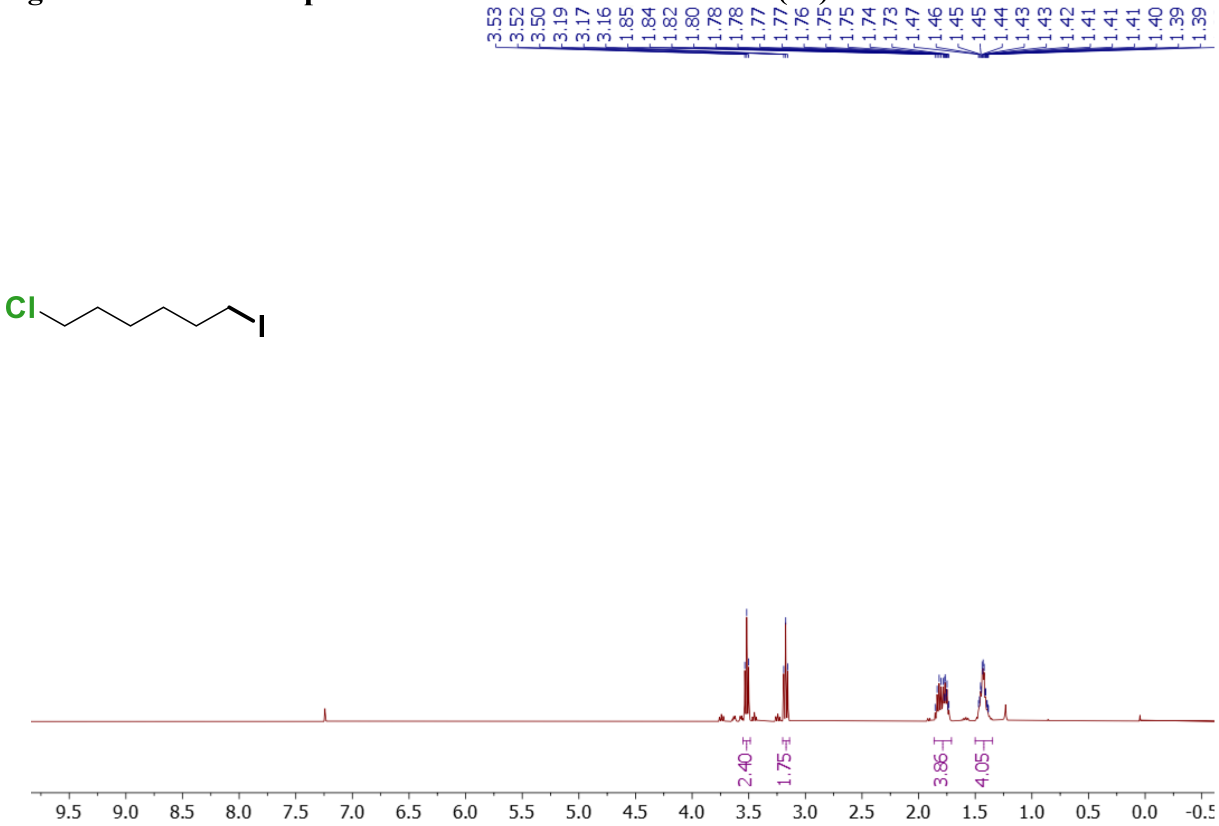

Figure S75. ^{13}**C NMR spectrum of 1-chloro-6-iodohexane (41).**

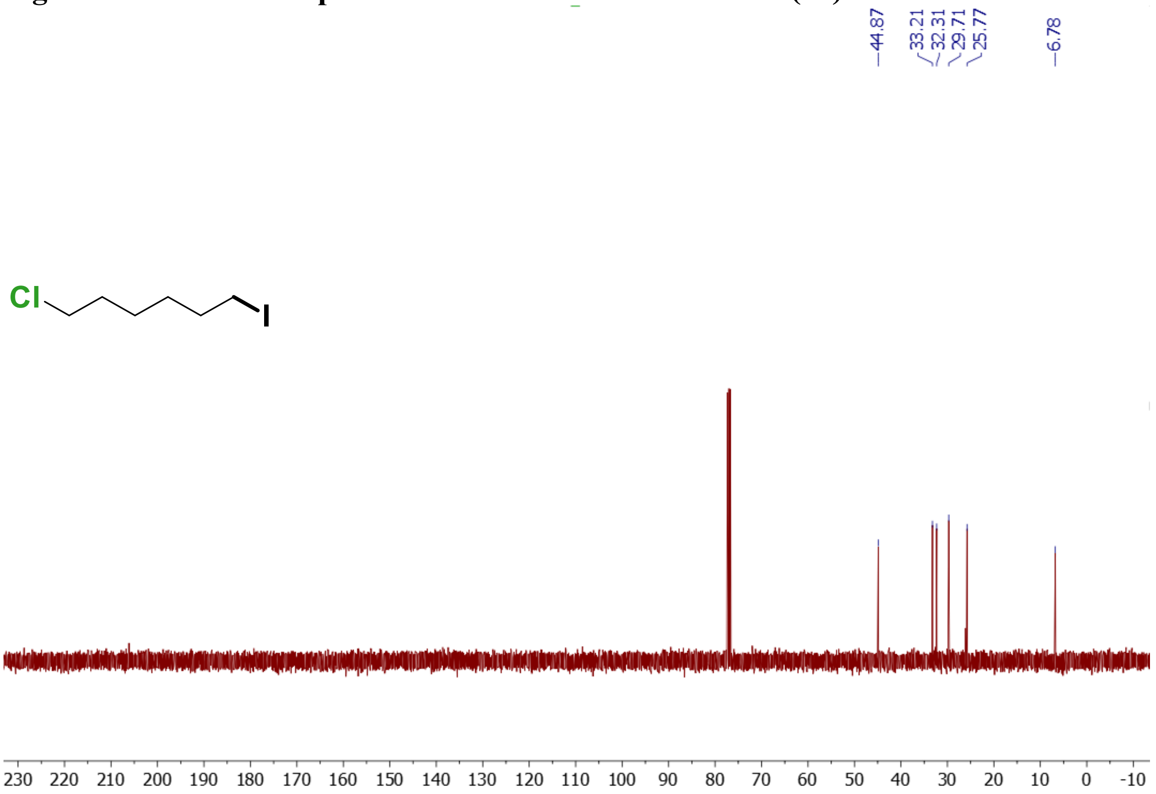

Figure S76. ^{1}H NMR spectrum of (5-bromopentyl)benzene (42).

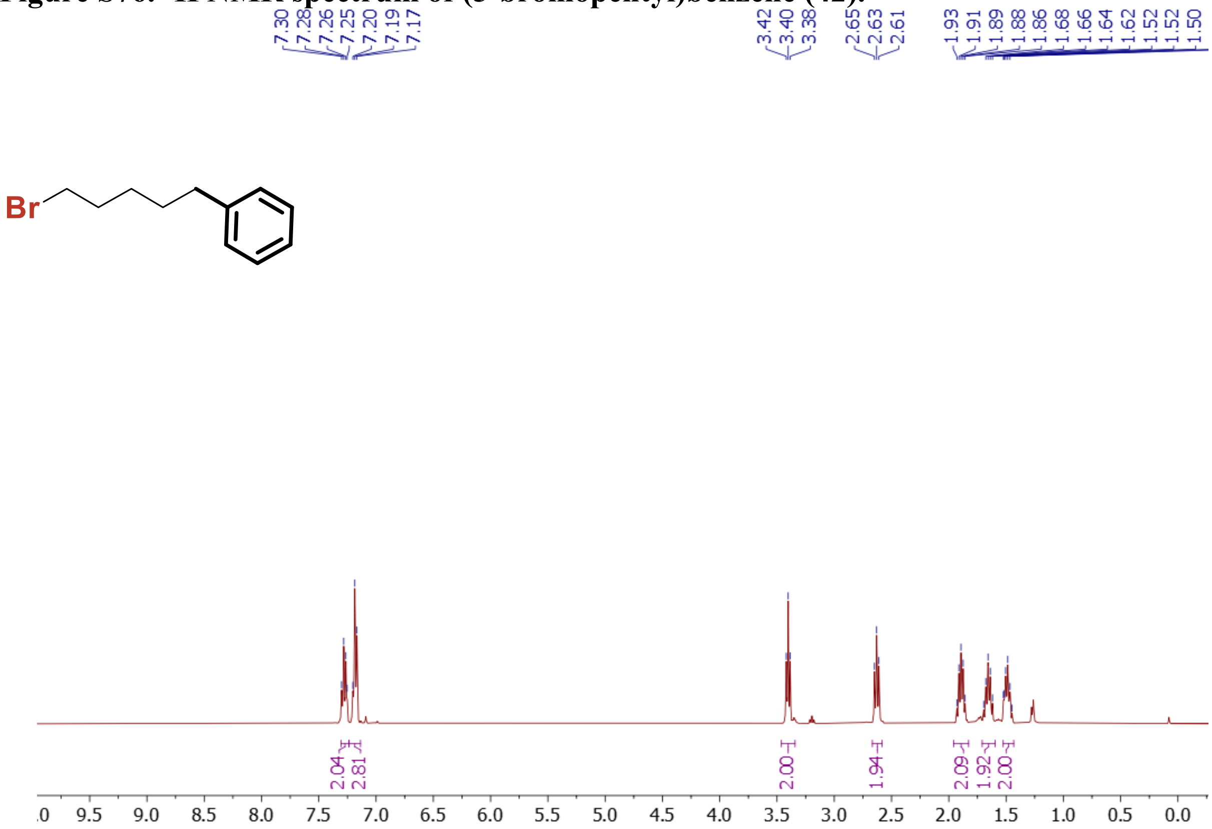

Figure S77. ^{13}C NMR spectrum of (5-bromopentyl)benzene (42).

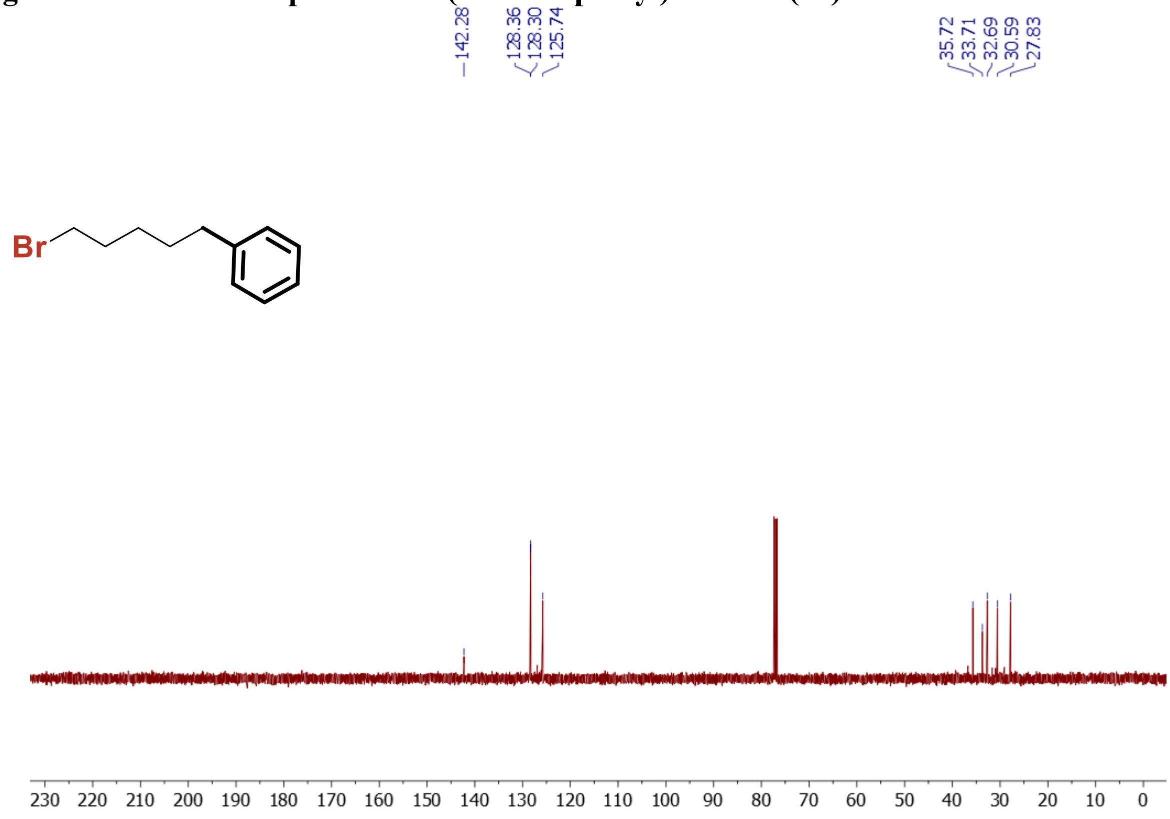

Figure S78. ^{1}H **NMR spectrum of 2-(5-bromopentyl)-1,3,5-trimethylbenzene (43).**

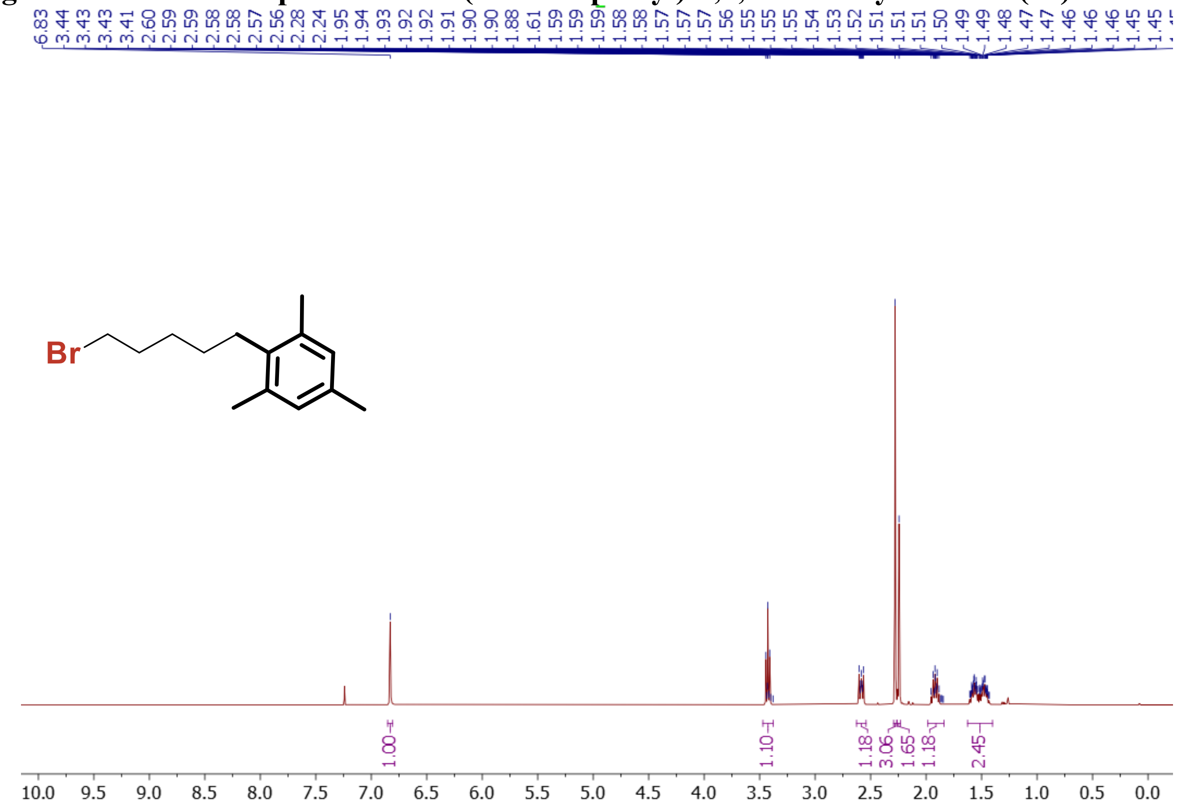

Figure S79. ^{13}C **NMR spectrum of 2-(5-bromopentyl)-1,3,5-trimethylbenzene (43).**

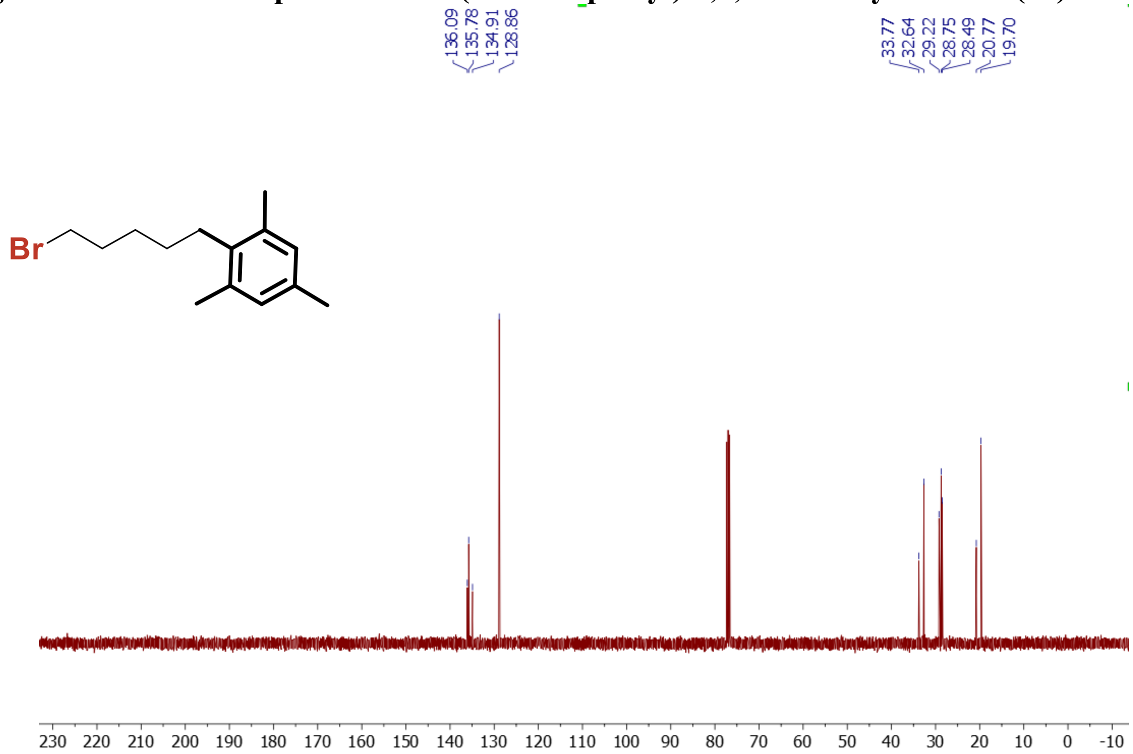

Figure S80. ^{1}H **NMR spectrum of 1-bromo-5-iodopentane (44).**

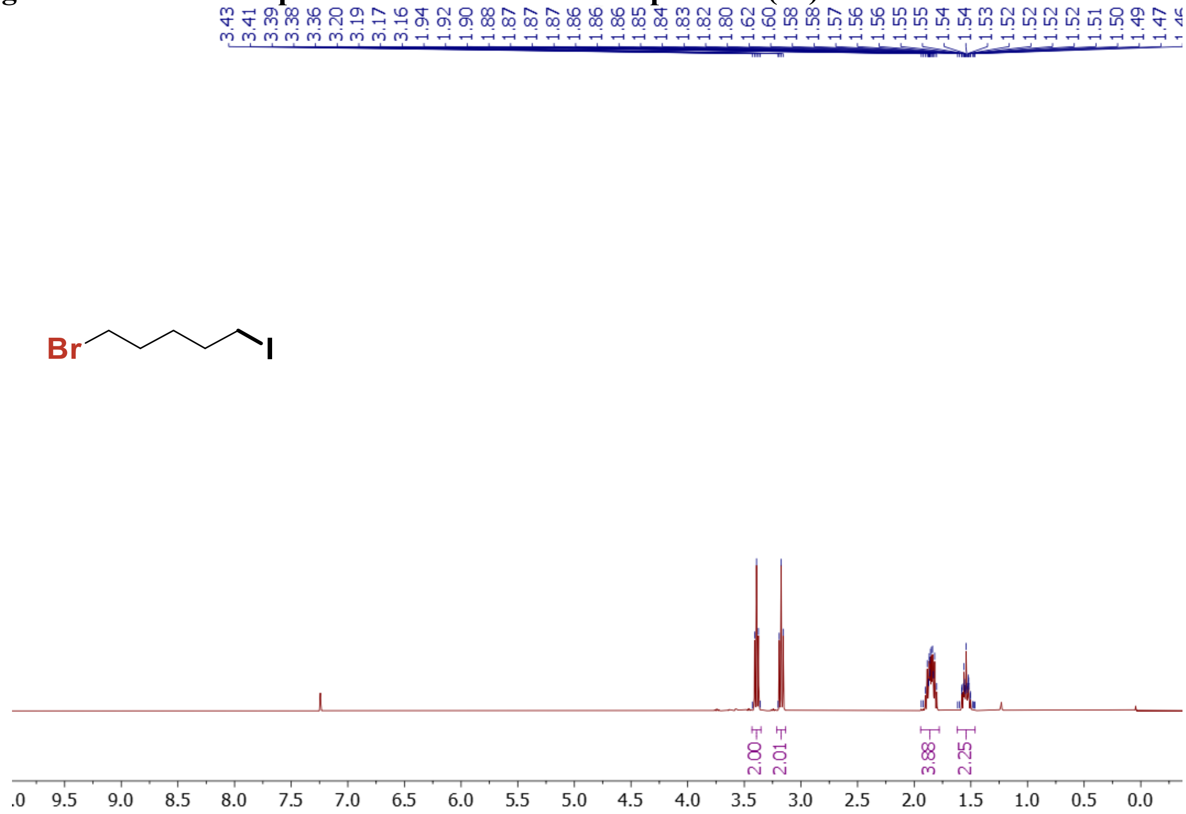

Figure S81. ^{13}C **NMR spectrum of 1-bromo-5-iodopentane (44).**

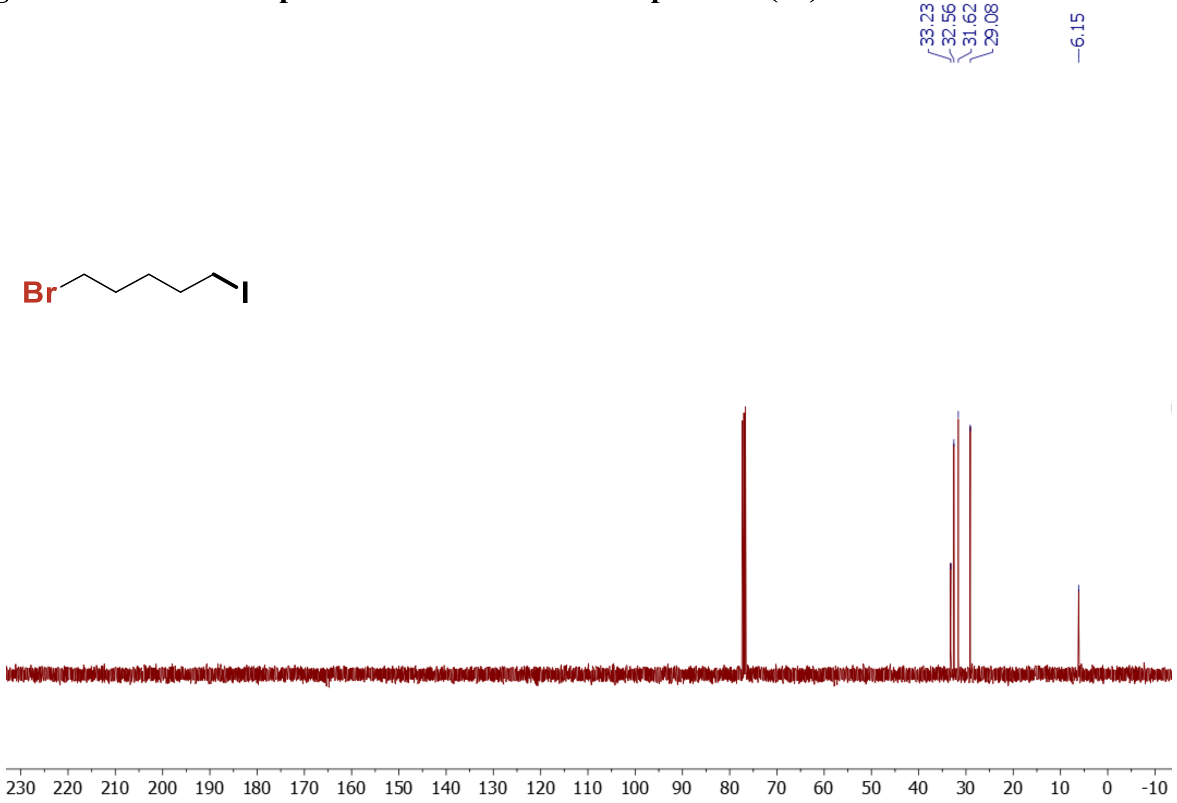

Figure S82. ^{1}H NMR spectrum of (*trans*-2-bromocyclohexyl)benzene (46).

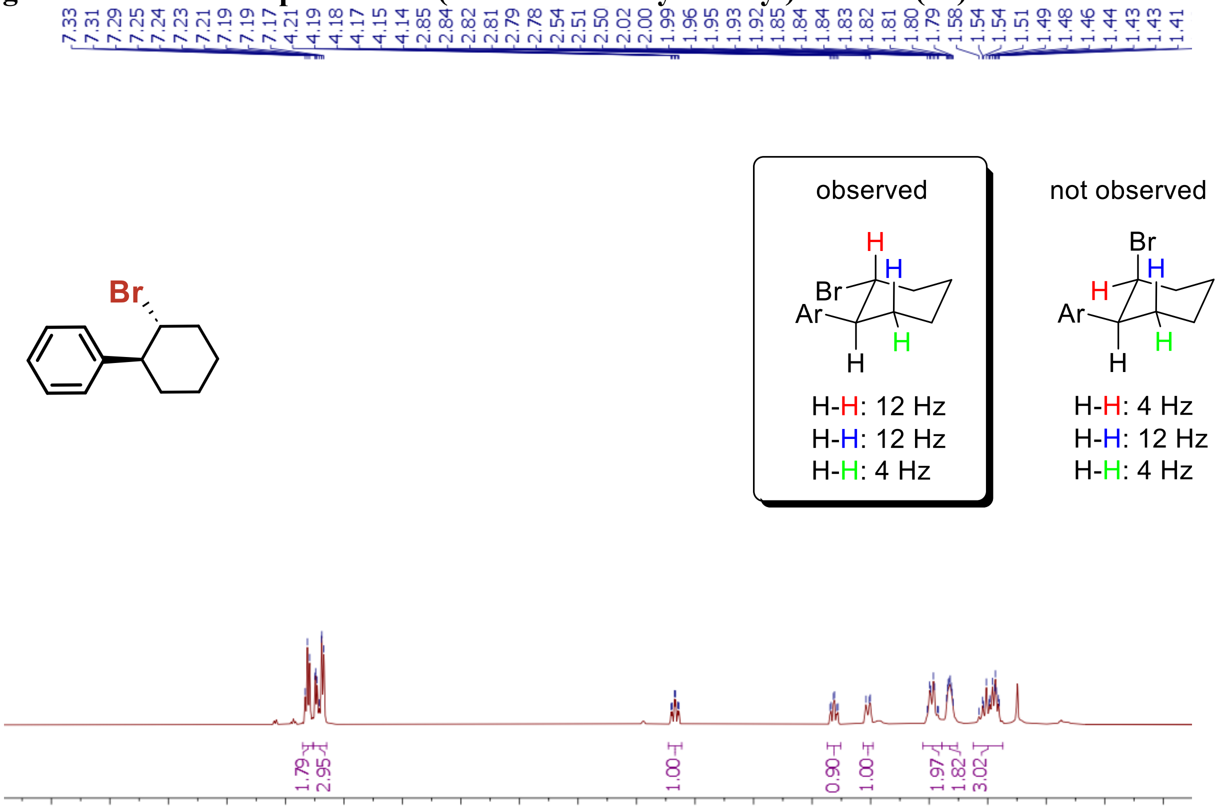

Figure S83. ^{13}C NMR spectrum of (*trans*-2-bromocyclohexyl)benzene (46).

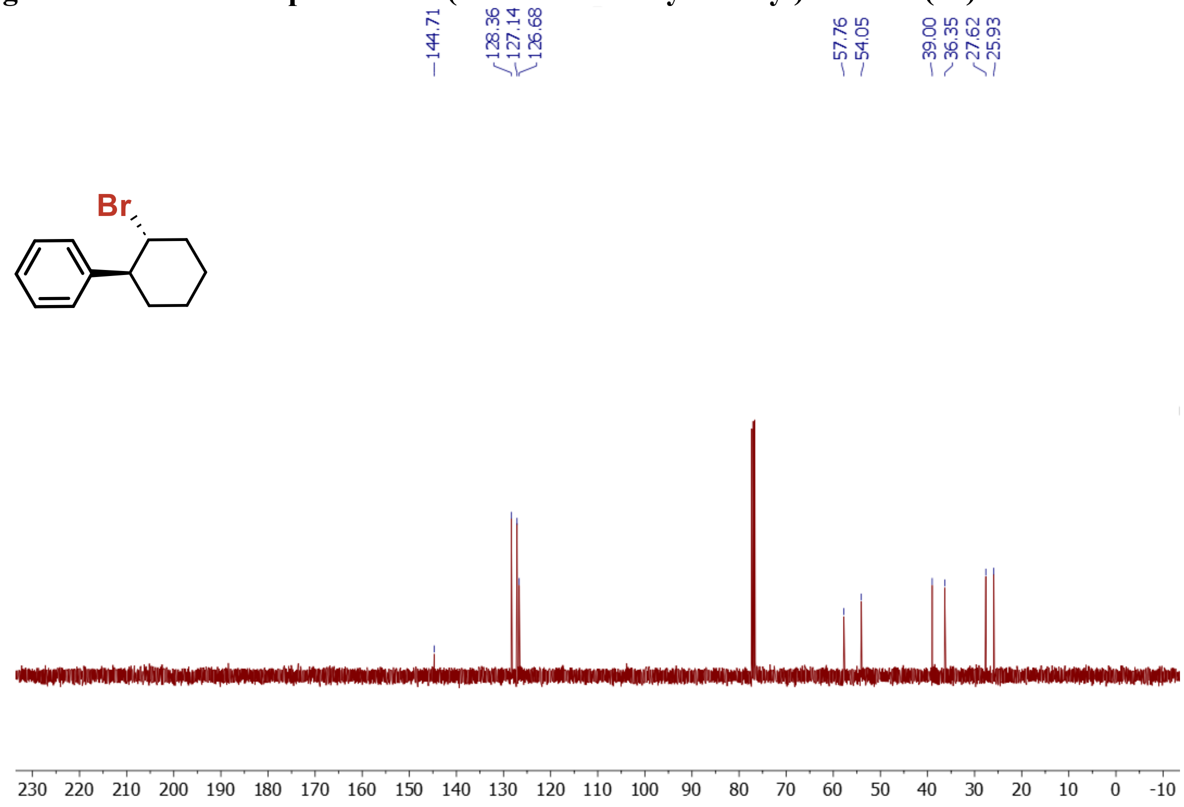

Figure S84. **^{1}H NMR spectrum of 2-(*trans*-2-bromocyclohexyl)-1,3,5-trimethylbenzene (47).**

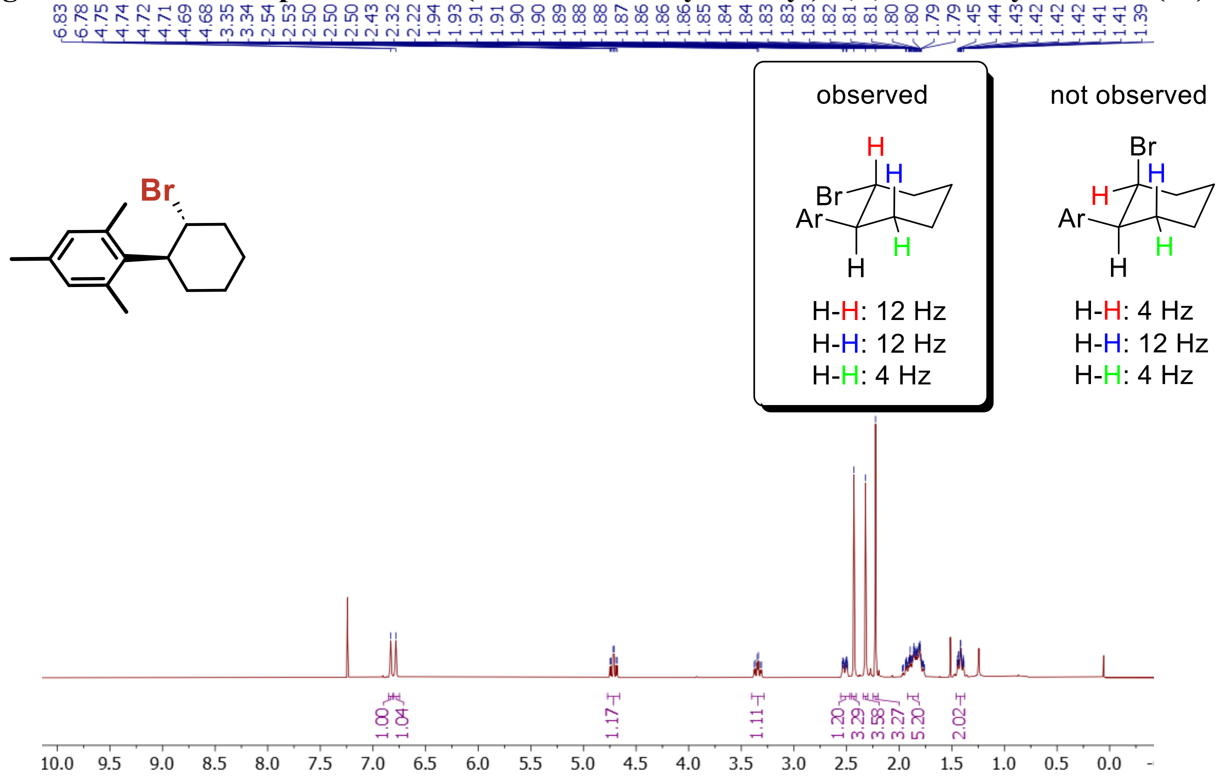

Figure S85. **^{13}C NMR spectrum of 2-(*trans*-2-bromocyclohexyl)-1,3,5-trimethylbenzene (47).**

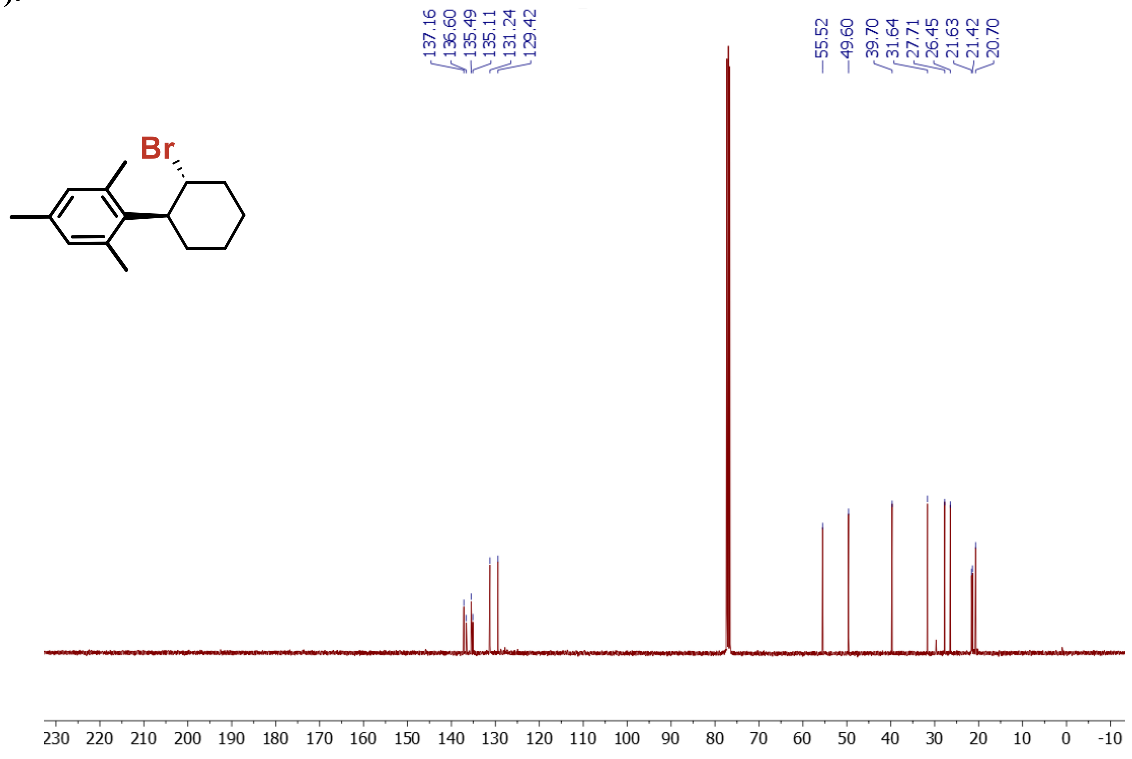

Figure S86. ^{1}H NMR spectrum of (1-bromo-2-methylpropan-2-yl)benzene (49).

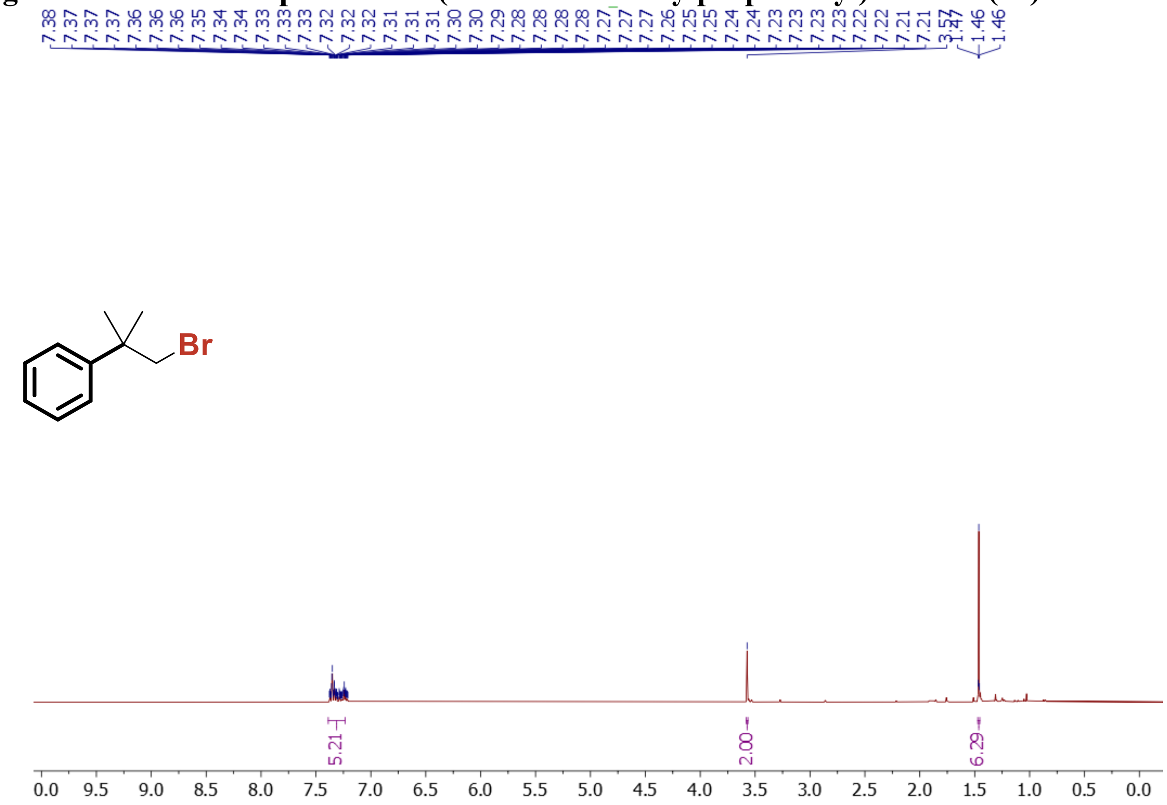

Figure S87. ^{13}C NMR spectrum of (1-bromo-2-methylpropan-2-yl)benzene (49).

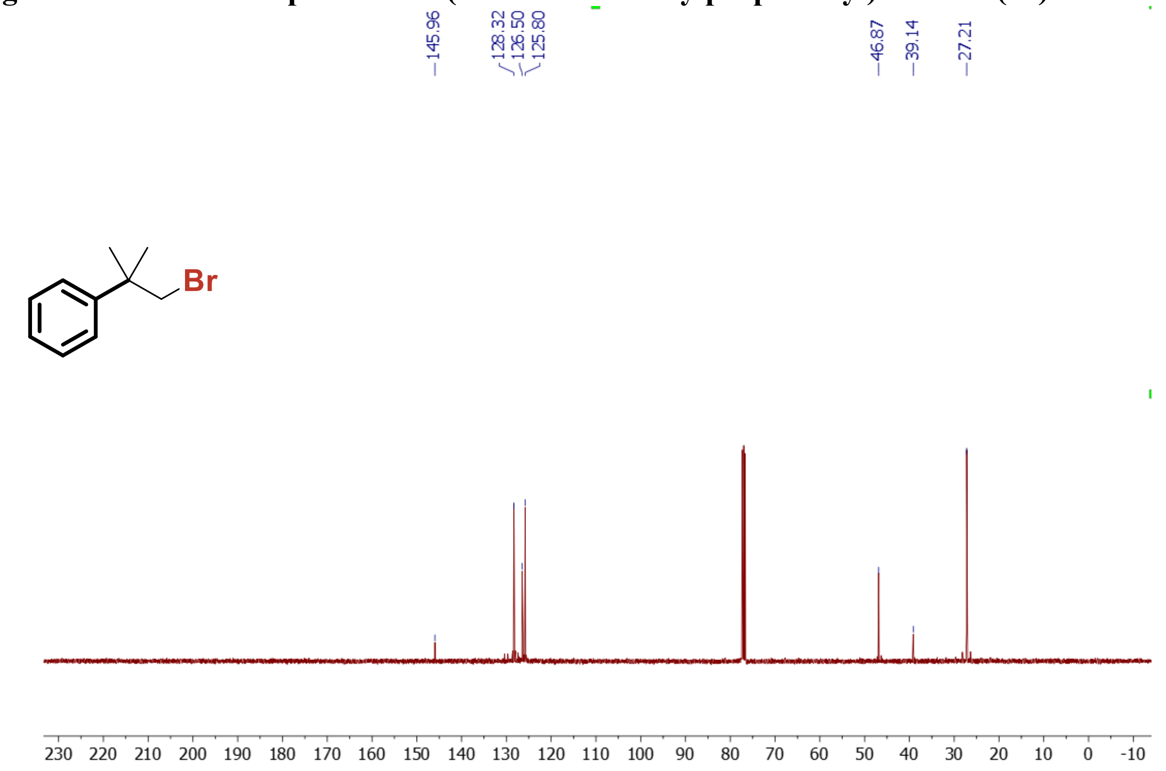

Figure S88. ^{1}**H NMR spectrum of (3-iodopropyl)benzene (50).**

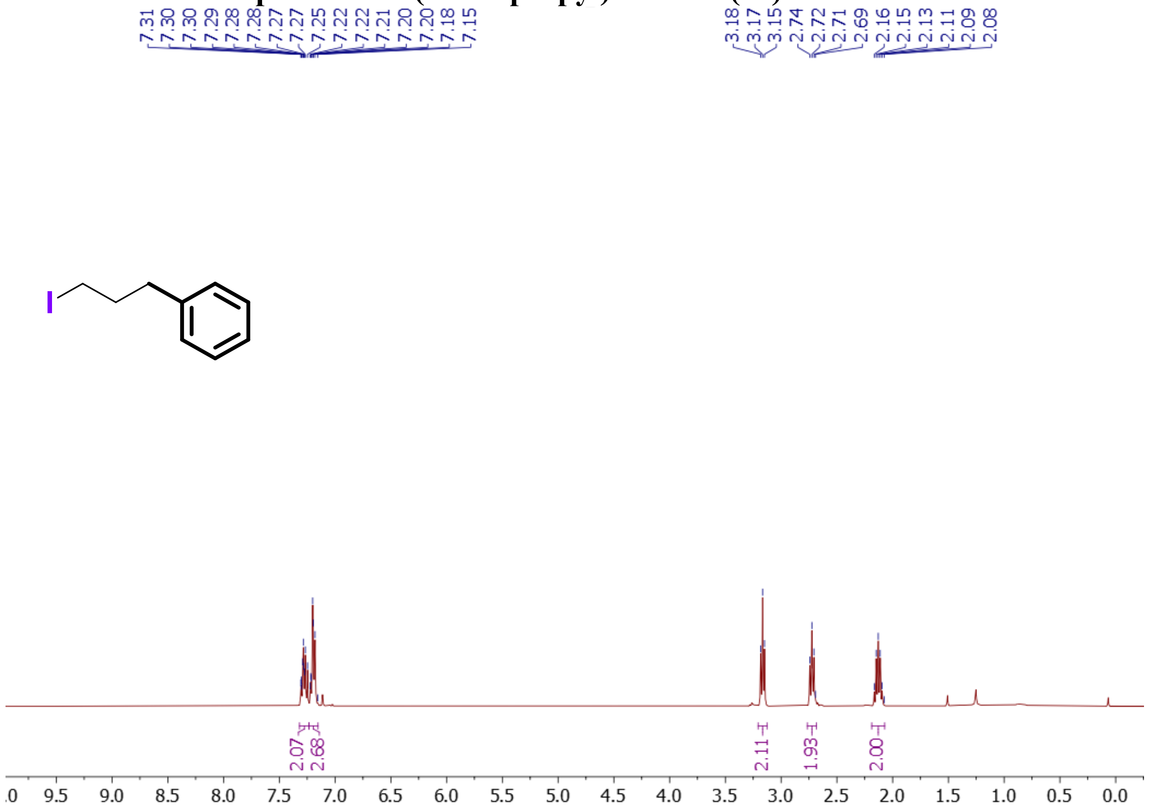

Figure S89. ^{13}**C NMR spectrum of (3-iodopropyl)benzene (50).**

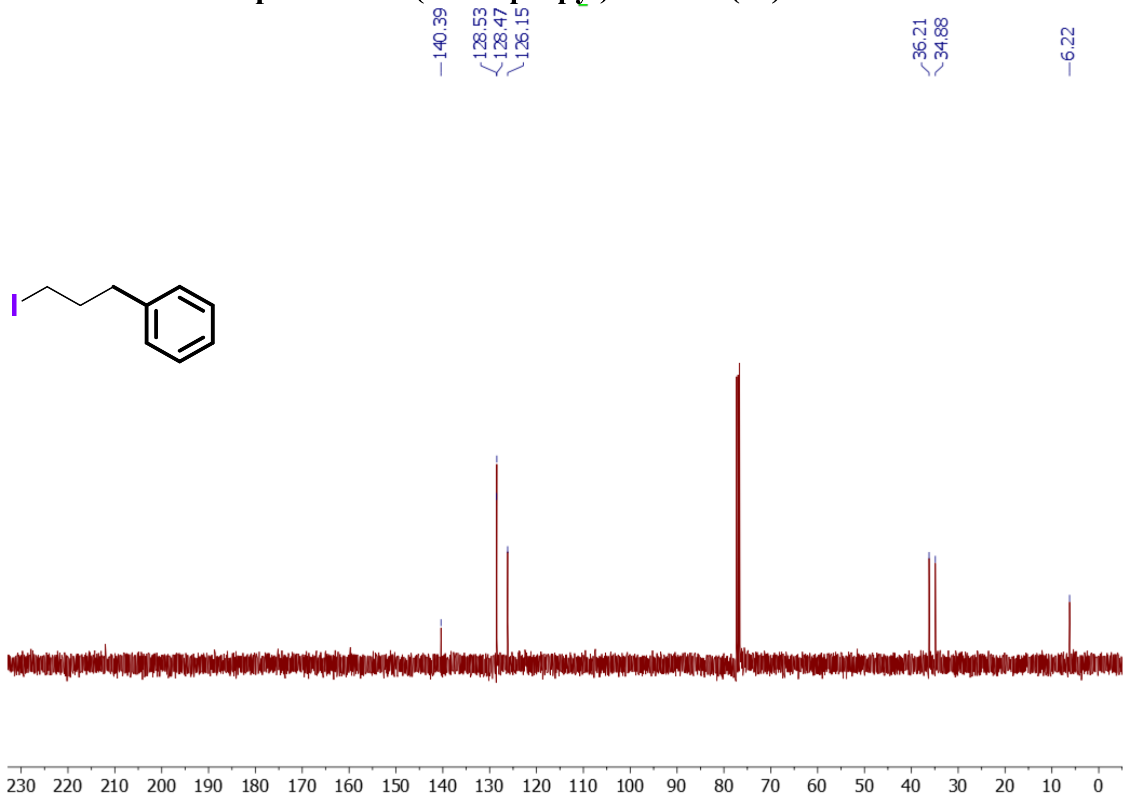

Figure S90. ^{1}H NMR spectrum of 2-(3-iodopropyl)-1,3,5-trimethylbenzene (51).

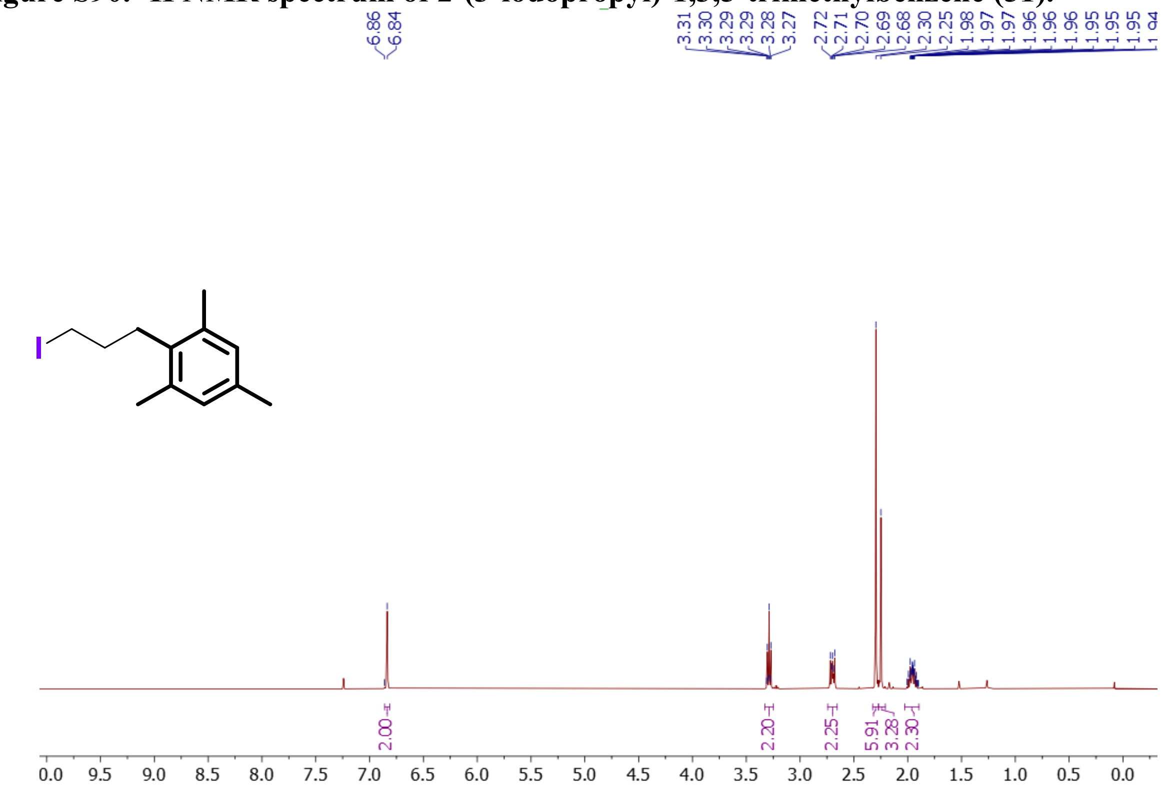

Figure S91. ^{13}C NMR spectrum of 2-(3-iodopropyl)-1,3,5-trimethylbenzene (51).

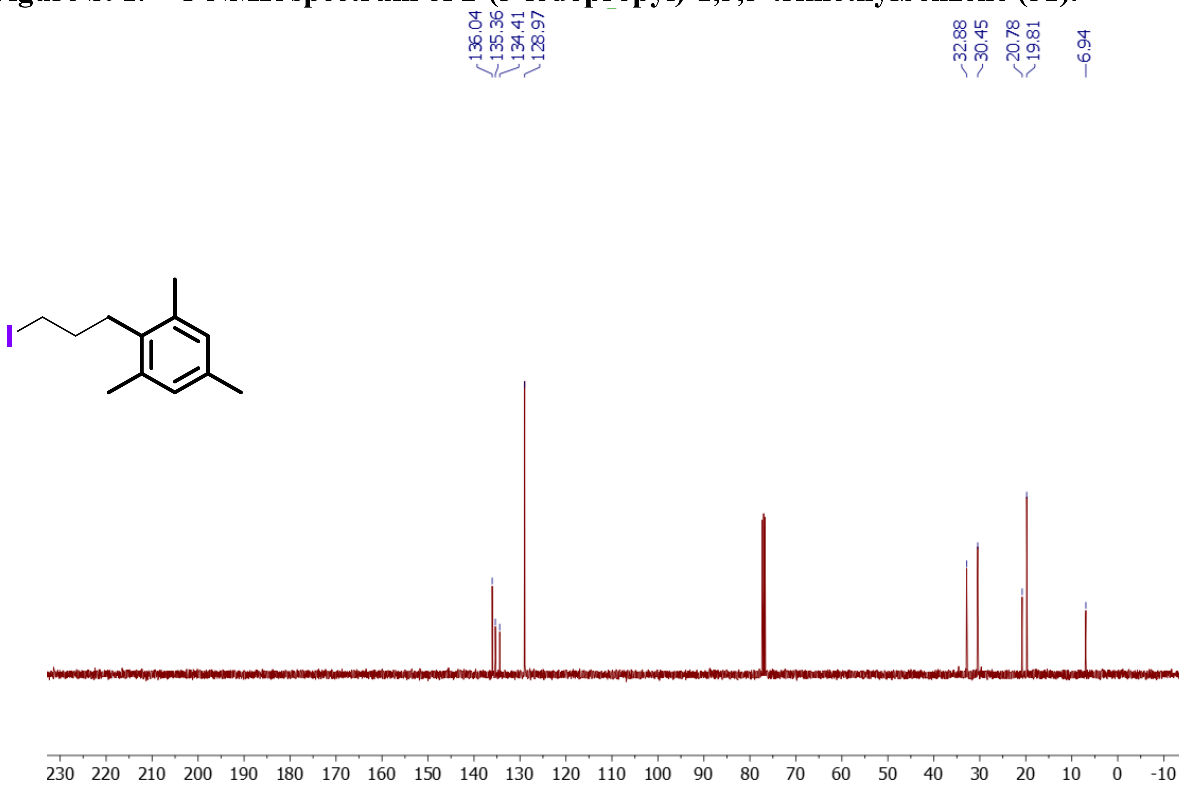

Figure S92. ^{1}H **NMR spectrum of dodecylbenzene (56).**

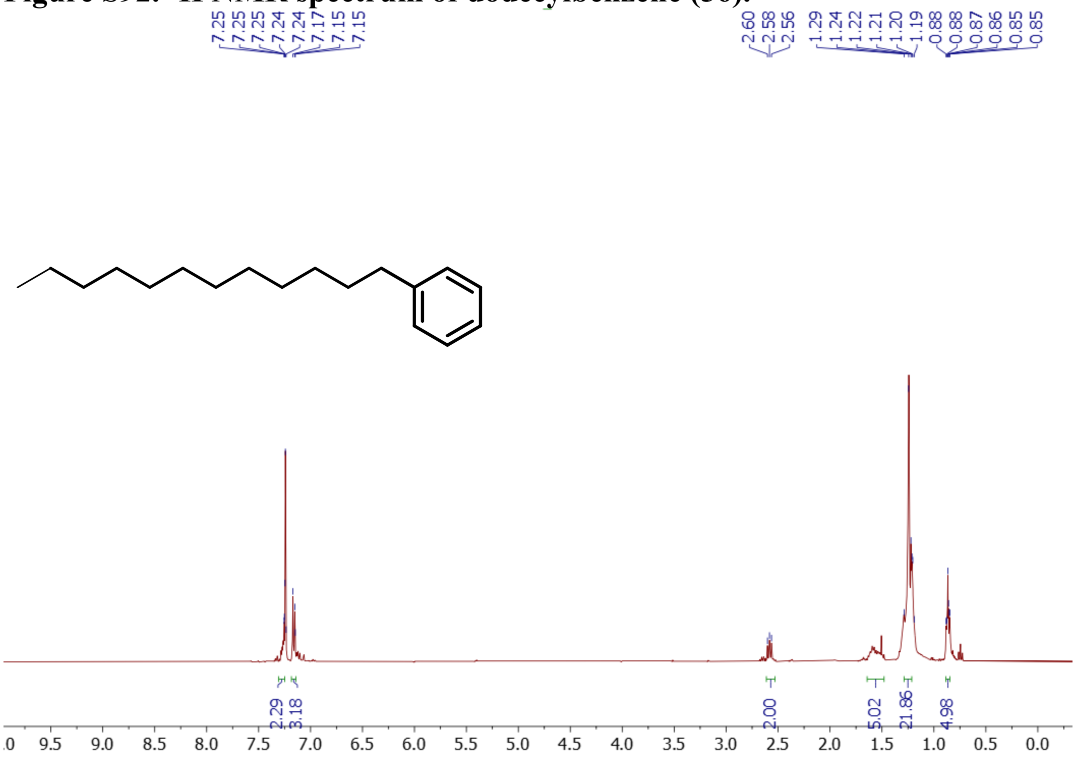

Figure S93. ^{13}C **NMR spectrum of dodecylbenzene (56).**

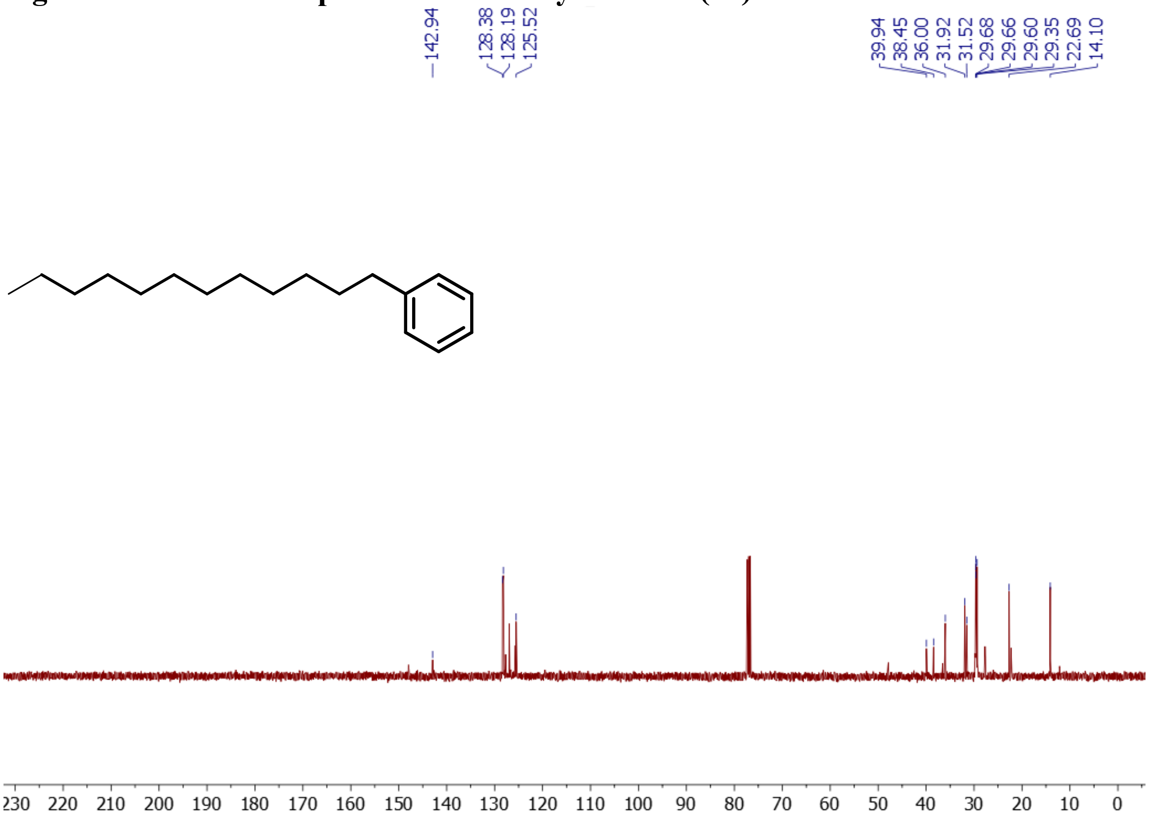

Figure S94. ^{1}H NMR spectrum of 1-chloro-4-(3-phenylpropyl)benzene (57).

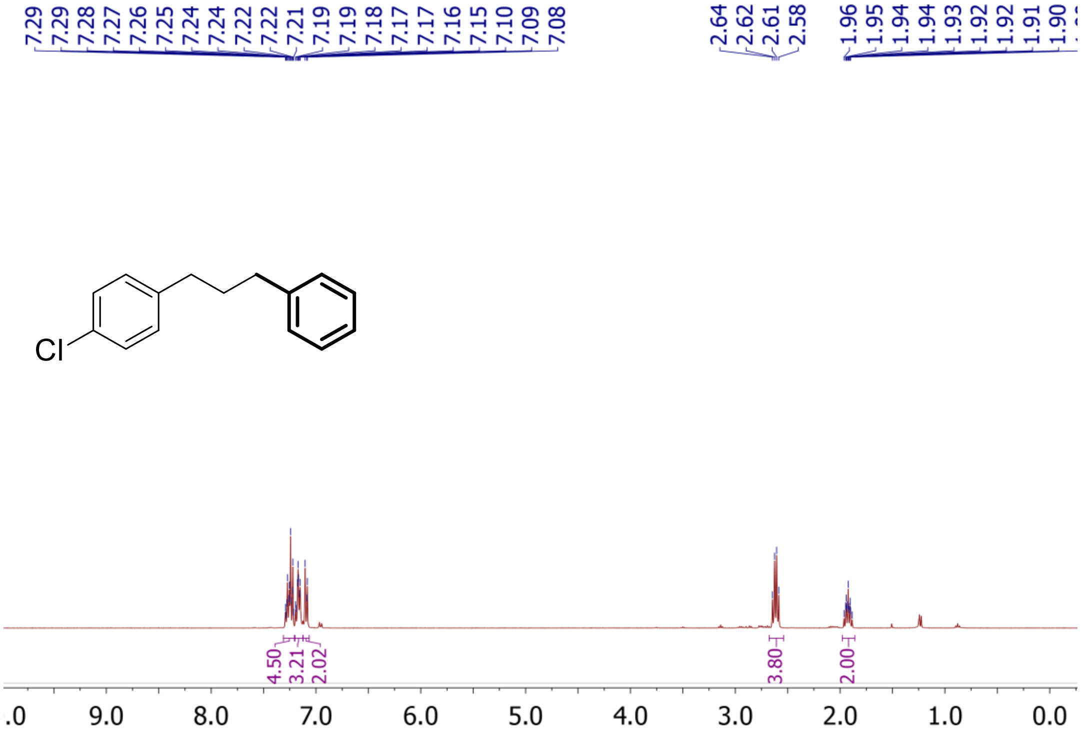

Figure S95. ^{13}C NMR spectrum of 1-chloro-4-(3-phenylpropyl)benzene (57).

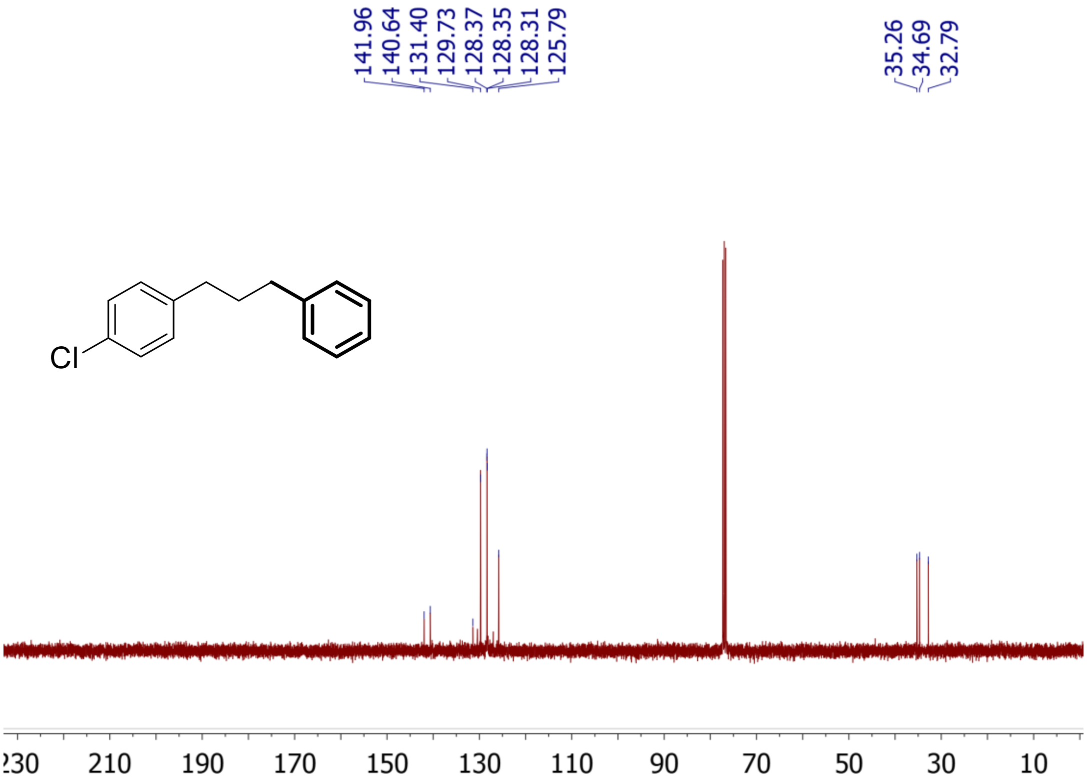

Figure S96. ^{1}H NMR spectrum of 3-phenyl-1-propanol (58).

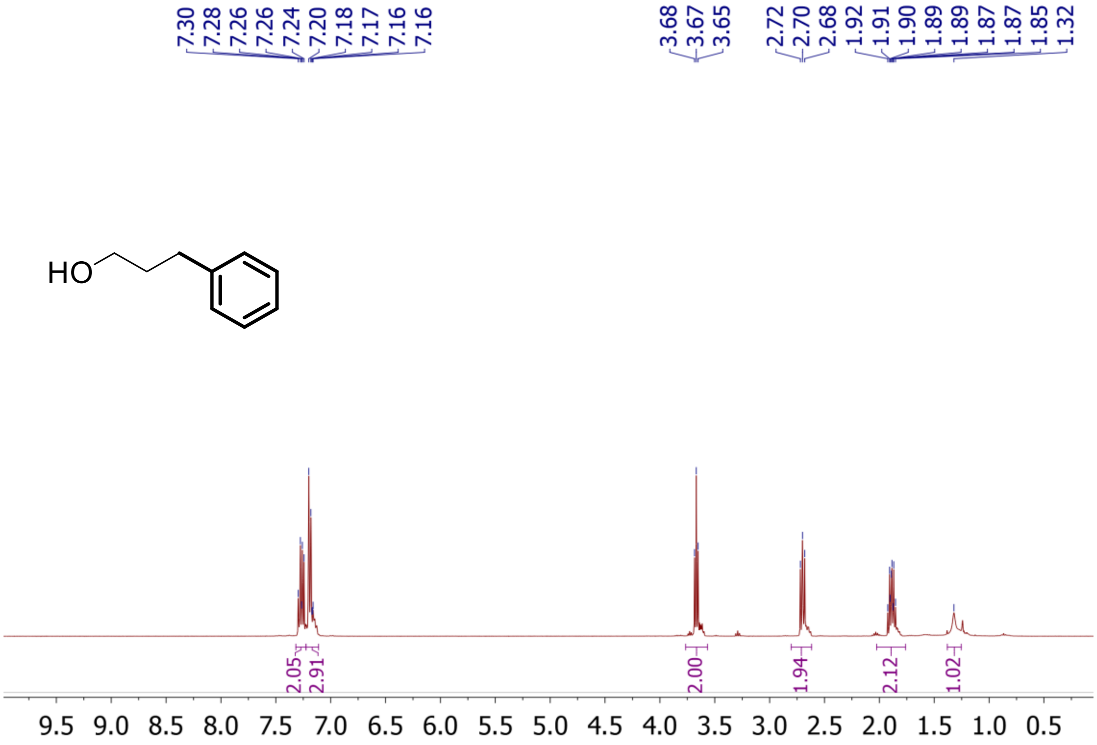

Figure S97. ^{13}C NMR spectrum of 3-phenyl-1-propanol (58).

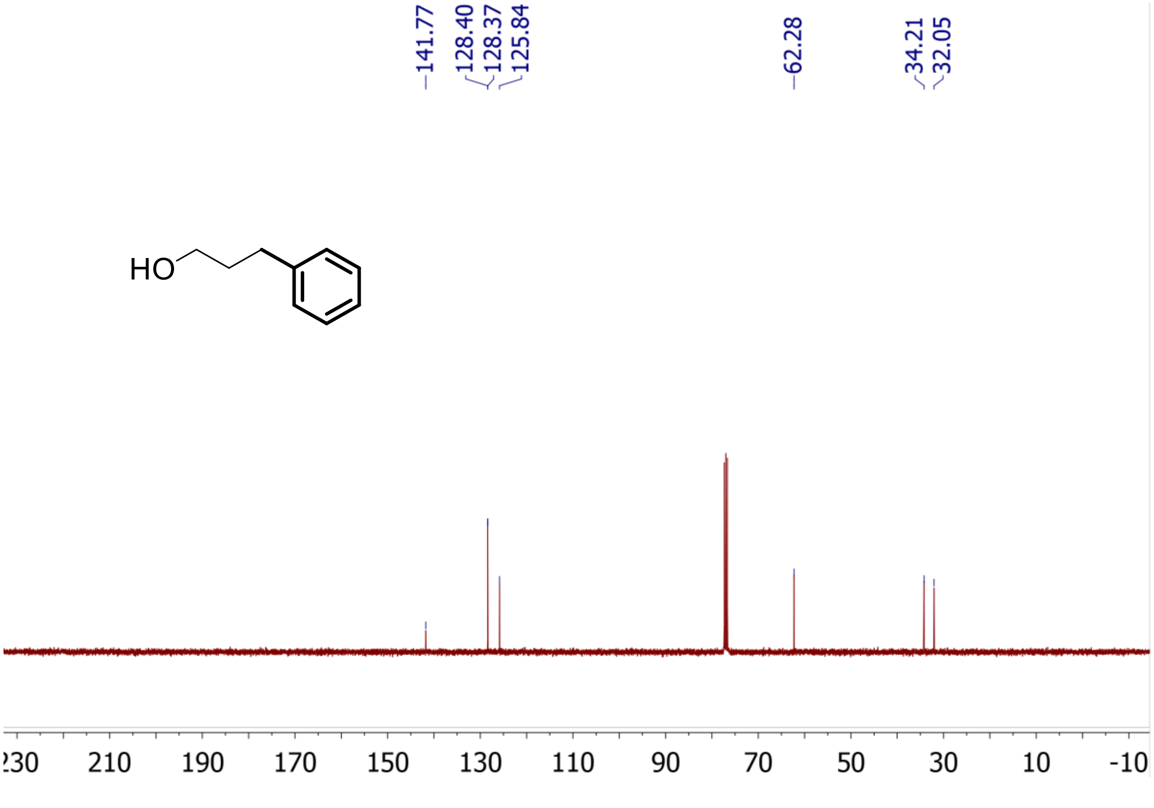

Figure S98. ^{1}H NMR spectrum of methyl 4-(4-phenylbutyl)benzoate (59).

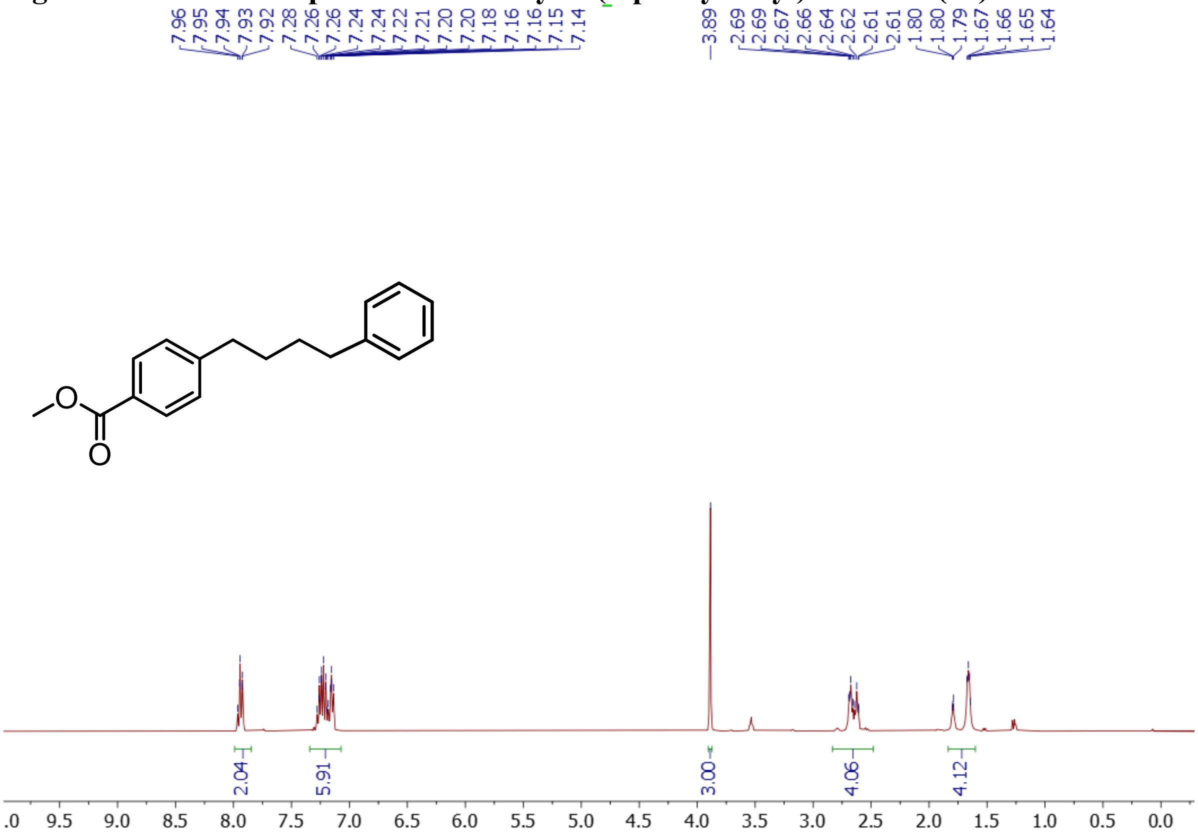

Figure S99. ^{13}C NMR spectrum of methyl 4-(4-phenylbutyl)benzoate (59).

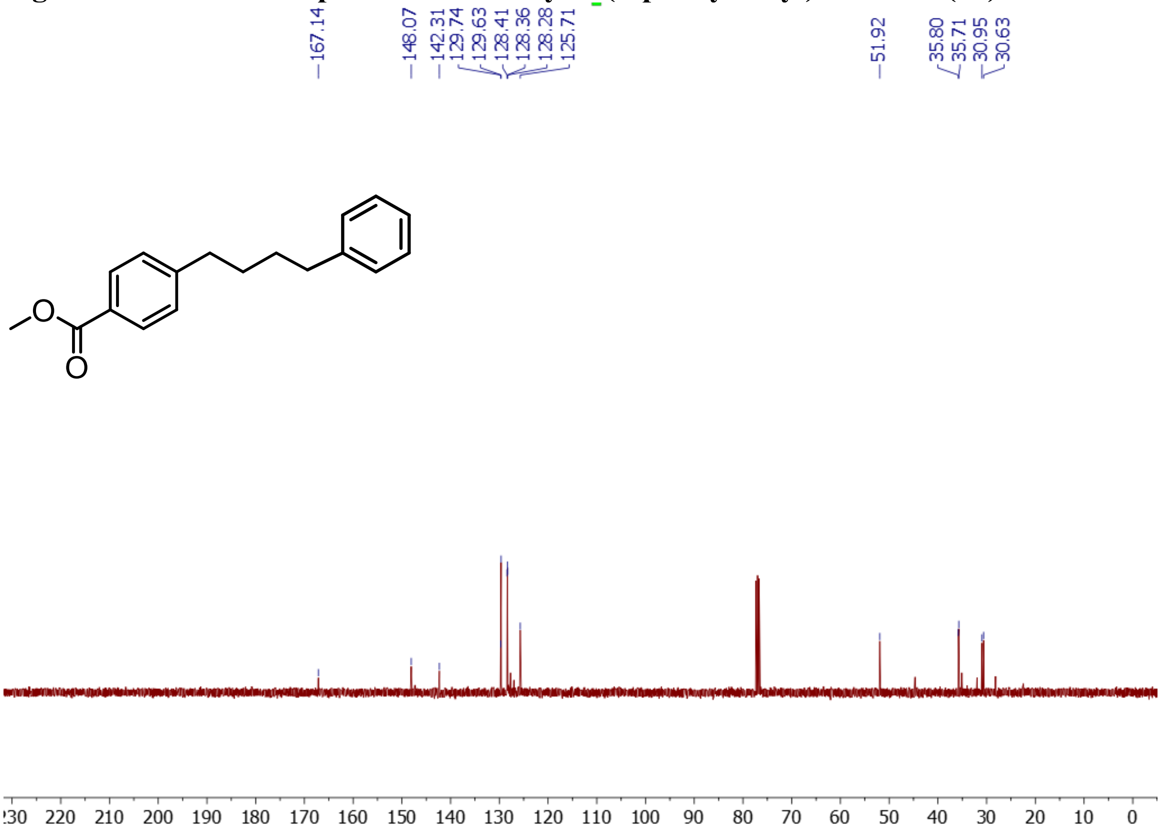

Figure S100. ^{1}H NMR spectrum of 1-chloro-4-(5-phenylpent-4-yn-1-yl)benzene (60).

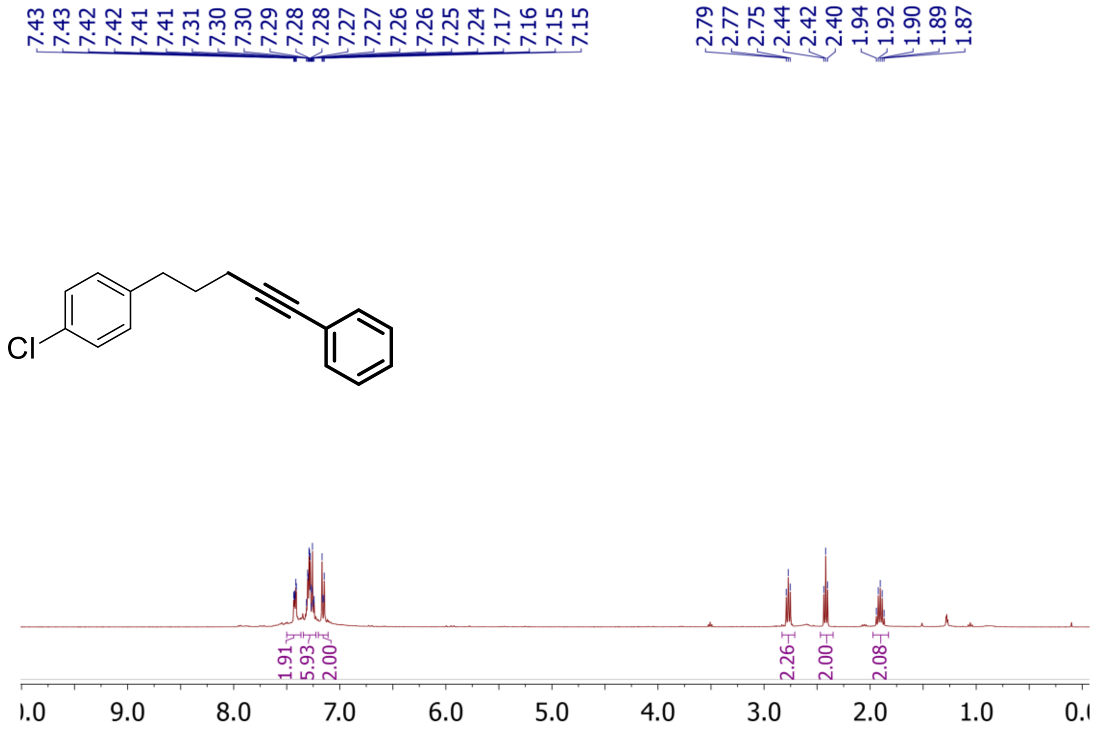

Figure S101. ^{13}C NMR spectrum of 1-chloro-4-(5-phenylpent-4-yn-1-yl)benzene (60).

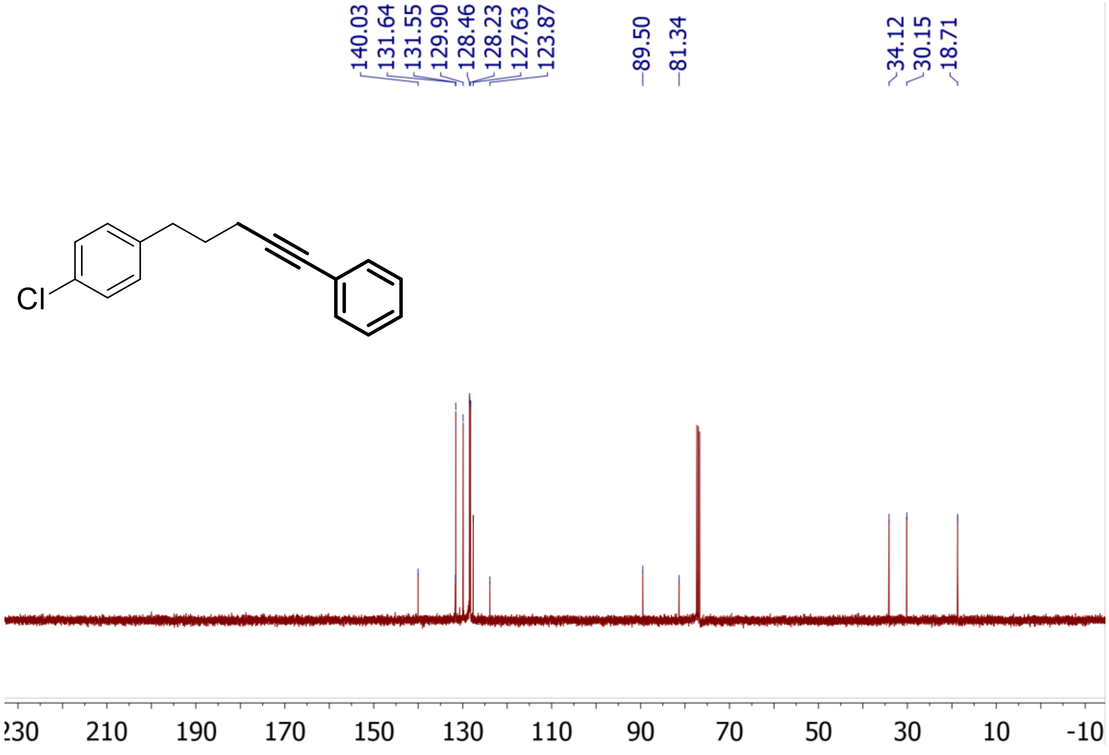

Figure S102. ^{1}**H NMR spectrum of dec-2-yn-1-ylbenzene (61).**

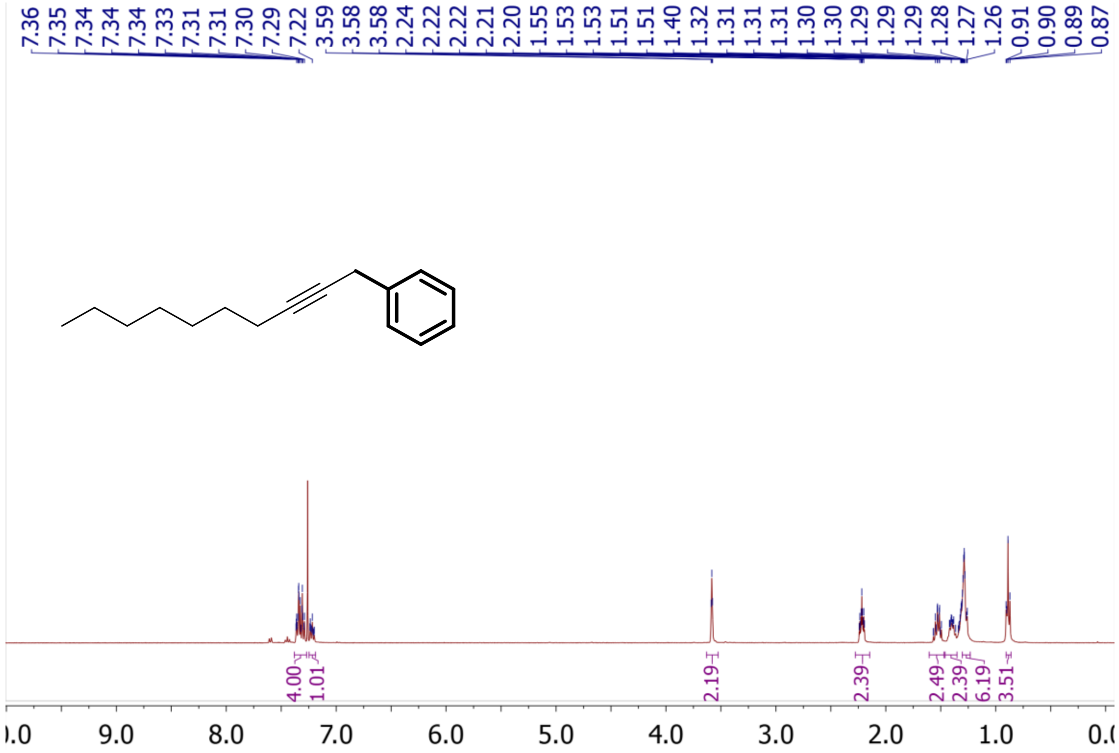

Figure S103. ^{13}**C NMR spectrum of dec-2-yn-1-ylbenzene (61).**

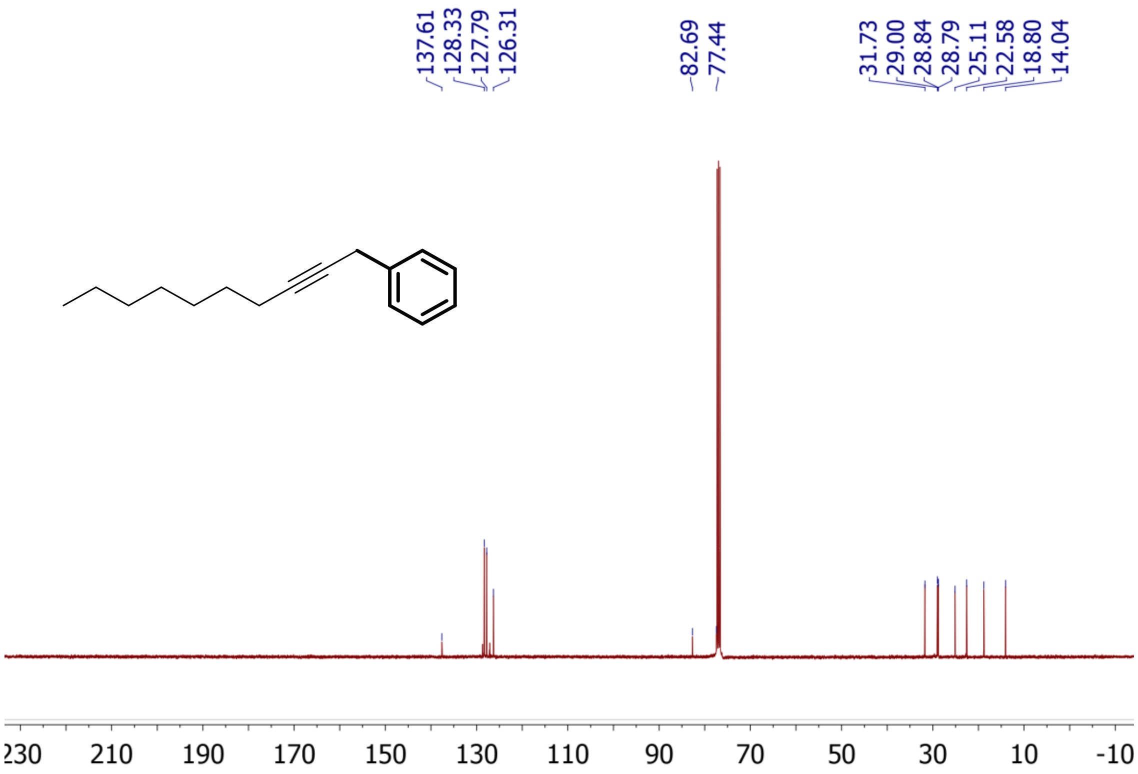

Figure S104. ^{1}H NMR spectrum of 3-benzyl-*N,N*-dimethylaniline (62).

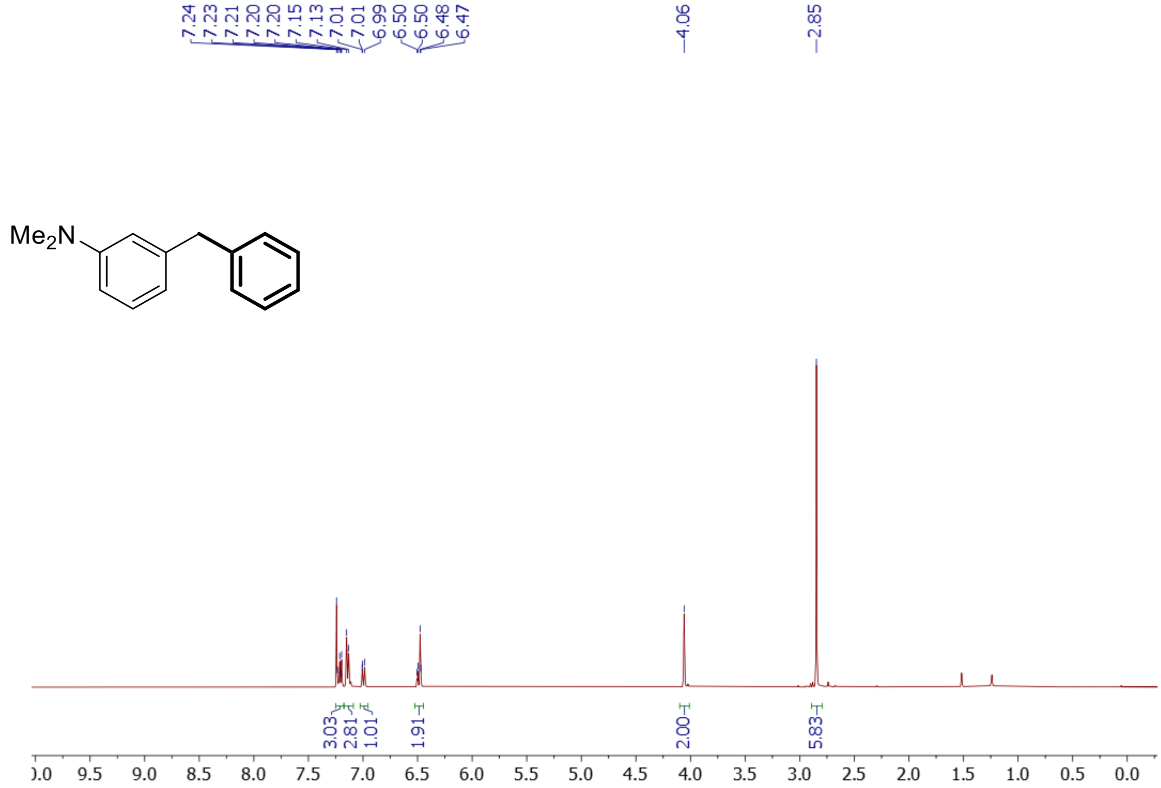

Figure S105. ^{13}C NMR spectrum of 3-benzyl-*N,N*-dimethylaniline (62).

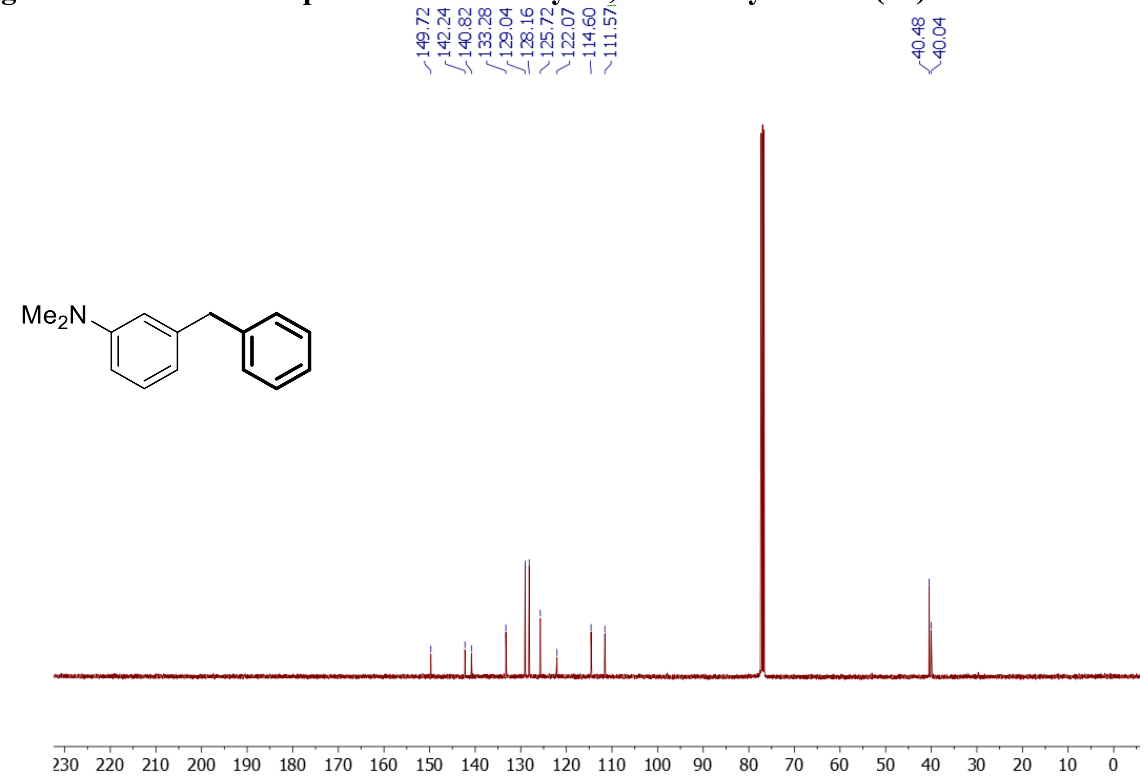

Figure S106. ^{1}H NMR spectrum of (cyclohexylethynyl)benzene (63).

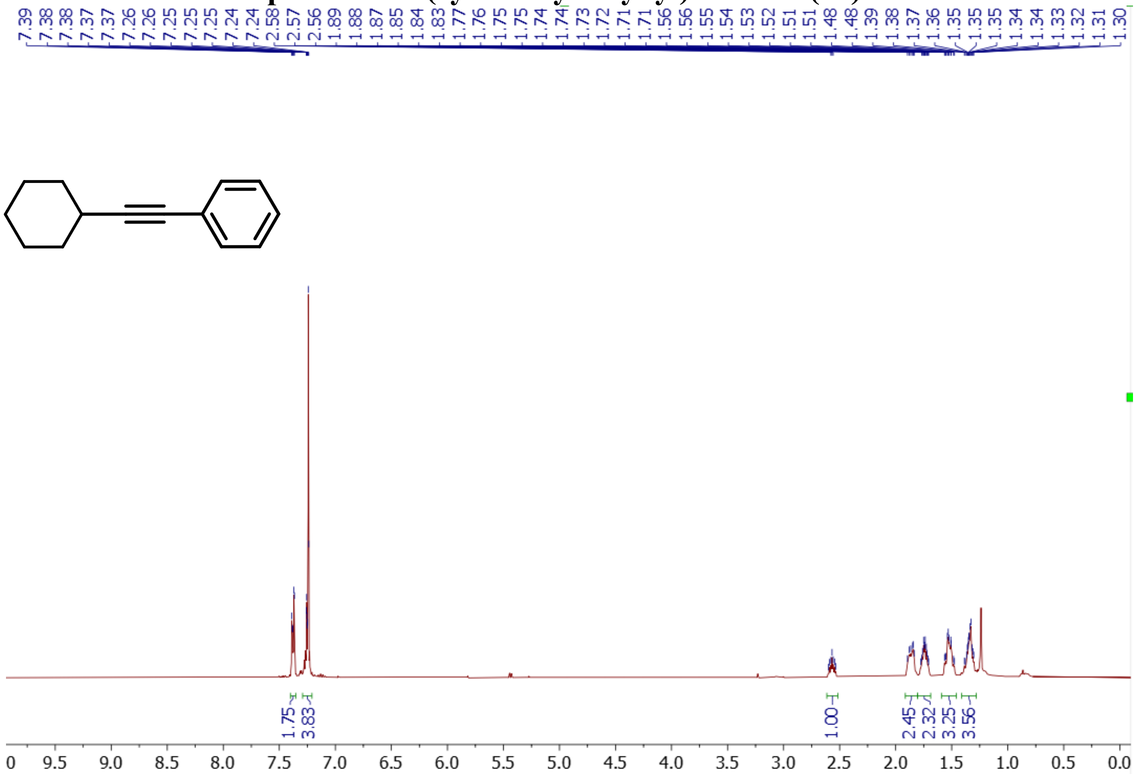

Figure S107. ^{13}C NMR spectrum of (cyclohexylethynyl)benzene (63).

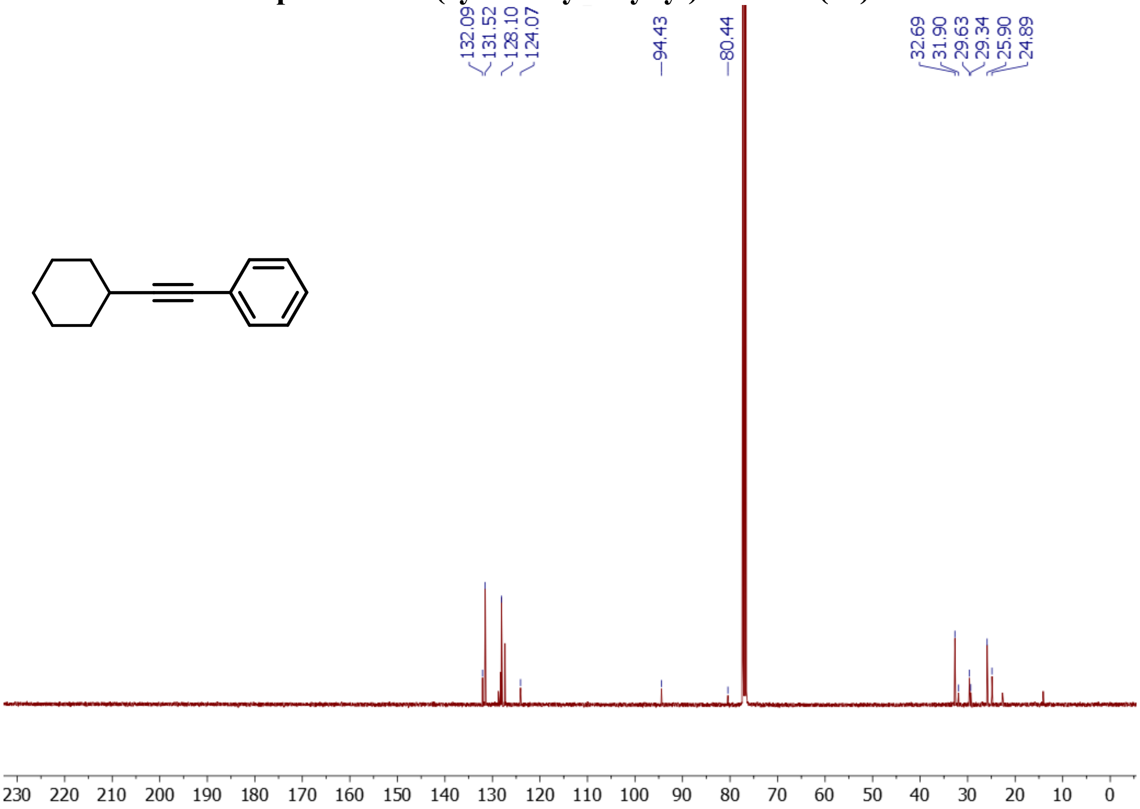

Figure S108. ^{1}H NMR spectrum of 1,3-diphenylbutane (64).

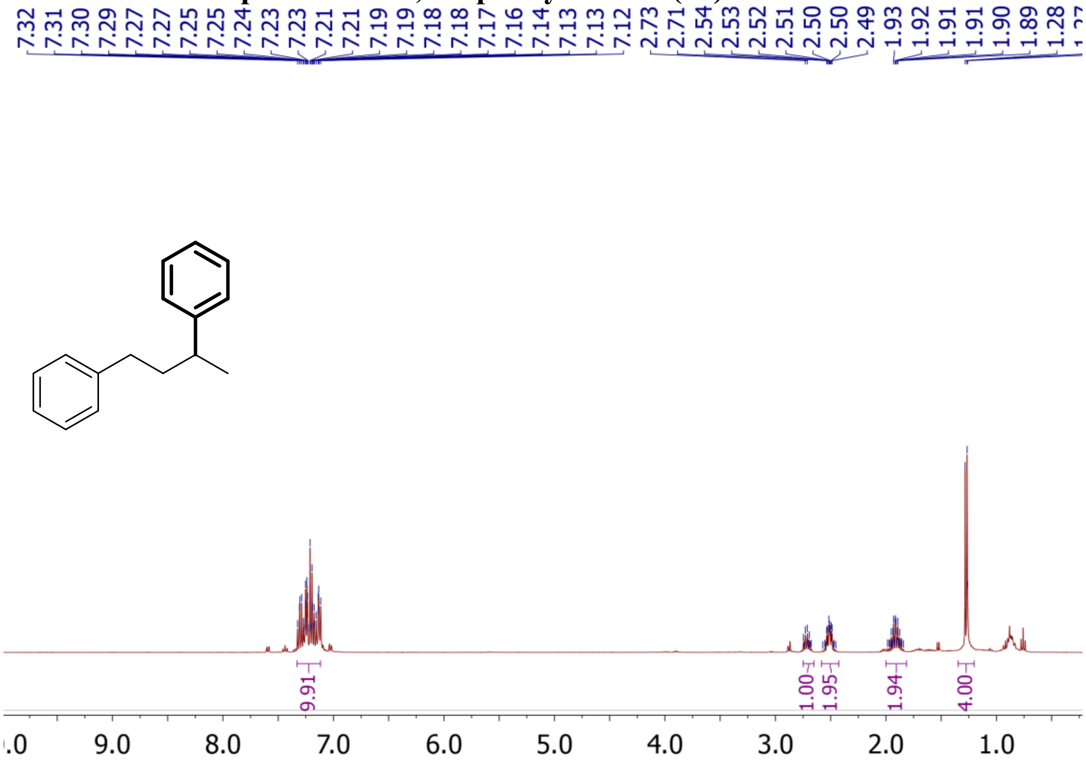

Figure S109. ^{13}C NMR spectrum of 1,3-diphenylbutane (64).

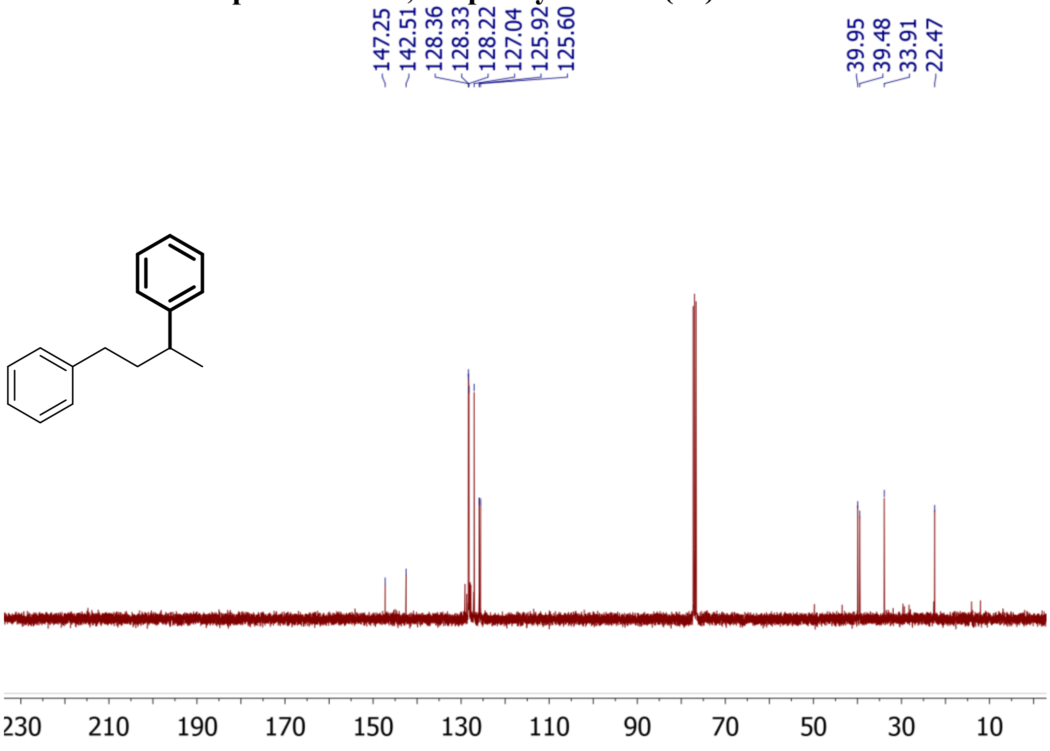

Figure S110. ^{1}H NMR spectrum of 5-(3-phenylbutyl)benzo[d][1,3]dioxole (65).

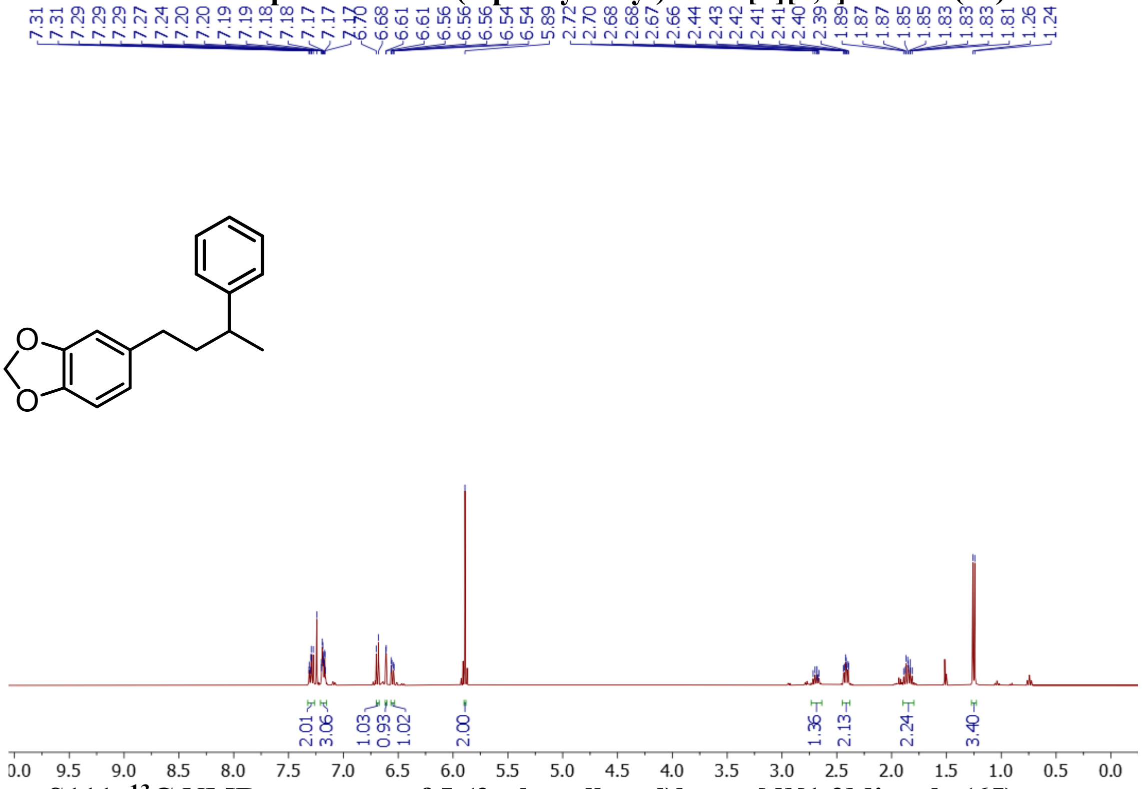

Figure S111. ^{13}C NMR spectrum of 5-(3-phenylbutyl)benzo[d][1,3]dioxole (65).

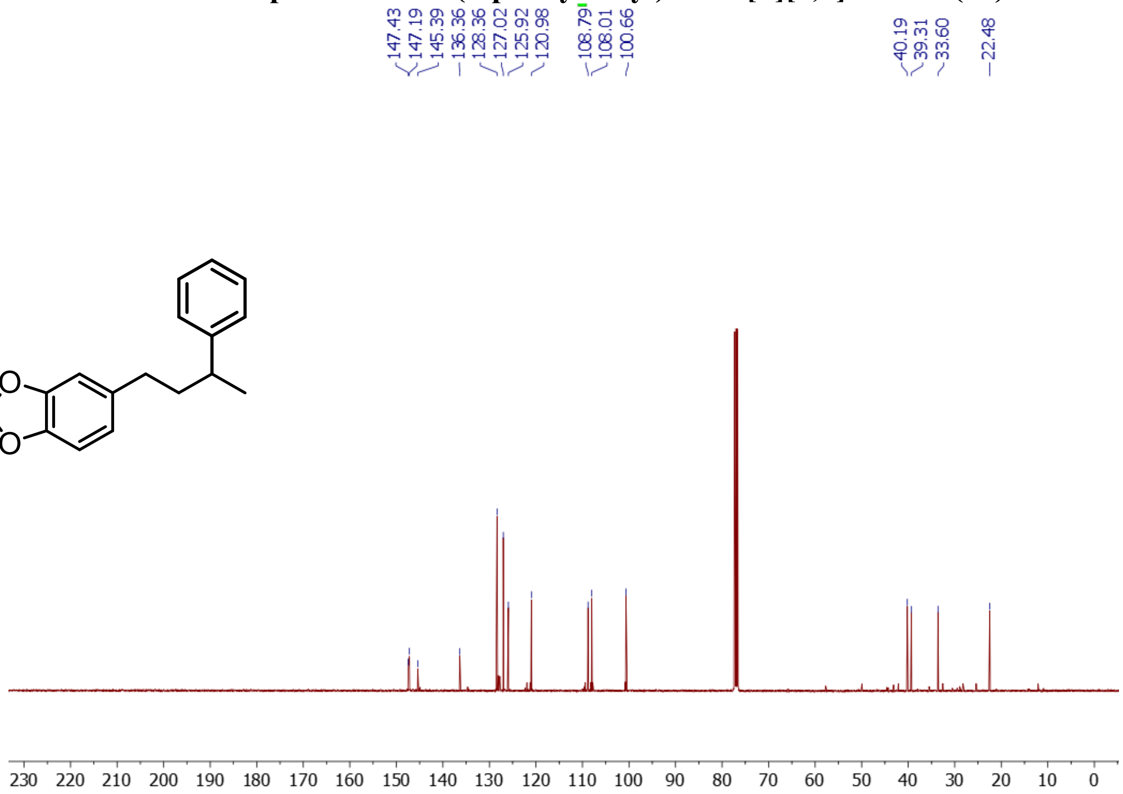

Figure S112. ^{1}H NMR spectrum of ((4-bromophenyl)methylene)dibenzene (66).

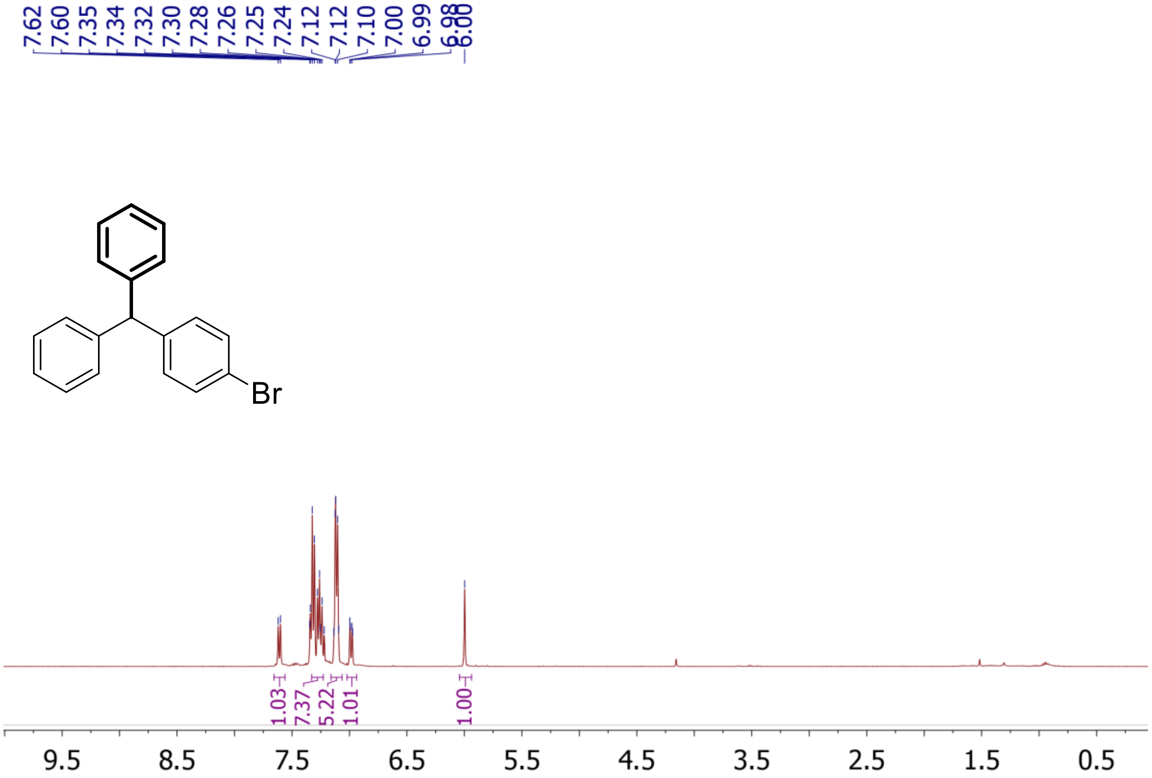

Figure S113. ^{13}C NMR spectrum of ((4-bromophenyl)methylene)dibenzene (66).

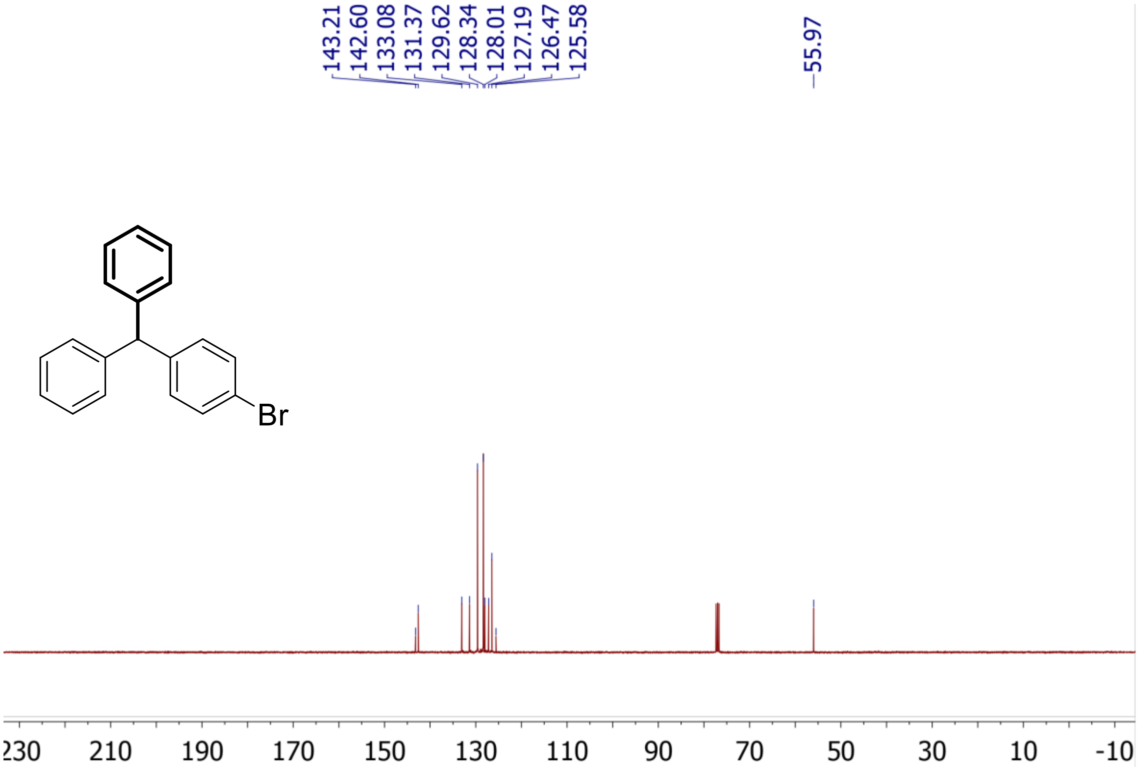

Figure S114. **^{1}H NMR spectrum of 2-((4-chlorophenyl)(phenyl)methyl)pyridine (67).**

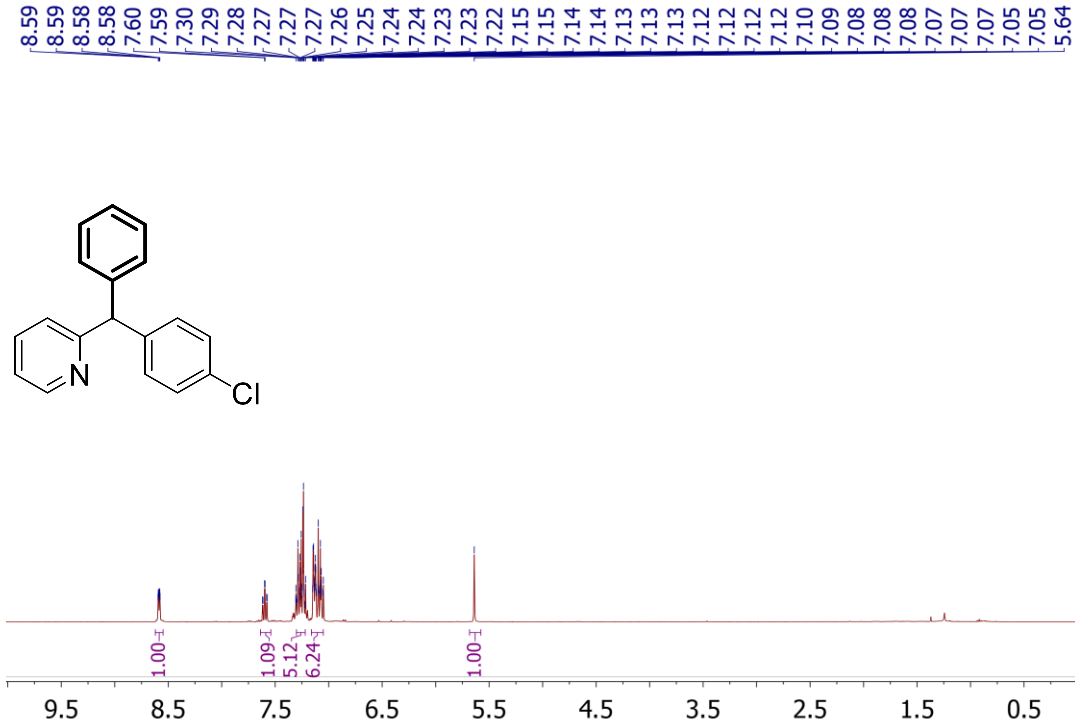

Figure S115. **^{13}C NMR spectrum of 2-((4-chlorophenyl)(phenyl)methyl)pyridine (67).**

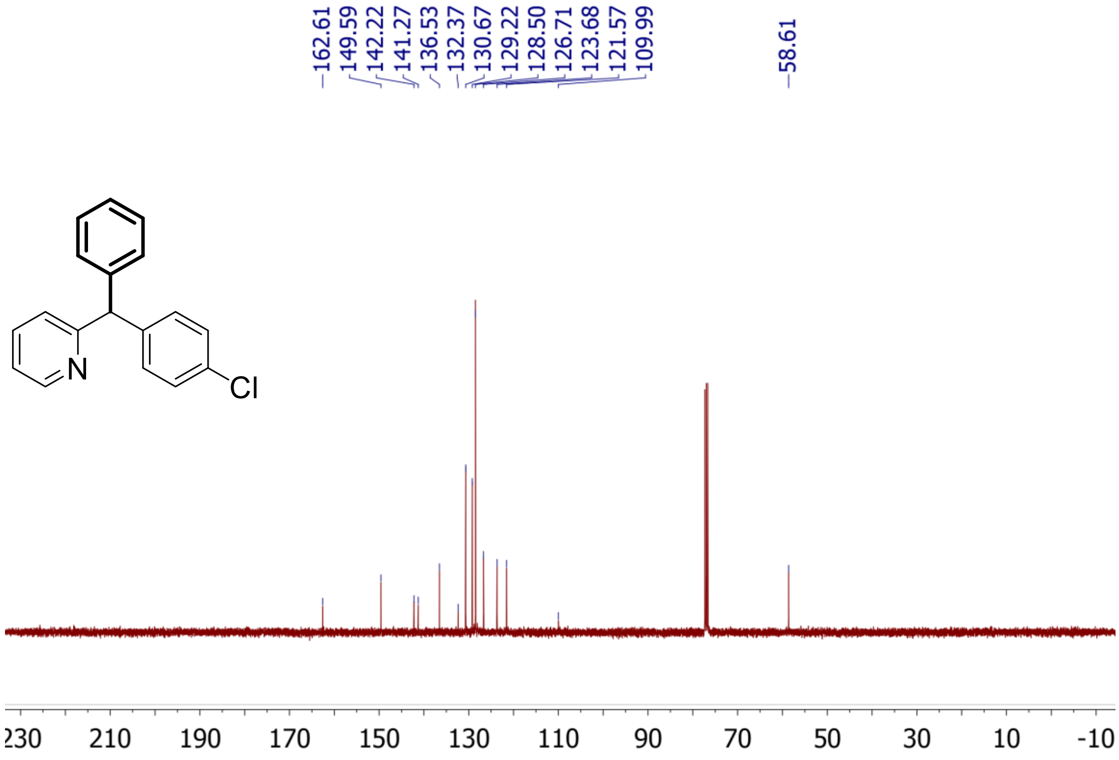

Figure S116. ^{1}H NMR spectrum of (cyclohexylmethylene)dibenzene (68).

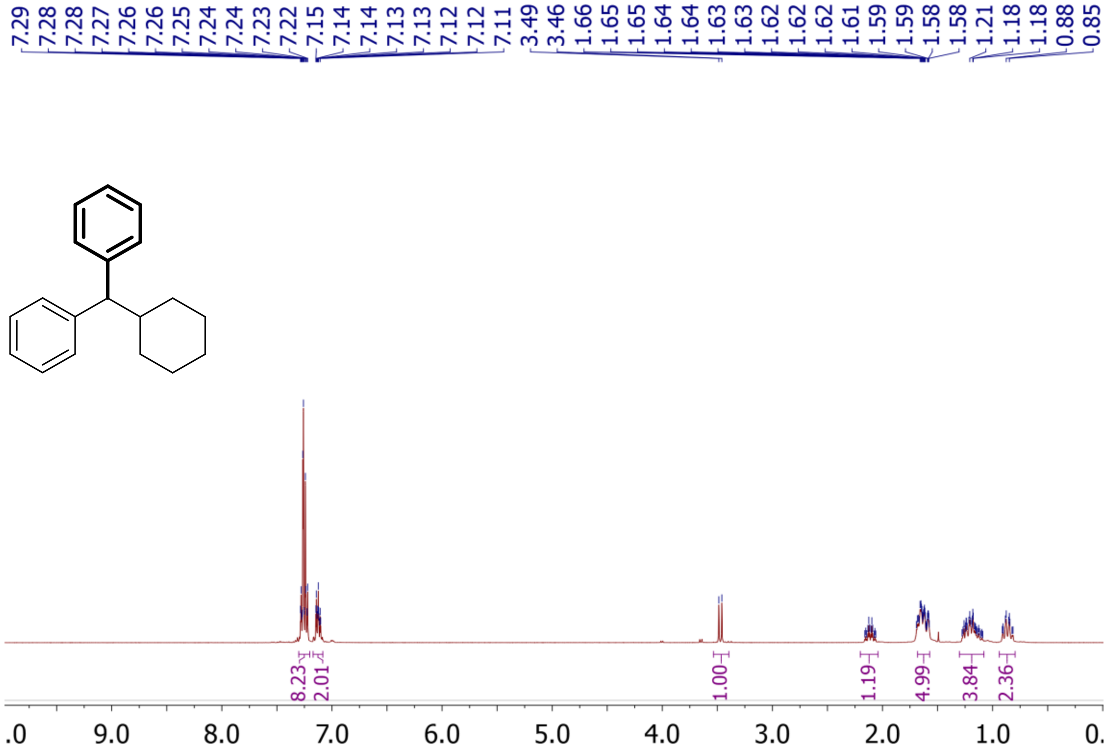

Figure S117. ^{13}C NMR spectrum of (cyclohexylmethylene)dibenzene (68).

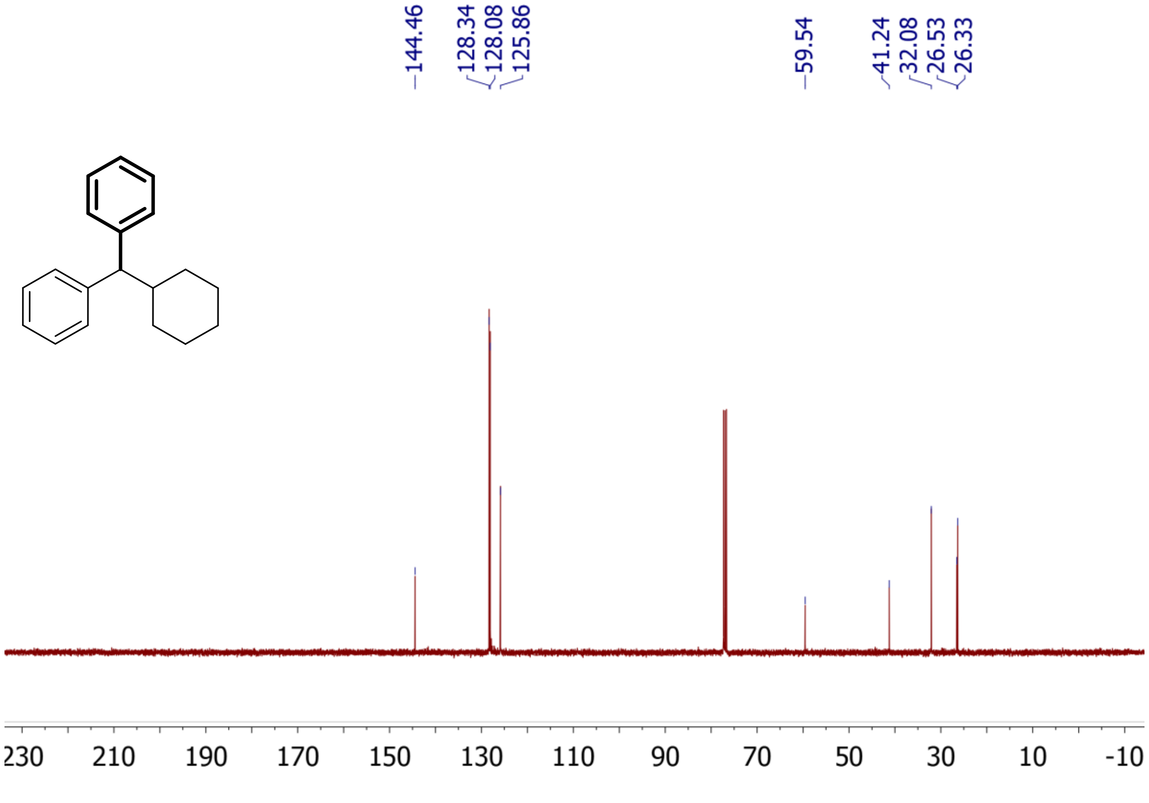

Figure S118. ^{1}**H NMR spectrum of 1-nitro-3-(1-phenylethyl)benzene (69).**

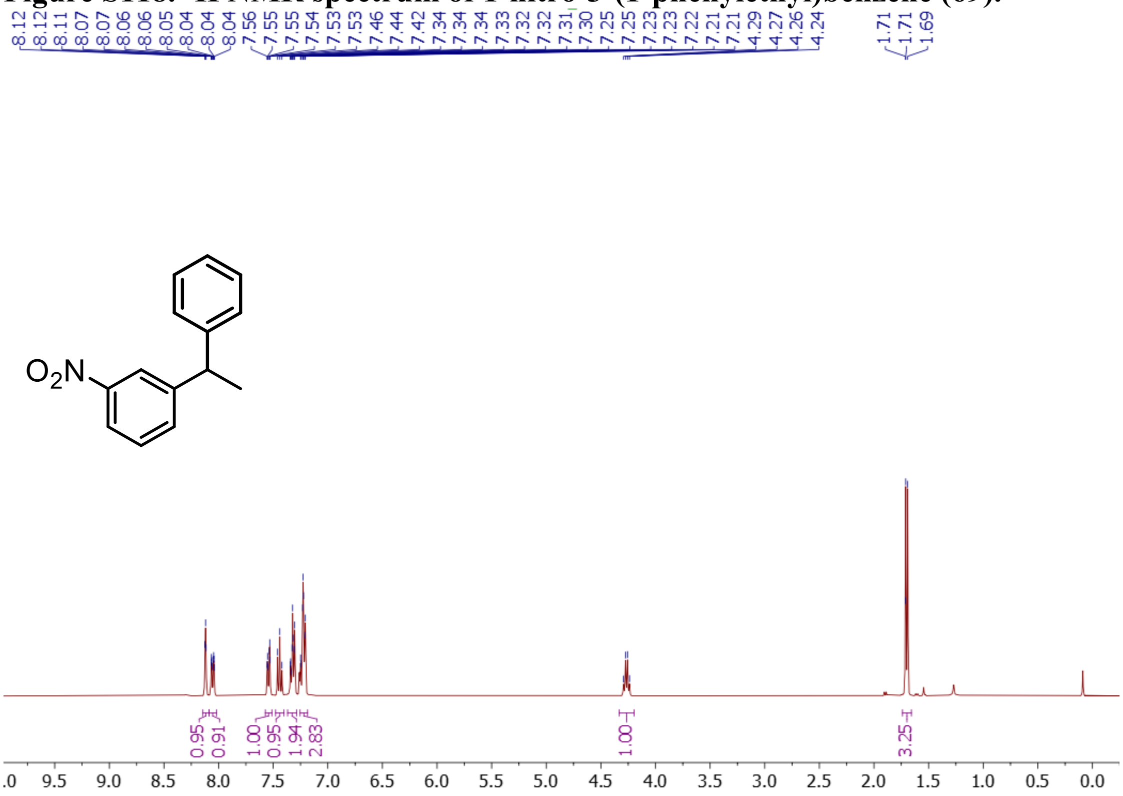

Figure S119. ^{13}**C NMR spectrum of 1-nitro-3-(1-phenylethyl)benzene (69).**

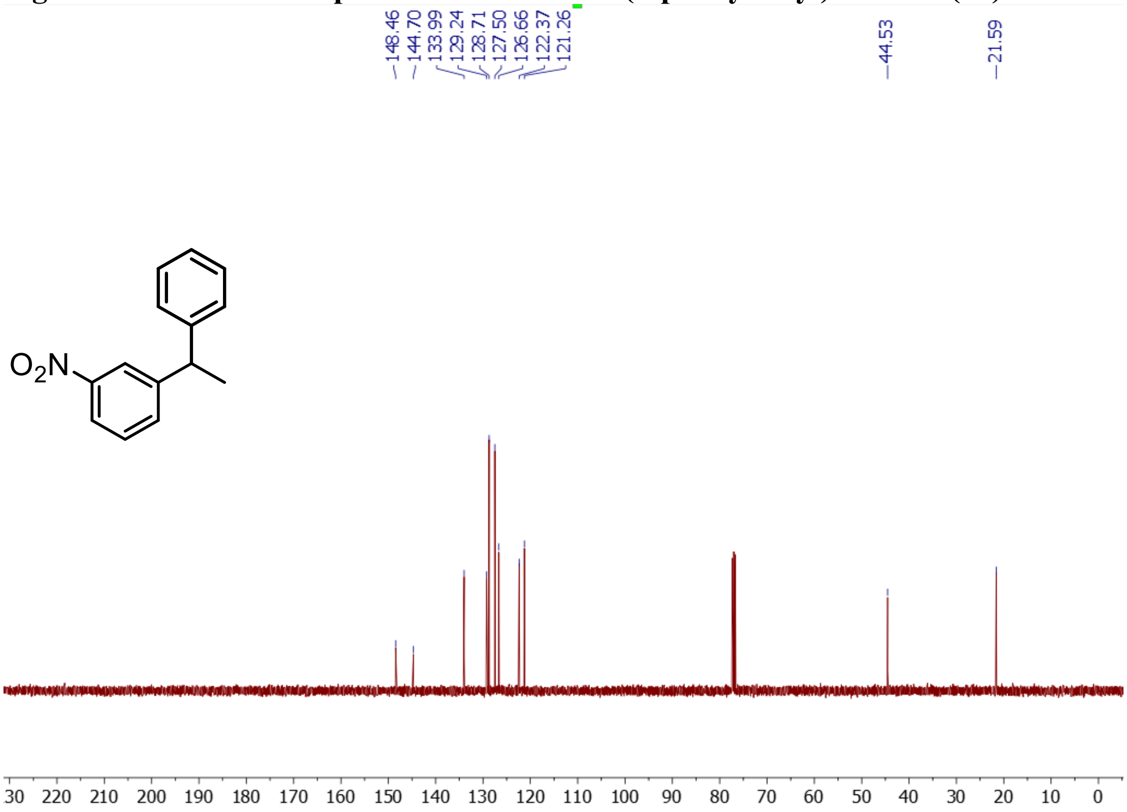

Figure S120. ^{1}H NMR spectrum of but-1-yne-1,3-diyldibenzene (70).

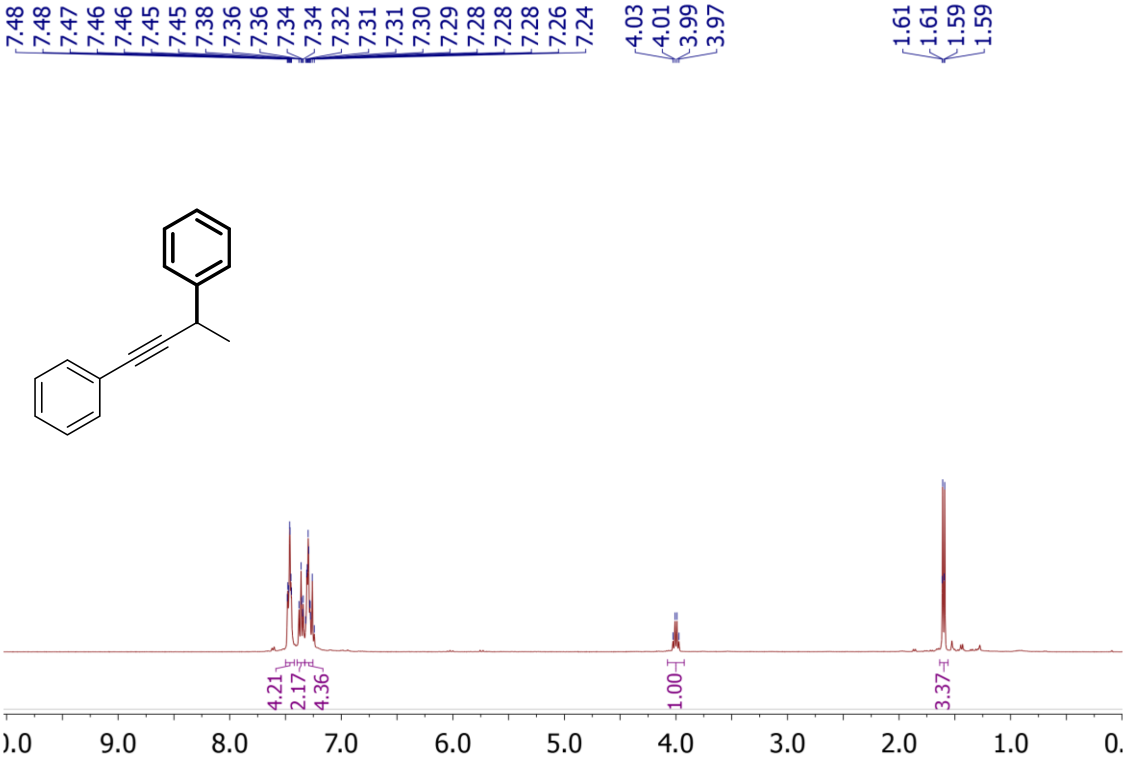

Figure S121. ^{13}C NMR spectrum of but-1-yne-1,3-diyldibenzene (70).

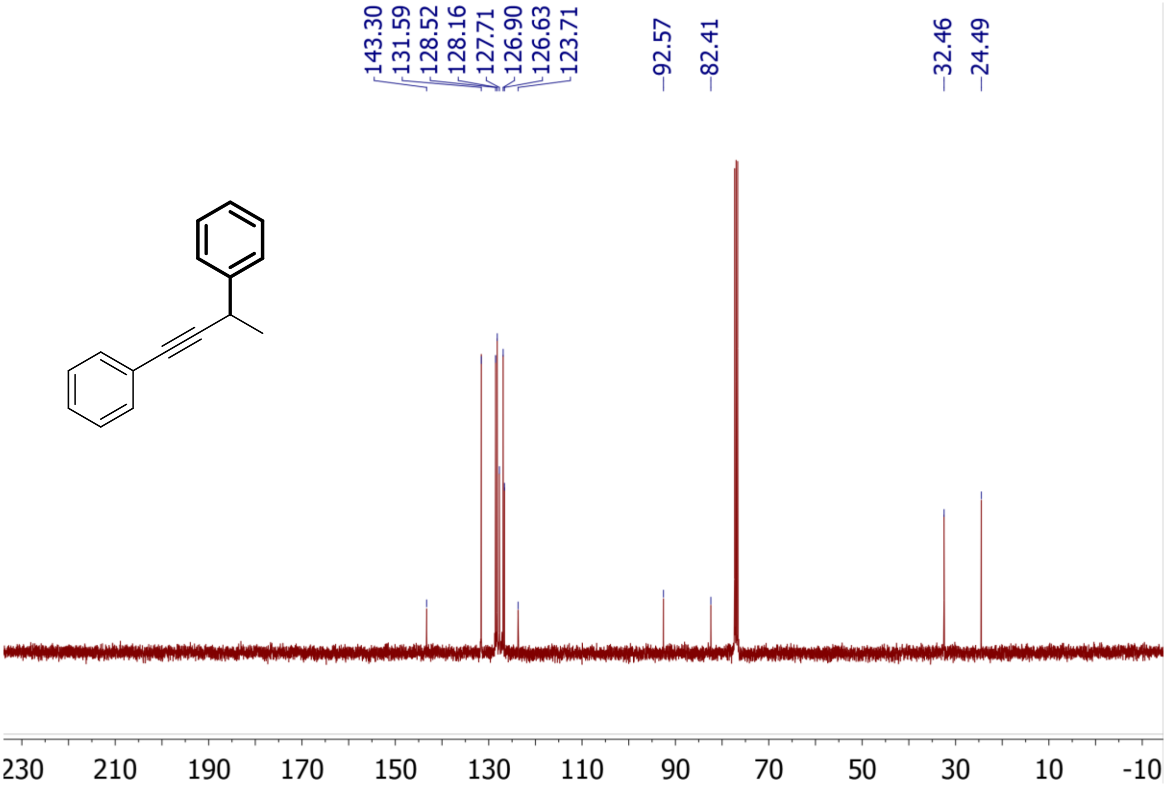

Figure S122. ^{1}H NMR spectrum of (3-methylpenta-1,4-diyne-1,5-diyl)dibenzene (71).

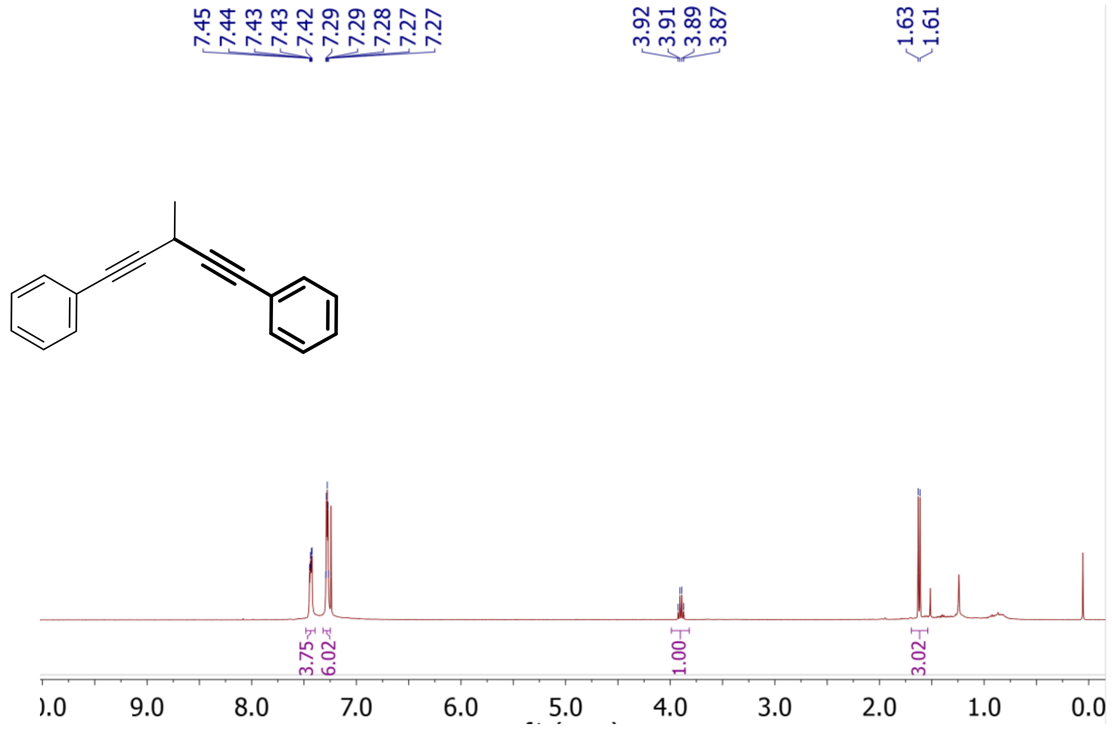

Figure S123. ^{13}C NMR spectrum of (3-methylpenta-1,4-diyne-1,5-diyl)dibenzene (71).

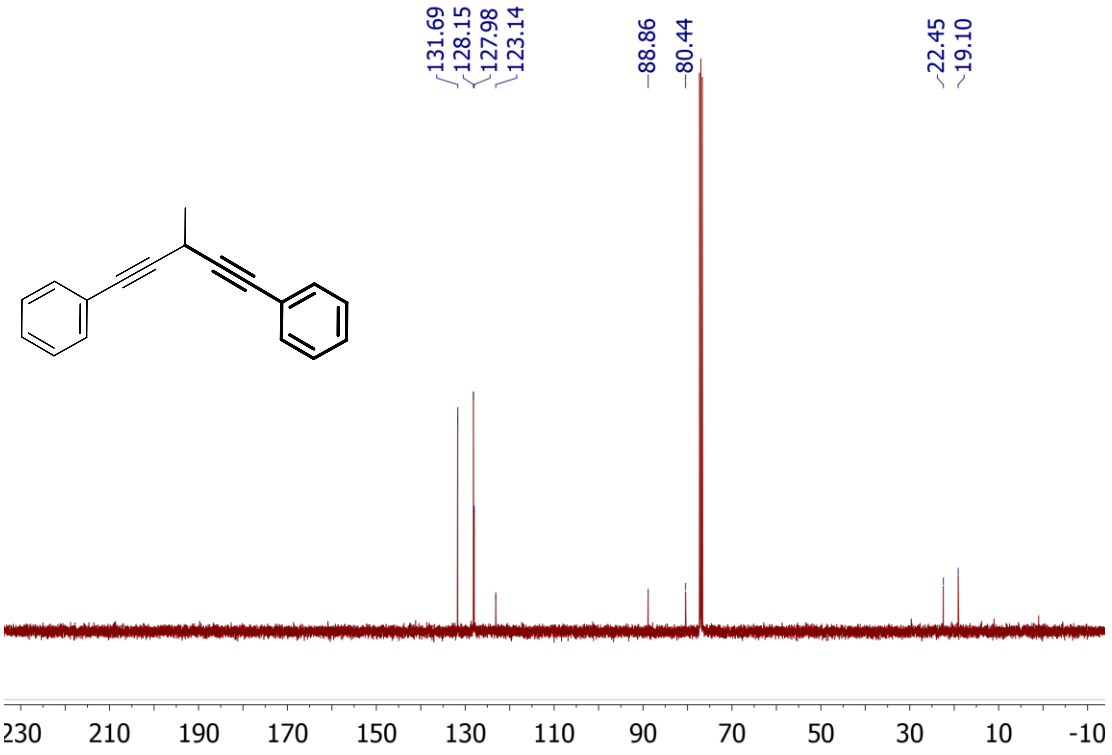

Figure S124. ^{1}H NMR spectrum of (1-(4-bromophenyl)pent-2-yne-1,5-diyl)dibenzene (72).

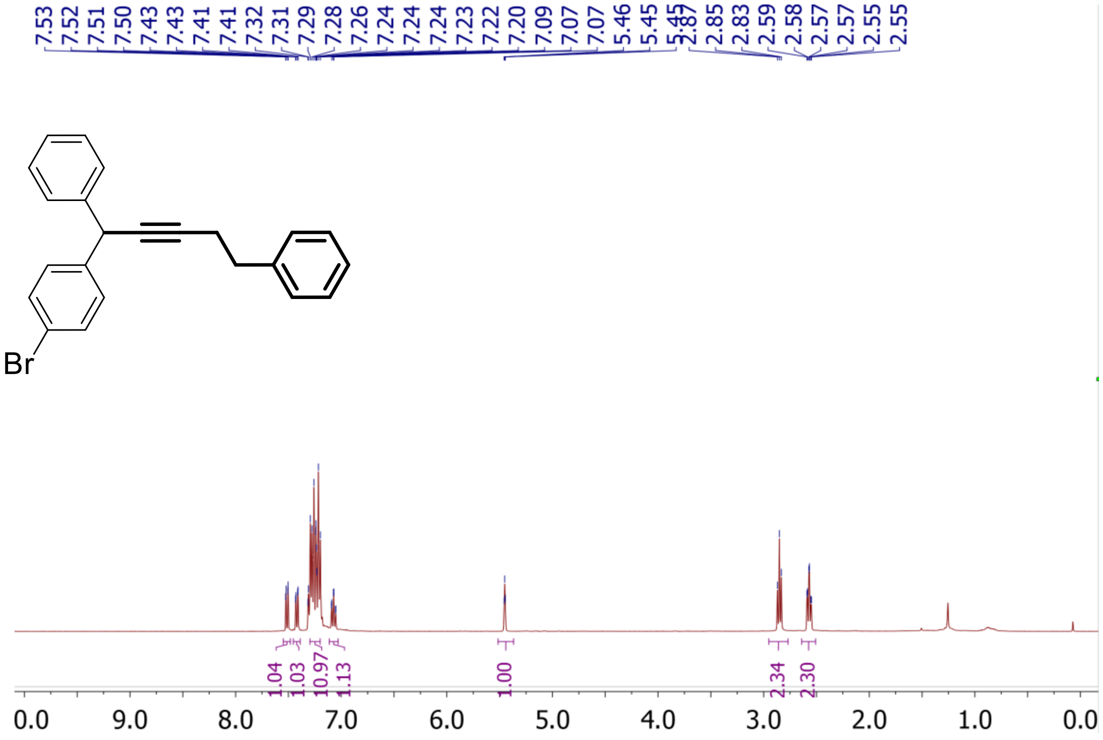

Figure S125. ^{13}C NMR spectrum of (1-(4-bromophenyl)pent-2-yne-1,5-diyl)dibenzene (72).

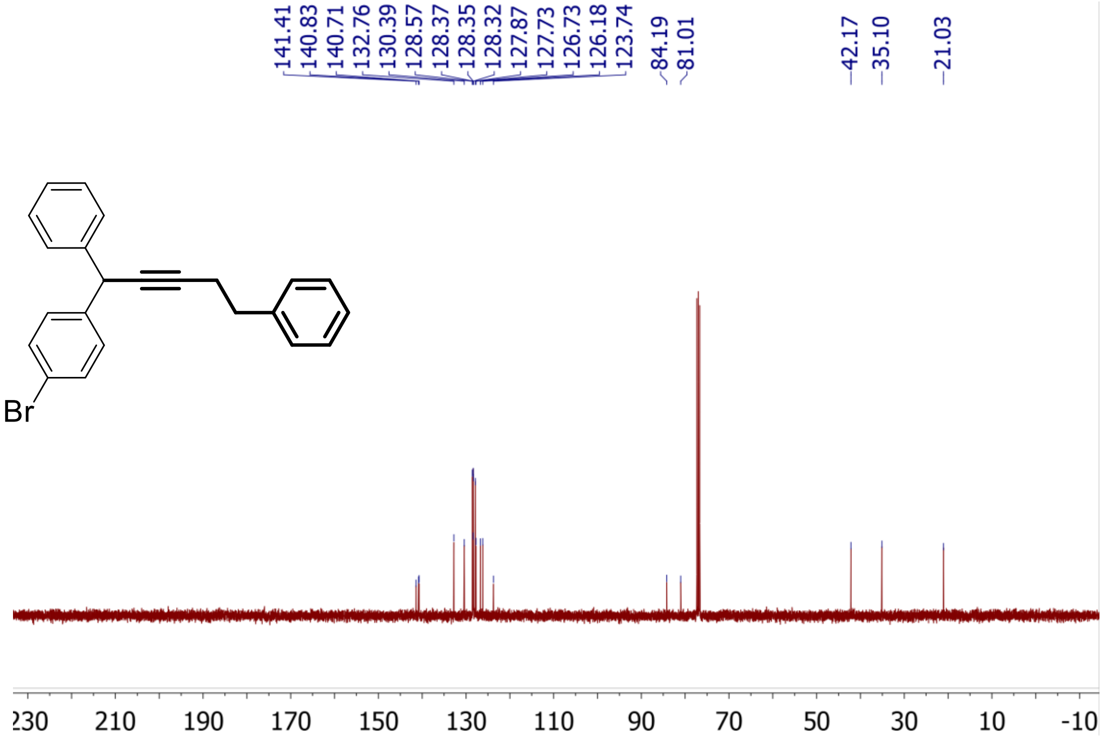

Figure S126. ¹H NMR spectrum of 3-methyl-1,3-diphenylindolin-2-one (73).

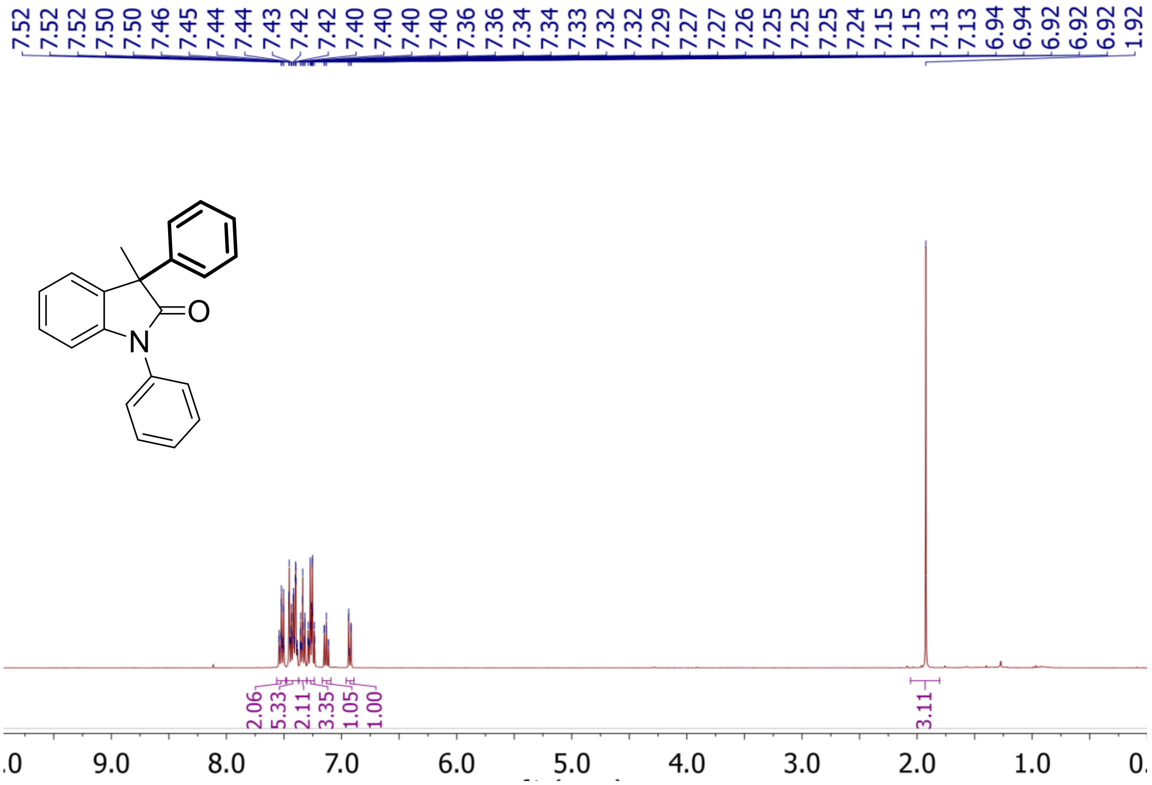

Figure S127. ¹³C NMR spectrum of 3-methyl-1,3-diphenylindolin-2-one (73).

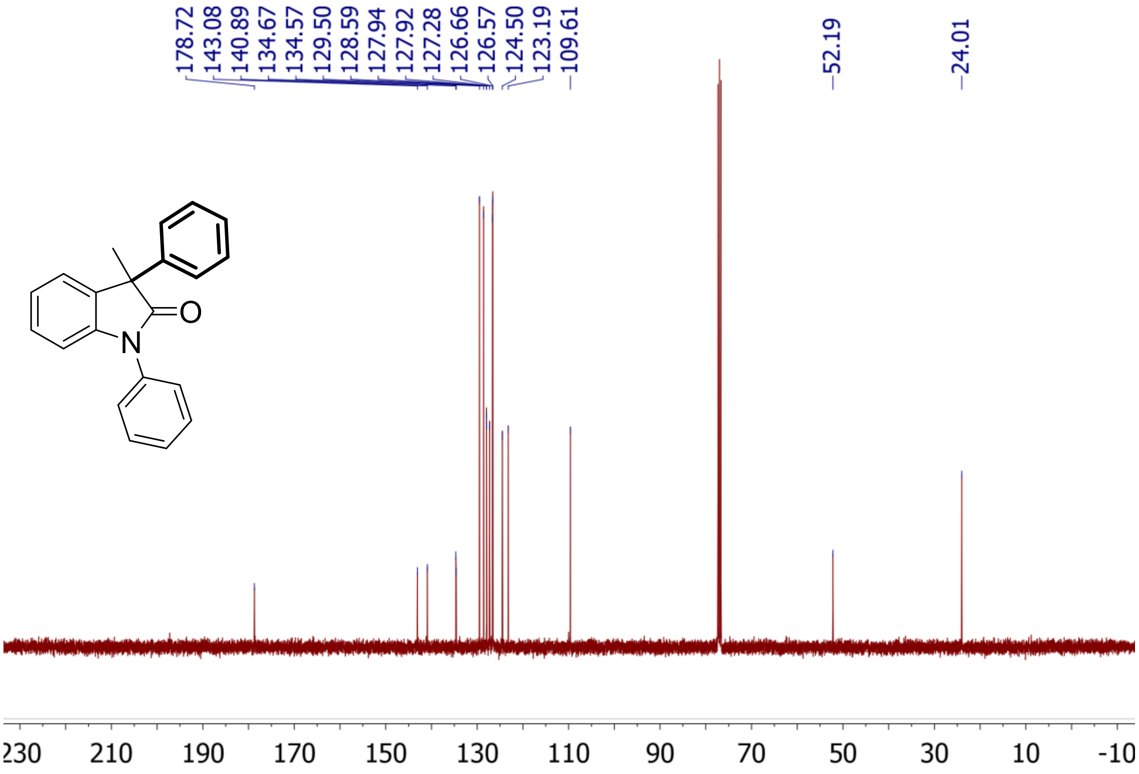

Figure S128. ^{1}H NMR spectrum of (*E*)-3-methyl-1-phenyl-3-styrylindolin-2-one (74).

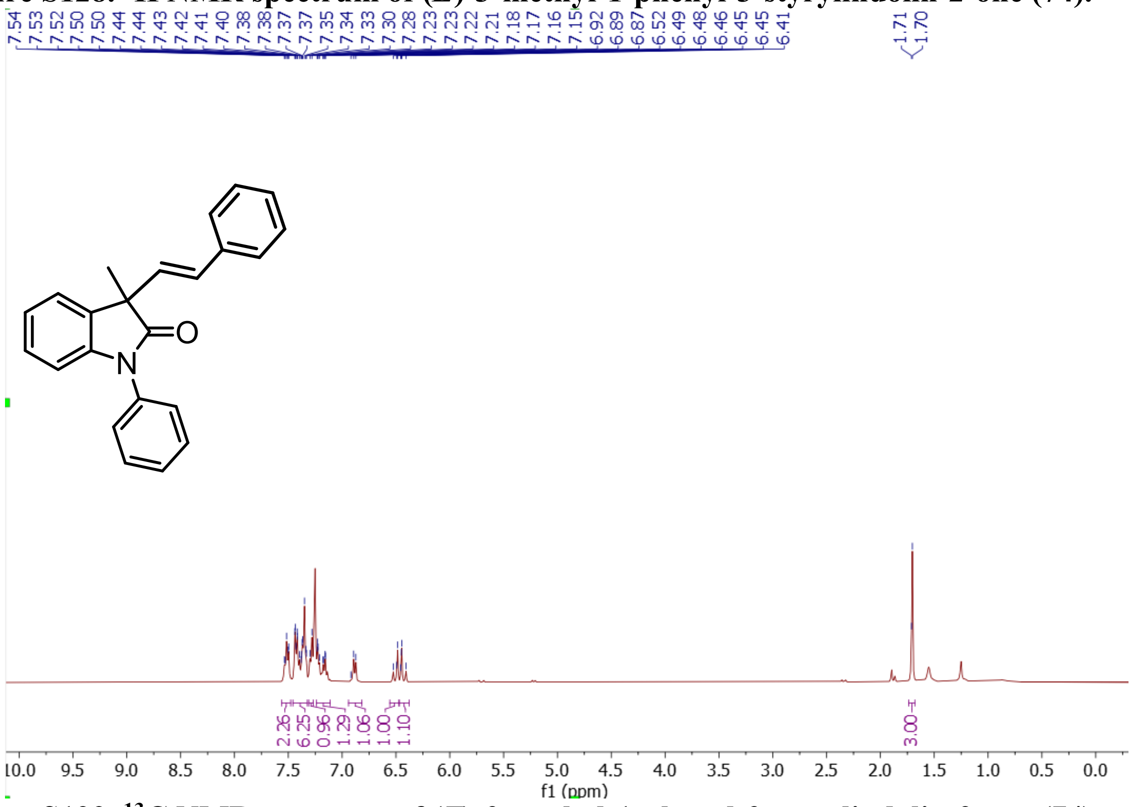

Figure S129. ^{13}C NMR spectrum of (*E*)-3-methyl-1-phenyl-3-styrylindolin-2-one (74).

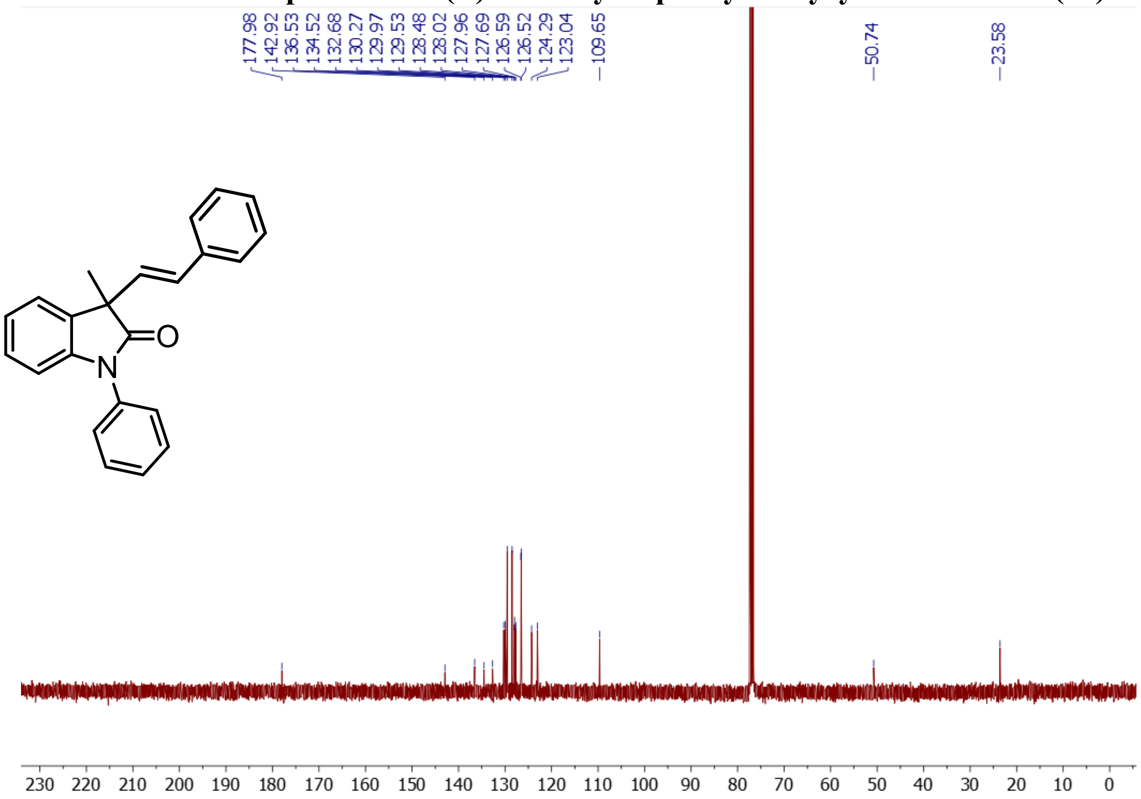

Figure S130. **^{1}H NMR spectrum of 1-(4-phenylbut-1-yn-1-yl)adamantane (75).**

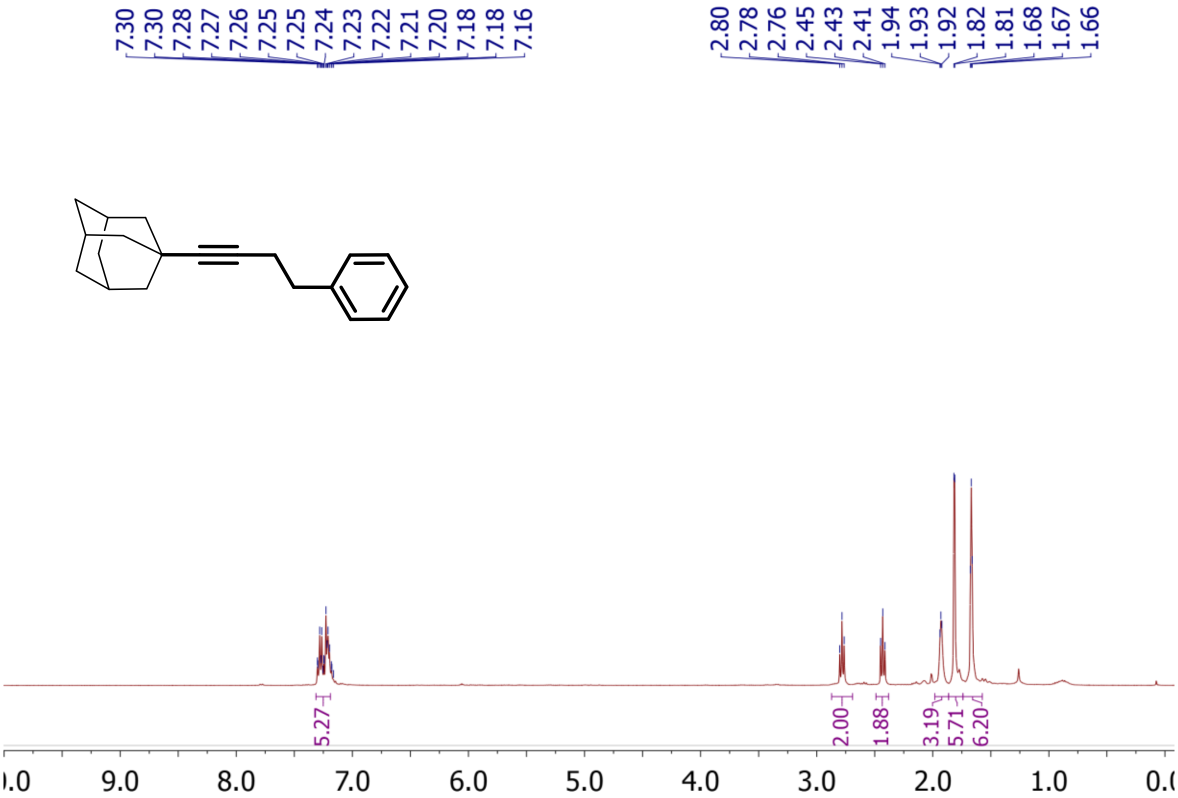

Figure S131. **^{13}C NMR spectrum of 1-(4-phenylbut-1-yn-1-yl)adamantane (75).**

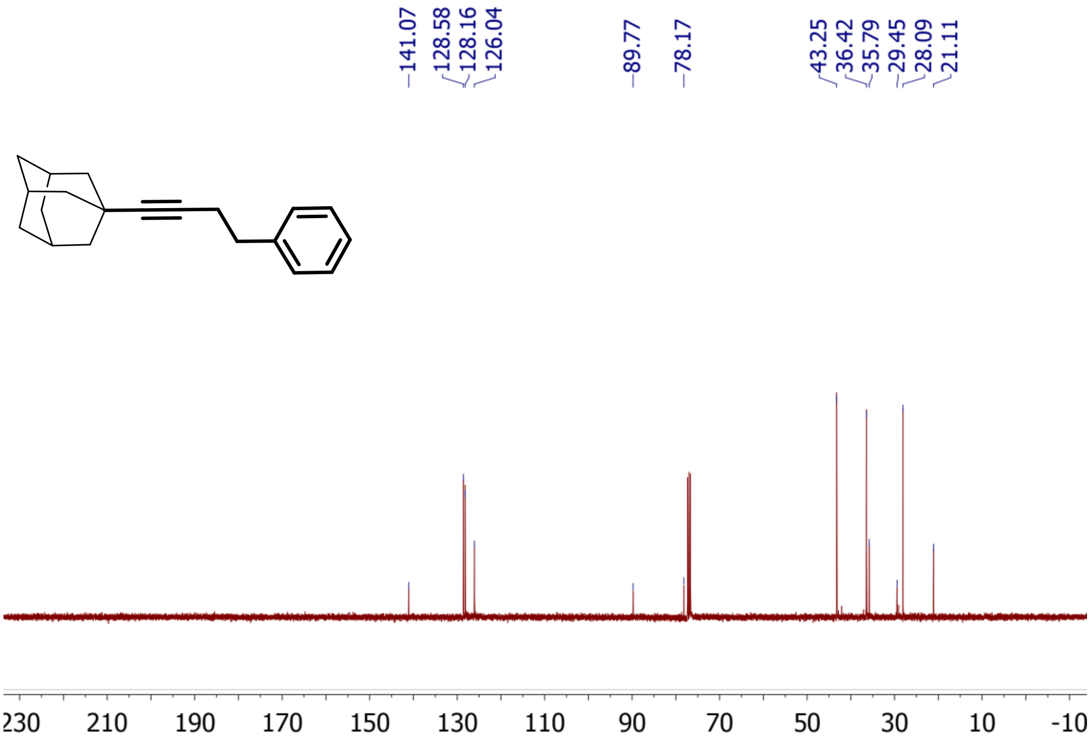

Figure S132. ^{1}**H NMR spectrum of 1-((*E*)-styryl)adamantane (76).**

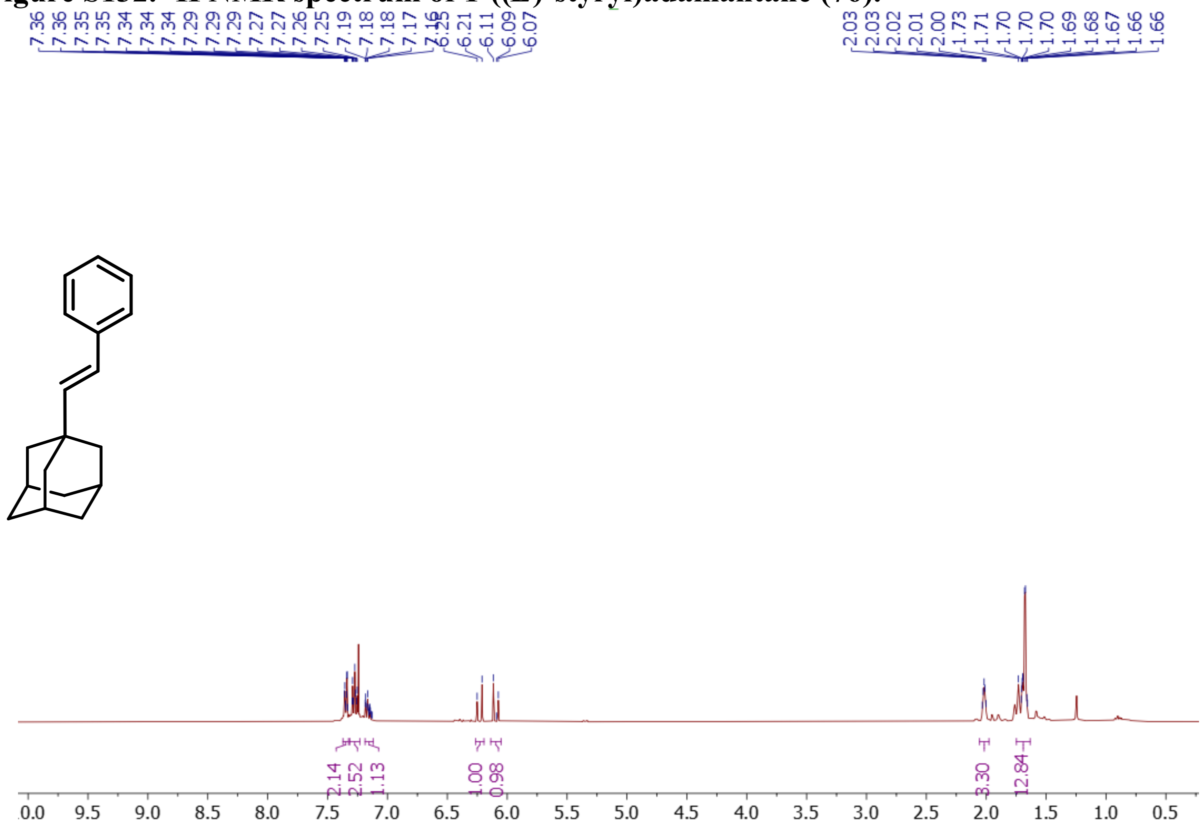

Figure S133. ^{13}**C NMR spectrum of 1-((*E*)-styryl)adamantane (76).**

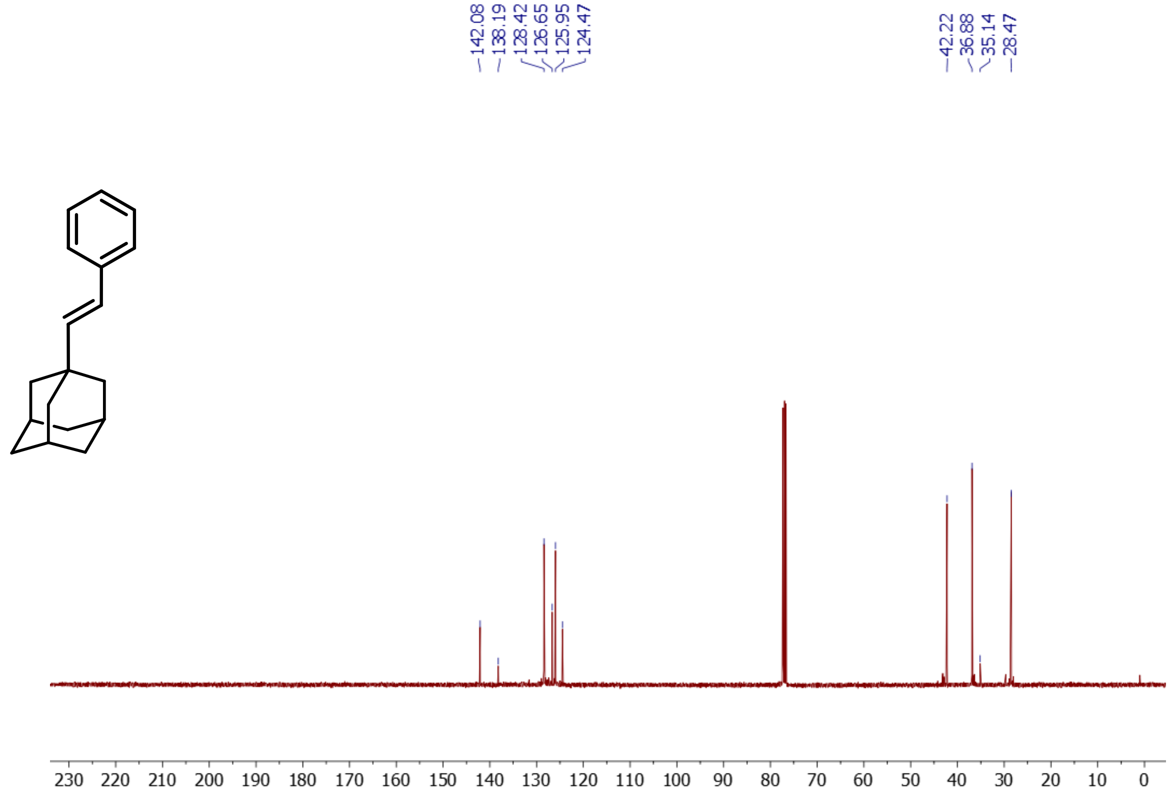

Figure S134. ^{1}**H NMR spectrum of 2,2-diphenylpropanenitrile (77).**

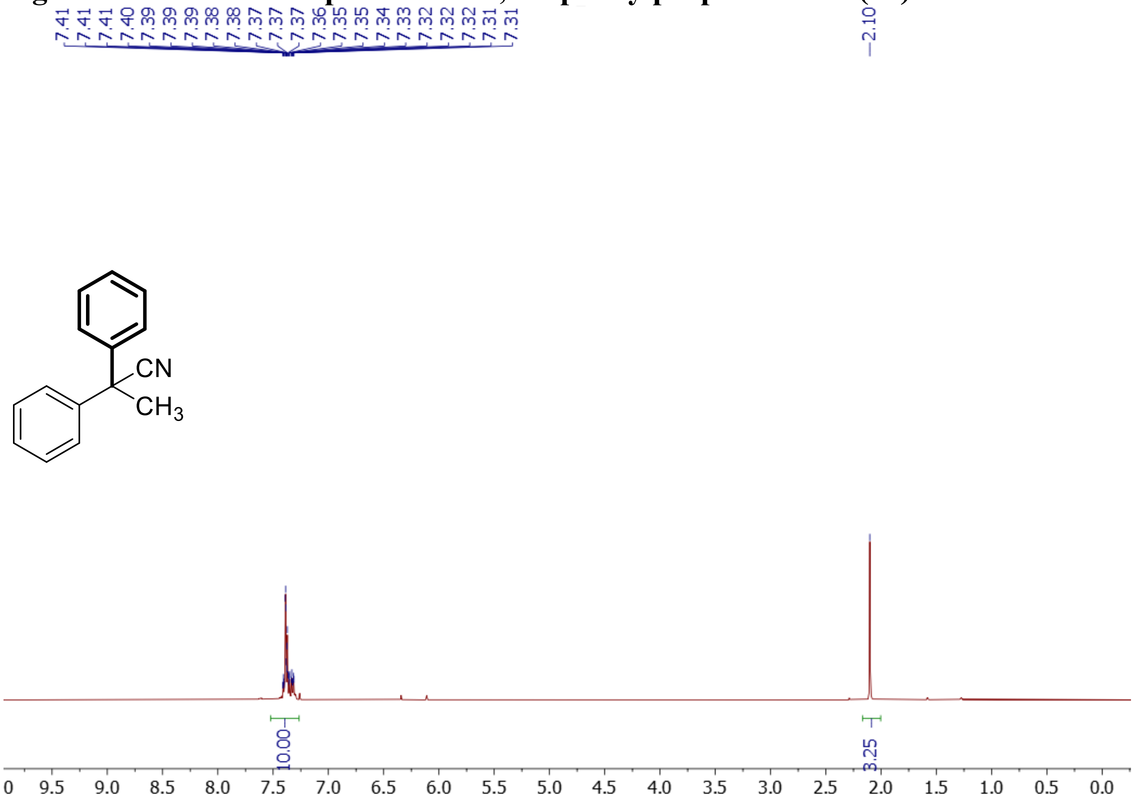

Figure S135. ^{13}**C NMR spectrum of 2,2-diphenylpropanenitrile (77).**

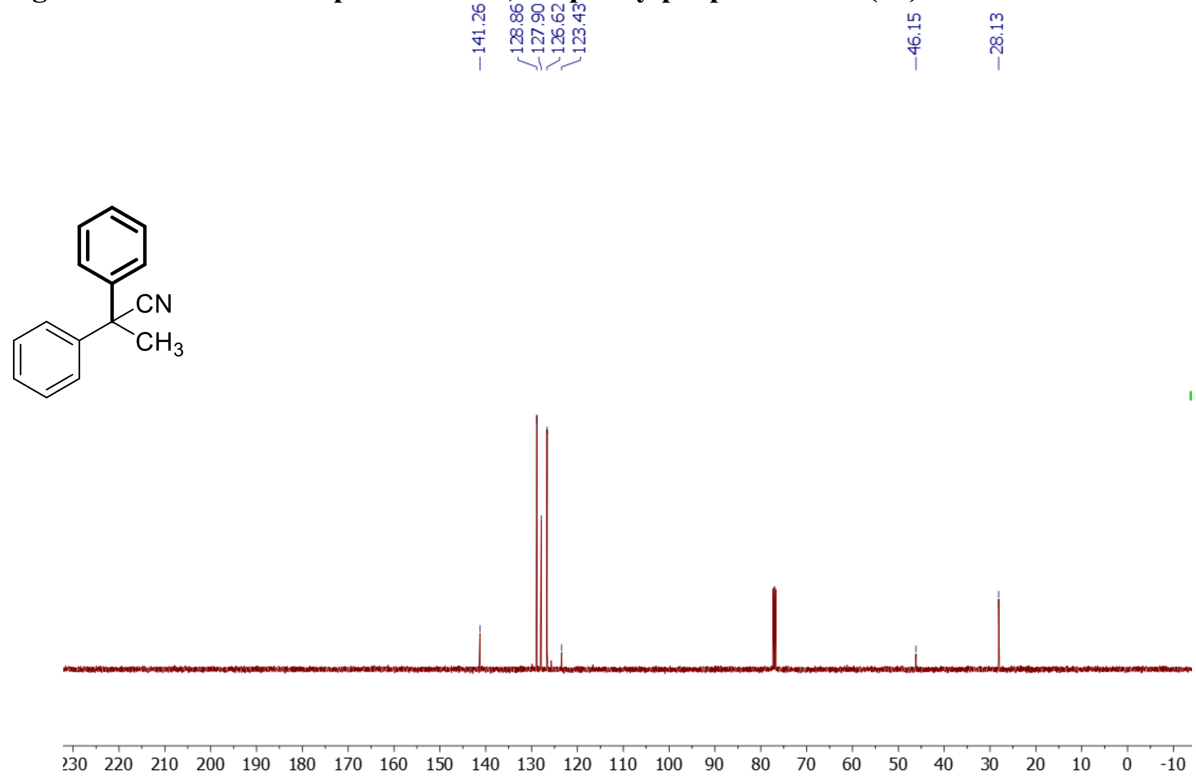

Figure S136. ^{1}H NMR spectrum of octylcyclohexane (80).

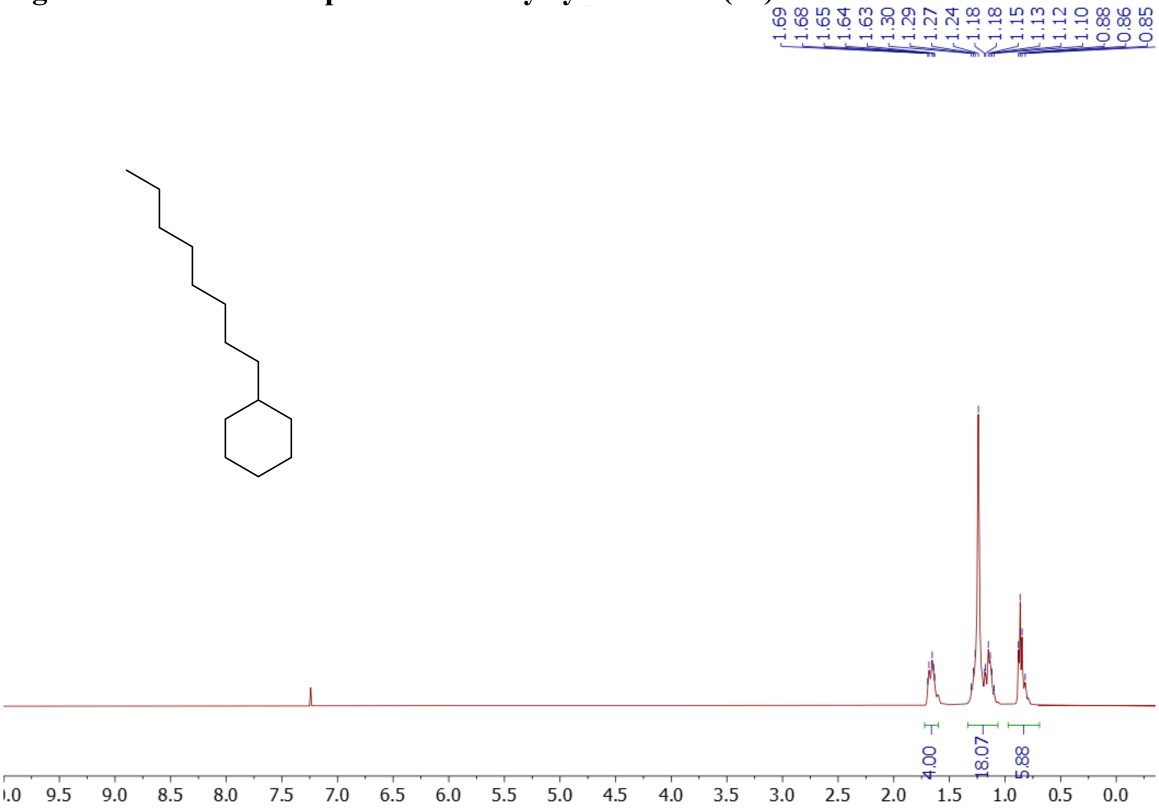

Figure S137. ^{13}C NMR spectrum of octylcyclohexane (80).

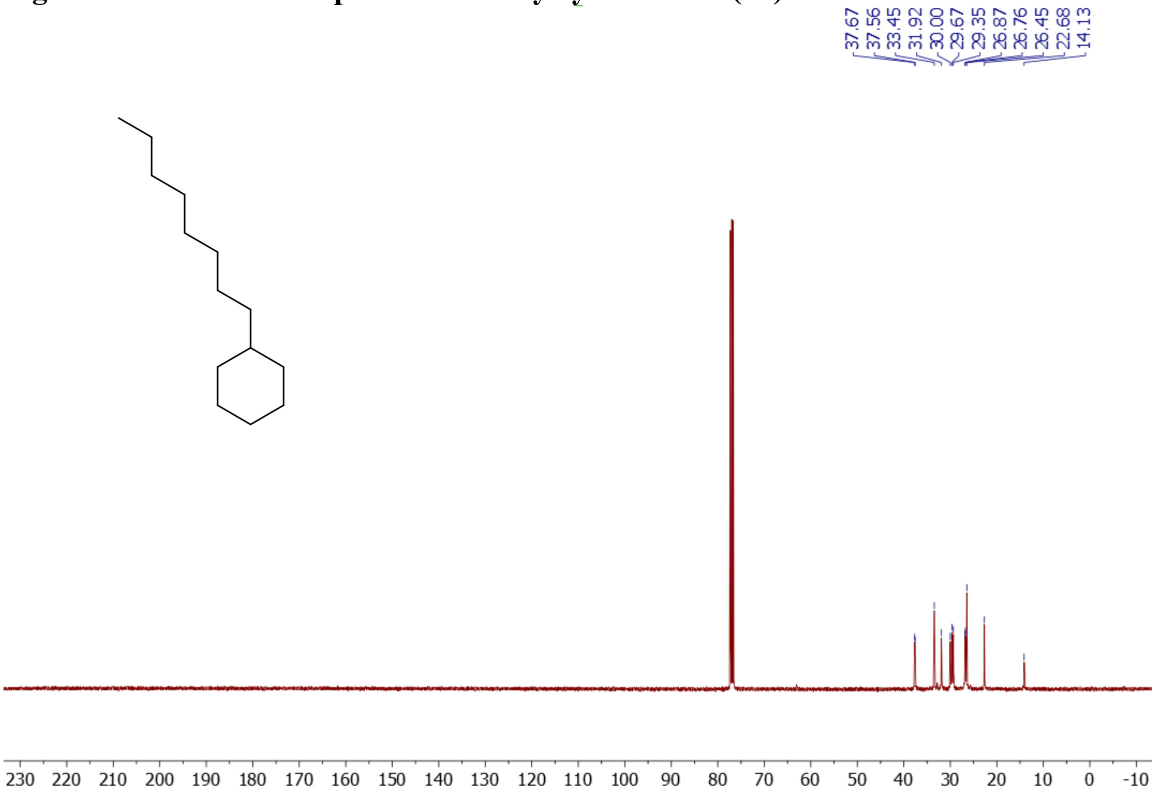

Figure S138. ^{1}H NMR spectrum of 4-ethylbenzonitrile (81).

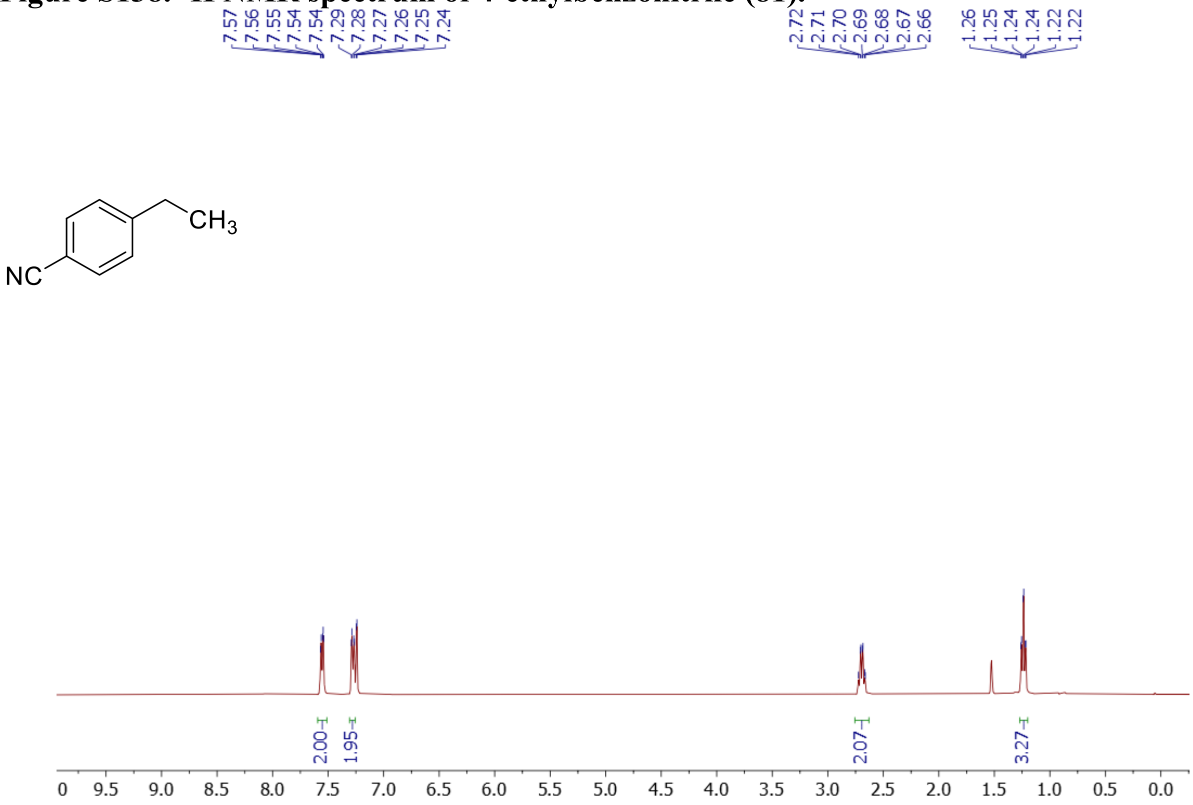

Figure S139. ^{13}C NMR spectrum of 4-ethylbenzonitrile (81).

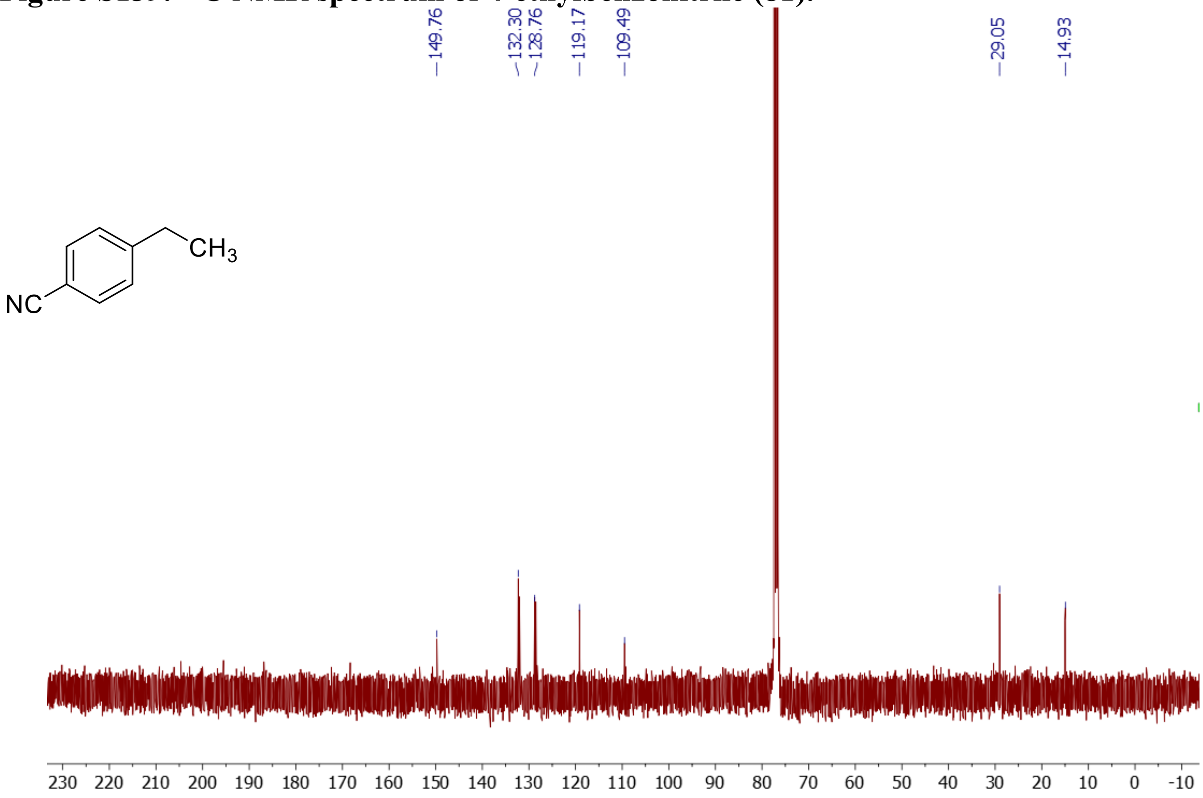

Figure S140. ^{1}H NMR spectrum of 1-benzyl-3-octylpyrrolidine (82).

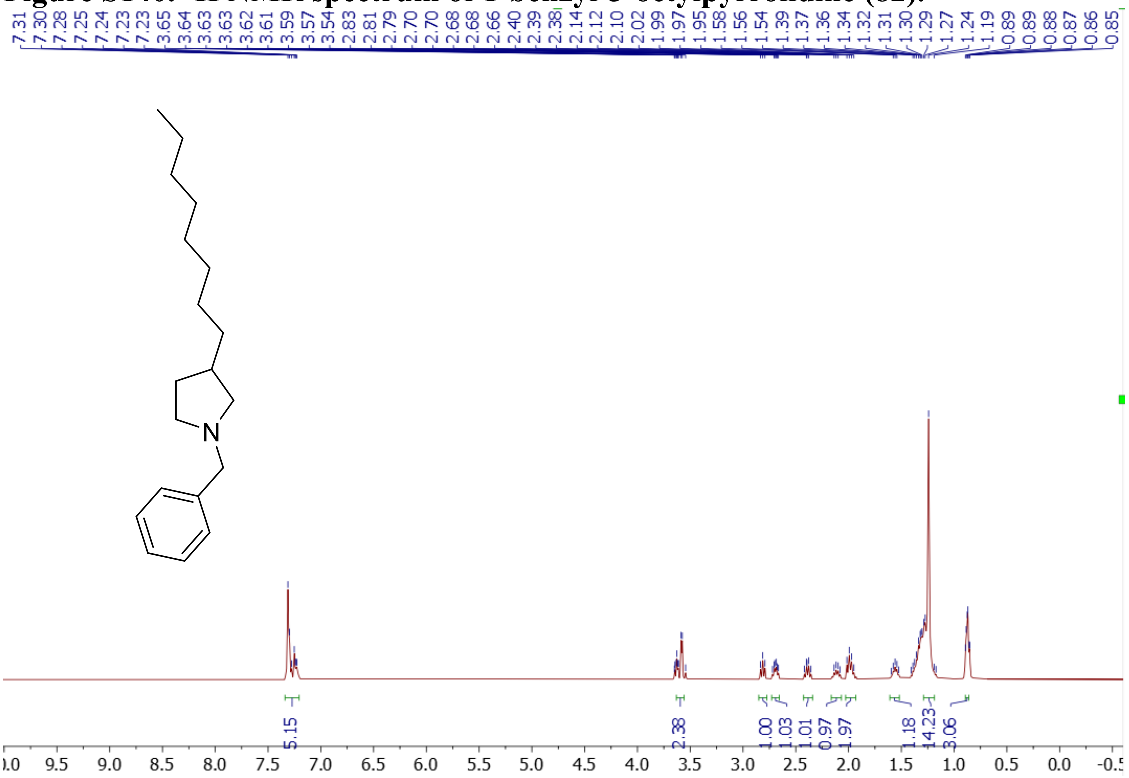

Figure S141. ^{13}C NMR spectrum of 1-benzyl-3-octylpyrrolidine (82).

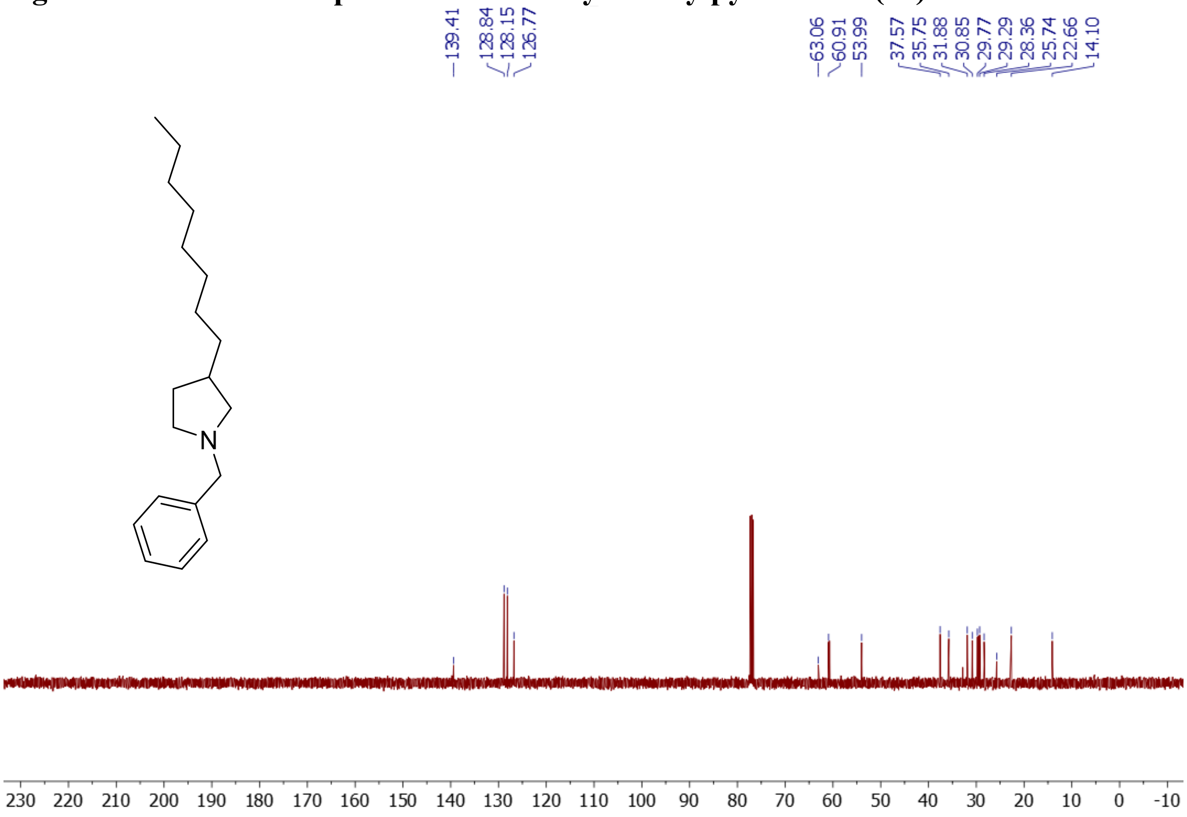

Figure S142. ^{1}H **NMR spectrum of 1-bromo-4-(1-phenylethyl)benzene (83)**

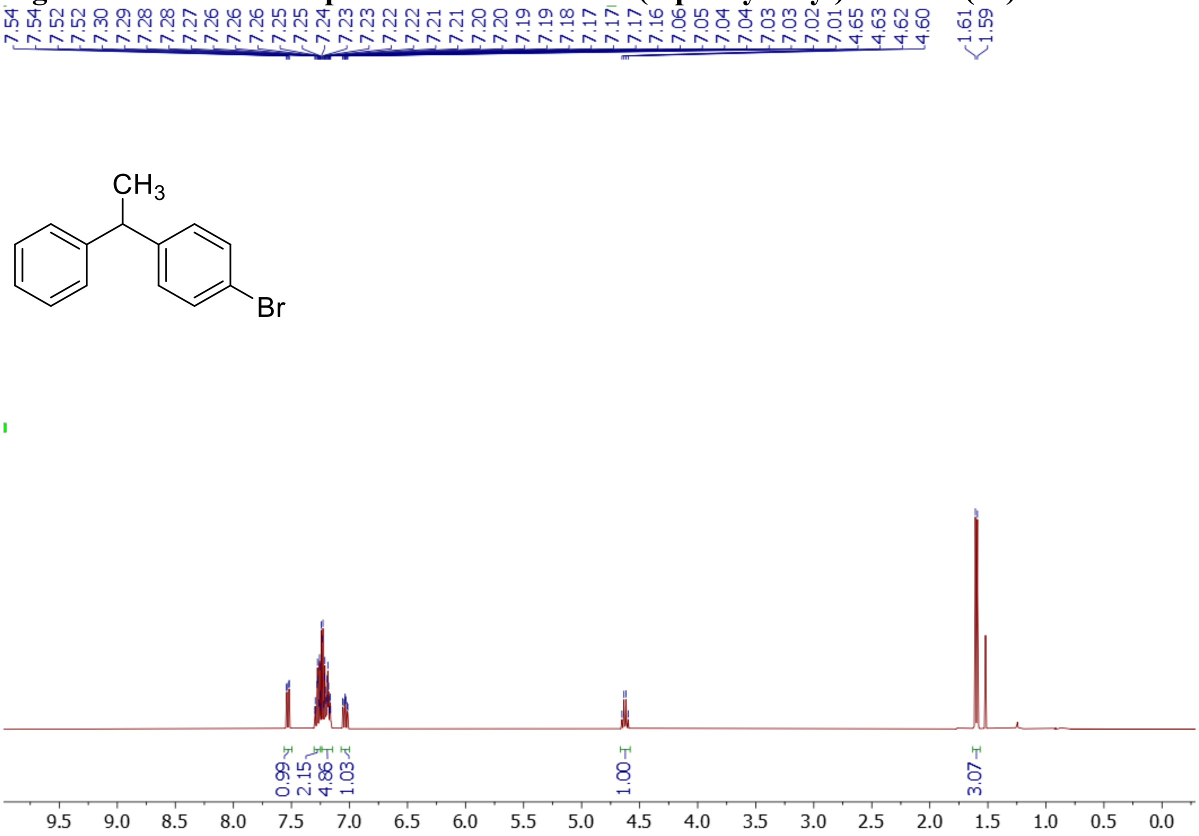

Figure S143. ^{13}C **NMR spectrum of 1-bromo-4-(1-phenylethyl)benzene (83).**

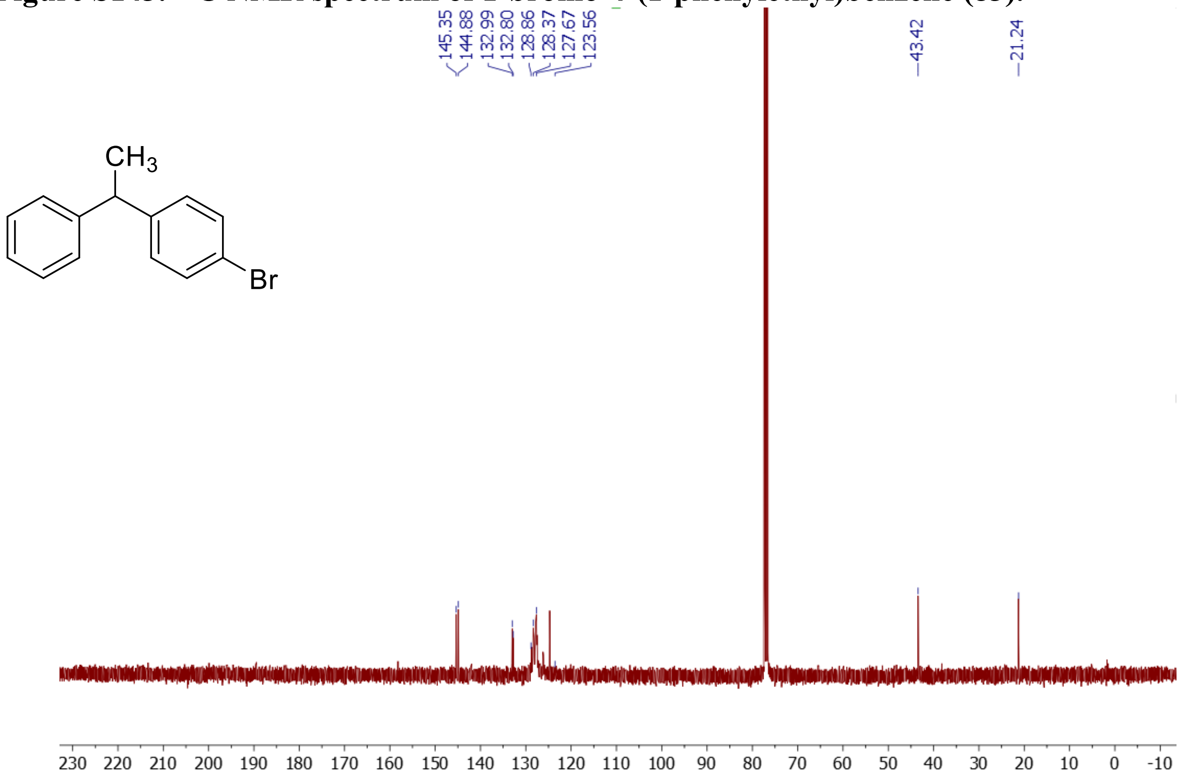

Figure S144. ^{1}H NMR spectrum of 3-methyl-3-octyl-1-phenylindolin-2-one (84).

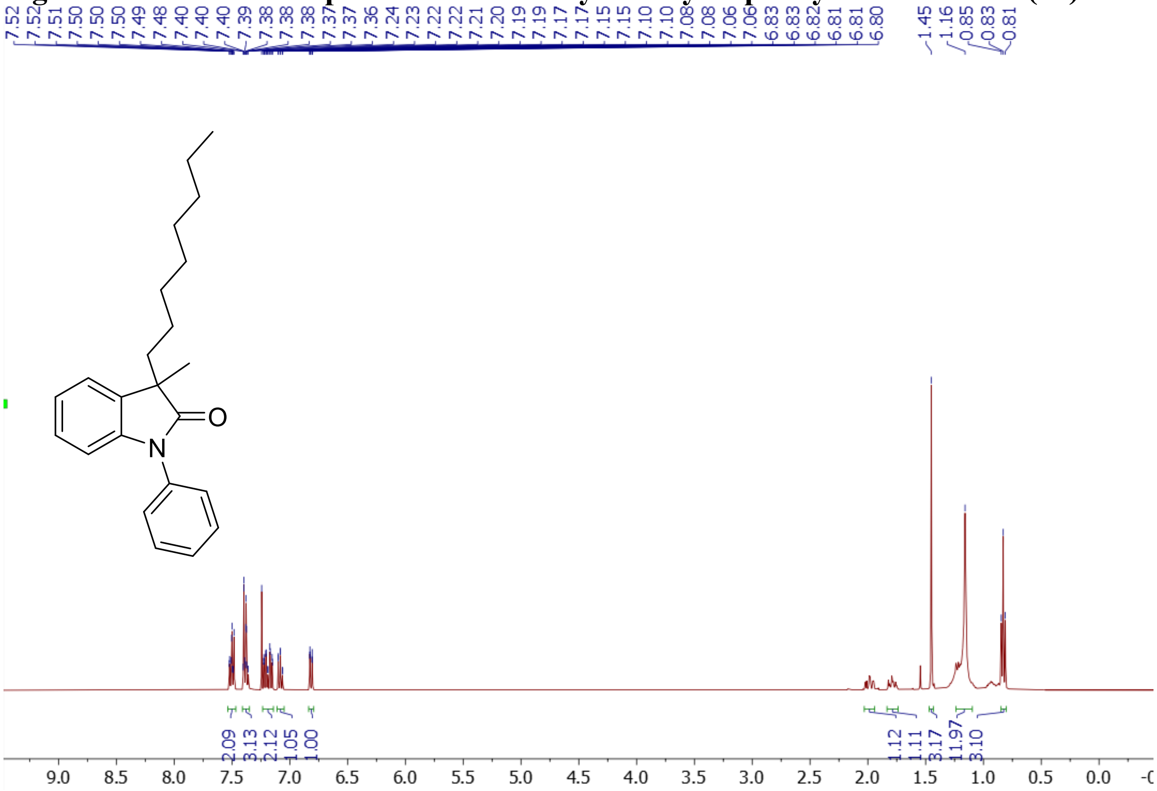

Figure S145. ^{13}C NMR spectrum of 3-methyl-3-octyl-1-phenylindolin-2-one (84).

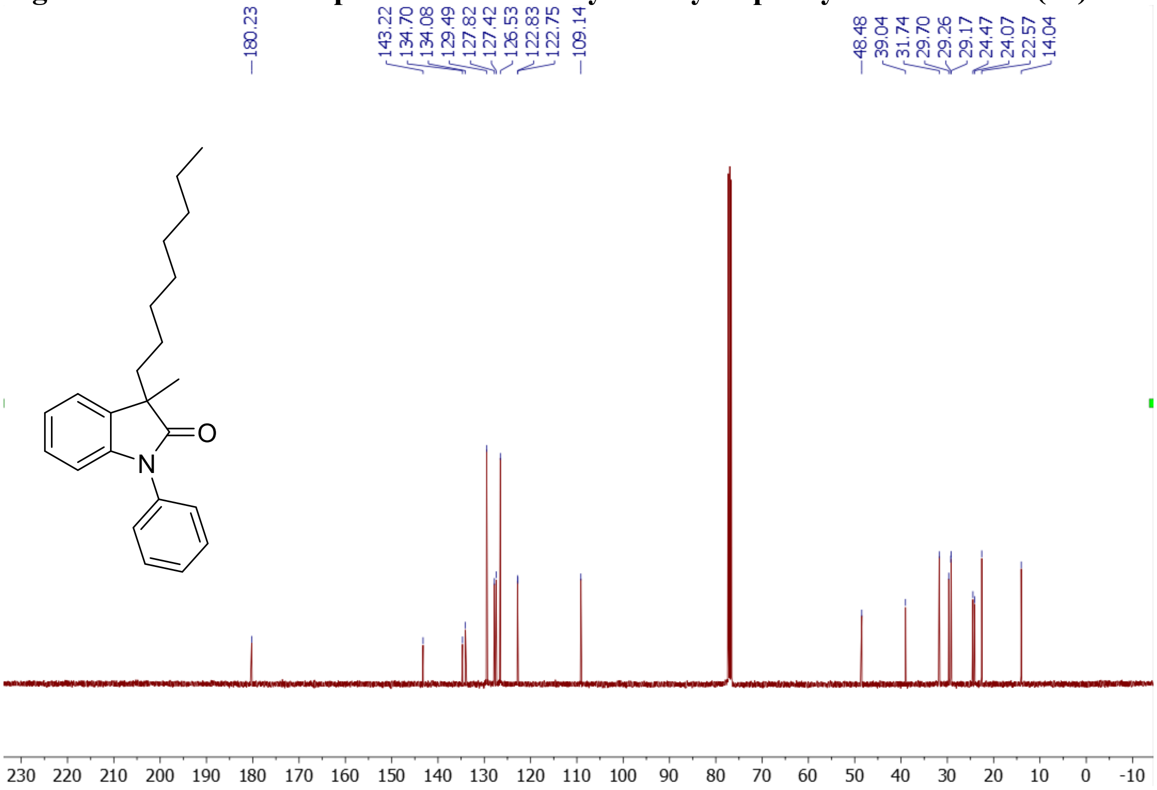

Figure S146. ^{1}H NMR spectrum of 1-bromo-7-methyloctane (85)

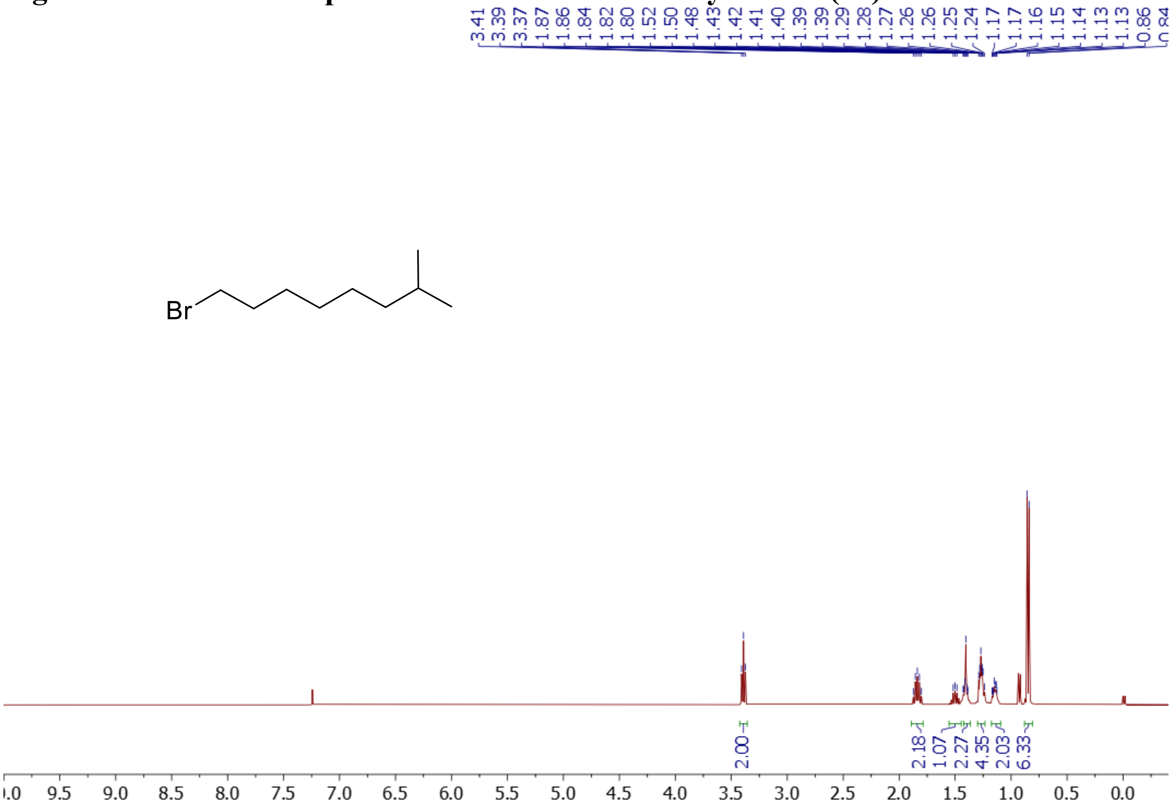

Figure S147. ^{13}C NMR spectrum of 1-bromo-7-methyloctane (85)

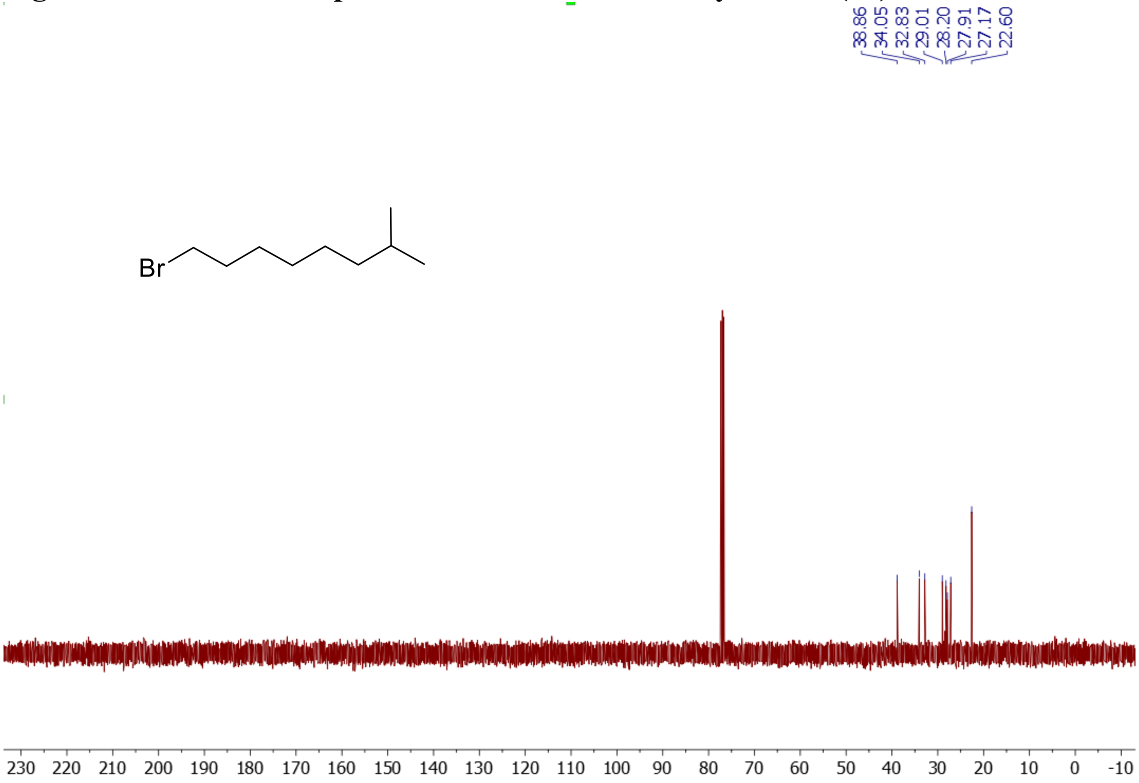

Figure S148. **^{1}H NMR spectrum of 1-bromotridecane (86).**

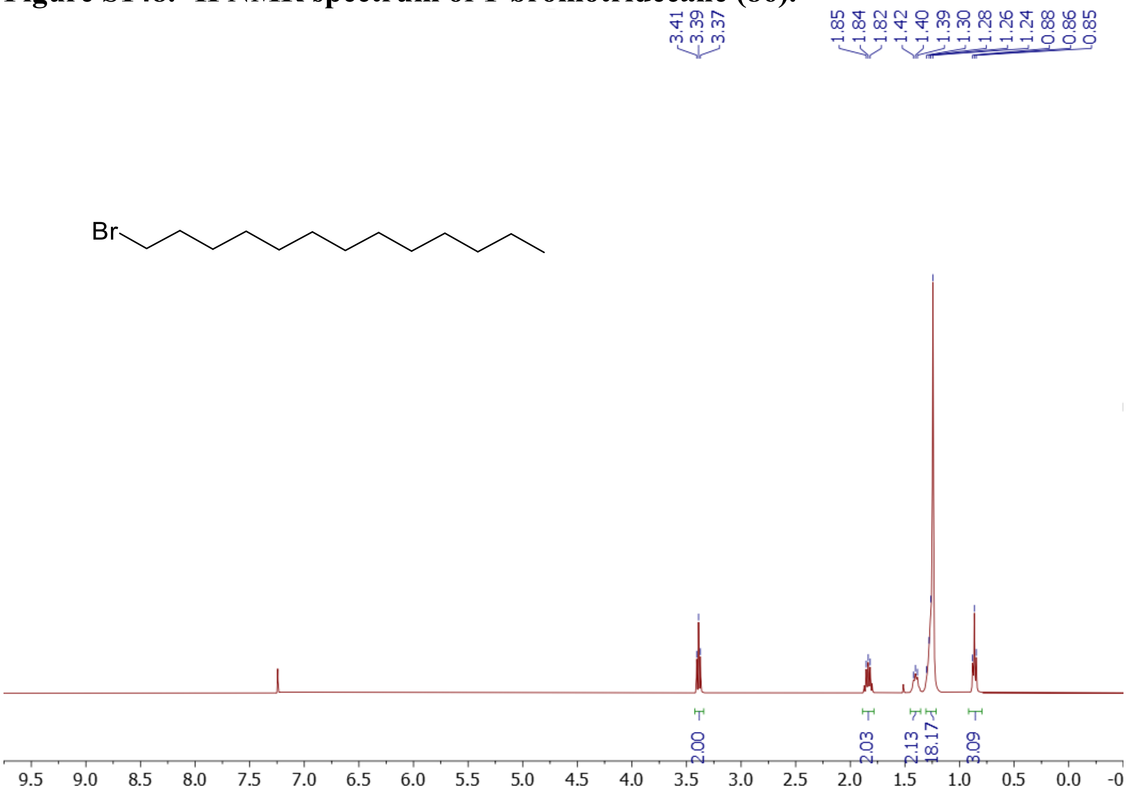

Figure S149. **^{13}C NMR spectrum of 1-bromotridecane (86).**

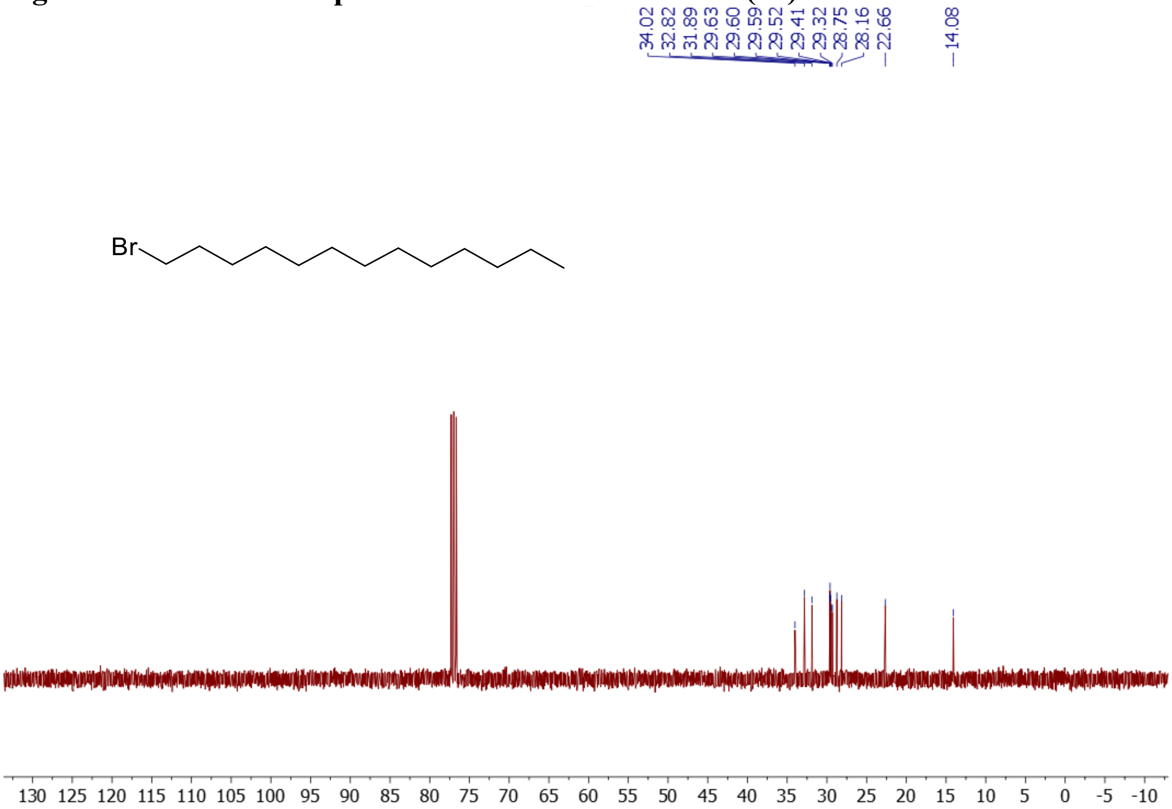

Figure S150. ^{1}H **NMR spectrum of 1-chlorotetradecane (87).**

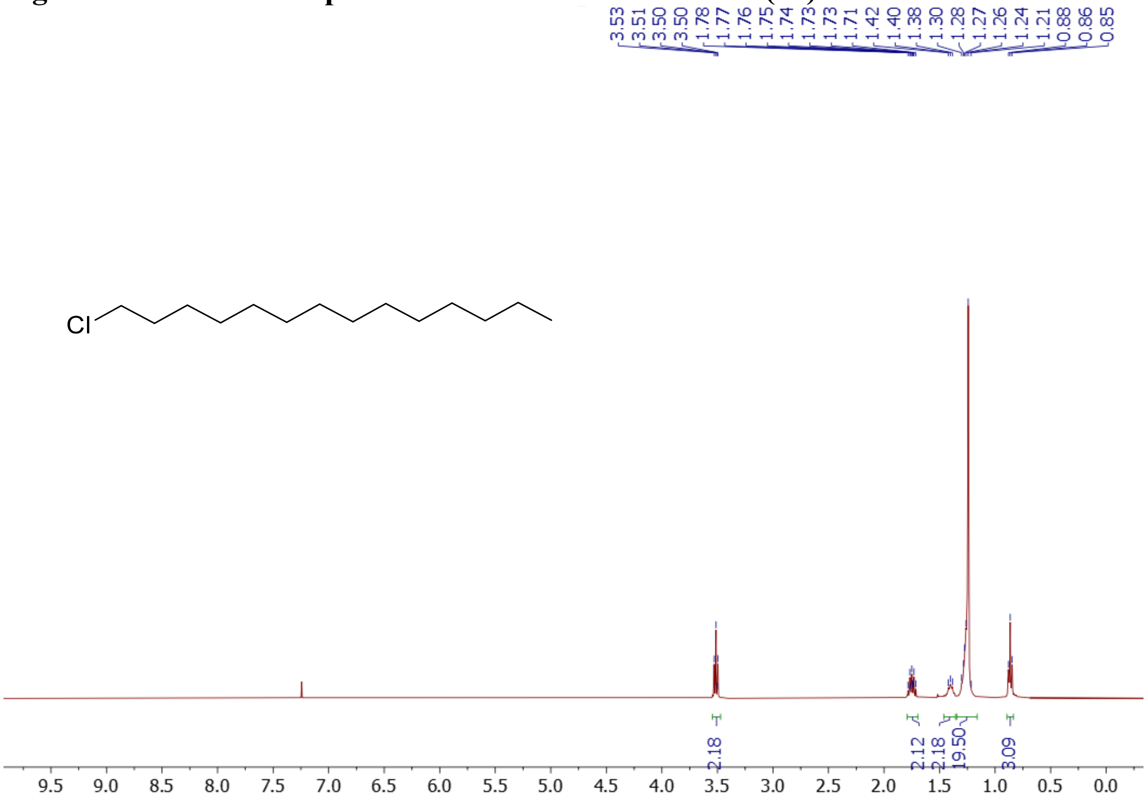

Figure S151. ^{13}C **NMR spectrum of 1-chlorotetradecane (87).**

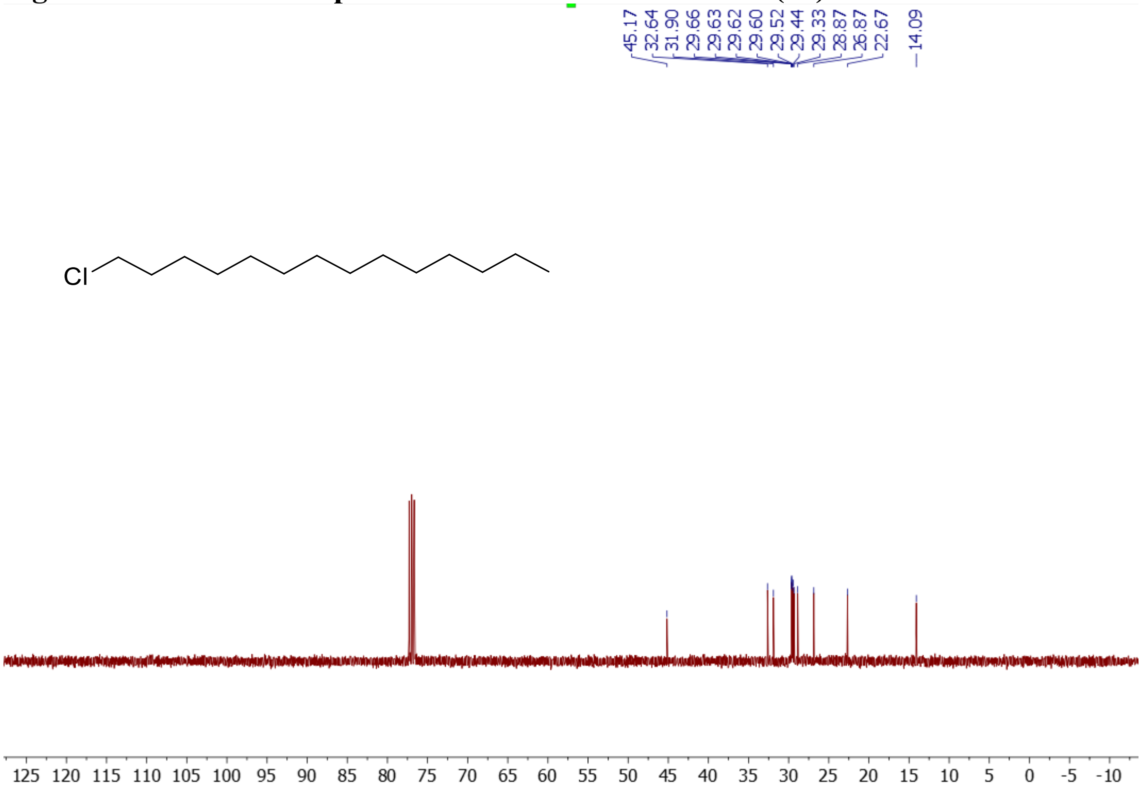

Figure S152. ^{1}H **NMR spectrum of 1-iodo-4-methylpentane (88).**

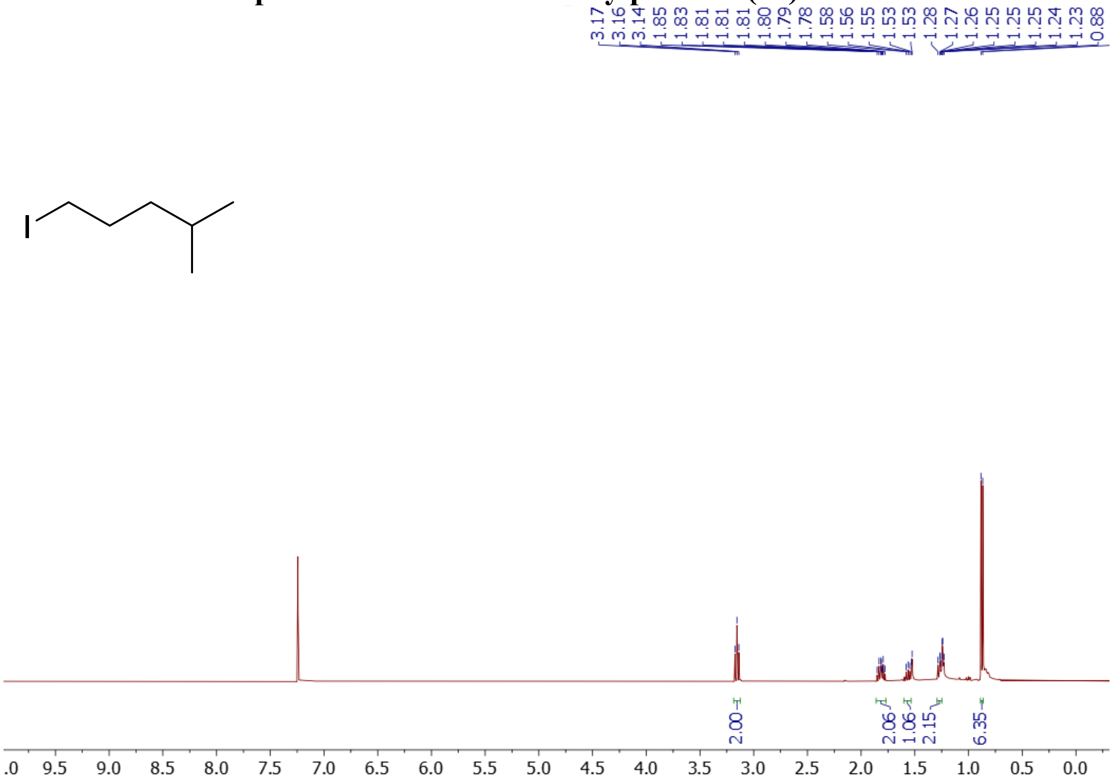

Figure S153. ^{13}C **NMR spectrum of 1-iodo-4-methylpentane (88).**

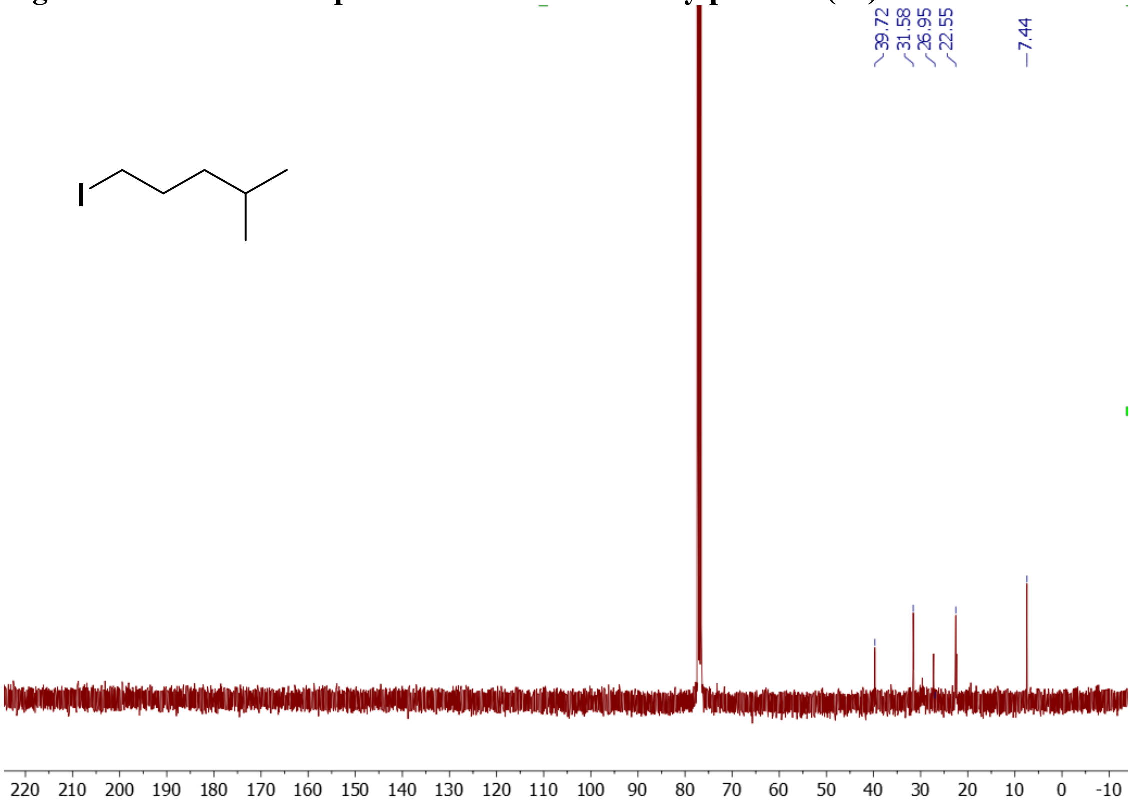

Figure S154. ^{1}**H NMR spectrum of (5R,5aR,8aR,9S)-9-methyl-5-(3,4,5-trimethoxyphenyl)-5,8,8a,9-tetrahydrofuro[3',4':6,7]naphtho[2,3-d][1,3]dioxol-6(5aH)-one (90).**

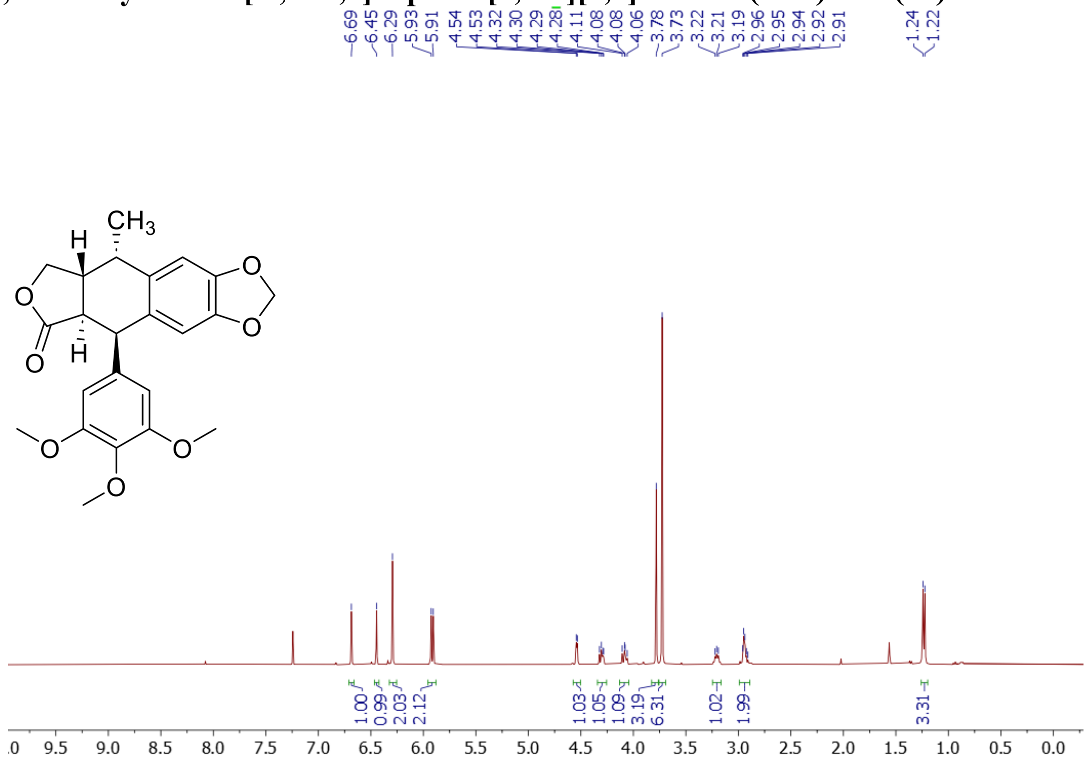

Figure S155. ^{13}**C NMR spectrum of (5R,5aR,8aR,9S)-9-methyl-5-(3,4,5-trimethoxyphenyl)-5,8,8a,9-tetrahydrofuro[3',4':6,7]naphtho[2,3-d][1,3]dioxol-6(5aH)-one (90).**

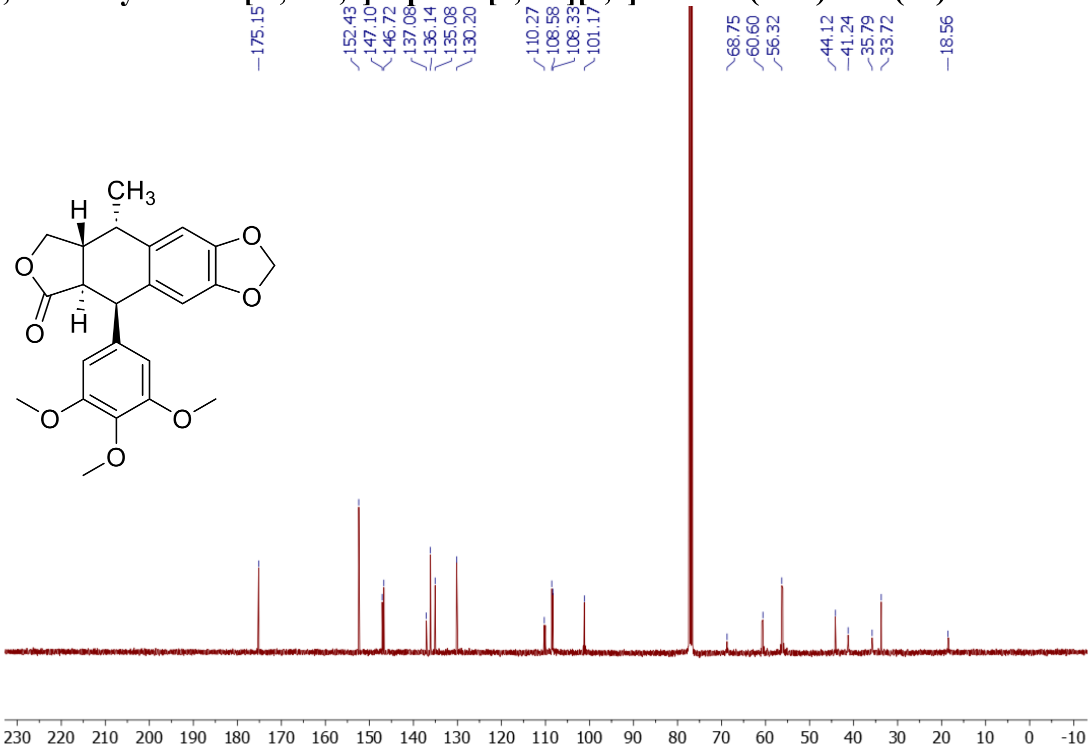

Figure S156. ^{1}H NMR spectrum of (5R,5aR,8aR,9R)-9-phenyl-5-(3,4,5-trimethoxyphenyl)-5,8,8a,9-tetrahydrofuro[3',4':6,7]naphtho[2,3-d][1,3]dioxol-6(5aH)-one (91).

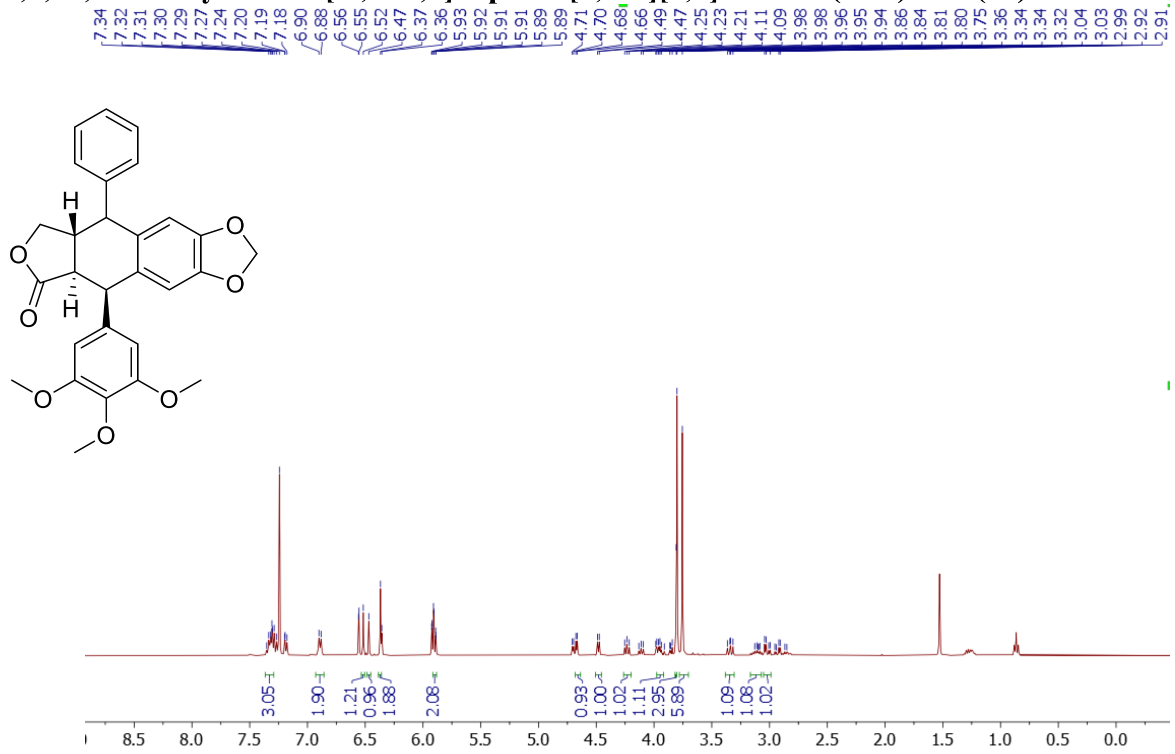

Figure S157. ^{13}C NMR spectrum of (5R,5aR,8aR,9R)-9-phenyl-5-(3,4,5-trimethoxyphenyl)-5,8,8a,9-tetrahydrofuro[3',4':6,7]naphtho[2,3-d][1,3]dioxol-6(5aH)-one (91).

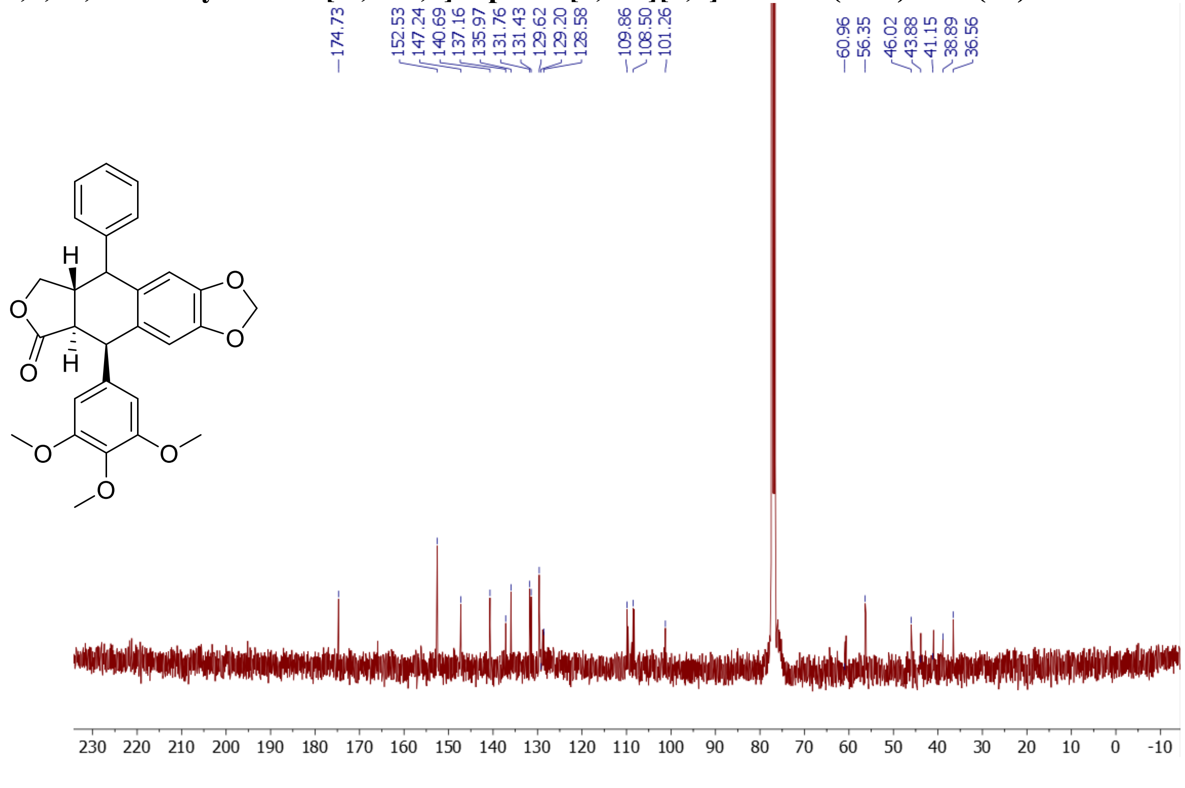

Figure S158. ^{1}H NMR spectrum of (5R,5aR,8aR)-9-(4-phenylbut-1-yn-1-yl)-5-(3,4,5-trimethoxyphenyl)-5,8,8a,9-tetrahydrofuro[3',4':6,7]naphtho[2,3-d][1,3]dioxol-6(5aH)-one (92).

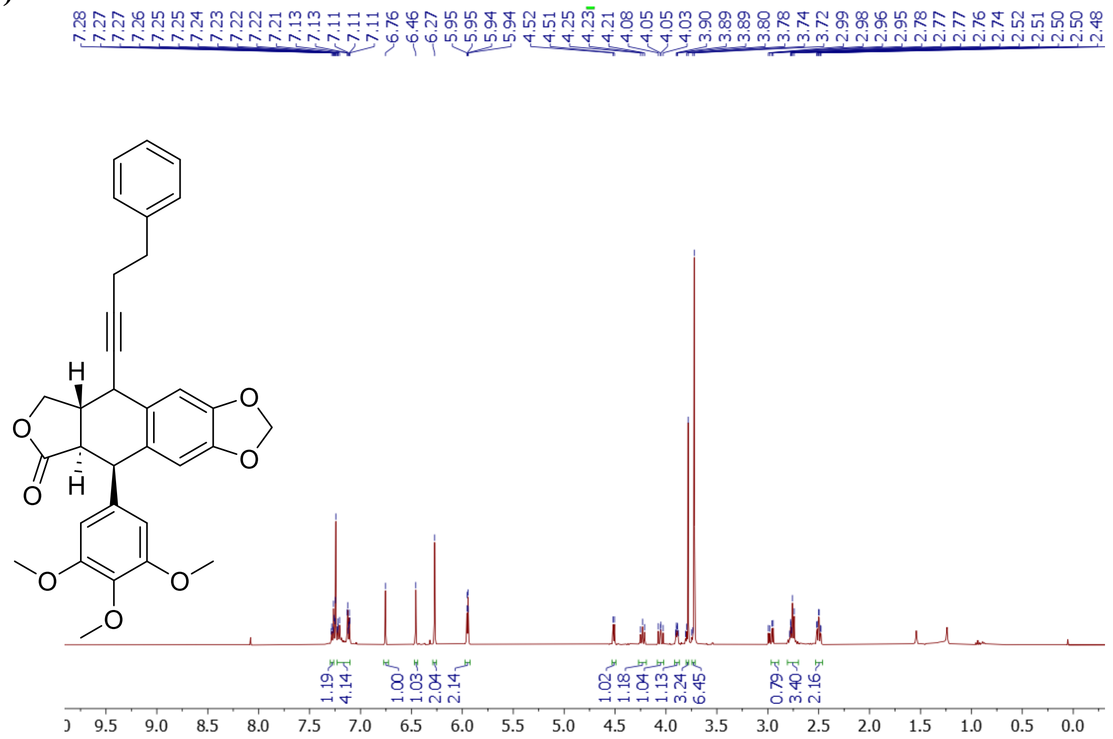

Figure S159. ^{13}C NMR spectrum of (5R,5aR,8aR)-9-(4-phenylbut-1-yn-1-yl)-5-(3,4,5-trimethoxyphenyl)-5,8,8a,9-tetrahydrofuro[3',4':6,7]naphtho[2,3-d][1,3]dioxol-6(5aH)-one (92).

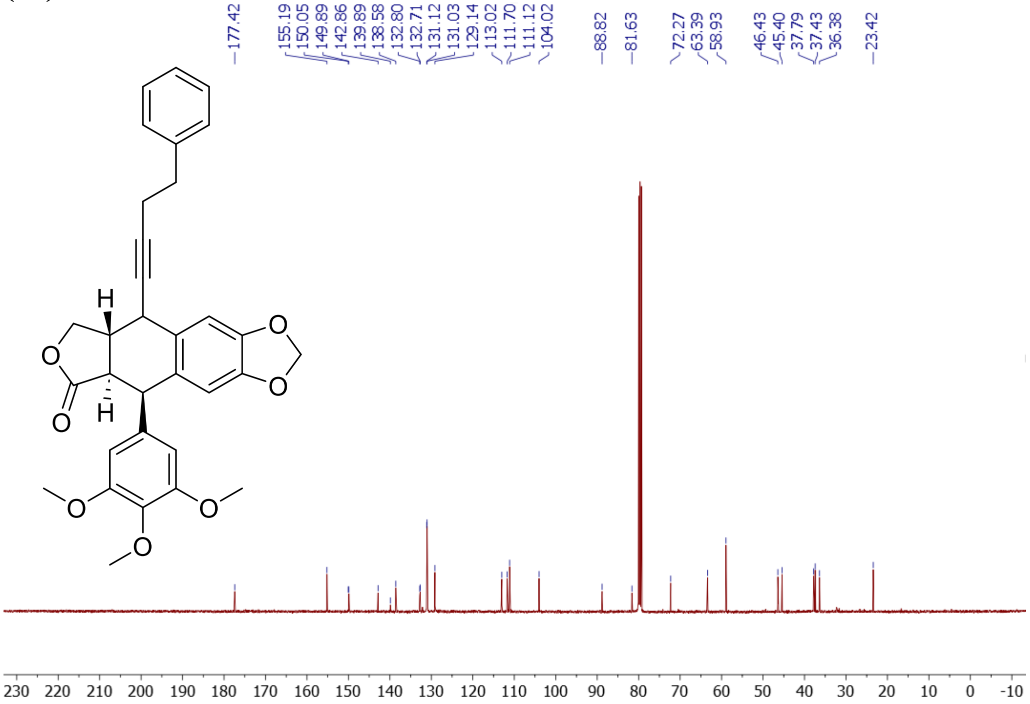

Figure S160. ^{1}H NMR spectrum of 5-((2-chlorophenyl)(4-fluorophenyl)(phenyl)methyl)pyrimidine (94).

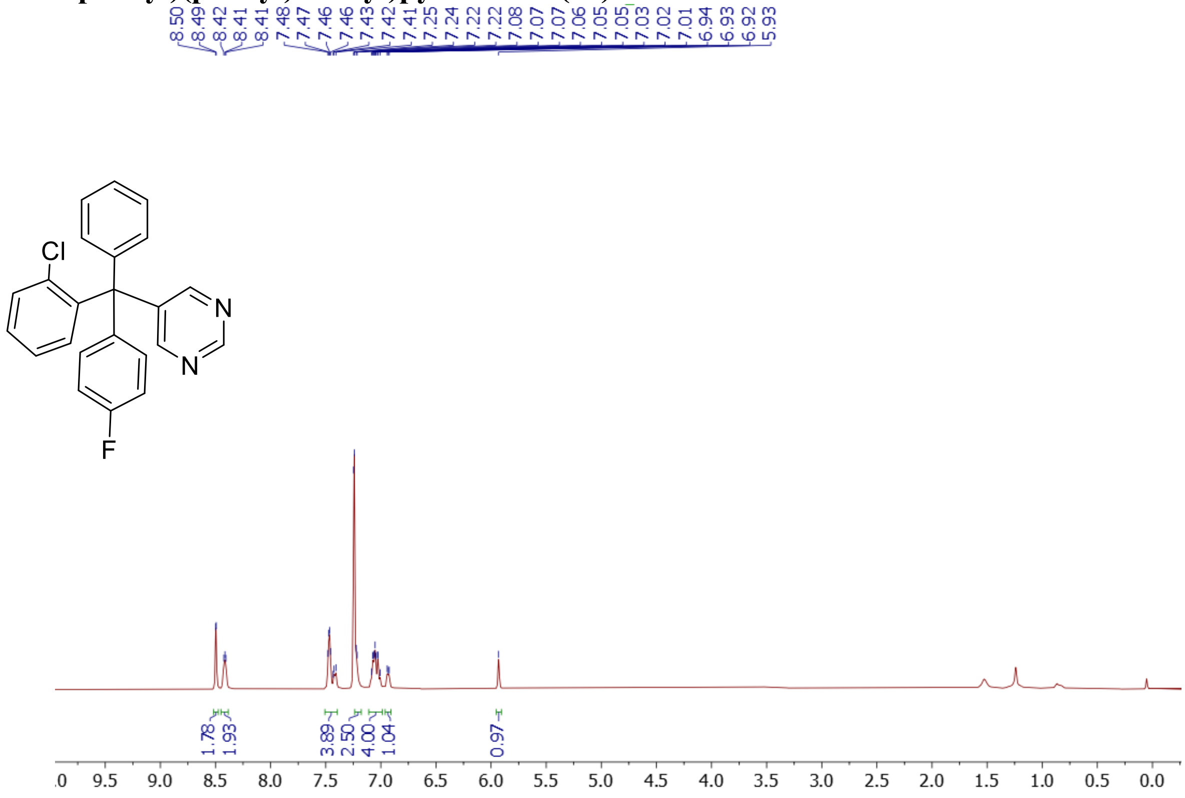

Figure S161. ^{13}C NMR spectrum of 5-((2-chlorophenyl)(4-fluorophenyl)(phenyl)methyl)pyrimidine (94).

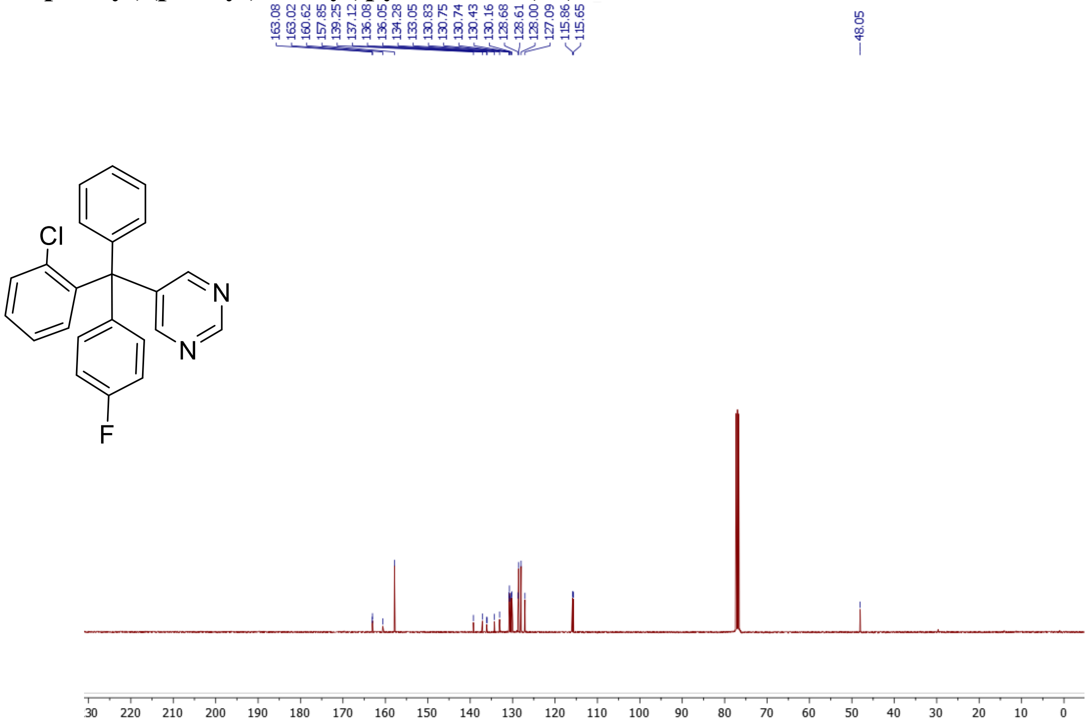

Figure S162. ^{19}**F NMR spectrum of 5-((2-chlorophenyl)(4-fluorophenyl)(phenyl)methyl)pyrimidine (94).**

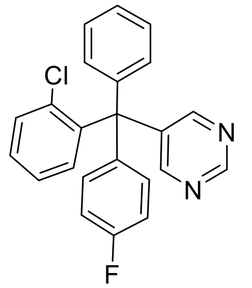

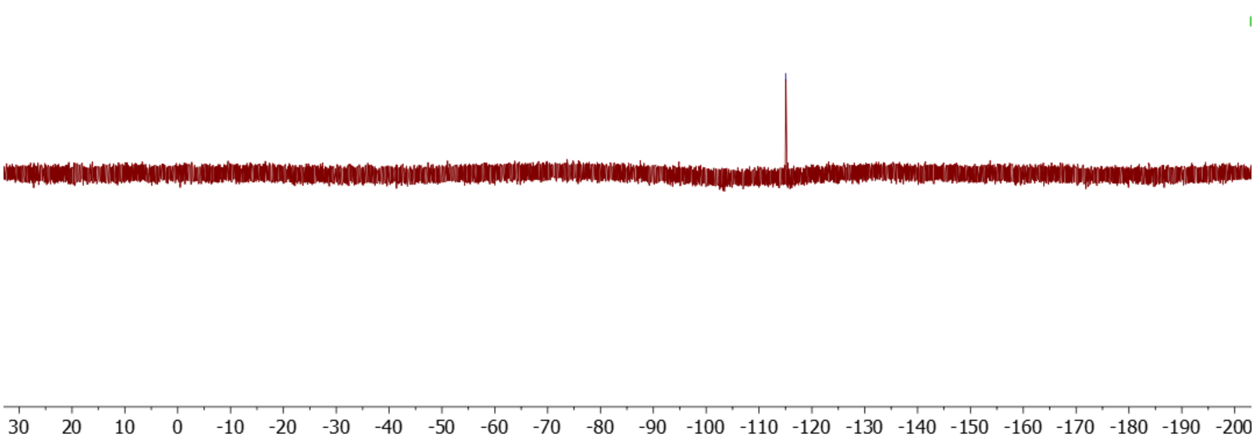

Figure S163. ^{1}H NMR spectrum of (1R,2R,3S,6S)-3-(6-methoxyquinolin-4-yl)-2-phenyl-6-vinyl-1-azabicyclo[3.2.2]nonane (96).

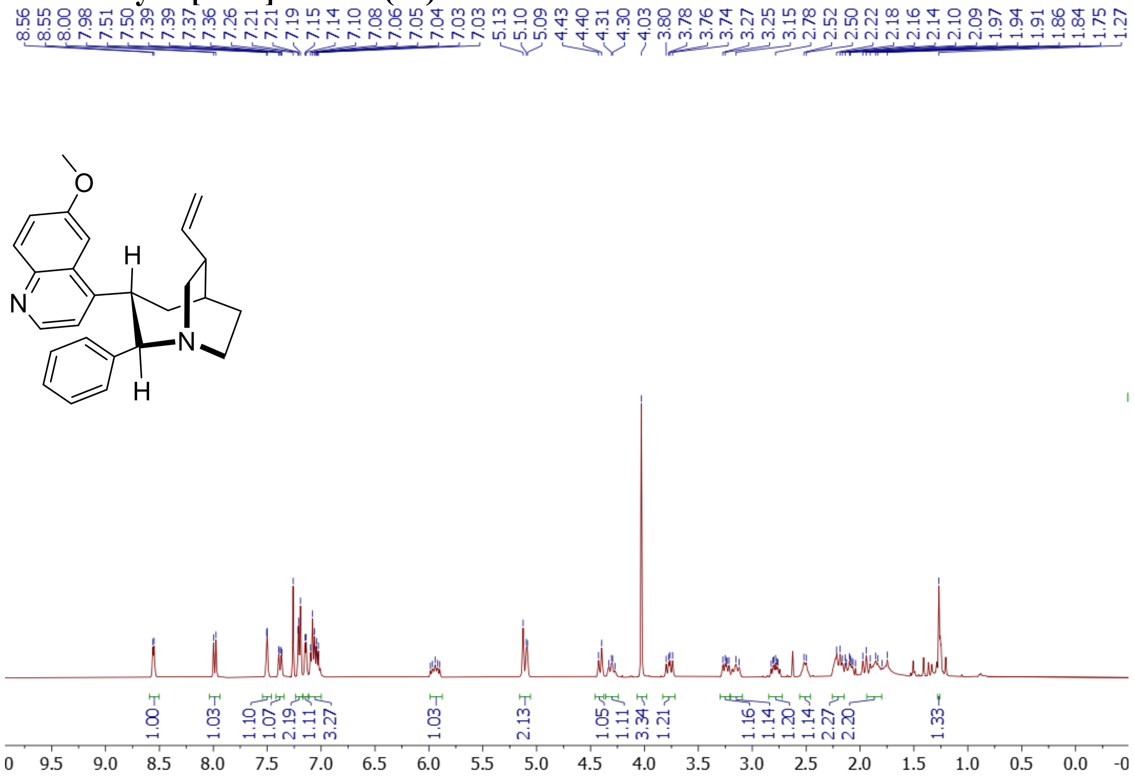

Figure S164. ^{13}C NMR spectrum of (1R,2R,3S,6S)-3-(6-methoxyquinolin-4-yl)-2-phenyl-6-vinyl-1-azabicyclo[3.2.2]nonane (96).

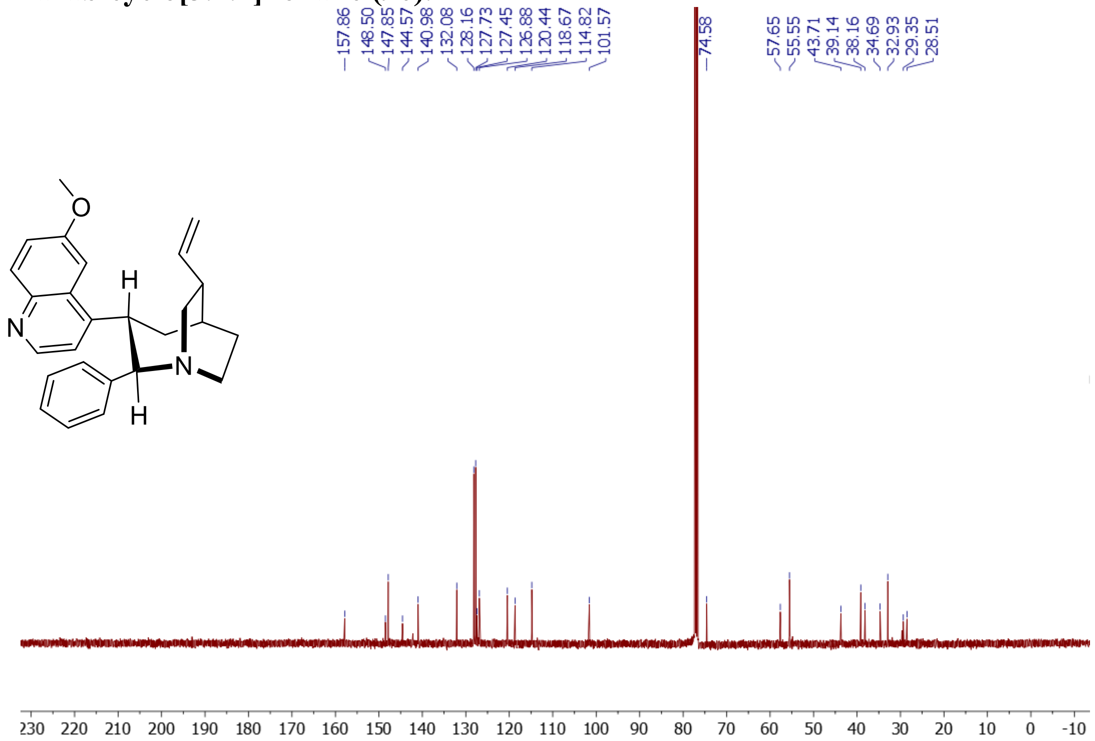

Figure S165. ^{1}H **NMR spectrum of (1R,2S,3S,6S)-3-(6-methoxyquinolin-4-yl)-2-methyl-6-vinyl-1-azabicyclo[3.2.2]nonane (97).**

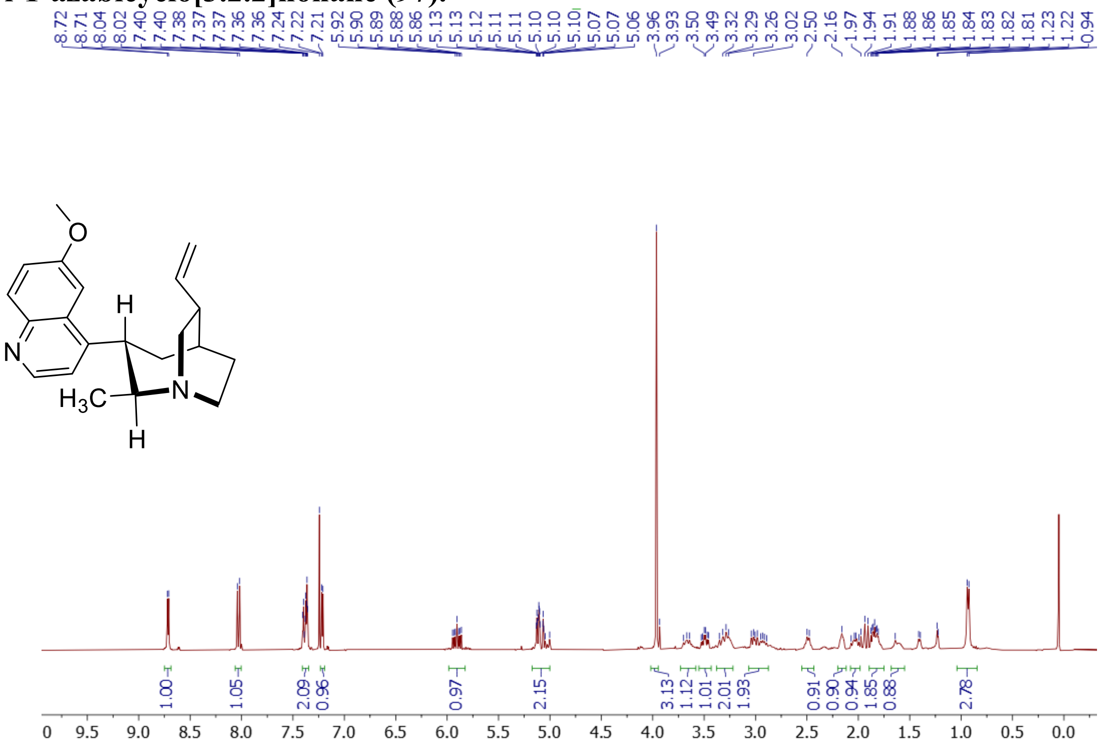

Figure S166. ^{13}C **NMR spectrum of (1R,2S,3S,6S)-3-(6-methoxyquinolin-4-yl)-2-methyl-6-vinyl-1-azabicyclo[3.2.2]nonane (97).**

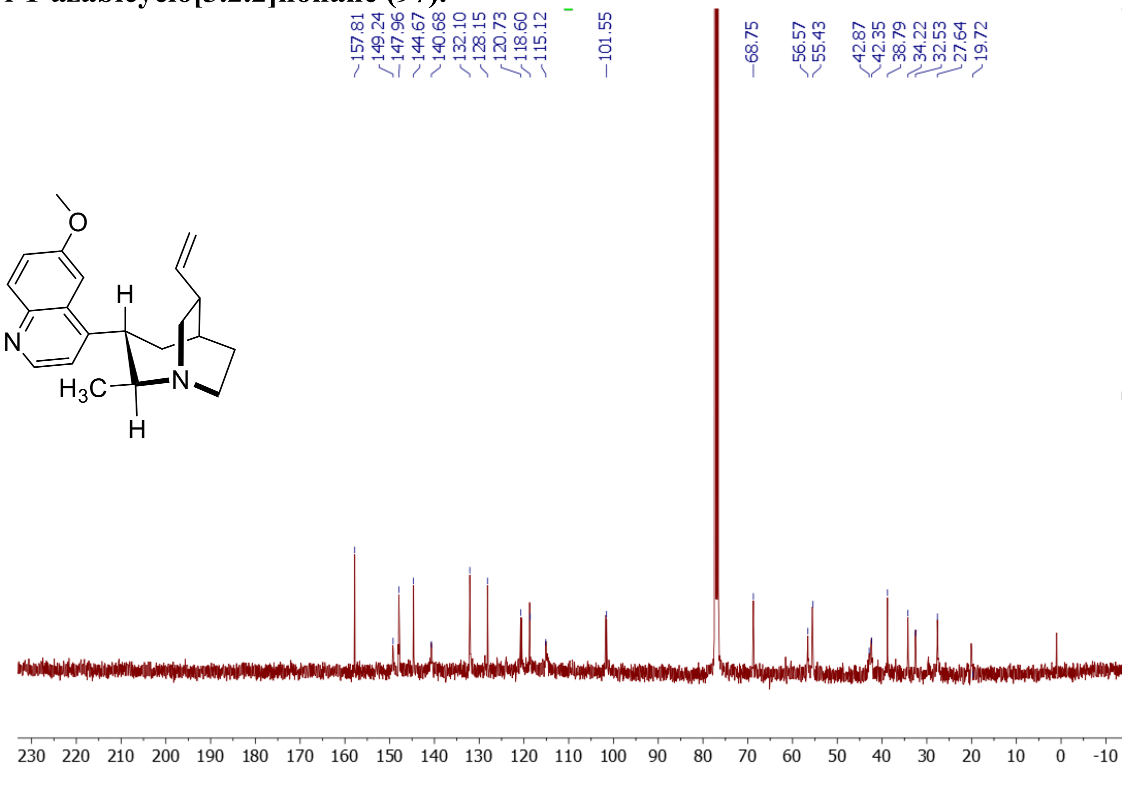

Figure S167. ^{1}H NMR spectrum of (3S,8S,9S,10R,13R,14S,17R)-10,13-dimethyl-17-((R)-6-methylheptan-2-yl)-3-phenyl-2,3,4,7,8,9,10,11,12,13,14,15,16,17-tetradecahydro-1H-cyclopenta[a]phenanthrene (99).

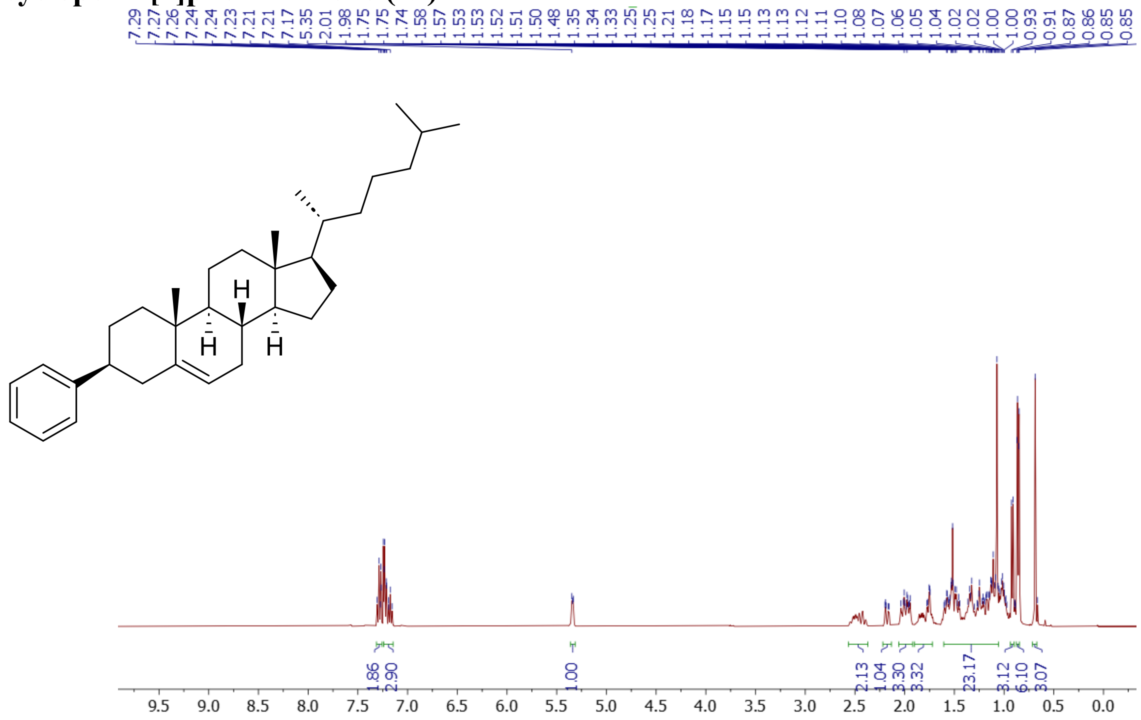

Figure S168. ^{13}C NMR spectrum of (3S,8S,9S,10R,13R,14S,17R)-10,13-dimethyl-17-((R)-6-methylheptan-2-yl)-3-phenyl-2,3,4,7,8,9,10,11,12,13,14,15,16,17-tetradecahydro-1H-cyclopenta[a]phenanthrene (99).

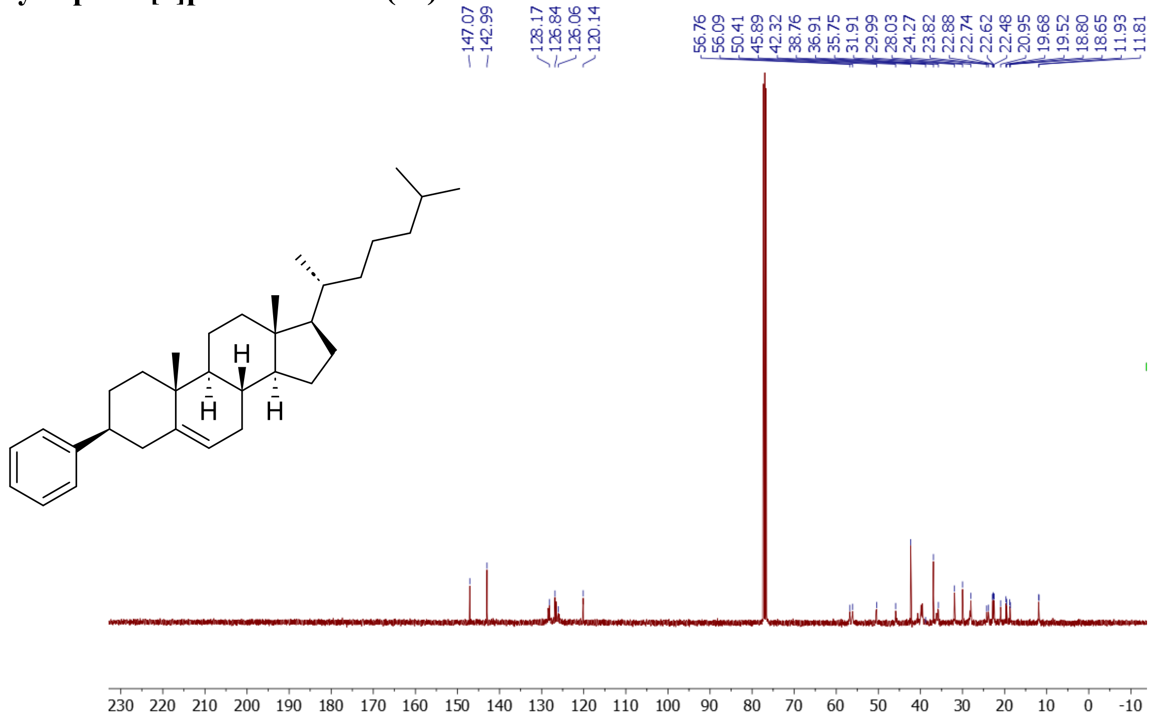

Figure S169. ^{1}H NMR spectrum of (1aR,3aR,3bS,5aR,6R,8aS,8bS,10R,10aR)-3a,5a,10-trimethyl-6-((*R*)-6-methylheptan-2-yl)hexadecahydrocyclopenta[a]cyclopropa[2,3]cyclopenta[1,2-f]naphthalene (100).

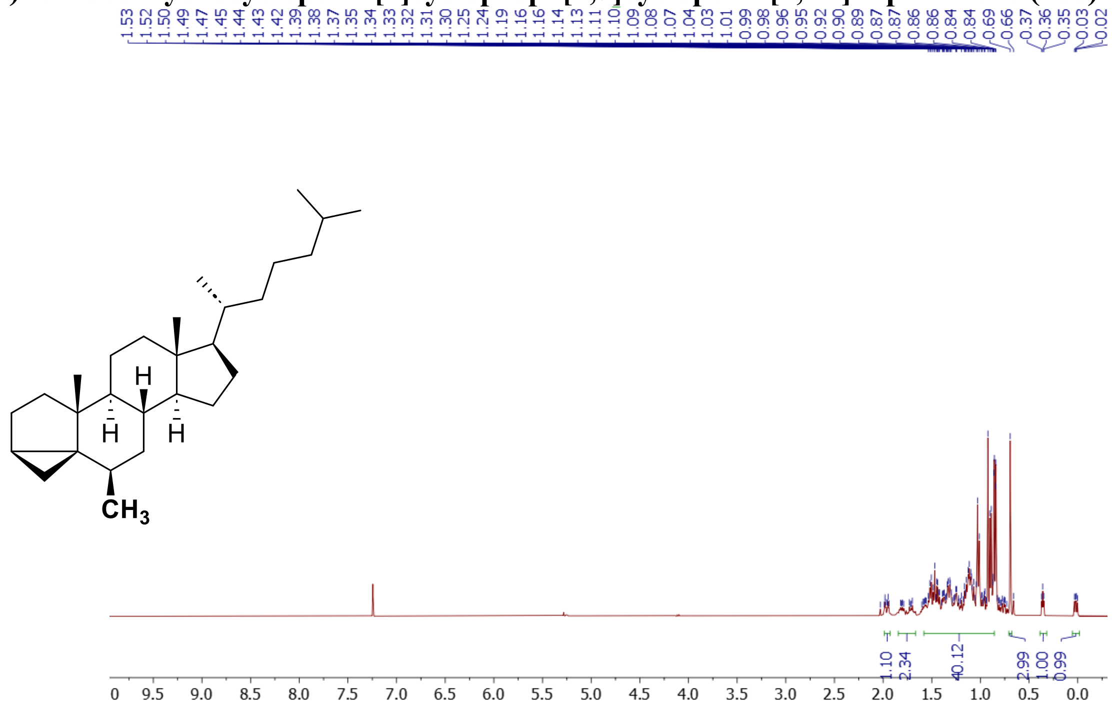

Figure S170. ^{13}C NMR spectrum of (1aR,3aR,3bS,5aR,6R,8aS,8bS,10R,10aR)-3a,5a,10-trimethyl-6-((R)-6-methylheptan-2-yl)hexadecahydrocyclopenta[a]cyclopropa[2,3]cyclopenta[1,2-f]naphthalene (100).

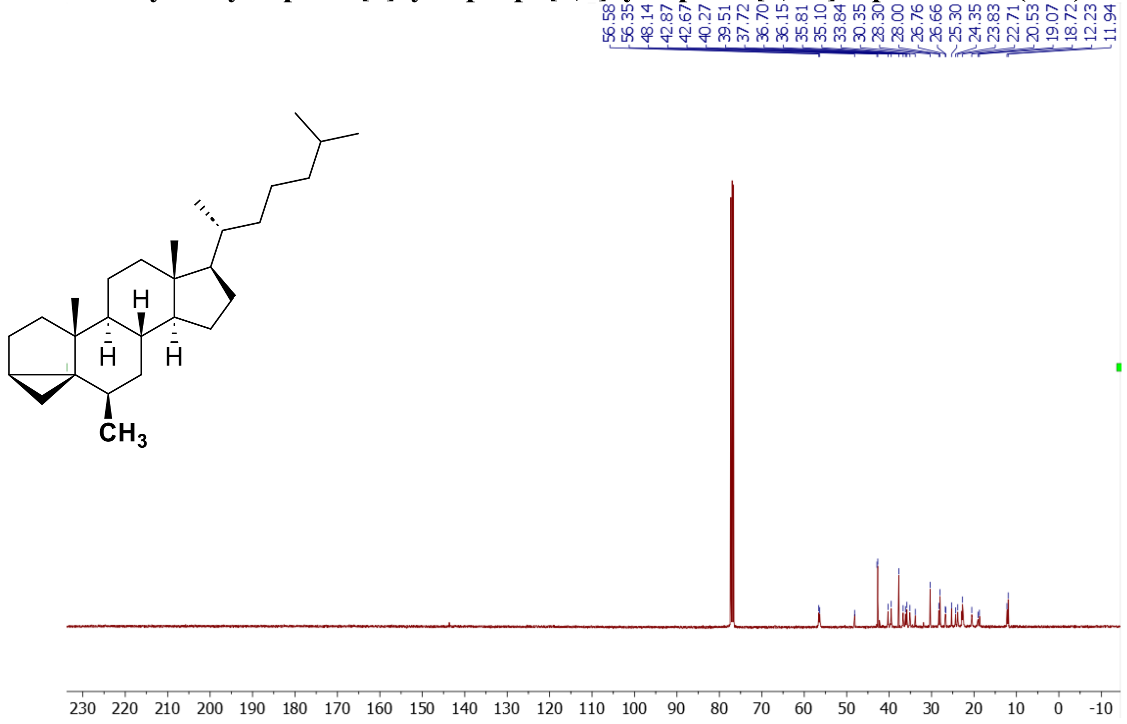

Figure S171. ^{1}H NMR spectrum of (3S,8S,9S,10R,13R,14S,17R)-10,13-dimethyl-17-((R)-6-methylheptan-2-yl)-3-(4-phenylbut-1-yn-1-yl)-2,3,4,7,8,9,10,11,12,13,14,15,16,17-tetradecahydro-1H-cyclopenta[a]phenanthrene (101).

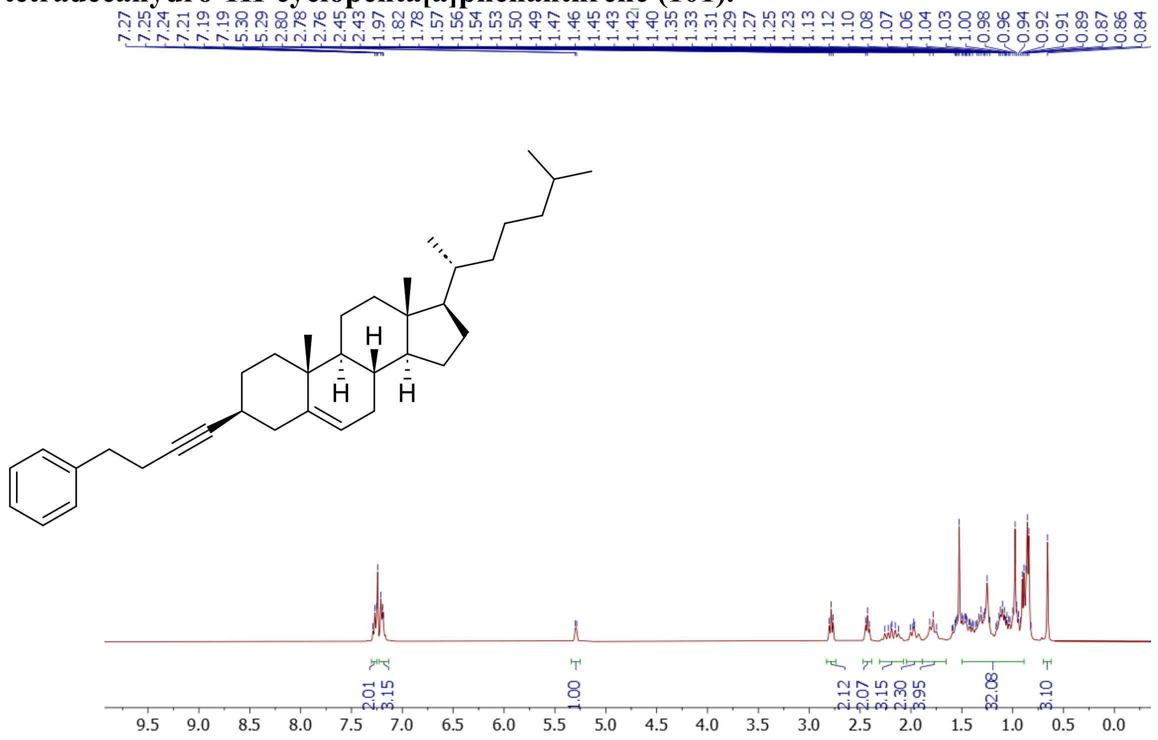

Figure S172. ^{13}C NMR spectrum of (3S,8S,9S,10R,13R,14S,17R)-10,13-dimethyl-17-((R)-6-methylheptan-2-yl)-3-(4-phenylbut-1-yn-1-yl)-2,3,4,7,8,9,10,11,12,13,14,15,16,17-tetradecahydro-1H-cyclopenta[a]phenanthrene (101).

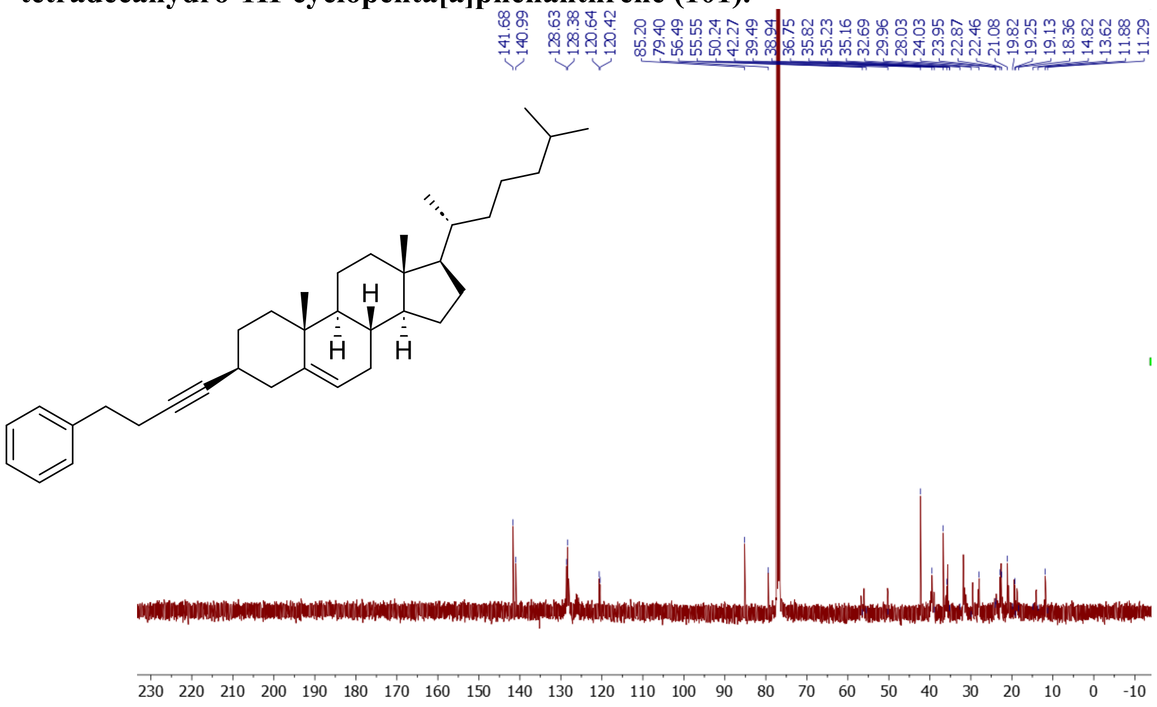

11. X-ray Crystallography

Single crystals of each compound were mounted under parabar oil on a Mitegen micromount and immediately placed in a cold nitrogen stream at 100(2) K prior to data collection. Data were collected a Bruker DUO equipped with an APEXII CCD detector and Mo fine-focus sealed source. Data were integrated with the Bruker SAINT program. Structure solution and refinement was performed using the SHELXTL/PC suite[35] and ShelXle.[36] Intensities were corrected for Lorentz and polarization effects and an empirical absorption correction was applied using Blessing's method as incorporated into the program SADABS.[37] Non-hydrogen atoms were refined with anisotropic thermal parameters. Hydrogen atoms were included in idealized positions unless otherwise noted.

X-ray structure of ion pair 27

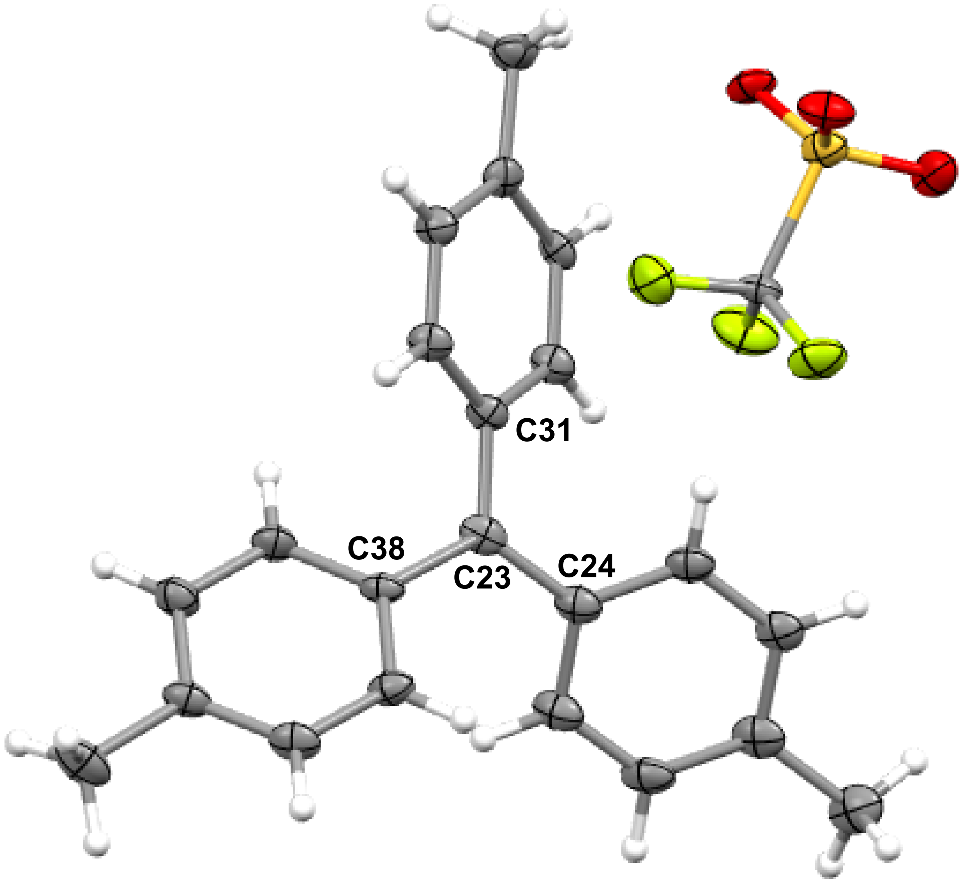

Figure S173. X-ray structure of ion pair **27**. Thermal ellipsoids are displayed at the 50% probability level. Only one out of two crystallographically distinct ion pairs are shown for clarity. Select bond lengths and angles: C23-C24 1.452(14) Å, C23-C31 1.461(15) Å, C23-C38 1.464(14) Å, C24-C23-C31 120.8°(9), C24-C23-C38 119.6°(9), C31-C23-C38 119.6°(9). Single crystals suitable for X-ray diffraction analysis were grown from a saturated dichloromethane solution layered with one volume of pentane at -40 °C. (CCDC 2313887)

X-ray structure of ion pair 27

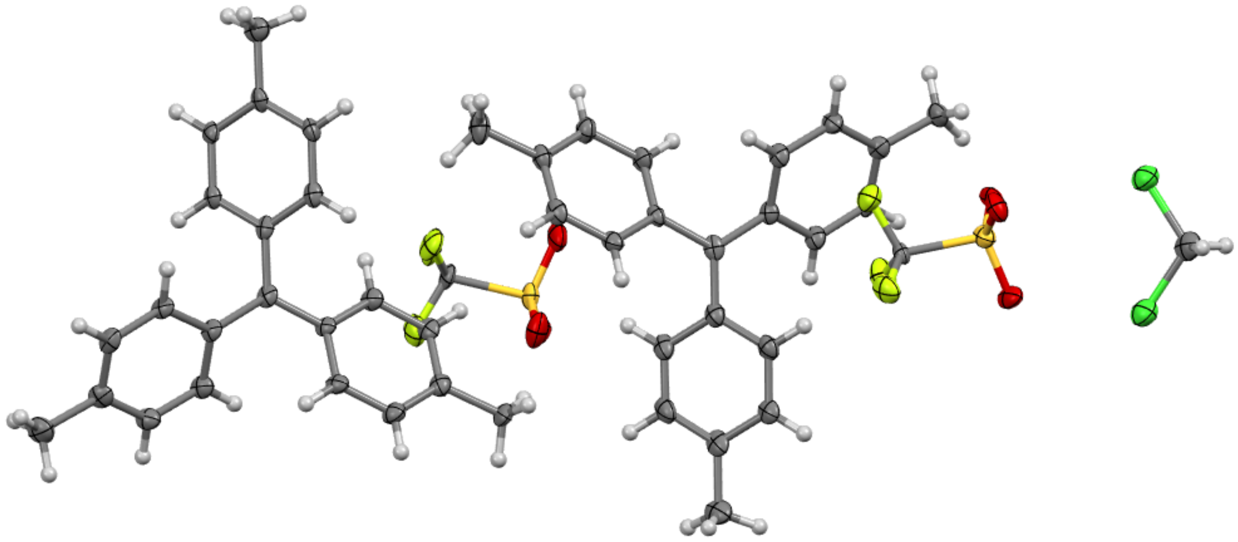

Figure S174. X-ray structure of ion pair **27**. Thermal ellipsoids are displayed at the 50% probability level. One triflate anion is disordered over two orientations. The like S-O, S-C, and C-F distances were restrained to be similar. The solvent molecule is disordered over two orientations. Sites of highest occupancy are shown for clarity. The like C-Cl distances were restrained to be similar. Rigid bond restraints were imposed on displacement parameters for all disordered sites and similar displacement amplitudes were imposed on disordered sites overlapping by less than the sum of van der Waals radii. Single crystals suitable for X-ray diffraction analysis were grown from a saturated dichloromethane solution layered with one volume of pentane at -40 °C.

X-ray structure of compound 28

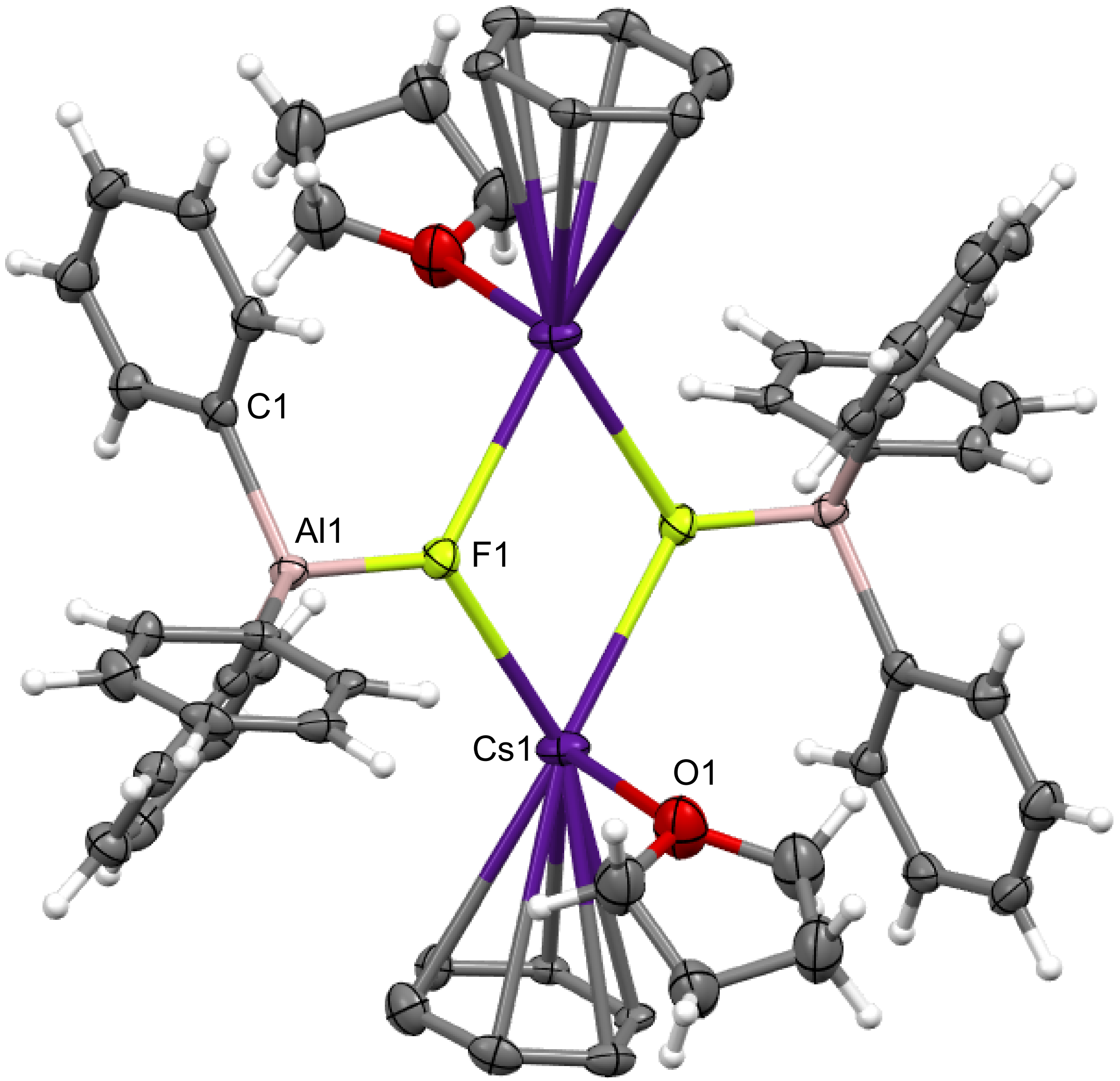

Figure S175. Dimeric X-ray structure of compound **28**. Thermal ellipsoids are displayed at the 50% probability level. Cesium atoms are coordinated η-6 to adjacent triphenylaluminum groups. The THF molecule is disordered and is shown in the orientation of highest occupancy for clarity. Select bond lengths and angles: Cs1-F1 2.922(3) Å, Al1-F1 1.739(3) Å, Al1-C1 1.991(5) Å, Al1-C13 1.994(5) Å, Al1-C7 2.001(5) Å, F1-Al1-C1 107.80°(17), Cs1-F1-Cs1 106.14°(8). Single crystals were grown from THF/Bu_2O solution layered with one volume of pentane at -40 °C.

X-ray structure of compound 28

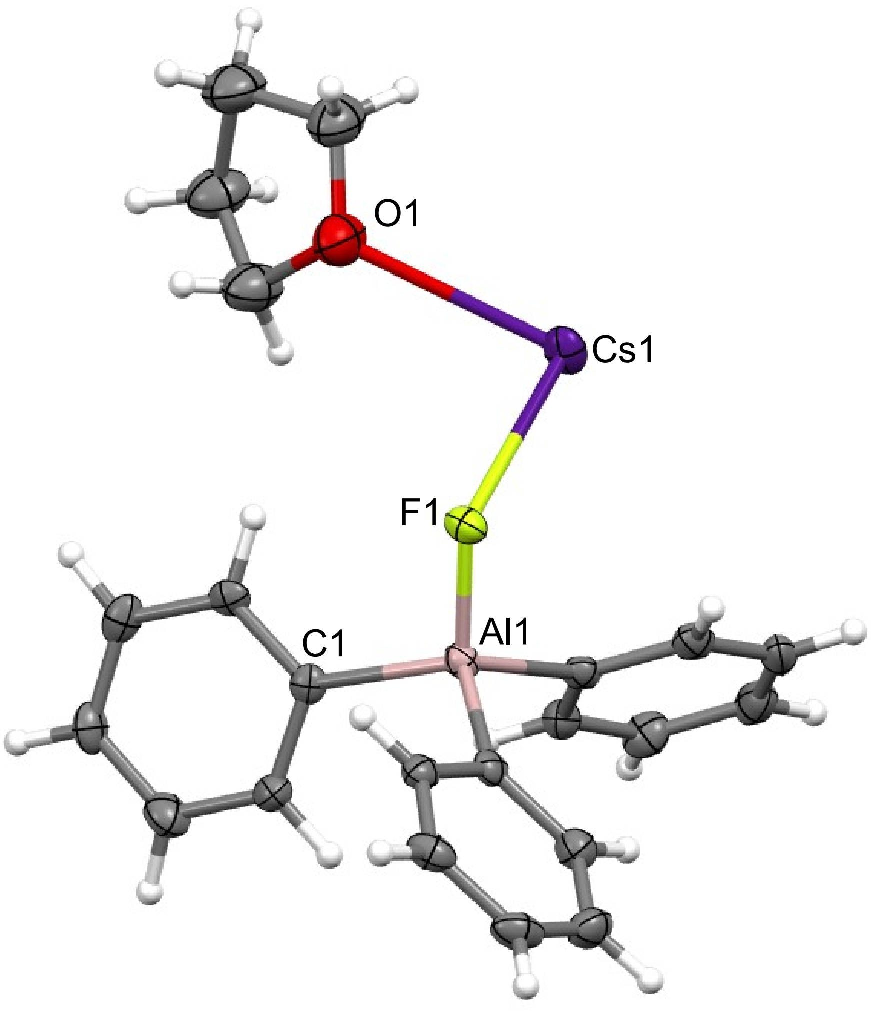

Figure S176. Asymmetric unit of compound **28.** Thermal ellipsoids are displayed at the 50% probability level. The disordered THF molecule is shown in the orientation of highest occupancy for clarity. Select bond lengths and angles: Cs1-F1 2.922(3) Å, Al1-F1 1.739(3) Å, Al1-C1 1.991(5) Å, Al1-C13 1.994(5) Å, Al1-C7 2.001(5) Å, F1-Al1-C1 107.80°(17). Single crystals were grown from THF/Bu_2O solution layered with one volume of pentane at -40 °C. (CCDC 2313889)

X-ray structure of 33

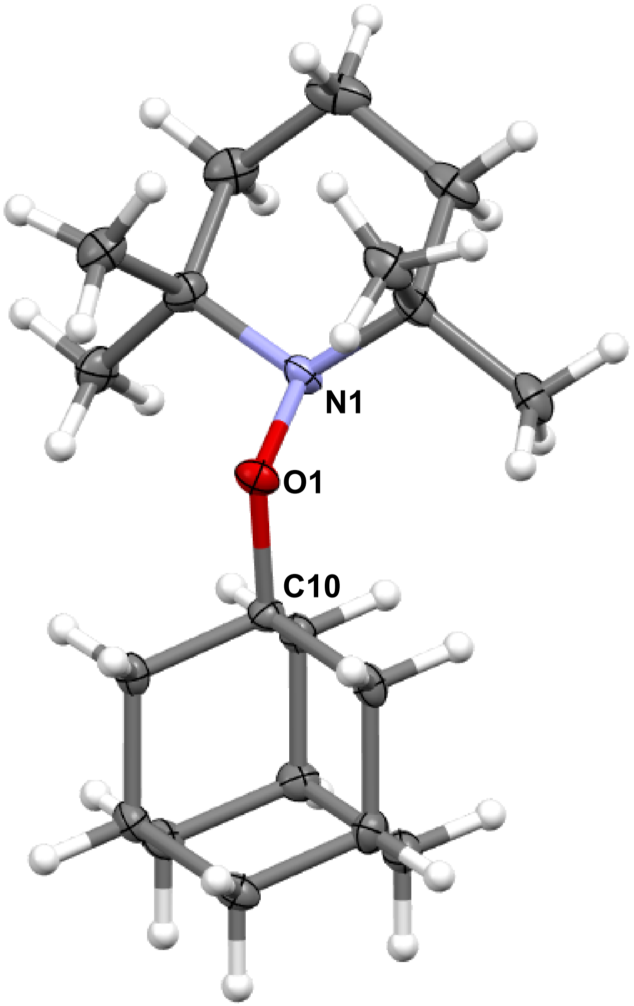

Figure S177. X-ray structure of compound **33.** Thermal ellipsoids are displayed at the 50% probability level. Select bond lengths and angles: C10-O1 1.469(2) Å, O1-N1 1.464(2) Å, N1-O1-C10 118.13°(15). Single crystals suitable for X-ray diffraction were grown from evaporation of a saturated pentane solution. (CCDC 2313888)

X-ray structure of compound 90

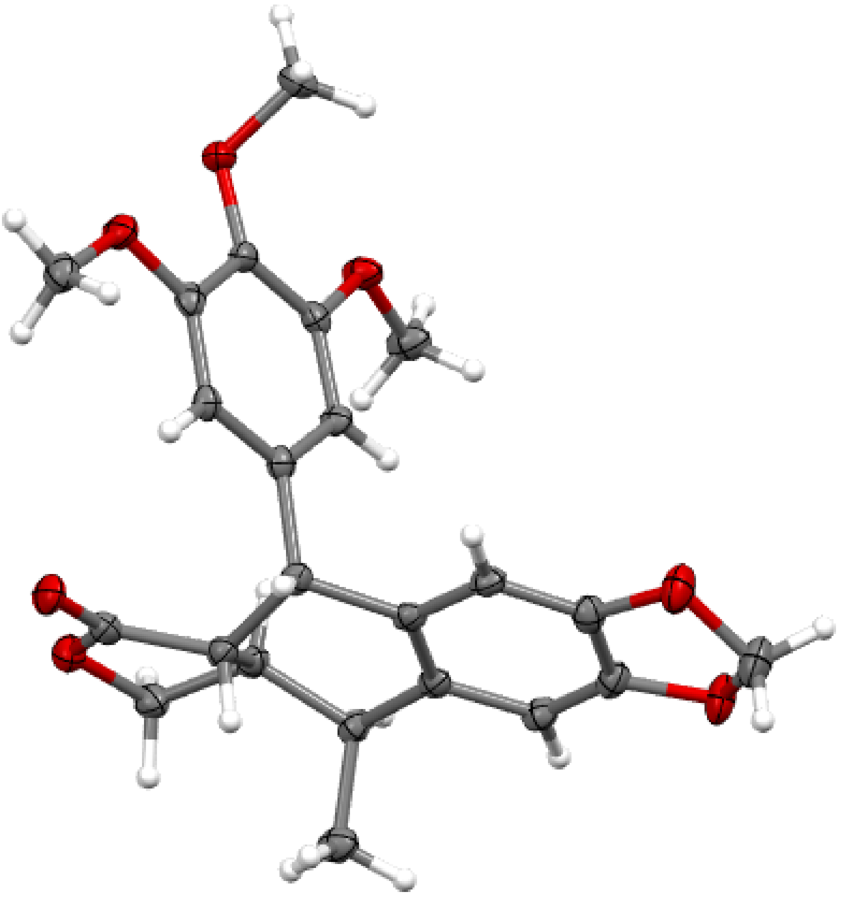

Figure S178. X-ray structure of compound **90.** Thermal ellipsoids are displayed at a 50% probability level. Single crystals were grown via slow evaporation of a saturated ethyl acetate solution. Select bond lengths (Å): C5-C14 1.536(3) Å, C5-C6 1.533(3) Å, C3-C5 1.530(3) Å, C3-C5-C6 108.65°(15), C3-C5-C14 113.76°(16), C6-C5-C14 111.22°(16). (CCDC 2362666)

X-ray structure of compound 99

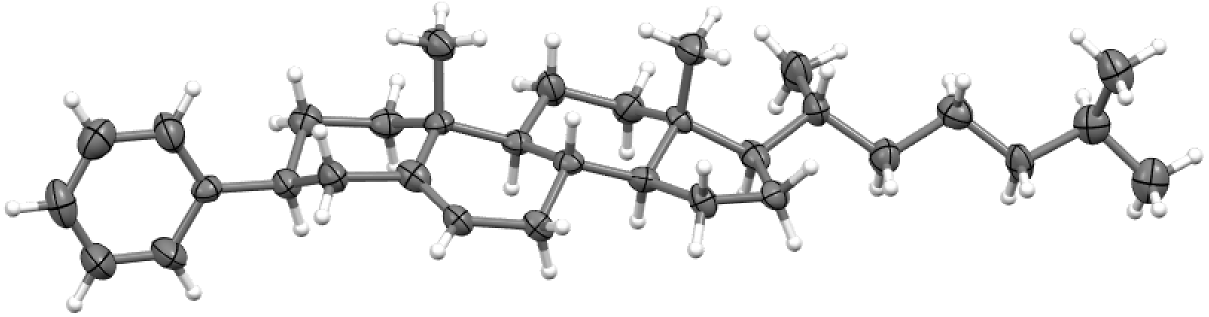

Figure S179. X-ray structure of compound **99.** Thermal ellipsoids are displayed at a 50% probability level. Single crystals were grown via slow evaporation of a saturated pentane solution. Select bond lengths (Å): C1-C2 1.519(10) Å, C1-C18 1.520(10) Å, C1-C17 1.546(11) Å, C2-C1-C18 113.6°(6), C2-C1-C17 108.5°(6), C18-C1-C17 111.4°(6). (CCDC 2362663)

X-ray structure of compound 100

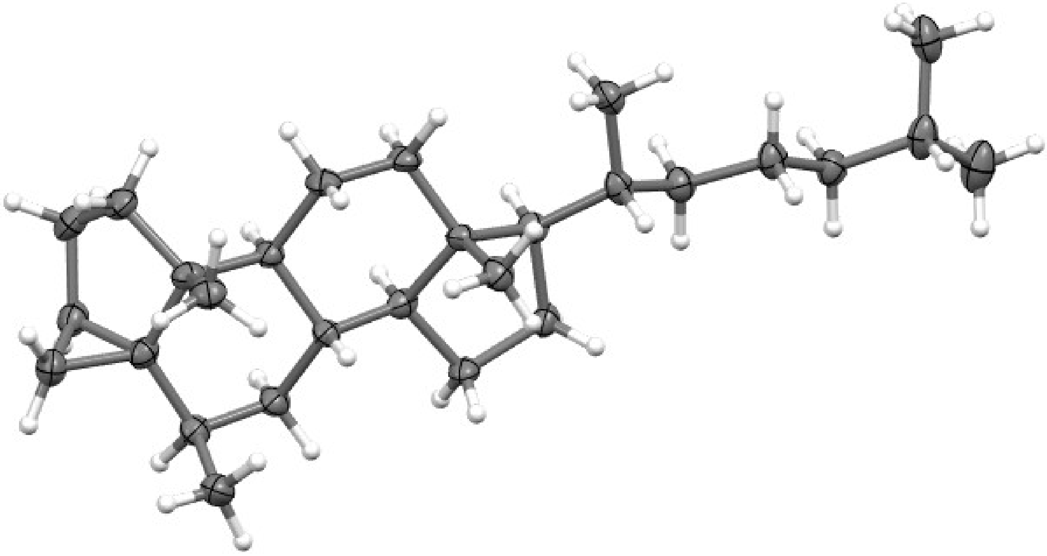

Figure S180. X-ray structure of compound **100.** Thermal ellipsoids are displayed at a 50% probability level. Single crystals were grown from a saturated diethyl ether solution at -10 °C.

Select bond lengths (Å): C1-C2 1.499(6) Å, C1-C3 1.512(6) Å, C2-C3 1.506(6) Å, C2-C1-C3 60.1°(3), C1-C2-C3 60.4°(3), C2-C3-C1 59.6°(3). (CCDC 2362664)

X-ray structure of compound 101

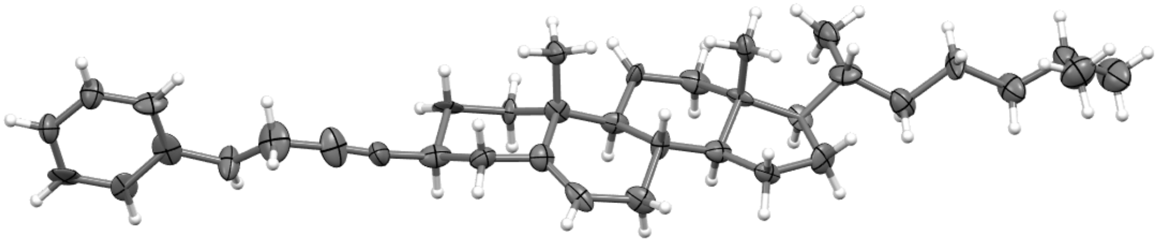

Figure S181. X-ray structure of compound **101.** Thermal ellipsoids are displayed at a 50% probability level. There are four molecules in the asymmetric unit, one molecule is shown for clarity. Three molecules in the asymmetric unit have disorder of the aryl ring, one orientation is shown for clarity. Single crystals were grown via slow evaporation of a saturated pentane solution. Select bond lengths (Å): C18-C19 1.19(2) Å, C19-C20B 1.48(2) Å, C19-C20 1.49(2) Å, C19-C18-C1 174°(2), C18-C19-C20 173°(2). (CCDC 2362665)

12. References

1. M. Stollenz, M. Barbasiewicz, A. J. Nawara-Hultzsch, T. Fiedler, R. M. Laddusaw, N. Bhuvanesh, J. A. Gladysz, Dibridgehead Diphosphines that Turn Themselves Inside Out. *Angew. Chem. Int. Ed.* **2011**, *50*, 6647-6651.

2. X. Yin, L. Zheng, A. Mohammadlou, B. R. Cagnon, W. D. Wulff, Resolution of Vaulted Biaryl Ligands via Borate Esters of Quinine and Quinidine. *J. Org. Chem.* **2020**, *85*, 10432-10450.

3. G. Bauer, M. D. Wodrich, R. Scopelliti, X. Hu, Iron Pincer Complexes as Catalysts and Intermediates in Alkyl–Aryl Kumada Coupling Reactions. *Organometallics* **2015**, *34*, 289-298.

4. B. C. Figula, D. L. Kane, K. Balaraman, C. Wolf, Organocuprate Cross−Coupling Reactions with Alkyl Fluorides. *Org. Lett.* **2022**, *24*, 8719-8723.

5. S. Jiang, Y. Xie, Y. Xie, L. Yu, X. Yan, F. Zhao, C. J. Madugamuwa, M. L. Coote, Z. Jia, K. Zhang, Lewis Acid-Induced Reversible Disproportionation of TEMPO Enables Aqueous Aluminum Radical Batteries. *J. Am. Chem. Soc.* **2023**, *145*, 14519-14528.

6. W. J. Middleton, New Fluorinating Reagents. Dialkylaminosulfur Fluorides. *J. Org. Chem.* **1975**, *40*, 574-578.

7. M. Tramontini, L. Angiolini, C. Fouquey, J. Jacques, Stereochemistry of Amino-Carbonyl Compounds – VII, Absolute and Relative Configuration of some Diastereomeric 1,3-Amino-Alcohols. *Tetrahedron.* **1973**, *29*, 4183-4187.

8. M. Pouloit, O. Mahe, J. Hamel, J. Desroches, J. Paquin, Halogenation of Primary Alcohols Using a Tetraethylammonium Halide/[Et_2NSF_2]BF_4 Combination. *Org. Lett.* **2012**, *14*, 5428-5431.

9. K. Balaraman, C. Wolf, Palladium and Nickel Catalyzed Suzuki Cross-Coupling with Alkyl Fluorides. *Org. Lett.* **2021**, *23*, 8994-8999.

10. S. Huo, Highly Efficient, General Procedure for the Preparation of Alkylzinc Reagents from Unactivated Alkyl Bromides and Chlorides**.** *Org. Lett.* **2003**, *5*, 423-425.

11. A. S. Sunagatulline, F. H. Lutter, P. Knochel, Preparation of Primary and Secondary Dialkylmagnesiums by a Radical I/Mg-Exchange Reaction Using sBu_2Mg in Toluene. *Angew. Chem.Int. Ed.* **2022**, *61*, e202116625.

12. W. F. Bailey, R. P. Gagnier, J. J. Patricia, Reactions of Tert-butyllithium with α,ω-Dihaloalkanes. Evidence for Single-Electron-Transfer-Mediated Metal-halogen Interchange Involving Alkyl Radical-halide Ion Adducts. *J. Org. Chem.* **1984**, *49*, 2098-2107.

13. K. Tamao, J. Yoshida, M. Akita, Y. Sugihara, T. Iwahara, M. Kumada, Organofluorosilicates in Organic Synthesis. XVI. Synthesis of Organopentafluorosilicates via the Diels-Alder, Ene, and Friedel-Crafts Reaction. Their Transformations to Organic Halides and Alcohols. *Bull. Chem. Soc. Jpn.* **1982**, *55*, 255-260.

14. H. Lv, L. Xiao, D. Zhao, Q. Zhao, Nickel(0)-Catalyzed Linear-Selective Hydroarylation of Unactivated Alkenes and Styrenes with Aryl Boronic Acids. *Chem. Sci.* **2018**, *33*, 6839-6843.

15. M. Uemura, H. Yorimitsu, K. Oshima, Synthesis of $Cp^*CH_2PPh_2$ and Its Use as a Ligand for the Nickel-Catalysed Cross-Coupling Reaction of Alkyl Halides with Aryl Grignard Reagents. *Chem. Commun.* **2006**, *45*, 4726.

16. M. Szostak, M. Spain, D. J. Proctor, Electron Transfer Reduction of Carboxylic Acids Using SmI_2-H_2O-Et_3N. *Org. Lett.* **2012**, *14*, 840-843.

17. M. Tobisu, T. Takahira, N. Chatani, Nickel-Catalyzed Cross-Coupling of Anisoles with Alkyl Grignard Reagents via C-O Bond Cleavage. *Org. Lett.* **2015**, *17*, 4352-4355.

18. E. R. Wearing, D. E. Blackmun, M. R. Becker, C. S. Schindler, 1- and 2-Azetines via Visible Light-Mediated [2+2]-Cycloadditions of Alkynes and Oximes. *J. Am. Chem. Soc.* **2021**, *143*, 16235-16242.

19. M. Sai, An Efficient $Ga(OTf)_3$/Isopropanol Catalytic System for Direct Reduction of Benzylic Alcohols. *Adv. Synth. Catal.* **2018**, *360*, 4330-4335.

20. Z. Zhang, D. Yadagiri, V. Gevorgyan, Light-Induced Metal-Free Transformations of Unactivated Pyridotriazoles. *Chem. Sci.* **2019**, *36*, 8399-8404.

21. R. Shinohara, N. Ogawa, H. Kawashime, K. Wada, S. Saito, T. Yamazaki, Y. Kobayashi, S_N2 Reaction of Diarylmethyl Anions at Secondary Alkyl and Cycloalkyl Carbons: S_N2 Reaction of Diarylmethyl Anions at Secondary Alkyl and Cycloalkyl Carbons. *Eur. J. Org. Chem.* **2019**, 1461-1478.

22. T. Saito, Y. Nishimoto, M. Yasuda, A. Baba, Direct Coupling Reaction between Alcohols and Silyl Compounds: Enhancement of Lewis Acidity of Me_3SiBr Using $InCl_3$. *J. Org. Chem.* **2006**, *71*, 8516-8522.

23. H. Mangunuru, C. Malapit, N. Haddad, J. Reeves, B. Qu, S. Rodriguez, H. Lee, N. Yee, J. Song, C. Busacca, C. Snanayake, Enantioselective Arylation of Oxindoles Using Modified BI-DIME Ligands. *Synthesis* **2018**, *50*, 4435-4443.

24. P. C. Too, G. H. Chan, Y. L. Tnay, H. Hirao, S. Chiba, Hydride Reduction by a Sodium Hydride-Iodide Composite. *Angew. Chem. Int. Ed.* **2016**, *55*, 3719-3723.

25. H. Miyamura, A. Suzuki, T. Yasukawa, S. Kobayashi, Polysilane-Immobilized Rh–Pt Bimetallic Nanoparticles as Powerful Arene Hydrogenation Catalysts: Synthesis, Reactions under Batch and Flow Conditions and Reaction Mechanism. *J. Am. Chem. Soc.* **2018**, *140*, 11325-11334.

26. T. Osako, K. Torii, A. Tazawa, Y. Uozumi, Continuous-flow Hydrogenation of Olefins and Nitrobenzenes Catalyzed by Platinum Nanoparticales Dispersed in an Amphiphilic Polymer. *RSC Adv.* **2015**, *5*, 45760-45766.

27. I. Chatterjee, M. Oestreich, Bronsted Acid-Catalyzed Transfer Hydrogenation of Imines and Alkenes Using Cyclohexa-1,4-dienes as Dihydrogen Surrogates. *Org. Lett.* **2016**, *18*, 2463-2466.

28. F. Liu, J. Zhong, S. Li, M. Li, L. Wu, Q. Wang, J Mao, S. Liu, B. Zheng, M. Wang, Q. Bain, Total Synthesis of (*R*)-Strongylodiols C and D. *J. Nat. Prod*. **2016**, *79*, 244-247.

29. J. Lu, E. Atochina-Vasserman, D. Maurya, D. Sahoo, N. Ona, E. Reagan, H. Ni, D. Weissman, V. Percec, Targeted and Equally Distributed Delivery of mRNA to Organs with Pentaerythritol-Based One-Component Ionizable Amphiphilic Janus Dendrimers. *J. Am. Chem. Soc.* **2023**, *145*, 18760-18766.

30. X. Li, J. Jin, P. Chen, G. Liu, Catalytic Remote Hydrohalogenation of Internal Alkenes. *Nat. Chem.* **2022**, *14*, 425-432.

31. S. Du, E. Kimball, J. Ragains, Visible-Light-Promoted Remote C-H Functionalization of *o*-Diazoniaphenyl Alkyl Sulfones. *Org. Lett.* **2017**, *19*, 5553-5556.

32. D. L. Kane, B. C. Figula, K. Balaraman, J. A. Bertke, C. Wolf, General Alkyl Fluoride Functionalization via Short-lived Carbocation-organozincate Ion Pairs. *Nat. Commun*. **2024**, *15*, 1866.

33. G. Bauer, C. W. Cheung, X. Hu, Cross-coupling of Non-activated Primary and Secondary Alkyl Halides with Aryl rignard Reagents Catalyzed by Chiral Iron Pincer Complexes. *Synthesis* **2015**, *47*, 1726-1732.

34. G. H. Posner, J.-S. Ting, C. M. Lentz, A Mechanistic and Synthetic Study of Organocopper Substitution Reactions with Some Homoallylic and Cyclopropylcarbinyl Substrates: Application to Isoprenoid Synthesis. *Tetrahedron* **1976**, *19*, 2281-2287.
35. G. M. Sheldrick, Crystal Structure Refinement with SHELXL. *Acta Cryst*. **C71**, 3-8 (2015).
36. C. B. Hubschle, G. M. Sheldrick, B. Dittrich, ShelXle: a Qt graphical user interface for SHELXL. *J. Appl. Cryst.* **2011**, *44*, 1281-1284.
37. Bruker **2016**. APEX3, SADABS, SAINT, SHELXTL, XCIF, XPREP. Bruker AXS, Inc., Madison, Wisconsin, USA.
